# Ir^III^‐Catalyzed Selective *ortho*‐Monoiodination of Benzoic Acids with Unbiased C−H Bonds

**DOI:** 10.1002/chem.202002204

**Published:** 2020-07-27

**Authors:** Erik Weis, Magnus J. Johansson, Belén Martín‐Matute

**Affiliations:** ^1^ Department of Organic Chemistry Stockholm University Stockholm 10691 Sweden; ^2^ Medicinal Chemistry, Research and Early Development Cardiovascular, Renal and Metabolism (CVRM) BioPharmaceuticals R&D AstraZeneca Gothenburg Sweden

**Keywords:** C−H activation, iodination, iridium, *ortho*-functionalization, selectivity

## Abstract

An iridium‐catalyzed selective *ortho*‐monoiodination of benzoic acids with two equivalent C−H bonds is presented. A wide range of electron‐rich and electron‐poor substrates undergo the reaction under mild conditions, with >20:1 mono/di selectivity. Importantly, the C−H iodination occurs selectively *ortho* to the carboxylic acid moiety in substrates bearing competing coordinating directing groups. The reaction is performed at room temperature and no inert atmosphere or exclusion of moisture is required. Mechanistic investigations revealed a substrate‐dependent reversible C−H activation/protodemetalation step, a substrate‐dependent turnover‐limiting step, and the crucial role of the Ag^I^ additive in the deactivation of the iodination product towards further reaction.

## Graphical abstract

**Selectivity in focus**: A methodology for the selective *ortho*‐monoiodination of sterically unbiased benzoic acids is reported. A>20:1 mono/di selectivity was achieved with a series of diverse substrates (see scheme). High regioselectivity was observed with compounds bearing other coordinating directing groups. Mechanistic investigations revealed a crucial role of the Ag^I^ additive.

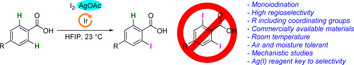

The introduction of functional groups through the direct replacement of C−H bonds is undoubtedly a powerful strategy in synthetic organic chemistry.^1^ C−H bonds are ubiquitous in organic compounds, and in general several C−H bonds with similar properties (i.e., bond strength and accessibility) are present within a compound.1a, 2 The selective functionalization of a single specific C−H bond within a complex molecule containing similar or even equivalent C−H bonds is still a major challenge. If an organic substrate contains a coordinating functional group that is able to interact with a transition‐metal complex, selective activation of C−H bonds in close proximity to this group can be achieved. For C(sp^2^)−H bonds in aromatic systems, it is usually the C−H bonds in the *ortho* positions relative to the coordinating group that is activated.^3^ Alternative strategies have been developed for the activation of C−H bonds in the *meta* and *para* positions.^4^ Despite these advances, it is usually still difficult to achieve selectivity when two or more equivalent C−H bonds are found at the right distance from the directing group. This is the case for symmetrical aromatic compounds that contain two equivalent C−H bonds in the positions *ortho* to the directing group. Often both *ortho*‐C(sp^2^)−H bonds can be activated and functionalized under the reaction conditions. In unsymmetrical substrates where two non‐equivalent *ortho*‐C(sp^2^)−H bonds are present, overfunctionalization can be avoided by the introduction of functional groups close to one of the C−H bonds (e.g. in aromatic systems *meta* to the directing group).^5^ An alternative catalytic approach in which the catalyst shows a high level of inherent selectivity for the functionalization of only one of the equivalent C−H bonds is undoubtedly more attractive.^6^ C−H‐activation protocols can give direct access to aryl halides, which are important compounds in synthetic organic chemistry. A number of directed C−H *ortho*‐monohalogenations have been reported by the Bedford,^7^ Yu,^8^ Tian,^9^ Koley,^10^ Glorius,^11^ Sanford,^12^ Gevorgyan,^13^ and Rao^14^ groups. While the selectivity is often dependent on the nature of the directing group, as demonstrated in the excellent work from the Glorius group,11a mono/di‐selectivity in certain systems can be improved by careful tuning of reaction conditions.^7^, 8b, 15 However, experimental evidence of the precise cause of selectivity is often limited. As part of our ongoing research, we have been interested in using benzoic acids for the synthesis of highly functionalized aromatic compounds.^16^ Methods that allow direct use of abundant, commercially available benzoic acids as substrates and avoid the use of protecting groups and designer directing groups are highly advantageous.8b, 16, 17 Pioneering work by Yu has shown that benzoic acids undergo efficient *ortho*‐C−H halogenation under Pd catalysis.8b Selective monobromination was achieved with a variety of *meta*‐ and *para*‐substituted substrates (Scheme 1, top), while iodination of sterically unbiased substrates resulted in the formation of the diiodinated products. More recently, we reported a method for the *ortho*‐iodination of benzoic acids using Ir‐catalysis.^16^ This method performed well under mild conditions but showed no selectivity towards monoiodination of substrates with unbiased *ortho*‐C−H bonds.

**Scheme 1:**
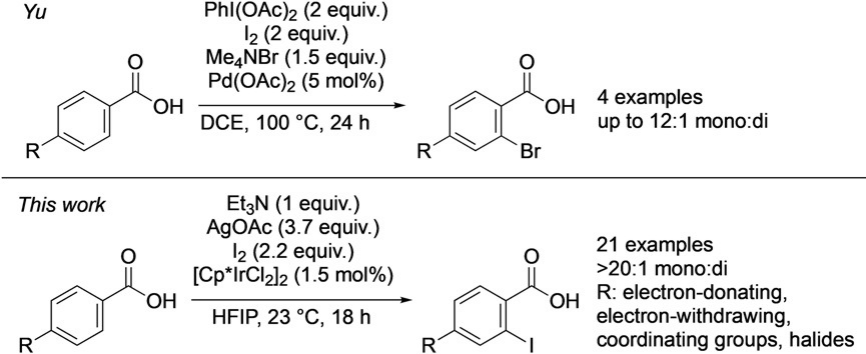
o*rtho*‐C−H monohalogenations of *para*‐substituted benzoic acids.

In this article, we set out to address the well‐established, yet challenging selectivity issue of mono‐ over disubstitution in the activation and functionalization of unbiased *ortho*‐C−H bonds. We report the *ortho*‐iodination of benzoic acids with outstanding levels of selectivity towards monoiodination (Scheme 1, bottom). Furthermore, we showcase excellent regioselectivitiy on substrates where multiple directing groups are present, which underlines the synthetic utility of the present study.

Based on our previous results,^16^ we began the optimization work using benzoic acid (**1 a**) as a model substrate, *N*‐iodosuccinimide (NIS) as the iodine source, and [Cp*Ir(H_2_O)_3_]SO_4_ (**I**) as catalyst, with 1,1,1,3,3,3‐hexafluoroisopropanol (HFIP) as the solvent. At room temperature, 45 % yield of products **2 a** and **2 a′** was obtained, with a ratio of 8:1 in favor of **2 a** (Table 1, entry 1).When potassium benzoate was used instead as the substrate, a decreased yield was observed, albeit with excellent selectivity (25 % yield, **2 a**/**2 a′** >20:1, Table 1, entry 2). As NIS decomposition was observed under these conditions, iodine monoacetate (AcOI), generated in situ from I_2_ and AgOAc,^18^ was tested as an alternative iodine source. AcOI generation in this fashion in the C−H activation context was previously reported by the Yu group.^19^ This led to a significant increase in yield, while maintaining high selectivity (Table 1, entry 3). Increasing the amount of AgOAc further improved the selectivity (Table 1, entry 4). A number of other silver sources were tested (Table S4), but only Ag_2_CO_3_ showed comparable results to AgOAc in terms of selectivity, albeit with a somewhat lower yield (Table 1, entry 5 vs. entry 4). No reaction took place in the absence of silver (Table 1, entry 6). We tested various other solvents (Table 1, entry 7, Table S3), however, HFIP proved to be crucial. When the commercially available [Cp^*^IrCl_2_]_2_ complex (**II**) was used (Table 1, entry 8), similar results were obtained as with aqua complex **I**. Therefore, complex **II** was used for further optimization. Interestingly, when the amount of I_2_ was increased, a significant loss of selectivity was observed (Table 1, entry 9). When the amount of AgOAc was further increased, the high selectivity was restored (Table 1, entry 10). Thus, an excess of AgOAc is crucial for the selectivity. The reaction worked well with benzoic acid as the substrate when a stoichiometric amount of Et_3_N was added (Table 1, entry 11). In the absence of Et_3_N, the conversion of **1 a** was significantly lower (68 %, Table S6). Finally, no reaction took place in the absence of the iridium precatalyst (Table S6).

**Table 1:** Optimization of the reaction conditions.^[a]^

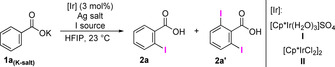						
Entry	Solv.	Ag salt (equiv)	I source (equiv)	[Ir]	Yield [%]	**2 a**/**2 a′**
1^[b,c]^	HFIP	–	NIS (1.5)	**I**	45	8:1
2^[b]^	HFIP	–	NIS (1.5)	**I**	25	>20:1
3	HFIP	AgOAc (1.3)	I_2_ (1.2)	**I**	91	13:1
4	HFIP	AgOAc (2.2)	I_2_ (1.2)	**I**	89	>20:1
5	HFIP	Ag_2_CO_3_ (1.2)	I_2_ (1.2)	**I**	85	>20:1
6	HFIP	–	I_2_ (1.2)	**I**	0	
7	TFE	AgOAc (1.3)	I_2_ (1.1)	**I**	51	2:1
8	HFIP	AgOAc (2.2)	I_2_ (1.1)	**II**	92	>20:1
9	HFIP	AgOAc (2.5)	I_2_ (2.0)	**II**	>95	10:1
10	HFIP	AgOAc (3.7)	I_2_ (2.2)	**II**	>99	>20:1
11^[d]^	HFIP	AgOAc (3.7)	I_2_ (2.2)	**II**	>99	>20:1

[a] Unless otherwise noted, BzOK (0.25 mmol), Ir (3 mol %), AgOAc, I_2_, HFIP as solvent (0.1 m), at rt (23 °C). The product distribution and yield were determined by ^1^H NMR spectroscopy. [b] NIS (1.5 equiv), [Cp*Ir(H_2_O)_3_]SO_4_ (3 mol %), HFIP (0.1 m), rt, 18 h. [c] BzOH instead of BzOK. [d] BzOH/ Et_3_N (0.25 mmol) instead of BzOK.

With the optimized reaction conditions (Table 1, entry 11), we went on to study the scope of the reaction. A wide range of *para*‐substituted benzoic acids were iodinated with excellent selectivity in favor of iodination of only one of the equivalent *ortho*‐C−H bonds. In all cases, with the exception of **1 d** (Scheme 2), the mono*/*diiodination ratio was >20:1. Benzoic acids **1 b**–**1 d** reacted to give **2 b**–**2 d** within 2 h. Additional functional groups such as the benzylic primary sulfonamide in **1 e**, the aliphatic alcohol in **1 f** or the acetate group in **1 g** did not interfere with the reaction outcome. Aryl halides were also compatible with the reaction conditions and the synthetically attractive dihalogenated compounds **2 h**–**2 j** were successfully obtained. Electron‐withdrawing groups such as trifluoromethoxy, trifluoromethyl, and nitro groups were well tolerated, as seen in the synthesis of compounds **2 k**–**2 m**.

**Scheme 2:**
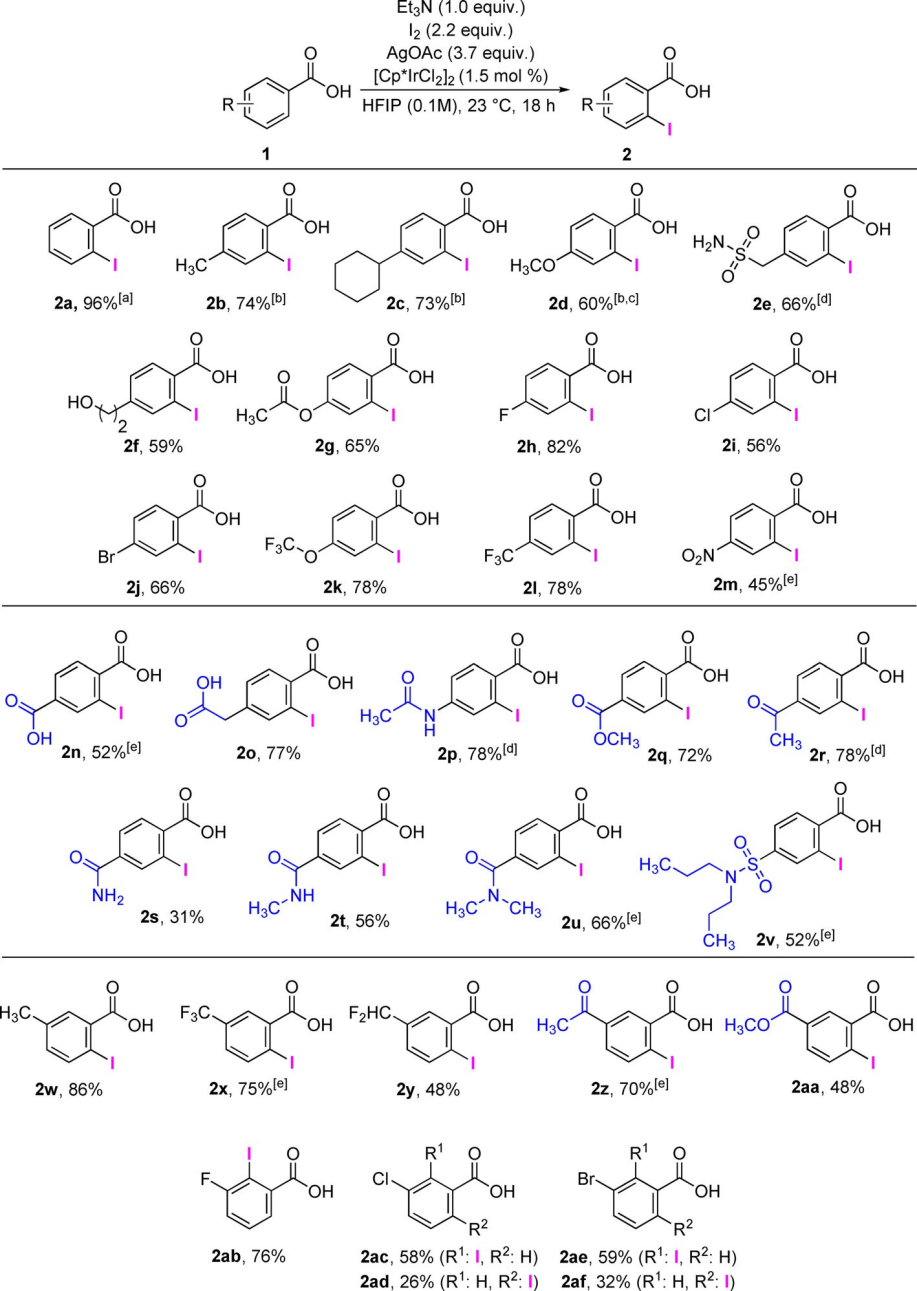
Substrate scope. Isolated yields are shown. Competing directing groups highlighted in blue. [a] Isolated as mono/disubstituted mixture, >20:1 mono/di ratio. [b] Reaction time 2 h. [c] Mono/di ratio 14:1. [d] [Cp*IrCl_2_]_2_ (2.5 mol %), AgOAc (3.8 equiv). [e] [Cp*IrCl_2_]_2_ (3.0 mol %), AgOAc (3.8 equiv).

A very important and largely unexplored feature in directed C−H activations are systems containing directing groups in the same substrate. High regioselectivity was observed in the presence of coordinating groups commonly used as directing groups in C−H functionalizations (Scheme 2, center).^20^ Interestingly, terephthalic acid gave monoiodinated product **2 n** rather than the expected 2,5‐diiodoterephthalic acid. The carboxylate and acetamide substituent were well tolerated, and compounds **2 o** and **2 p** were obtained with no iodination observed *ortho* to these coordinating groups. Similarly, neither the ester group in **1 q** nor the ketone group in **1 r** served as a competing directing group. Even strongly coordinating functional groups such as the primary, secondary and tertiary amides in **1 s**–**1 u** were tolerated. These substrates were iodinated with high regioselectivity, exclusively *ortho* to the carboxylic functional group. The method was also successfully applied to the late‐stage functionalization (LSF) of probenecid, and the iodinated analogue **2 v** was obtained in a synthetically useful yield.

Although this method was developed for the selective monoiodination of *para*‐substituted benzoic acids, we also tested a series of *meta*‐substituted benzoic acids (Scheme 2, bottom). Under the same conditions, 3‐toluic acid **1 w** gave **2 w** in high yield. Electron‐poor fluorinated products **2 x** and **2 y** were also readily obtained. Yet again, coordinating groups as in **1 z** and **1 aa** did not interfere with the expected regioselectivity. On the other hand, 3‐fluorobenzoic acid was iodinated selectively in the 2‐position to give **2 ab**, consistent with previously published results.^16^ When 3‐chloro‐ and 3‐bromobenzoic acids were subjected to the reaction conditions, regioisomeric mixtures of monoiodinated products **2 ac**–**2 af**, with the iodo‐substituent at the 2‐ or 6‐position, were obtained. The more sterically congested 2‐iodo compound was the major product in both cases. The observed regioselectivity is likely the result of an interaction between the catalyst and the electron pairs of the Lewis base substituents in the 3‐position.

To understand the origin of the high selectivity for monosubstitution a mechanistic investigation was conducted. The kinetic profile (Figure 1) revealed that only trace amounts of the diiodinated product **2 a′** were formed, even after near complete depletion of **1 a**. Thus, the high selectivity arises from the very large difference in the reaction rates of the first iodination (to form **2 a** from **1 a**) and the second iodination (to form **2 a′** from **2 a**). The kinetic profile also shows a remarkable rate, as 50 % yield of **2 a** is obtained after only 4.5 min; and after 30 min, the yield is 92 %.

**Figure 1:**
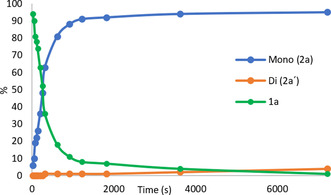
Kinetic profile: iodination of **1 a** under standard conditions.

Next, we studied the reversibility of the C−H activation step. An acetate‐assisted concerted metalation‐deprotonation (CMD) pathway for a similar iridium‐catalyzed C−H activation was previously proposed and calculated by Ison.^21^ Compound **1 a** was stirred in HFIP‐*d* under otherwise standard conditions for the iodination reaction (Scheme 3 a). After 5 minutes, we observed a 55 % conversion to **2 a** with significant D incorporation at the *ortho*‐positions in both recovered **1 a** and in **2 a**. Deuterium incorporation at *meta* or *para* was not observed (Figure S2). This result supports the idea that the iridacycle (Scheme 3 a, **Ic‐1** *)* formed via C−H activation does undergo protodemetalation under the reaction conditions, and consequently, that the formation of this iridacycle is reversible. Deuterium incorporation also occurred when **1 a** was subjected to the reaction conditions in the absence of I_2_, forming a mixture of non‐deuterated, mono‐deuterated and dideuterated **1 a** (45 % D total, Scheme 3 b; 24 % **1 a**‐D1 and 34 % **1 a**‐D2, Table S8).

**Scheme 3:**
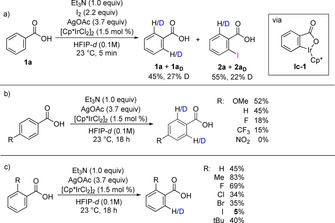
Selected mechanistic studies.

The D incorporation, and thus the reversibility of the C−H activation, is highly dependent on the nature and position of the substituents on the benzoic acids **1** (Scheme 3 b and c). Significant decrease of D incorporation was observed with substrates bearing electron‐withdrawing groups in the *para*‐position (Scheme 3 b) such as the trifluoromethyl group in **1 l** (15 % D) or nitro in **1 m** (no D incorporation). With *ortho*‐substituents present, a general decrease in D incorporation was observed (Scheme 3 c). Importantly, when 2‐iodobenzoic acid **2 a** was subjected to the reaction conditions, only 5 % D was incorporated, significantly less then with the sterically demanding *tert*‐butyl substituent (40 %, Scheme 3 c). This indicates that the formation of an iridacycle from **2 a** is inhibited, which agrees well with the observed selectivity for monoiodination. In our previous work,^16^ the rate of iodination of **1 a** was similar to that of **2 a**, and Ag^I^ salts were not used as additives. As the AgOAc loading was shown to be crucial to obtaining high selectivity (vide supra, Table 1), control experiments were carried out to understand its role (Scheme 4).

**Scheme 4:**
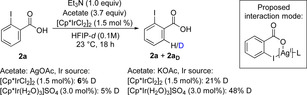
Ag^I^ effect on D incorporation. Comparison experiments between AgOAc and KOAc additives.

We observed that D was only incorporated in **2 a** to a small extent when AgOAc was used, while a significant increase in D incorporation was observed with KOAc. Thus, we can conclude that Ag inhibits the activation of the remaining *ortho*‐C−H bond in **2 a** under these conditions. A possible explanation is that silver forms a coordination complex with the carboxylate group of **2 a**, inhibiting further reaction (Scheme 4). This is consistent with the significantly lower rate of iodination of **2 a** compared with **1 a** (Figure 1).

Next, we turned our attention towards identifying the turnover‐limiting step (TLS) by measuring kinetic isotope effects^22^ (KIE, Scheme 5). Two sets of benzoic acids, **1 a/1 a‐D5** and 4‐(trifluoromethyl) benzoic acid **1 l/1 l‐D2** were subjected to the reaction conditions, using a parallel experiment set‐up (Figures S2–S5). A KIE of 2.19 was obtained for **1 a/1 a‐D5**. Its relatively low magnitude, and the fact that the C−H activation step for this substrate is reversible, indicates that the C−H activation is only partially rate determining for **1 a**. On the other hand, for **1 l/1 l‐D2**, a KIE of 4.95 was obtained, indicating that the C−H bond is broken in the turnover‐limiting step. This agrees well again with the D incorporation study (Scheme 3 b), where a significantly lower incorporation of D was observed for substrates bearing electron‐withdrawing substituents at the *para*‐position. Thus, the TLS in the catalytic cycle is proposed to be dependent on the electronic properties of the substrate.

**Scheme 5:**
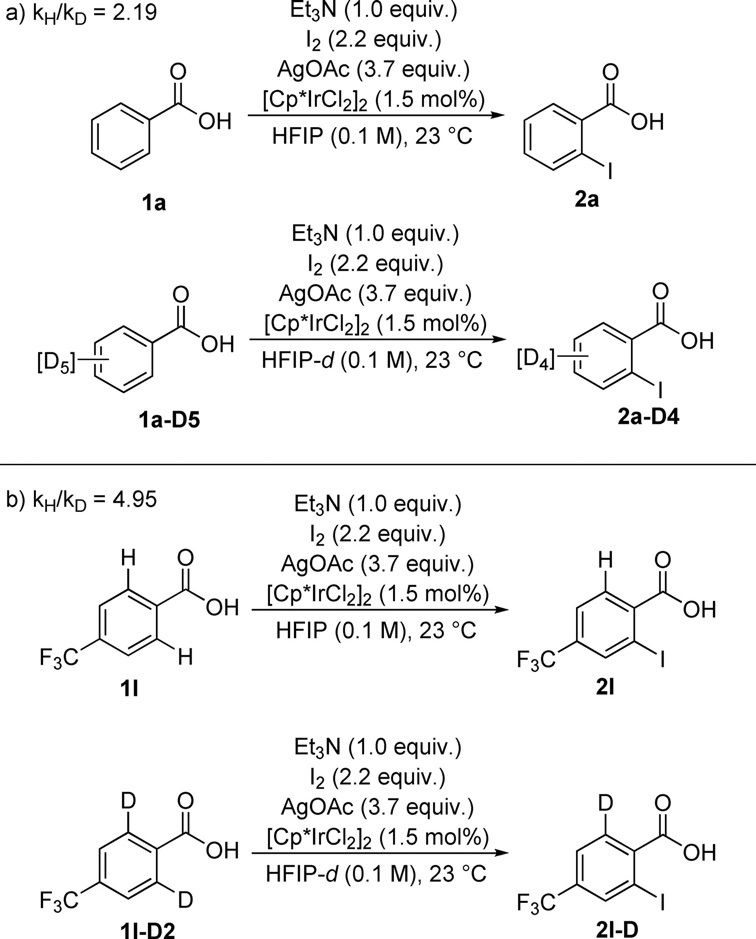
KIE studies.

A catalytic cycle is proposed in Scheme 6. After activation of the precatalyst through formation of an acetate‐iridium(III) complex and ligand exchange, intermediate **INT1** is formed. ^13^C NMR studies indicate that the resting state for **1 a** is a complex consisting of the benzoate anion of **1 a** coordinated to Ir (see Supporting Information). The C−H activation step occurs through an acetate‐assisted CMD step.^16^, ^21^ This step is essentially irreversible for electron‐poor substrates (e.g., **1 l**), and it is also the TLS of the reaction for these substrates.

**Scheme 6:**
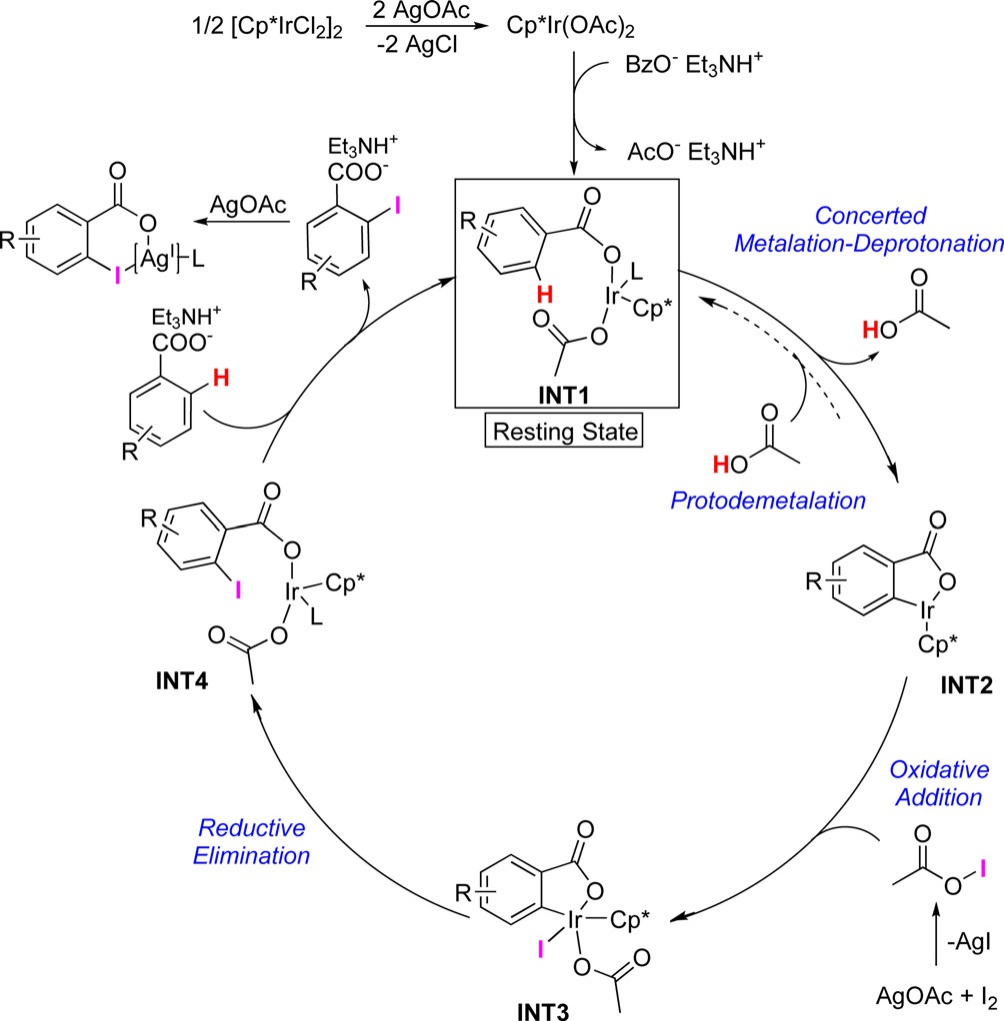
Proposed reaction mechanism.

For **1 a**, the CMD is reversible to a greater extent than for **1 l**, and the observed KIE of 2.19 indicates that this step is only partially rate limiting. Oxidative addition of AcOI to iridacycle **INT2** leads to the formation of **INT3**. Subsequent reductive elimination gives **INT4**, which, upon ligand exchange, reforms **INT1**. After the product is released, it is proposed to interact with Ag^I^, forming a coordination complex that inhibits activation of the *ortho′*‐C−H bond.

A catalytic method for the regioselective *ortho*‐monoiodination of benzoic acids with two equivalent *ortho*‐C−H bonds has been developed. Commercially available [Cp*IrCl_2_]_2_ acts as an efficient precatalyst for the transformation of a range of *para*‐, as well as *meta*‐substituted benzoic acids. Notably, when additional directing groups are present in the substrates, the carboxylic acid acts as the only directing group for the iodination reaction. With the high synthetic utility of the C‐I motif, the use of the products in cross‐coupling reaction is expected, including diversity‐oriented synthesis in context of drug discovery. The products of this reaction can potentially be used for generation of diverse cyclic hypervalent iodine reagents.^23^ The Ag^I^ additive is proposed to play a triple role in the reaction mechanism: precatalyst activation, generation of the active iodinating agent AcOI, as well as a crucial role in the high selectivity obtained. The observation of the substrate‐dependent turnover‐limiting step not only shows the complexity of the proposed catalytic cycle, but also stresses the need for testing electronically diverse substrates in KIE investigations in order to come to correct conclusions. Finally, the mechanistic insights will hopefully contribute to the development of future catalytic methods for selective C−H monofunctionalization.

## Conflict of interest

The authors declare no conflict of interest.

## Supporting information

As a service to our authors and readers, this journal provides supporting information supplied by the authors. Such materials are peer reviewed and may be re‐organized for online delivery, but are not copy‐edited or typeset. Technical support issues arising from supporting information (other than missing files) should be addressed to the authors.

Supplementary
