# Supplementary material for: IrIII‐Catalyzed Selective ortho‐Monoiodination of Benzoic Acids with Unbiased C−H Bonds
Source: Chemistry. 2020 Jul 27;26(45):10185–90. doi: 10.1002/chem.202002204 (PMC7496429; doi:10.1002/chem.202002204)
Supplement: Supplementary file 1 — Supplementary [file CHEM-26-10185-s001.pdf]

Chemistry–A European Journal

Supporting Information

Ir^{III}-Catalyzed Selective *ortho*-Monoiodination of Benzoic Acids with Unbiased C–H Bonds

Erik Weis,[a, b] Magnus J. Johansson,*[b] and Belén Martín-Matute*[a]

Table of Contents

S3 General Information

S4 Optimization studies

- S4 Initial screening
- S5 Counterion effect
- S6 Solvent screening
- S6 Silver source screening
- S7 $[Cp^*IrCl_2]_2$ as precatalyst
- S8 Dependence of AgOAc loading on conversion and selectivity
- S9 Base screening

S11 General procedure for the *ortho* monoiodination of benzoic acids (**1**)

S12 Synthesis and characterization data for *ortho*-iodobenzoic acids (**2**)

S24 Synthesis and characterization data for 4-(trifluoromethyl)benzoic-2,6-*d*2 acid (**1m-D2**)

S25 Synthesis and characterization data for $Cp^{*OCH(CF3)2}Ir(O_2CC_6H_4)$ (**Ic-2**)

S26 Mechanistic studies

- S26 Deuterium incorporation under standard conditions
- S27 Deuterium incorporation – substituent effect
  - S27 *ortho*-Substituent effect
  - S32 *para*-Substituent effect
- S34 Deuterium incorporation: site selectivity
- S41 Ag^I effect on C–H activation of **2a**
- S42 Iridacycle **Ic-3** as precatalyst
- S43 C–I bond cleavage experiments
- S43 KIE experiments
  - S43 E1: Benzoic acid (**1a**, **1a-D5**) as model system
  - S45 E2: 4-(trifluoromethyl) benzoic acid (**1l**, **1l-D2**) as model system
- S47 Hammett plot studies
- S49 Resting state studies
- S53 Iridacycle **Ic-2**
- S53 Ortho substituent steric effect
- S54 Comparison with NIS methodology
- S55 Reaction kinetic profile
  - S55 (**2a**) reaction kinetics
  - S56 (**2l**) reaction kinetics

S58 References

S59 ^{1}H, ^{13}C and ^{19}F NMR of iodobenzoic acids 2

S97 ^{1}H NMR, ^{13}C NMR and ^{19}F of 4-(trifluoromethyl) benzoic-2,6-d2 acid (**1l-D2**)

S99 ^{1}H NMR, ^{13}C NMR, ^{19}F and 2-D NMR spectra of $Cp^{*OCH(CF_3)_2}Ir(O_2CC_6H_4)$ (**Ic-2**)

General information

All reactions were performed under air atmosphere with temperature monitored at 23 °C unless otherwise stated. Solvents were used as obtained from vendors. Organic phases (after aqueous workup) were dried over anhydrous $MgSO_4$, filtered, and evaporated to dryness under reduced pressure. Silica plug purifications were performed on Biotage ISOLUTE SI 10g pre-loaded columns. Products were purified by preparative reverse-phase HPLC on a Kromasil C8 column (10 µm, 250x50 ID mm), using gradient elution (A: H_2O/MeCN/AcOH 95/5/0.2, B: MeCN) with a flow rate of 100 mL/min over 20 minutes. Gilson GX-281 liquid handler/autosampler was used. UV detector Gilson UV/VIS-155 was used for UV-triggered collection of fractions at 254 nm wavelength. LCMS analysis was carried out on a Waters Acquity UPLC system on an HSS C18 column. For SFC-MS analysis a Waters Acquity UPC^2 SFC-MS system with a BEH column was used. Nuclear magnetic resonance spectra (^{1}H, ^{13}C, ^{19}F, COSY, HSQC, HMBC, NOSEY) were recorded on Bruker ULTRASHIELD 500 MHz spectrometer with a Bruker CRYO PLATFORM. ^{1}H NMR spectra were referenced to $CDCl_3$ (7.26 ppm), CD_3OD (3.31 ppm) and C_6D_6 (7.16 ppm). ^{13}C NMR spectra were recorded at 126 MHz, referenced to in $CDCl_3$ (77.16 ppm), CD_3OD (49.00 ppm) and C_6D_6 (128.06 ppm). Coupling constant (J) values were measured in Hertz (Hz) and chemical shift (δ) values in parts per million (ppm). High resolution mass spectra (HRMS) were recorded on a Bruker microTOF ESI-TOF mass spectrometer.

Reagents: All the reagents used were commercially available and used without further purification. I_2 was purchased from Alfa Aesar and was ground to a fine powder with a pestle and mortar prior to use. AgOAc and $[Cp^*IrCl_2]_2$ were purchased from STREM, HFIP from Chem-Impex International, Et_3N from Aldrich. Benzoic acids were purchased from Combi-Blocks, Aldrich, Enamine, Fluorochem, Lancaster, Acros, Ubichem, WuXi AppTec and Fluka.

Optimization Studies

Initial Screening

General conditions 1: Potassium benzoate (**1a**$_{K\text{-salt}}$, 40.1 mg, 0.25 mmol) was dissolved in 1,1,1,3,3,3-hexafluoro-2-propanol (HFIP, 2.5 mL, 0.1 M). In a separate screw capped vial $[Cp^*Ir(H_2O)_3]SO_4$ (3.5 mg, 7.5 µmol, 3 mol%), AgOAc, I_2 and additive were added. The substrate (**1a**$_{K\text{-salt}}$) solution was added to this mixture with a pasteur pipette, the vial closed and covered with aluminum foil, and the mixture stirred vigorously at room temperature (23 °C) under air atmosphere. After the given reaction time, 0.5 mL of the reaction mixture was added to a mixture of saturated aqueous $Na_2S_2O_3$ solution (0.5 mL), aqueous HCl (1.5 mL, 1 M) and CH_2Cl_2 (2 mL). The organic phase was separated, and the aqueous phase extracted with CH_2Cl_2 (2 x 1.5 mL). The combined organic phases were concentrated, and the residue dissolved in CD_3OD (0.5 mL). Screening details and entries shown in Table S1.

Table S1. Initial screening

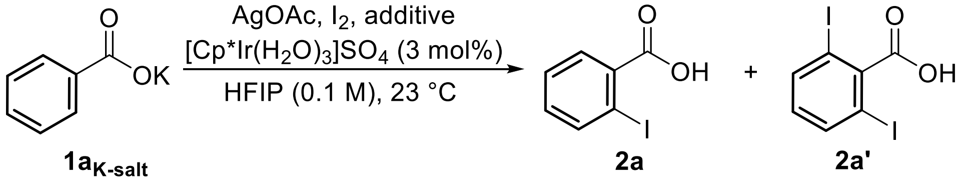

Entry	AgOAc (equiv.)	I_2 (equiv.)	Additive (equiv.)	Time	**2a**:**2a'**	Conversion (%)
1[a]	0	NIS (1.5)		16 h	8:1	45
2	0	NIS (1.5)		16 h	>20:1	25
3	1.5	NIS (1.5)		16 h	>20:1	11
4[a]	1.5	NIS (1.5)		16 h	>20:1	13
5	1.3	1.2		16 h	13:1	91
6	1.3	1.3		18 h	10:1	85
7	2.2	1.2		16 h	>20:1	89
8	2.2	2.2		16 h	10:1	95
9	0	2.2		18 h		0
10[b]	2.2	2.2		18 h		0
11	1.3	1.3	KOAc (1.0)	18 h	10:1	94
12[c]	1.3	1.3		18 h	10:1	78
13	1.3	1.3		10 min	>20:1	38
14	1.3	1.3		1 h	13:1	82
15[d]	1.2	1		16 h	8:1	82
16[e]	1.2	1		2 h	>20:1	62
17[e]	1.2	1		1 week	>20:1	81
18	1.6	1.3		19 h	9:1	94
19	1.5	1.3	Bu_4NOAc (1.3)	16 h	6:1	94
20	1.6	1.3		19 h	9:1	94
21	1.6	1.3		19 h	9:1	94
22	1.3	1.3	LiOAc (1.0)	19 h	10:1	94
23	1.3	1.3	K_2CO_3 (1.0)	19 h	16:1	95
24	1.3	1.3	K_2CO_3 (1.0), LiOAc (1.0)	19 h	>20:1	55
25	1.3	1.3	K_2CO_3 (0.5), KOAc (1.0)	19 h	17:1	95
26[f]	1.4	1.3	K_2CO_3 (1.2)	21 h	>20:1	7

27[g]	1.4	1.3	K_2CO_3 (1.2)	21 h	>20:1	18
28[a]	1.3	1.3	K_2CO_3 (3.0)	21 h	>20:1	51
29	1.3	1.1	K_2CO_3 (1.2)	21 h	17:1	92
30	1.3	1.1	K_2CO_3 (1.2)	18 h	18:1	72
31	1.3	1.1	K_2CO_3 (1.2)	10 min	>20:1	23
32	1.3	1.1	K_2CO_3 (1.2)	1 h	14:1	77
33	1.5	1.3	K_2CO_3 (1.2)	10 min	>20:1	38
34	1.5	1.3	K_2CO_3 (1.2)	1 h	18:1	86

[a] BzOH used as starting material. [b] No catalyst. [c] HFIP/CH_2Cl_2 4:1. [d] Portion wise AgOAc (2 equal portions, 1-hour time difference). [e] Portion wise I_2 (2 equal portions, 1h time difference). [f] AgOAc and I_2 prestirred in HFIP for 24 h, then filtered and the solution added to BzOK and catalyst. [g] AgOAc and I_2 prestirred in TFE (0.5 ml) for 12 h, then filtered and the solution added to BzOK and catalyst. in HFIP (2 ml).

Counterion Effect

General conditions 2: The benzoate salts were prepared from benzoic acid (**1a**, 30.5 mg, 0.25 mmol) and the corresponding carbonate salt M_XCO_3 (0.125 mmol). The mixture was added to a screw capped vial and suspended in MeOH (2.5 mL). The volatiles were removed under reduced pressure at 40 °C. The residue was coevaporated with toluene (2x1 mL) and dried overnight. The resulting solid was directly used for the screening. 1,1,1,3,3,3-hexafluoro-2-propanol (HFIP, 2.5 mL, 0.1 M) was added to the vial containing the substrate. To a separate screw capped vial $[Cp^*Ir(H_2O)_3]SO_4$ (3.5 mg, 7.5 µmol, 3 mol%), AgOAc and I_2 were added. The solution/suspension of the benzoate salt was added to this mixture, the vial closed and covered with aluminum foil, and the mixture stirred vigorously at room temperature (23 °C) under air atmosphere. After the given reaction time, 0.5 mL of the reaction mixture was added to a mixture of saturated aqueous $Na_2S_2O_3$ solution (0.5 mL), aqueous HCl (1.5 mL, 1 M) and CH_2Cl_2 (2 mL). The organic phase was separated, and the aqueous phase extracted with CH_2Cl_2 (2 x 1.5 mL). The organic phase was separated, and the aqueous phase extracted with CH_2Cl_2 (2x1 mL). The combined organic phases were concentrated, and the residue dissolved in CD_3OD (0.5 mL). The product distribution and conversion were determined by ^{1}H NMR. Screening details and entries shown in Table S2.

Table S2. Counterion effect

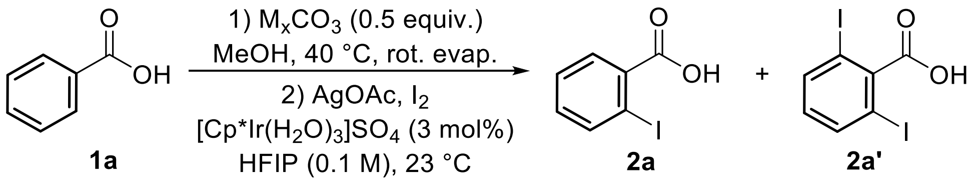

Entry	M_xCO_3	Benzoate salt	AgOAc (equiv.)	I_2 (equiv.)	Time	**2a**:**2a'**	Conversion
1	Na_2CO_3	BzONa	1.3	1.1	90 min	9:1	65
2	K_2CO_3	BzOK	1.3	1.1	90 min	12:1	82
3	Cs_2CO_3	BzOCs	1.3	1.1	90 min	11:1	84
4	$CaCO_3$	BzO_2Ca	1.3	1.1	90 min	14:1	65

Solvent Screening

Screening conducted according to General conditions 1, alternative solvents used. Screening details and entries shown in Table S3.

Table S3. Solvent screen

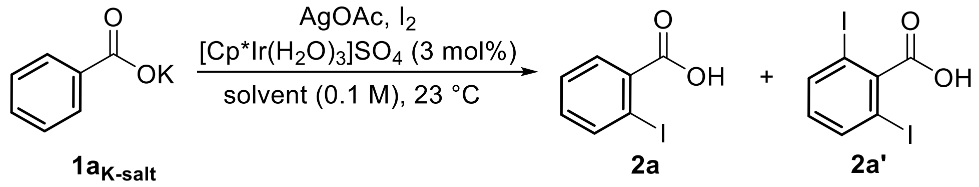

Entry	Solvent	AgOAc (equiv.)	I_2 (equiv.)	Time	**2a**:**2a’**	Conversion
1	CH_2Cl_2	1.3	1.1	16 h		0
2	$CHCl_3$	1.3	1.1	18 h		0
3	THF	1.3	1.1	18 h		0
4	AcOH	1.3	1.1	18 h		0
5	TFE	1.3	1.1	18 h	2:1	51
6	HFIP	1.3	1.1	16 h	13:1	89

Silver source screening

Screening conducted according to General conditions 1, alternative silver sources used. Screening details and entries shown in Table S4.

Table S4. Silver source screening

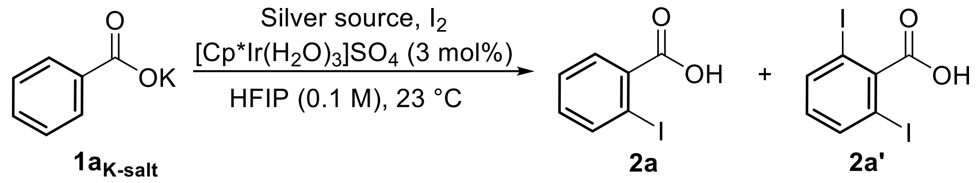

Entry	Silver source (equiv.)	I_2 (equiv.)	Time	**2a**:**2a’**	Conversion
1	Ag_2CO_3 (1.2)	1.2	15 h	>20:1	85
2[a]	$AgSbF_6$ (2.3)	1.2	15 h		71
3[b]	$AgNTf_2$ (2.3)	1.2	15 h		74
4	AgOAc (2.3)	1.2	15 h	>20:1	91
5	Ag_2O (1.2)	1.2	15 h	>20:1	85

[a] Loss of regioselectivity observed. *ortho*:*meta* 1:2. [b] Loss of regioselectivity observed. *ortho:meta* 2:1.

[Cp*IrCl$_2$]$_2$ as precatalyst

Screening conducted according to General conditions 1, [Cp*IrCl$_2$]$_2$ (3.0 mg, 3.75 µmol, 1.5 mol%) used as catalyst. Screening details and entries shown in Table S5.

Table S5. Reoptimization with [Cp*IrCl$_2$]$_2$

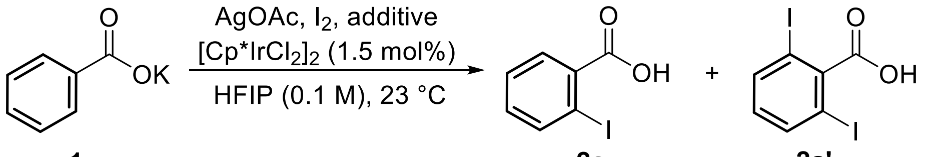

Entry	AgOAc (equiv.)	I$_2$ (equiv.)	Additive (equiv.)	Time	**2a**:**2a'**	Conversion
1	1.3	1.2		18 h	13:1	95
2	1.3	1.2	KOAc (1.0)	21 h	9:1	95
3[a]	1.3	1.1		18 h	>20:1	89
3[b]	1.3	1.1		18 h	>20:1	78
4[c]	1.3	1.1		18 h	>20:1	43
5	2.2	1.1		18 h	>20:1	92
6[d]	1.2	1.05		18 h	14:1	58
7[e]	1.2	1.05		18 h	20:1	66
8[f]	1.2	1.05		18 h		0
9[g]	1.2	1.05		18 h	11:1	83
10	1.26	1.2		18 h	10:1	92
11	1.46	1.2		18 h	9:1	95
12	1.66	1.2		18 h	>20:1	82
13	1.86	1.2		18 h	>20:1	81
14	2.06	1.2		18 h	>20:1	91
15	2.26	1.2		18 h	>20:1	92
16	2.46	1.2		18 h	>20:1	93
17	1.2	5.0		16 h	9:1	90
18	2.3	1.2		18 h	>20:1	94
19	2.3	1.2	K_2CO_3 (0.5)	18 h	>20:1	93
20	2.4	1.3		18 h	>20:1	93
21	2.7	1.2		18 h	>20:1	94
22	2.5	1.5		18 h	>20:1	90
23	3.0	1.5		18 h	>20:1	91
24	2.5	2.0		18 h	10:1	>95
25	3.0	2.0		18 h	>20:1	>95
26	3.5	2.0		18 h	>20:1	>95
27	3.7	2.2		18 h	>20:1	100
28	3.7	1.1		18 h	>20:1	95
29	5.0	3.0		21 h	9:1	100
30[h]	3.7	2.2		18 h	>20:1	100
31[i]	3.7	2.2		18 h	>20:1	100
32	3.7	2.2	AcOH (1.0)	18 h	>20:1	>95
33[j]	3.7	2.2		18 h	3:1	100
34[k]	3.7	2.2		18 h	>20:1	56

35	0.56	1.2	18 h	>20:1	41
36	1.06	1.2	18 h	>20:1	64

[a] Catalyst 1.5 mol%. [b] Catalyst 1 mol%. [c] Catalyst 0.75 mol%. [d] AgOAc and I_2 10 min prestir in CH_2Cl_2 (0.2 mL), [e] AgOAc and I_2 10 min prestir in $CHCl_3$ (0.2 mL), [f] AgOAc and I_2 10 min prestir in THF (0.2 mL), [g] AgOAc and I_2 10 min prestir in TFE (0.2 mL), [h] Temperature 40 °C. [i] Temperature 60 °C. [j] Catalyst 5 mol%. [k] Catalyst 0.5 mol%

Dependence of AgOAc loading on conversion and selectivity

Screening conducted according to General conditions 1, $[Cp^*IrCl_2]_2$ (3.0 mg, 3.75 µmol, 1.5 mol%) used as catalyst. Entries 10-16, 35 and 36 from Table S5 plotted (Figure S1).

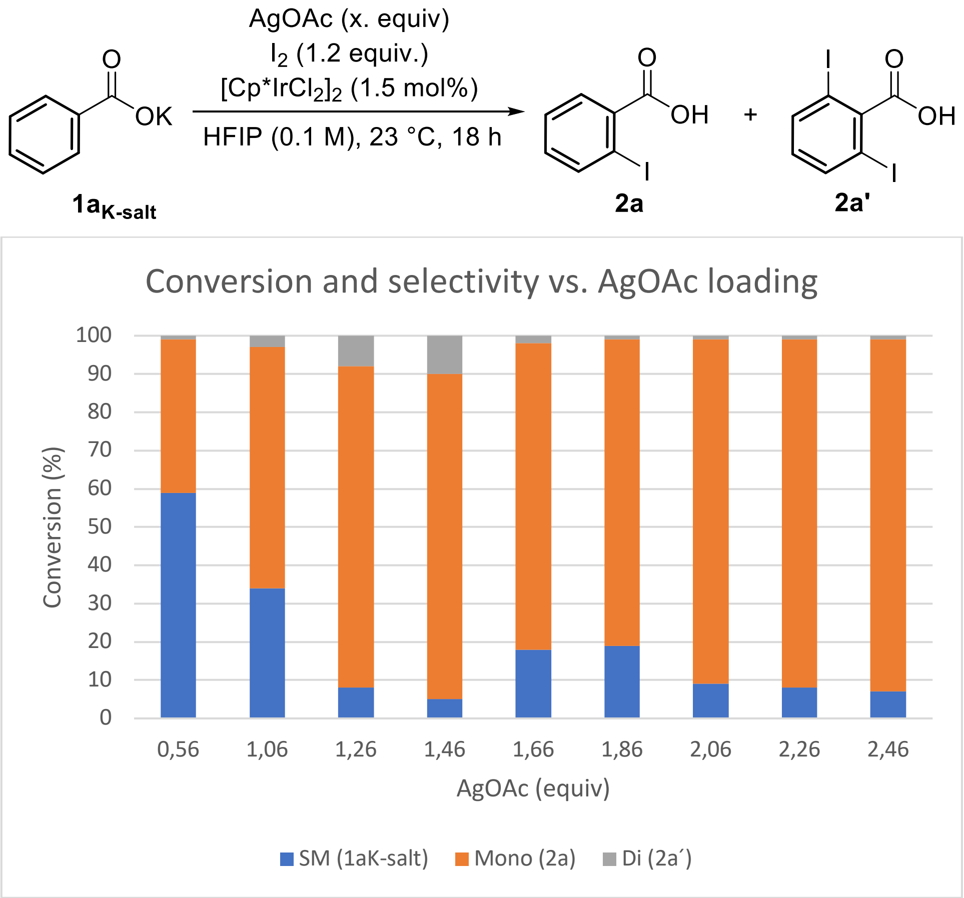

Figure S1. Dependence of conversion to **2a** and **2a’** on AgOAc loading. An overall increase in conversion was observed with AgOAc loading from 0.56 to 1.46 equiv, albeit with decreasing selectivity for **2a**. Selectivity was restored with 1.66 equiv AgOAc, albeit with lower conversion. Further increasing AgOAc loading led to improved conversion and high selectivity for **2a**.

Base screening

General conditions 4: Benzoic acid (**1a**, 30.5 mg, 0.25 mmol) was dissolved in 1,1,1,3,3,3-hexafluoro-2-propanol (HFIP, 2.5 mL, 0.1 M), followed by addition of base. In a separate screw capped vial $[Cp^*IrCl_2]_2$ (3.0 mg, 3.75 µmol, 1.5 mol%), AgOAc, I_2 and additive were added. The substrate (**1a**) solution was added to this mixture, the vial covered with aluminum foil, and the mixture stirred vigorously at room temperature (23 °C) under air atmosphere. After the given reaction time, 0.5 mL of the reaction mixture was added to a mixture of saturated aqueous $Na_2S_2O_3$ solution (0.5 mL), aqueous HCl (1.5 mL, 1 M) and CH_2Cl_2 (2 mL). The organic phase was separated, and the aqueous phase extracted with CH_2Cl_2 (2 x 1.5 mL). The combined organic phases were concentrated, and the residue dissolved in CD_3OD (0.5 mL). The product distribution and conversion were determined by ^{1}H NMR. Screening details and entries shown in Table S6.

Table S6. Base screen

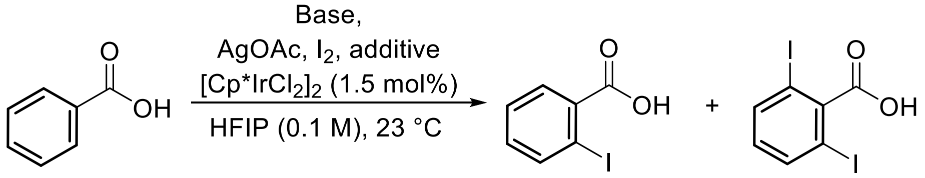

1a **2a** **2a'**

Entry	Base (equiv.)	Additive (equiv.)	AgOAc (equiv.)	I_2 (equiv.)	Time	**2a**:**2a'**	Conversion
1			3.7	2.2	18 h	>20:1	68
2[a]	Cs_2CO_3 (0.5)		3.7	2.2	18 h	>20:1	100
3[a]	K_2CO_3 (0.5)		3.7	2.2	18 h	>20:1	100
4	$KHCO_3$ (0.5)		3.7	2.2	18 h	>20:1	7
5	K_2CO_3 (1.0)	$MgSO_4$ (50.0 mg)	1.3	1.05	18 h	>20:1	86
6	K_2CO_3 (1.0)	MS 3Å (58.1 mg)	1.3	1.05	18 h	>20:1	91
7	K_2CO_3 (1.0)	$MgSO_4$ (48.7 mg)	1.3	1.05	18 h	>20:1	82
8	K_2CO_3 (1.0)		1.3	1.05	18 h	>20:1	83
9	DIPEA (2.3)		1.3	1.05	18 h	18:1	95
10	Et_3N (2.3)		1.3	1.05	18 h	>20:1	93
11	Pyridine (2.3)		1.3	1.05	18 h		0
12	DABCO (2.3)		1.3	1.05	18 h	>20:1	24
13	Et_3N (3.3)		1.4	1.1	18 h	>20:1	91
14	Et_3N (3.3)		1.3	1.1	18 h	>20:1	92
15	Et_3N (3.0)		1.3	1.1	18 h	>20:1	90
16	Et_3N (1.0)		1.3	1.1	18 h	>20:1	80
17	Et_3N (2.0)		1.3	1.1	18 h	>20:1	87
18[b]	Et_3N (3.3)		1.3	1.1	18 h	>20:1	94
19[c]	Et_3N (3.3)		1.3	1.1	18 h	>20:1	92
20[d]	Et_3N (3.3)		1.3	1.1	18 h	>20:1	93
21[e]	Et_3N (3.3)		1.3	1.1	18 h	>20:1	81

22	Et_3N (5.0)		1.3	1.1	18 h	>20:1	80
23	Et_3N (3.3)		1.5	1.2	18 h	>20:1	95
24	Et_3N (3.3)		1.6	1.3	18 h	16:1	>95
25	K_2CO_3 (0.5)	MS 3Å (60.1 mg)	3.7	2.2	18 h	>20:1	100
26[f]	Et_3N (1.0)		3.7	2.2	18 h		0
**27**	**Et_3N (1.0)**		**3.7**	**2.2**	**18 h**	**>20:1**	**100**

[a] According to General conditions 2. BzOK used as starting material. [b] RM concentration 0.05 M. [c] RM concentration 0.1 M. [d] RM concentration 0.15 M. [e] RM concentration 0.2 M. [f] No $[Cp^*IrCl_2]_2$. Highlighted in bold are the optimized conditions chosen for the scope investigation.

General procedure for the *ortho* monoiodination of benzoic acids (1)

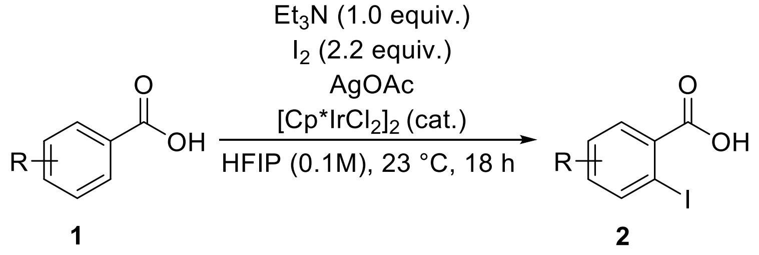

Note: I_2 was ground to a fine powder prior to use.

Method A
Substrate (**1**) (0.5 mmol) was weighed in a vial and suspended in 1,1,1,3,3,3-hexafluoro-2-propanol (HFIP, 5 mL, 0.1 M). Et_3N (69.7 µL, 0.5 mmol) was added and the mixture sonicated for 30 seconds. To a separate vial $[Cp^*IrCl_2]_2$ (6.0 mg, 7.5 µmol, 1.5 mol%) and AgOAc (309 mg, 1.85 mmol) were added. The substrate (**1**) solution was added to this mixture, followed by the addition of ground I_2 (279 mg, 1.1 mmol), and the vial was covered with aluminum foil. The mixture was stirred vigorously at 23 °C for 18 hours, upon which Na_2SO_3 (600 mg) and water (3 mL) were added. The mixture was sonicated for 30 seconds and added to aqueous HCl (25 mL, 1.0 M). The aqueous phase was extracted with EtOAc (5 x 30 mL). The combined organic fractions were washed with brine (20 mL), dried over $MgSO_4$ and concentrated under reduced pressure.

Method B
Procedure as Method A, $[Cp^*IrCl_2]_2$ (10.0 mg, 12.5 µmol, 2.5 mol%), AgOAc (317 mg, 1.9 mmol) used.

Method C
Procedure as Method A, $[Cp^*IrCl_2]_2$ (12.0 mg, 15.0 µmol, 3.0 mol%), AgOAc (317 mg, 1.9 mmol) used.

Synthesis and characterization data for *ortho*-iodobenzoic acids (2)

2-iodobenzoic acid (2a)[1]

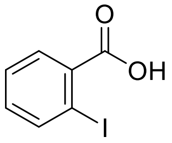

Method A. The crude material was dissolved in CH_2Cl_2, applied on a silica plug (10 g) and the product was eluted with EtOAc/heptane/AcOH (49/49/2, 70 mL). The volatiles were removed *in vacuo* to provide 2-iodobenzoic acid (**2a**) as an off-white solid (119.0 mg, 96 %). Isolated as a mixture with 2,6-diiodobenzoic (**2a'**), >20:1 **2a**: **2a'** ratio.

^{1}H NMR (500 MHz, CD_3OD): δ 8.01 (d, *J* = 8.0 Hz, 1H), 7.79 (dd, *J* = 7.8, 1.7 Hz, 1H), 7.45 (td, *J* = 7.7, 0.8 Hz, 1H), 7.19 (td, *J* = 7.7, 1.6 Hz, 1H)

^{13}C NMR (126 MHz, CD_3OD): δ 170.1, 142.3, 137.8, 133.5, 131.6, 129.1, 94.2

HRMS (ESI) m/z calcd. for $C_7H_5IO_2$ $[M-H]^-$: 246.9256, found 246.9261

2-iodo-4-methylbenzoic acid (2b)[2]

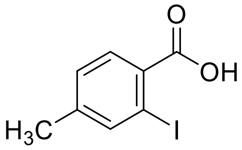

Method A. Reaction time 2 hours. The crude material was dissolved in DMSO and the product purified by preparative HPLC, gradient 32-72 % MeCN in acidic buffer, to provide 2-iodo-4-methylbenzoic acid (**2b**) as an off-white solid (96.9 mg, 74 %).

^{1}H NMR (500 MHz, $CDCl_3$): δ 7.95 (d, *J* = 8.0 Hz, 1H), 7.92-7.90 (m, 1H), 7.26-7.23 (m, 1H), 2.36 (s, 3H)

^{13}C NMR (126 MHz, $CDCl_3$): δ 170.9, 144.9, 142.8, 132.3, 130.0, 129.0, 95.2, 21.1

HRMS (ESI) m/z calcd. for $C_8H_7IO_2$ $[M+H]^+$: 262.9569, found 262.9571

4-cyclohexyl-2-iodobenzoic acid (2c)

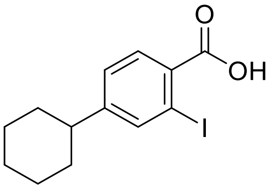

Method A. Reaction time 2 hours. The crude material was dissolved in DMSO and the product purified by preparative HPLC, gradient 43-83 %, to provide 4-cyclohexyl-2-iodobenzoic acid (**2c**) as an off-white solid (120.5 mg, 73 %).

^{1}H NMR (500 MHz, CD_3OD): δ 7.85 (s, 1H), 7.75 (d, *J* = 7.5 Hz, 1H), 7.30 (d, *J* = 7.5 Hz, 1H), 2.56-2.47 (m, 1H), 1.91-1.79 (m, 4H), 1.79-1.72 (m, 1H), 1.49-1.37 (m, 4H), 1.34-1.25 (m, 1H)

^{13}C NMR (126 MHz, CD_3OD): δ 169.8, 154.5, 141.0, 134.6, 132.0, 127.6, 94.8, 45.3, 35.2, 27.7, 27.0

HRMS (ESI) m/z calcd. for $C_{13}H_{15}IO_2$ $[M+H]^+$: 331.0195, found 331.0194

2-iodo-4-methoxybenzoic acid (2d)[3]

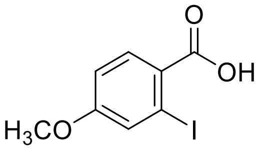

Method A. Reaction time 2 hours. The crude material was dissolved in DMSO and the product purified by preparative HPLC, gradient 23-63 %, to provide 2-iodo-4-methoxybenzoic acid (**2d**) as an off-white solid (83.4 mg, 60 %).
^{1}H NMR (500 MHz, CD_3OD): δ 7.88 (d, *J* = 8.8 Hz, 1H), 7.56 (d, *J* = 2.5 Hz, 1H), 7.00 (dd, *J* = 8.8, 2.5 Hz, 1H), 3.84 (s, 3H)

^{13}C NMR (126 MHz, CD_3OD) δ 169.1, 163.5, 133.6, 128.3, 128.2, 114.3, 95.9, 56.2

HRMS (ESI) m/z calcd. for $C_8H_7IO_3$ $[M+H]^+$: 278.9518, found 278.9523

2-iodo-4-(sulfamoylmethyl) benzoic acid (2e)

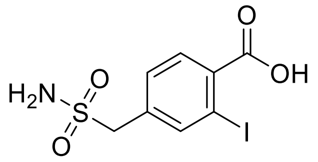

Method B. The crude material was dissolved in DMSO and the product purified by preparative HPLC, gradient 0-30 %, to provide 2-iodo-4-(sulfamoylmethyl) benzoic acid (**2e**) as a colorless solid (112.5 mg, 66 %).
^{1}H NMR (500 MHz, CD_3OD): δ 8.08 (d, *J* = 1.4 Hz, 1H), δ 7.80 (d, *J* = 8.0 Hz, 1H), δ 7.52 (dd, *J* = 8.0, 1.4 Hz, 1H), δ 4.34 (s, 2H)

^{13}C NMR (126 MHz, CD_3OD) δ 169.8, 144.4, 137.5, 136.6, 131.6, 131.4, 94.1, 60.3

HRMS (ESI) m/z calcd. for $C_8H_8INO_4S$ $[M-H]^-$: 339.9140, found 339.9142

4-(2-hydroxyethyl)-2-iodobenzoic acid (2f)

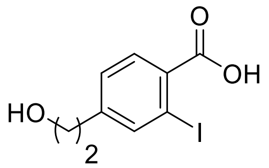

Method A. The crude material was dissolved in DMSO and the product purified by preparative HPLC, gradient 0-37 %, to provide 4-(2-hydroxyethyl)-2-iodobenzoic acid (**2f**) as a colorless solid (86.1 mg, 59 %).

^{1}H NMR (500 MHz, CD_3OD): δ 7.93 (d, *J* = 1.6 Hz, 1H), 7.76 (d, *J* = 8.0 Hz, 1H), 7.33 (dd, *J* = 8.0, 1.6 Hz, 1H), 3.77 (t, *J* = 6.7 Hz, 2H), 2.81 (t, *J* = 6.7 Hz, 2H)

^{13}C NMR (126 MHz, CD_3OD): δ 169.9, 146.2, 143.0, 135.1, 131.7, 129.8, 94.6, 63.3, 39.3

HRMS (ESI) m/z calcd. for $C_9H_9IO_3$ $[M+H]^+$: 292.9675, found 292.9675

4-acetoxy-2-iodobenzoic acid (2g)

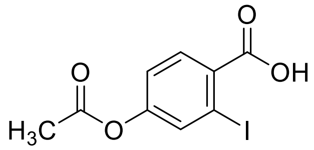

Method B. Saturated aqueous citric acid solution used instead of aqueous HCl in work-up. The crude material was dissolved in DMSO and the product purified by preparative HPLC, gradient 25-65 %, to provide 4-acetoxy-2-iodobenzoic acid (**2g**) as a colorless solid (99.4 mg, 65 %).

^{1}H NMR (500 MHz, CD_3OD): δ 7.87 (d, *J* = 8.5 Hz, 1H), 7.80 (d, *J* = 2.2 Hz, 1H), 7.24 (dd, *J* = 8.5, 2.2 Hz, 1H), 2.29 (s, 3H)

^{13}C NMR (126 MHz, CD_3OD): δ 170.3, 169.1, 154.0, 135.6, 134.6, 132.6, 122.6, 94.3, 20.8

HRMS (ESI) m/z calcd. for $C_9H_7IO_4$ $[M+H]^+$: 306.9467, found 306.9466

4-fluoro-2-iodobenzoic acid (2h)[4]

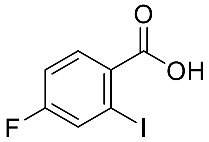

Method A. The crude material was dissolved in DMSO and the product purified by preparative HPLC, gradient 30-70 %, to provide 4-fluoro-2-iodobenzoic acid (**2h**) as a colorless solid (109.0 mg, 82 %).

^{1}H NMR (500 MHz, CD_3OD): δ 7.90 (dd, *J* = 8.7, 6.0 Hz, 1H), 7.80 (dd, *J* = 8.5, 2.5 Hz, 1H), 7.23 (td, *J* = 8.3, 2.5 Hz, 1H)

^{13}C NMR (126 MHz, CD_3OD): δ 168.8, 164.7 (d, J_{C-F} = 255.3 Hz, 1C), 133.7 (d, J_{C-F} = 9.1 Hz, 1C), 133.6 (d, J_{C-F} = 3.0 Hz, 1C), 129.4 (d, J_{C-F} = 23.9 Hz, 1C), 116.1 (d, J_{C-F} = 21.9 Hz, 1C), 95.1 (d, J_{C-F} = 8.4 Hz, 1C)

^{19}F NMR (470 MHz, CD_3OD): δ -109.81 (s)

HRMS (ESI) m/z calcd. for $C_7H_4FIO_2$ $[M-H]^-$: 264.9162, found 264.9160

4-chloro-2-iodobenzoic acid (2i)[5]

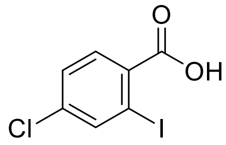

Method A. The crude material was dissolved in DMSO and the product purified by preparative HPLC, gradient 30-70 %, to provide 4-chloro-2-iodobenzoic acid (**2i**) as an off-white solid (78.9 mg, 56 %).

^{1}H NMR (500 MHz, CD_3OD): δ 8.05 (d, *J* = 2.1 Hz, 1H), 7.80 (d, *J* = 8.5 Hz, 1H), 7.48 (dd, *J* = 8.5, 2.1 Hz, 1H)

^{13}C NMR (126 MHz, CD_3OD): δ 168.9, 141.7, 138.6, 136.1, 132.7, 129.3, 94.9

HRMS (ESI) m/z calcd. for $C_7H_4ClIO_2$ $[M-H]^-$: 280.8866, found 280.8868

4-bromo-2-iodobenzoic acid (2j)

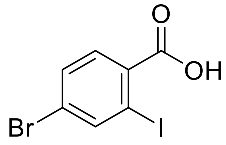

Method A. The crude material was dissolved in DMSO and the product purified by preparative HPLC, gradient 25-65 %, to provide 4-bromo-2-iodobenzoic acid (**2j**) as an off-white solid (107.5 mg, 66 %).

^{1}H NMR (500 MHz, CD_3OD): δ 8.20 (d, *J* = 2.0 Hz, 1H), 7.72 (d, *J* = 8.3 Hz, 1H), 7.63 (dd, *J* = 8.3, 2.0 Hz, 1H)

^{13}C NMR (126 MHz, CD_3OD): δ 169.1, 144.3, 136.7, 132.8, 132.3, 126.8, 95.2

HRMS (ESI) m/z calcd. for $C_7H_4BrIO_2$ $[M-H]^-$: 324.8361, found 324.8362

2-iodo-4-(trifluoromethoxy) benzoic acid (2k)

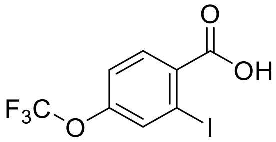

Method A. The crude material was dissolved in DMSO and the product purified by preparative HPLC, gradient 25-65 %, to provide 2-iodo-4-(trifluoromethoxy) benzoic acid (**2k**) as an off-white solid (129.4 mg, 78 %).

^{1}H NMR (500 MHz, $CDCl_3$): δ 8.09 (d, *J* = 8.8 Hz, 1H), 7.92–7.90 (m, 1H), 7.33–7.30 (m, 1H)

^{13}C NMR (126 MHz, $CDCl_3$): δ 169.4, 151.7 (q, $J_{C\text{-}F}$ = 1.8 Hz, 1C), 133.8, 133.5, 131.2, 120.3 (q, $J_{C\text{-}F}$ = 260.3 Hz, 1C), 119.8 (d, $J_{C\text{-}F}$ = 0.9 Hz, 1C), 95.5

^{19}F NMR (470 MHz, $CDCl_3$): δ -57.61 (s)

HRMS (ESI) m/z calcd. for $C_8H_4F_3IO_3$ $[M-H]^-$: 330.9079, found 330.9080

2-iodo-4-(trifluoromethyl) benzoic acid (2l)[6]

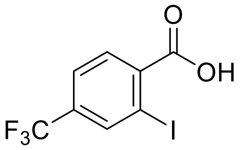

Method A. The crude material was dissolved in DMSO and the product purified by preparative HPLC, gradient 32-72 %, to provide 2-iodo-4-(trifluoromethyl) benzoic acid (**2l**) as a colorless solid (123.2 mg, 78 %).
^{1}H NMR (500 MHz, CD_3OD): δ 8.25 (d, J = 1.0 Hz, 1H), δ 7.91 (d, J = 8.0 Hz, 1H), δ 7.77 (dd, J = 8.0, 1.0 Hz, 1H)

^{13}C NMR (126 MHz, CD_3OD): δ 169.2, 142.3, 138.7 (q, $J_{C\text{-}F}$ = 3.9 Hz,), 134.4 (q, $J_{C\text{-}F}$ = 33.3 Hz,), 131.7, 126.0 (q, $J_{C\text{-}F}$ = 3.8 Hz,), 124.0 (q, $J_{C\text{-}F}$ = 270.4 Hz,), 94.0

^{19}F NMR (470 MHz, CD_3OD): δ -64.68 (s)

HRMS (ESI) m/z calcd. for $C_8H_4F_3IO_2$ $[M-H]^-$: 314.9130, found 314.9125

2-iodo-4-nitrobenzoic acid (2m)[7]

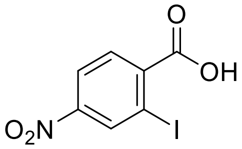

Method C. The crude material was dissolved in DMSO and the product purified by preparative HPLC, gradient 10-50 %, to provide 2-iodo-4-nitrobenzoic acid (**2m**) as a yellow solid (65.9 mg, 45 %).

^{1}H NMR (500 MHz, CD_3OD): δ 8.76 (s, 1H), 8.29 (d, J = 8.5 Hz, 1H), 7.91 (d, J = 8.5 Hz, 1H)

^{13}C NMR (126 MHz, CD_3OD): δ 168.8, 150.0, 144.1, 136.5, 131.8, 123.9, 93.6

HRMS (ESI) m/z calcd. for $C_7H_4INO_4$ $[M-H]^-$: 291.9107, found 291.9106

2-iodoterephthalic acid (2n)[8]

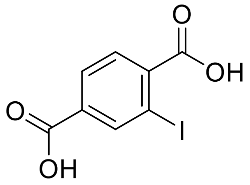

Method C. The crude material was dissolved in DMSO and the product purified by preparative HPLC, gradient 0-30%, to provide 2-iodoterephthalic acid (**2n**) as a colorless solid (75.9 mg, 52 %).

^{1}H NMR (500 MHz, CD_3OD): δ 8.57 (s, 1H), 8.06 (d, *J* = 8.0 Hz, 1H), 7.82 (d, *J* = 8.0 Hz, 1H)

^{13}C NMR (126 MHz, CD_3OD): δ 169.7, 167.2, 143.0, 142.1, 135.2, 131.1, 130.1, 93.5

HRMS (ESI) m/z calcd. for $C_8H_5IO_4$ $[M-H]^-$: 290.9154, found 290.9151

4-(carboxymethyl)-2-iodobenzoic acid (2o)

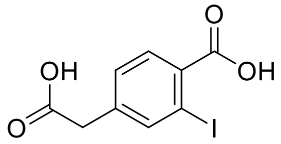

Method A. Higher base loading (Et_3N, 139.4 µL, 1.0 mmol) was used. The crude material was dissolved in DMSO and the product purified by preparative HPLC, gradient 0-30 %, to provide 4-(carboxymethyl)-2-iodobenzoic acid (**2o**) as a colorless solid (117.8 mg, 77 %).

^{1}H NMR (500 MHz, CD_3OD): δ 7.97 (d, *J* = 1.4 Hz, 1H), 7.77 (d, *J* = 8.0 Hz, 1H), 7.38 (dd, *J* = 8.0, 1.4 Hz, 1H), 3.63 (s, 2H)

^{13}C NMR (126 MHz, CD_3OD): δ 174.3, 169.8, 143.3, 141.2, 136.0, 131.6, 130.2, 94.3, 40.8

HRMS (ESI) m/z calcd. for $C_9H_7IO_4$ $[M-H]^-$: 304.9311, found 304.9312

4-acetamido-2-iodobenzoic acid (2p)

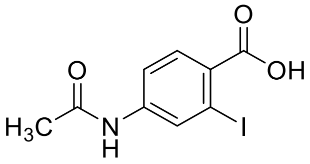

Method B. The crude material was dissolved in DMSO and the product purified by preparative HPLC, gradient 23-63 %, to provide 4-acetamido-2-iodobenzoic acid (**2p**) as an off-white solid (118.9 mg, 78 %).

^{1}H NMR (500 MHz, CD_3OD): δ 8.33 (d, *J* = 2.1 Hz, 1H), 7.83 (d, *J* = 8.5 Hz, 1H), 7.64 (dd, *J* = 8.5, 2.1 Hz, 1H), 2.14 (s, 3H)

^{13}C NMR (126 MHz, CD_3OD): δ 171.9, 169.1, 143.5, 132.8, 132.6, 131.3, 119.4, 95.1, 24.0

HRMS (ESI) m/z calcd. for $C_9H_8INO_3$ $[M+H]^+$: 305.9627, found 305.9623

2-iodo-4-(methoxycarbonyl) benzoic acid (2q)[9]

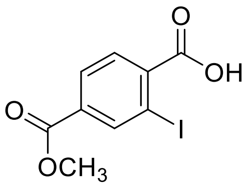

Method A. The crude material was dissolved in DMSO and the product purified by preparative HPLC, gradient 22-62 %, to provide 2-iodo-4-(methoxycarbonyl) benzoic acid (**2q**) as a colorless solid (110.2 mg, 72 %).

^{1}H NMR (500 MHz, CD_3OD): δ 8.56 (d, *J* = 1.5 Hz, 1H), 8.05 (dd, *J* = 8.0, 1.5 Hz, 1H), 7.82 (d, *J* = 8.0 Hz, 1H), 3.93 (s, 3H)

^{13}C NMR (126 MHz, CD_3OD): δ 169.6, 166.2, 142.7, 142.4, 134.4, 131.2, 129.9, 93.6, 53.1

HRMS (ESI) m/z calcd. for $C_9H_7IO_4$ $[M-H]^-$: 304.9311, found 304.9313

4-acetyl-2-iodobenzoic acid (2r)

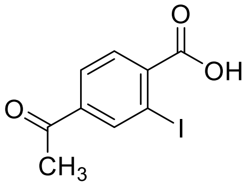

Method B. The crude material was dissolved in DMSO and the product purified by preparative HPLC, gradient 17-57 %, to provide 4-acetyl-2-iodobenzoic acid (**2r**) as a colorless solid (113.1 mg, 78 %).

^{1}H NMR (500 MHz, CD_3OD): δ 8.50 (d, *J* = 1.7 Hz, 1H), 8.03 (dd, *J* = 8.0, 1.7 Hz, 1H), 7.83 (d, *J* = 8.0 Hz, 1H), δ 2.61 (s, 3H)
^{13}C NMR (126 MHz, CD_3OD): δ 198.1, 169.6, 142.2, 141.6, 140.8, 131.4, 128.9, 94.0, 26.8

HRMS (ESI) m/z calcd. for $C_9H_7IO_3$ $[M-H]^-$: 288.9362, found 288.9361

4-carbamoyl-2-iodobenzoic acid (2s)

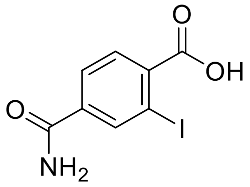

Method C. The crude material was dissolved in DMSO and the product purified by preparative HPLC, eluent (A: H_2O/MeCN/TFA 95/5/0.2, B: MeCN) used, gradient 0-20 %, to provide 4-carbamoyl-2-iodobenzoic acid (**2s**) as an off-white solid (45.0 mg, 31 %).

^{1}H NMR (500 MHz, CD_3OD): δ 8.47 (d, *J* = 1.6 Hz, 1H), 7.92 (dd, *J* = 8.0, 1.6 Hz, 1H), 7.82 (d, *J* = 8.0 Hz, 1H)

^{13}C NMR (126 MHz, CD_3OD): δ 169.68, 169.65, 141.3, 141.0, 138.4, 131.2, 128.1, 93.9

HRMS (ESI) m/z calcd. for $C_8H_6INO_3$ $[M+H]^+$: 291.9471, found 291.9469

2-iodo-4-(methylcarbamoyl) benzoic acid (2t)

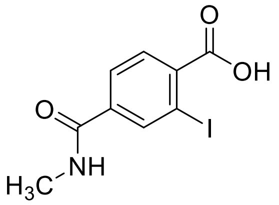

Method A. The crude material was dissolved in DMSO and the product purified by preparative HPLC, eluent (A: H_2O/MeCN/TFA 95/5/0.2, B: MeCN) used, gradient 0-20 %, to provide 2-iodo-4-(methylcarbamoyl) benzoic acid (**2t**) as a colorless solid (85.4 mg, 56 %).

^{1}H NMR (500 MHz, CD_3OD): δ 8.63 (br s, 1H), 8.41 (d, *J* = 1.6 Hz, 1H), 7.85 (dd, *J* = 8.0, 1.6 Hz, 1H), 7.81 (d, *J* = 8.0 Hz, 1H), 2.93-2.91 (m, 3H)

^{13}C NMR (126 MHz, CD_3OD): δ 169.6, 168.1, 140.9, 140.7, 138.8, 131.3, 127.7, 94.0, 27.0

HRMS (ESI) m/z calcd. for $C_9H_8INO_3$ $[M+H]^+$: 305.9627, found 305.9625

4-(dimethylcarbamoyl)-2-iodobenzoic acid (2u)

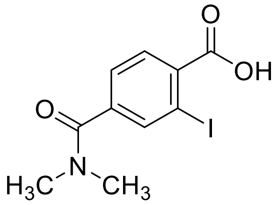

Method C. The crude material was dissolved in DMSO and the product purified by preparative HPLC, gradient 0-35 %, to provide 4-(dimethylcarbamoyl)-2-iodobenzoic acid (**2u**) as an off-white solid (105.3 mg, 66 %).

^{1}H NMR (500 MHz, CD_3OD): δ 8.04 (d, *J* = 1.5 Hz, 1H), 7.85 (d, *J* = 7.9 Hz, 1H), 7.50 (dd, *J* = 7.9, 1.5 Hz, 1H), 3.10 (s, 3H), 2.98 (s, 3H)

^{13}C NMR (126 MHz, CD_3OD): δ 171.0, 169.4, 141.1, 140.4, 139.1, 131.5, 127.5, 94.2, 39.8, 35.6

HRMS (ESI) m/z calcd. for $C_{10}H_{10}INO_3$ $[M+H]^+$: 319.9784, found 319.9783

4-(*N*,*N*-dipropylsulfamoyl)-2-iodobenzoic acid (2v)

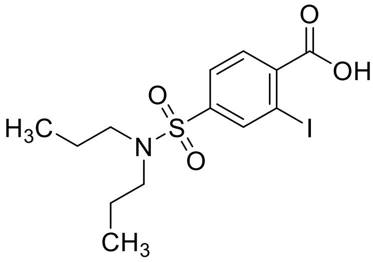

Method C. The crude material was dissolved in DMSO and the product purified by preparative HPLC, gradient 28-68 %, to provide 4-(*N*,*N*-dipropylsulfamoyl)-2-iodobenzoic acid (**2v**) as an off-white solid (106.9 mg, 52 %).

^{1}H NMR (500 MHz, CD_3OD): δ 8.33 (s, 1H), 7.92-7.86 (m, 2H), 3.12 (t, J = 7.5 Hz, 4H), 1.60-1.52 (m, 4H), 0.89 (t, J = 7.5 Hz, 6H)

^{13}C NMR (126 MHz, CD_3OD): δ 169.1, 144.4, 142.1, 140.0, 131.9, 127.5, 94.2, 51.2, 23.1, 11.4

HRMS (ESI) m/z calcd. for $C_{13}H_{18}INO_4S$ $[M+H]^+$: 412.0079, found 412.0078

2-iodo-5-methylbenzoic acid (2w)[10]

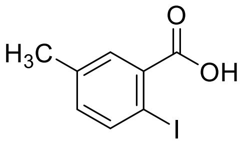

Method A. The crude material was dissolved in DMSO and the product purified by preparative HPLC, gradient 18-58 %, to provide 2-iodo-5-methylbenzoic acid (**2w**) as a colorless solid (112.7 mg, 86 %).

^{1}H NMR (500 MHz, CD_3OD): δ 7.85 (d, J = 8.3 Hz, 1H), 7.61 (d, J = 1.9 Hz, 1H), 7.05-7.01 (m, 1H), 2.33 (s, 3H)

^{13}C NMR (126 MHz, CD_3OD): δ 170.2, 142.1, 139.5, 137.6, 134.4, 132.3, 90.2, 20.8

HRMS (ESI) m/z calcd. for $C_8H_7IO_2$ $[M-H]^-$: 260.9412, found 260.9409

2-iodo-5-(trifluoromethyl) benzoic acid (2x)[11]

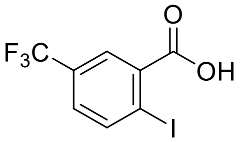

Method C. The crude material was dissolved in DMSO and the product purified by preparative HPLC, gradient 23-63 %, to provide 2-iodo-5-(trifluoromethyl) benzoic acid (**2x**) as a colorless solid (118.5 mg, 75 %).

^{1}H NMR (500 MHz, $CDCl_3$): δ 8.28 (d, 2.1 Hz, 1H), 8.23 (d, 8.3 Hz, 1H), 7.45 (dd, 8.3, 2.3 Hz, 1H)

^{13}C NMR (126 MHz, $CDCl_3$): δ 169.9, 143.1, 133.9, 131.0 (q, J_{C-F} = 34.0 Hz,), 129.9 (q, J_{C-F} = 3.6 Hz,), 128.9 (q, J_{C-F} = 3.9 Hz,), 123.5 (q, J_{C-F} = 272.9 Hz,), 99.5 (q, J_{C-F} = 1.3 Hz,)

^{19}F NMR (470 MHz, $CDCl_3$): δ -63.15 (s)

HRMS (ESI) m/z calcd. for $C_8H_4F_3IO_2$ $[M\text{-}H]^-$: 314.9130, found 314.9132

5-(difluoromethyl)-2-iodobenzoic acid (2y)

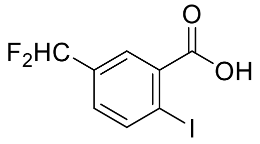

Method A. The crude material was dissolved in DMSO and the product purified by preparative HPLC, gradient 37-77 %, to provide 5-(difluoromethyl)-2-iodobenzoic acid (**2y**) as a colorless solid (71.5 mg, 48 %).

^{1}H NMR (500 MHz, CD_3OD): δ 8.15 (d, J = 8.1 Hz, 1H), 7.96-7.91 (m, 1H), 7.39-7.35 (m, 1H), 6.80 (t, J = 56.3 Hz, 1H)

^{13}C NMR (126 MHz, CD_3OD): δ 169.2, 143.1, 138.4, 136.2 (t, $J_{C\text{-}F}$ = 23.3 Hz, 1C), 130.2 (t, $J_{C\text{-}F}$ = 5.7 Hz,), 128.7 (t, $J_{C\text{-}F}$ = 6.3 Hz, , 115.4 (t, $J_{C\text{-}F}$ = 237.7 Hz,), 97.3 (t, $J_{C\text{-}F}$ = 2.5 Hz,)

^{19}F NMR (470 MHz, CD_3OD): δ -113.48 (s)

HRMS (ESI) m/z calcd. for $C_8H_5F_2IO_2$ $[M\text{-}H]^-$: 296.9224, found 296.9224

5-acetyl-2-iodobenzoic acid (2z)

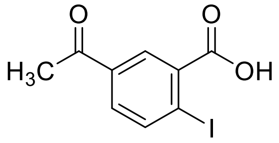

Method C. The crude material was dissolved in DMSO and the product purified by preparative HPLC, gradient 10-50 %, to provide 5-acetyl-2-iodobenzoic acid (**2z**) as a colorless solid (101.5 mg, 70 %).

^{1}H NMR (500 MHz, CD_3OD): δ 8.32 (d, J = 2.2 Hz, 1H), 8.17 (d, J = 8.3 Hz, 1H), 7.76 (dd, 8.3, 2.2 Hz, 1H), 2.60 (s, 3H)

^{13}C NMR (126 MHz, CD_3OD): δ 198.8, 169.3, 143.1, 138.4, 138.0, 132.4, 130.9, 100.9, 26.6

HRMS (ESI) m/z calcd. for $C_9H_7IO_3$ $[M\text{-}H]^-$: 288.9362, found 288.9364

2-iodo-5-(methoxycarbonyl) benzoic acid (2aa)[10]

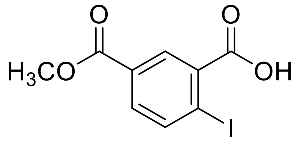

Method A. The crude material was dissolved in DMSO and the product purified by preparative HPLC, gradient 37-77 %, to provide 2-iodo-5-(methoxycarbonyl) benzoic acid (**2aa**) as a colorless solid (73.4 mg, 48 %).

^{1}H NMR (500 MHz, CD_3OD): δ 8.36 (d, *J* = 2.2 Hz, 1H), 8.16 (d, *J* = 8.0 Hz, 1H), 7.77 (dd, *J* = 8.0, 2.2 Hz, 1H), 3.92 (s, 3H)

^{13}C NMR (126 MHz, CD_3OD): δ 169.1, 167.2, 143.1, 138.2, 133.5, 132.1, 131.4, 100.8, 53.0

HRMS (ESI) m/z calcd. for $C_9H_7IO_4$ $[M-H]^-$: 304.9311, found 304.9311

3-fluoro-2-iodobenzoic acid (2ab)[11]

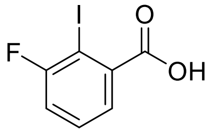

Method A. The crude material was dissolved in DMSO and the product purified by preparative HPLC, gradient 37-77 %, to provide 3-fluoro-2-iodobenzoic acid (**2ab**) as a colorless solid (101.1 mg, 76 %).

^{1}H NMR (500 MHz, CD_3OD): δ 7.55 (dd, *J* = 7.7, 0.9 Hz, 1H), δ 7.44 (td, *J* = 8.0, 5.3 Hz, 1H), δ 7.26 (td, *J* = 8.3, 1.4 Hz, 1H)

^{13}C NMR (126 MHz, CD_3OD) δ 169.4 (d, *J* = 2.8 Hz,), 163.6 (d, *J* = 245.2 Hz,), 140.7 (d, *J* = 1.4 Hz,), 131.2 (d, *J* = 8.3 Hz,), 127.1 (d, *J* = 3.1 Hz,), 118.6 (d, *J* = 25.8 Hz), 82.7 (d, *J* = 27.7 Hz,)

^{19}F NMR (470 MHz, CD_3OD): δ -89.98 (s)

HRMS (ESI) m/z calcd. for $C_7H_4FIO_2$ $[M-H]^-$: 264.9162, found 264.9160

3-chloro-2-iodobenzoic acid (2ac),[12] 5-chloro-2-iodobenzoic acid (2ad)[10]

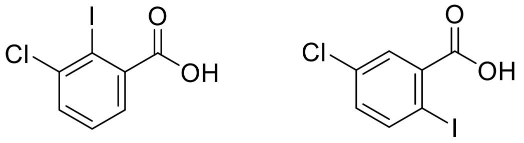

Method A. The crude material was dissolved in DMSO and the product purified by preparative HPLC, gradient 37-77 %, to provide 3-chloro-2-iodobenzoic acid (**2ac**) as a colorless solid (81.8 mg, 58 %) and 5-chloro-2-iodobenzoic acid (**2ad**) as a colorless solid (36.7 mg, 26 %).

(**2ac**)

^{1}H NMR (500 MHz, CD_3OD): δ 7.62 (dd, *J* = 7.8, 1.6 Hz, 1H), 7.48 (dd, *J* = 7.8, 1.6 Hz, 1H), 7.41 (t, *J* = 7.8 Hz, 1H)

^{13}C NMR (126 MHz, CD_3OD) δ 170.8, 143.4, 141.6, 131.9, 130.5, 128.2, 97.9

HRMS (ESI) m/z calcd. for $C_7H_4ClIO_2$ $[M-H]^-$: 280.8866, found 280.8864

(**2ad**)

^{1}H NMR (500 MHz, CD_3OD): δ 7.97 (d, *J* = 8.5 Hz, 1H), 7.77 (d, *J* = 2.6 Hz, 1H), 7.23 (dd, *J* = 8.5, 2.6 Hz, 1H)

^{13}C NMR (126 MHz, CD_3OD) δ 168.8, 143.8, 139.6, 135.5, 133.4, 131.3, 91.8

HRMS (ESI) m/z calcd. for $C_7H_4ClIO_2$ $[M-H]^-$: 280.8866, found 280.8870

3-bromo-2-iodobenzoic acid (2ae),[12] 5-bromo-2-iodobenzoic acid (2af)[13]

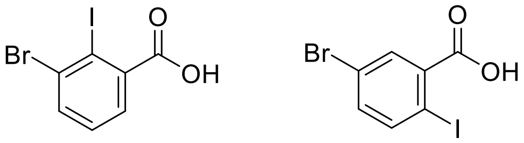

Method A. The crude material was dissolved in DMSO and the product purified by preparative HPLC, gradient 37-77 %, to provide 3-bromo-2-iodobenzoic acid (**2ae**) as an off-white solid (96.1 mg, 59 %) and 5-bromo-2-iodobenzoic acid (**2ae**) as an off-white solid (52.1 mg, 32 %).

(**2ae**)

^{1}H NMR (500 MHz, CD_3OD): δ 7.79 (dd, *J* = 8.0, 1.5 Hz, 1H), 7.48 (dd, *J* = 7.5, 1.5 Hz, 1H), 7.34 (t, *J* = 7.8 Hz, 1H)

^{13}C NMR (126 MHz, CD_3OD) δ 171.1, 144.0, 135.3, 133.2, 130.6, 128.3, 100.6

HRMS (ESI) m/z calcd. for $C_7H_4BrIO_2$ $[M-H]^-$: 324.8361, found 324.8365

(**2af**)

^{1}H NMR (500 MHz, CD_3OD): δ 7.90 (m, 2H), 7.36 (dd, *J* = 8.5, 2.3 Hz, 1H)

^{13}C NMR (126 MHz, CD_3OD) δ 168.5, 144.0, 139.6, 136.4, 134.3, 123.1, 92.6

HRMS (ESI) m/z calcd. for $C_7H_4BrIO_2$ $[M-H]^-$: 324.8361, found 324.8361

4-(trifluoromethyl) benzoic-2,6-*d*2 acid (1l-D2)

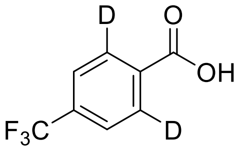

4-(trifluoromethyl) benzoic acid (**1l**) (760 mg, 4.0 mmol), $[Cp^*IrCl_2]_2$ (63.7 mg, 2 mol%) were suspended in D_2O (20 mL) in a microwave vial. The contents were sonicated for 2 minutes, after which the mixture was heated in a microwave reactor at 170 °C for 1 hour. The resulting mixture was extracted with CH_2Cl_2 (3 x 30 mL). The Organic phase was dried over $MgSO_4$ and concentrated *in vacuo*. The residue was dissolved in Et_2O (20 mL), upon which most of the present catalyst precipitated. The solution was then applied on a silica plug (10 g) and eluted with EtOAc/heptane/AcOH (60/38/2, 70 mL). The volatiles were evaporated to provide 4-(trifluoromethyl) benzoic-2,6-*d*2 acid (**1l-D2**) as a colorless solid (634 mg, 82%).

^{1}H NMR (500 MHz, CD_3OD): δ 7.79 (s, 2H)

^{13}C NMR (126 MHz, CD_3OD): δ 168.2, 135.6, 135.2 (q, $J_{C\text{-}F}$ = 32.1 Hz, 1C), 131.1 (t, $J_{C\text{-}D}$ = 25.8 Hz, 2C), 128.5 (2C), 125.3 (q, $J_{C\text{-}F}$ = 270.4 Hz,1C)

^{19}F NMR (470 MHz, CD_3OD): δ -64.59 (s)

HRMS (ESI) m/z calcd. for $C_8H_3D_2F_3O_2$ $[M-H]^-$: 191.0294, found 191.0300

$Cp^{*OCH(CF3)2}Ir(O_2CC_6H_4)$ (Ic-2)

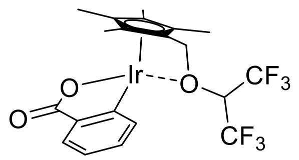

$Cp^{*OCH(CF3)2}$ = η^5-$C_5Me_4CH_2OCH(CF_3)_2$

Benzoic acid (**1a**) (12.2 mg, 0.1 mmol) was dissolved in 1,1,1,3,3,3-hexafluoro-2-propanol (HFIP, 1 mL, 0.1 M), followed by addition of Et_3N (55.8 µL, 0.4 mmol). In an amber vial $[Cp^*IrCl_2]_2$ (42.4 mg, 53 mol%) and AgOAc (61.8 mg, 0.37 mmol) were added. The substrate (**1a**) solution was added to this mixture and the mixture was stirred vigorously at 23 °C for 1 hour. After this an aliquot (200 µL) was taken from the reaction mixture and added to C_6D_6 (0.6 mL) in an NMR tube. Subsequent ^1H NMR experiment showed a single major species (**Ic-2**). The remaining reaction mixture was filtered through a glass syringe filter (0.45 µm pore size) and the complex purified by preparative supercritical fluid chromatography (prep-SFC) (gradient?). Fractions containing the product were pooled and the volatiles removed with a flow of nitrogen, to provide **Ic-2** as a yellow solid (15.4 mg, 24%).

^1H NMR (500 MHz, CD_3OD): δ 7.65 (d, *J* = 7.5 Hz, 1H), 7.46 (dd, *J* = 7.5, 1.5 Hz, 1H), 7.21 (td, *J* = 7.5, 1.5 Hz, 1H), 6.99 (td, *J* = 7.5, 1.5 Hz, 1H), 5.00 (heptet, *J* = 6.3, 1H), 4.55 (s, 2H), 1.82-1.75 (m, 12H)

^1H NMR (500 MHz, C_6D_6): δ 8.05 (dd, *J* = 7.5, 1.1 Hz, 1H), 7.55 (dd, *J* = 7.5, 0.8 Hz, 1H), 7.25 (td, *J* = 7.5, 1.5 Hz, 1H), 7.13 (td, *J* = 7.5, 1.0 Hz, 1H), 4.49 (d, *J* = 10.5 Hz, 1H), 4.24 (d, *J* = 10.5 Hz, 1H), 3.88 (heptet, 6.0 Hz), 1.63 (s, 3H), 1.53 (s, 3H), 1.36 (s, 3H), 1.25 (s, 3H)

^{13}C NMR (126 MHz, C_6D_6): δ 184.5, 157.52, 139.5, 134.7, 132.3, 130.1, 123.7, 123.7-123.4 (br, 1C), 121.5-121.1 (br, 1C), 96.1, 92.2, 84.8, 82.1, 75.6, 69.1, 8.8, 8.6, 8.4, 8.2

^{19}F NMR (470 MHz, C_6D_6): δ -73.9—-74.0 (m)

HRMS (ESI) m/z calcd. for $C_{20}H_{19}F_6IrO_3$ $[M+H]^+$: 613.0923, found 613.0931

Mechanistic studies

Deuterium incorporation under standard conditions

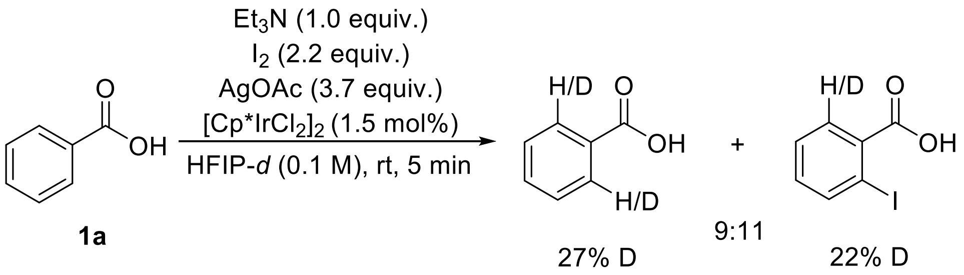

Benzoic acid (**1a**, 12.2 mg, 0.1 mmol) was dissolved in 1,1,1,3,3,3-hexafluoro-2-propanol-OD (HFIP-*d*, 1.0 mL, 0.1 M), followed by addition of Et_3N (13.9 µL, 0.1 mmol). In a separate amber vial $[Cp^*IrCl_2]_2$ (1.2 mg, 1.5 mol%) and AgOAc (61.8 mg, 0.37 mmol) were added. The substrate (**1a**) solution was added to this mixture, followed by addition of I_2 (55.8 mg, 0.22 mmol), and stirred vigorously at room temperature (23 °C) for 5 minutes. After this, Na_2SO_3 (100 mg) and water (1 mL) were added and the mixture shaked. The mixture was then partitioned between aqueous HCl (1.0 M, 8 mL) and EtOAc (10 mL). The aqueous phase was extracted with EtOAc (4 x 10 mL). The combined organic phases were washed with brine (10 mL), dried over $MgSO_4$ and concentrated under reduced pressure. The residue was dissolved in CD_3OD (3 mL). Deuterium incorporation to the *ortho* position of **2a** (22% D) and conversion (55%) was established by ^{1}H NMR. Starting material **1a** incorporated 27% D (^{1}H NMR). Percentage of mono- and dideuteration established by ion count from direct injection HRMS (adjusted to ^{13}C content), **1a$_{D1}$** (33%), **1a$_{D2}$** (9%).

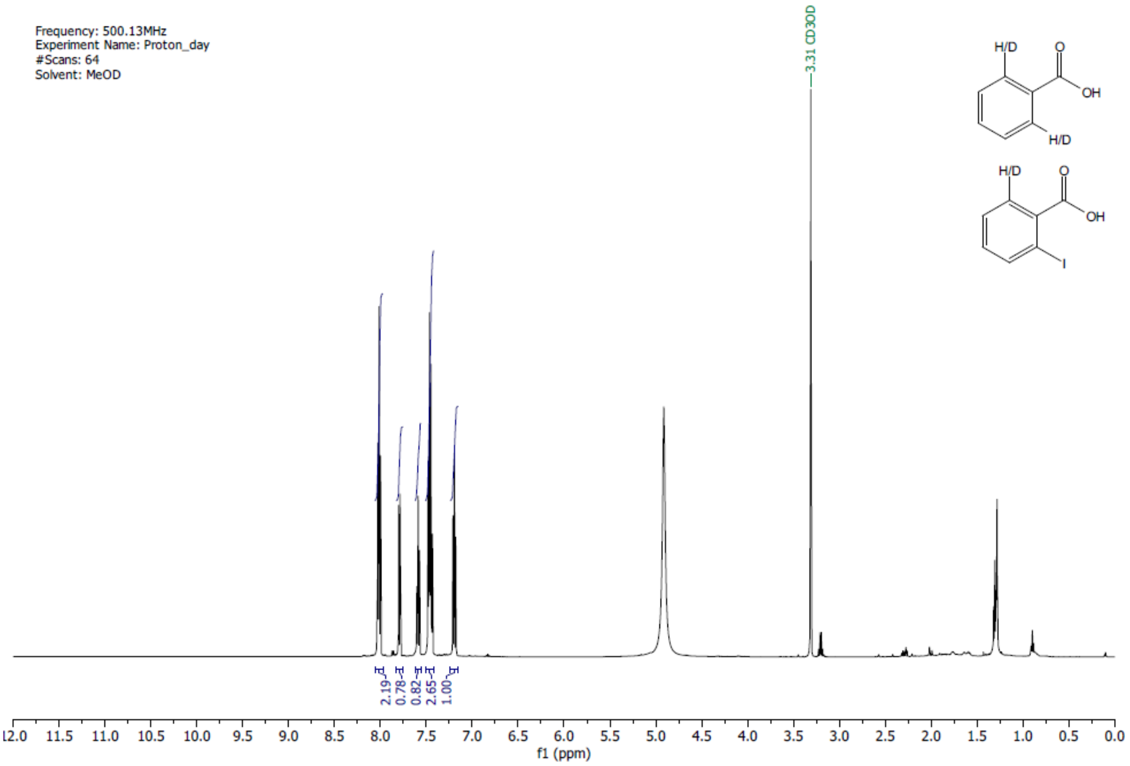

Deuterium incorporation – substituent effect

ortho-Substituent effect

Table S7. *ortho*-substituent effect on deuterium incorporation

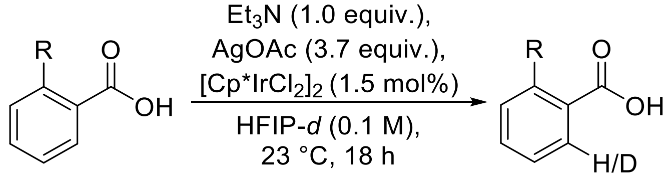

Entry	R	D incorporation
1a	H	45%
2a	Me	83%
3a	F	69%
4a	Cl	34%
5a	Br	35%
6a	I	5%
7a	*t*Bu	40%

Substrate (0.1 mmol) was dissolved in 1,1,1,3,3,3-hexafluoro-2-propanol-OD (HFIP-*d*, 1.0 mL, 0.1 M), followed by addition of Et_3N (13.9 µL, 0.1 mmol). In a separate amber vial $[Cp^*IrCl_2]_2$ (1.2 mg, 1.5 mol%) and AgOAc (61.8 mg, 0.37 mmol) were added. The substrate solution was added to this mixture and stirred vigorously at room temperature (23 °C) for 18 hours. The mixture was then partitioned between aqueous HCl (1.0 M, 8 mL) and EtOAc (10 mL). The aqueous phase was extracted with EtOAc (4 x 10 mL). The combined organic phases were washed with brine (10 mL), dried over $MgSO_4$ and concentrated under reduced pressure. The residue was dissolved in DCM and applied on a silica plug (2.0 g). The plug was washed with heptane/EtOAc (1:1, 2% AcOH, 30 mL). The organic phase was concentrated under reduced pressure. The residue was dissolved in CD_3OD (3 mL). Deuterium incorporation determined by ^1H NMR (Table S7).

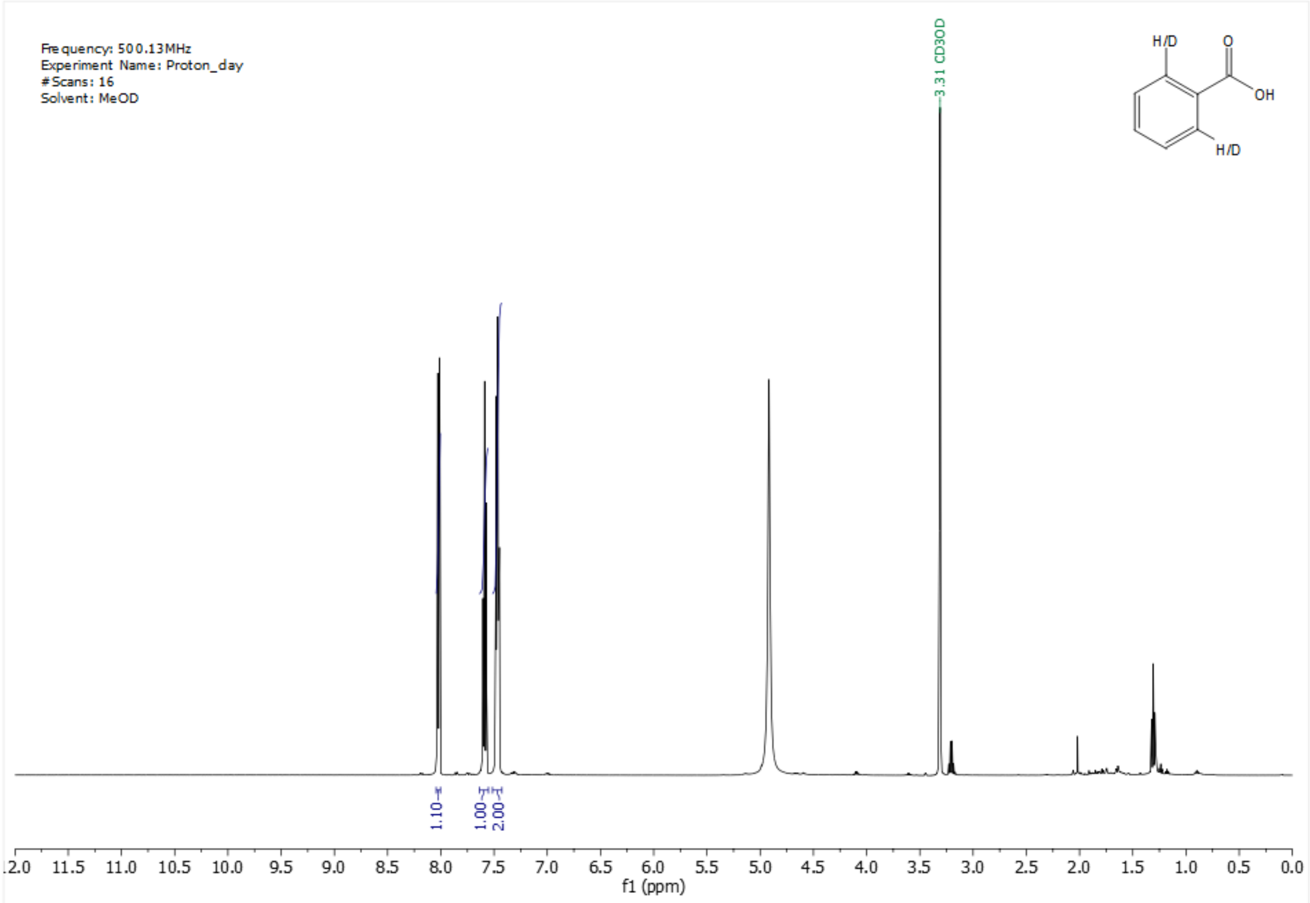

Frequency: 500.13MHz
Experiment Name: Proton_day
#Scans: 16
Solvent: MeOD
3.31 CD3OD
H/D
O
OH
H/D
1.10
1.00
2.00
12.0 11.5 11.0 10.5 10.0 9.5 9.0 8.5 8.0 7.5 7.0 6.5 6.0 5.5 5.0 4.5 4.0 3.5 3.0 2.5 2.0 1.5 1.0 0.5 0.0
f1 (ppm)

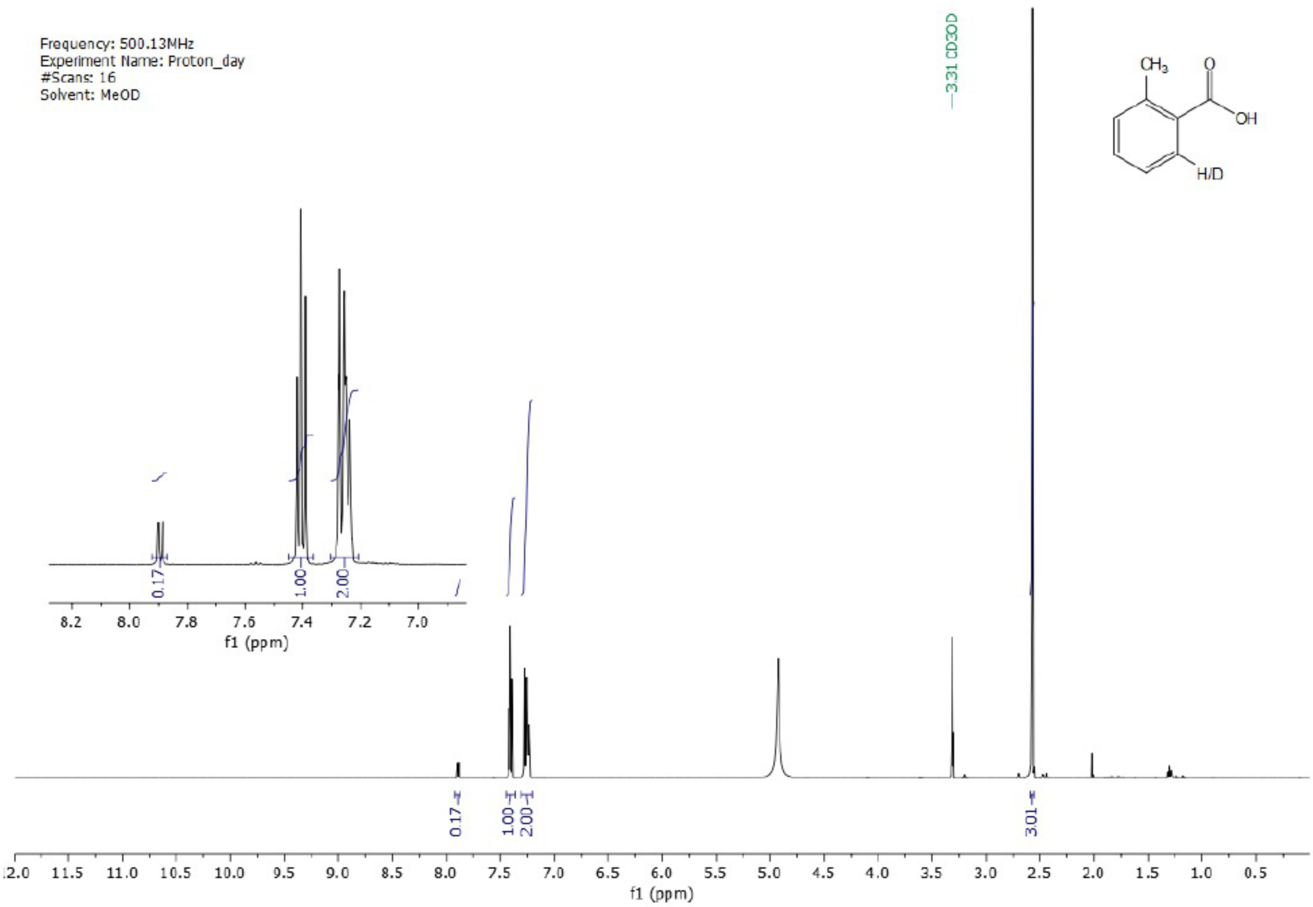
Frequency: 500.13MHz
Experiment Name: Proton_day
#Scans: 16
Solvent: MeOD
3.31 CD3OD
CH3
O
OH
H/D
0.17
1.00
2.00
3.01
f1 (ppm)

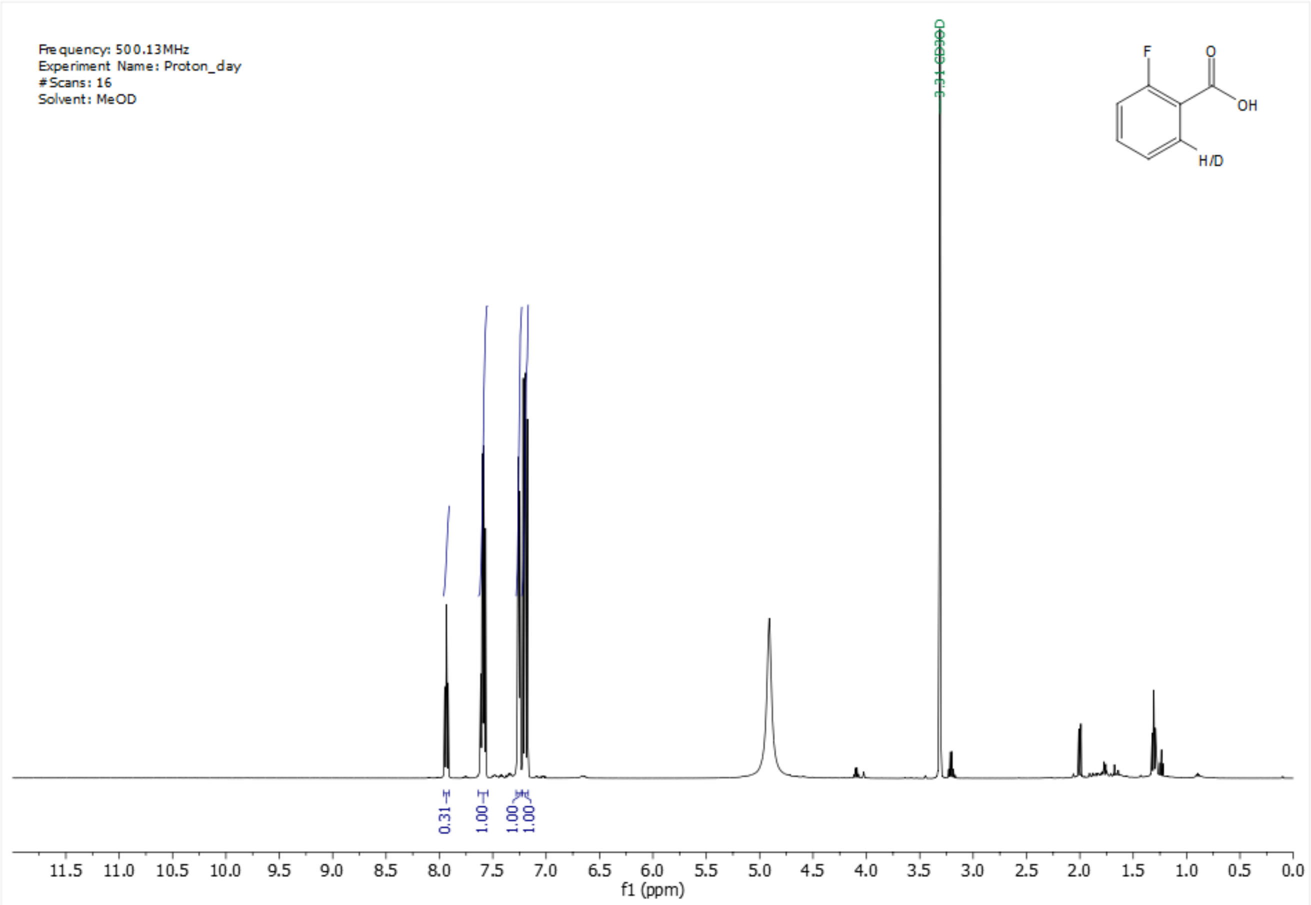
Frequency: 500.13MHz
Experiment Name: Proton_day
#Scans: 16
Solvent: MeOD
3.31 CD3OD
F
O
OH
H/D
0.31
1.00
1.00
1.00
f1 (ppm)

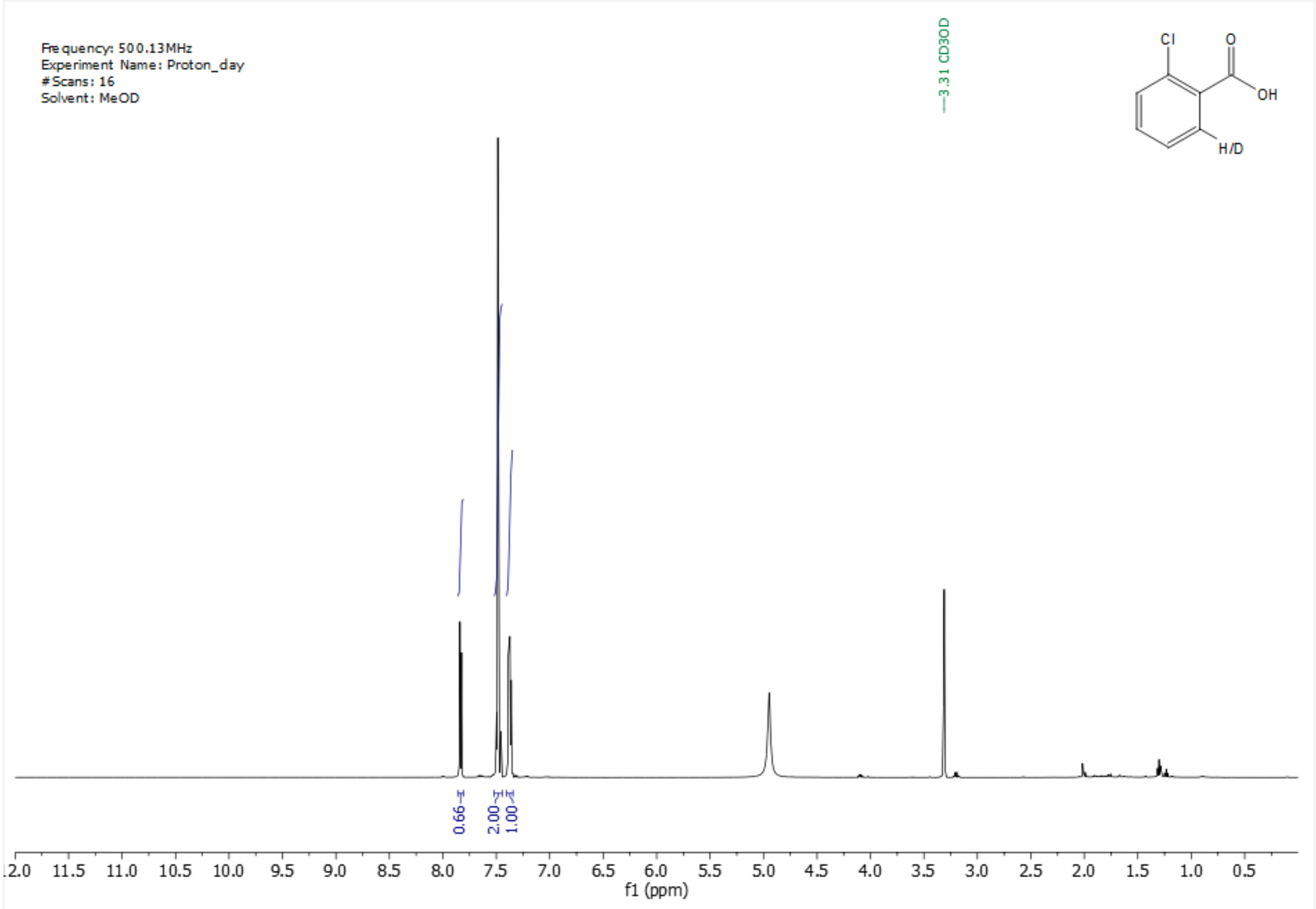
Frequency: 500.13MHz
Experiment Name: Proton_day
#Scans: 16
Solvent: MeOD
3.31 CD3OD
Cl
O
OH
H/D
0.66
2.00
1.00
f1 (ppm)

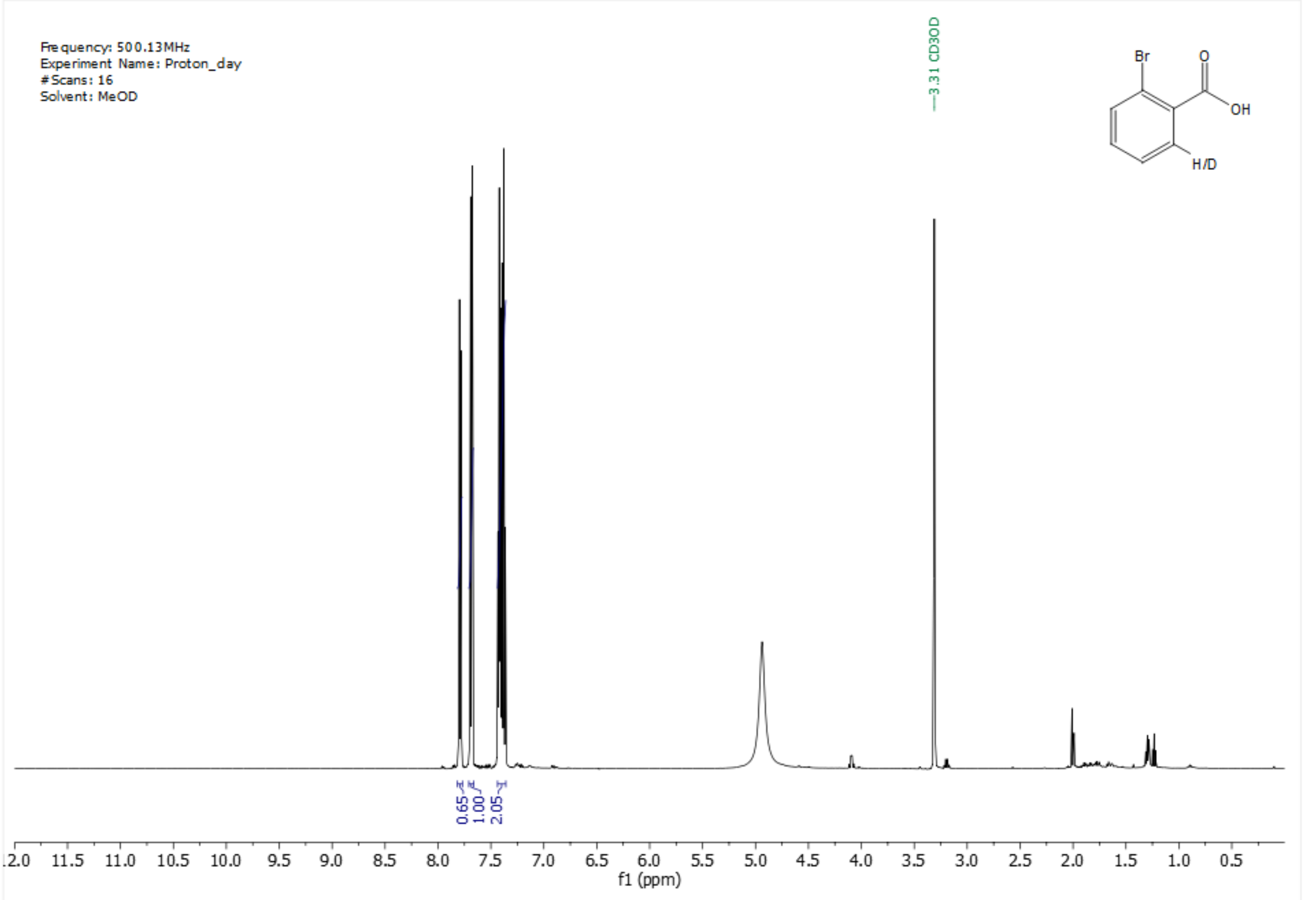
Frequency: 500.13MHz
Experiment Name: Proton_day
#Scans: 16
Solvent: MeOD
3.31 CD3OD
Br
O
OH
H/D
0.65
1.00
2.05
f1 (ppm)

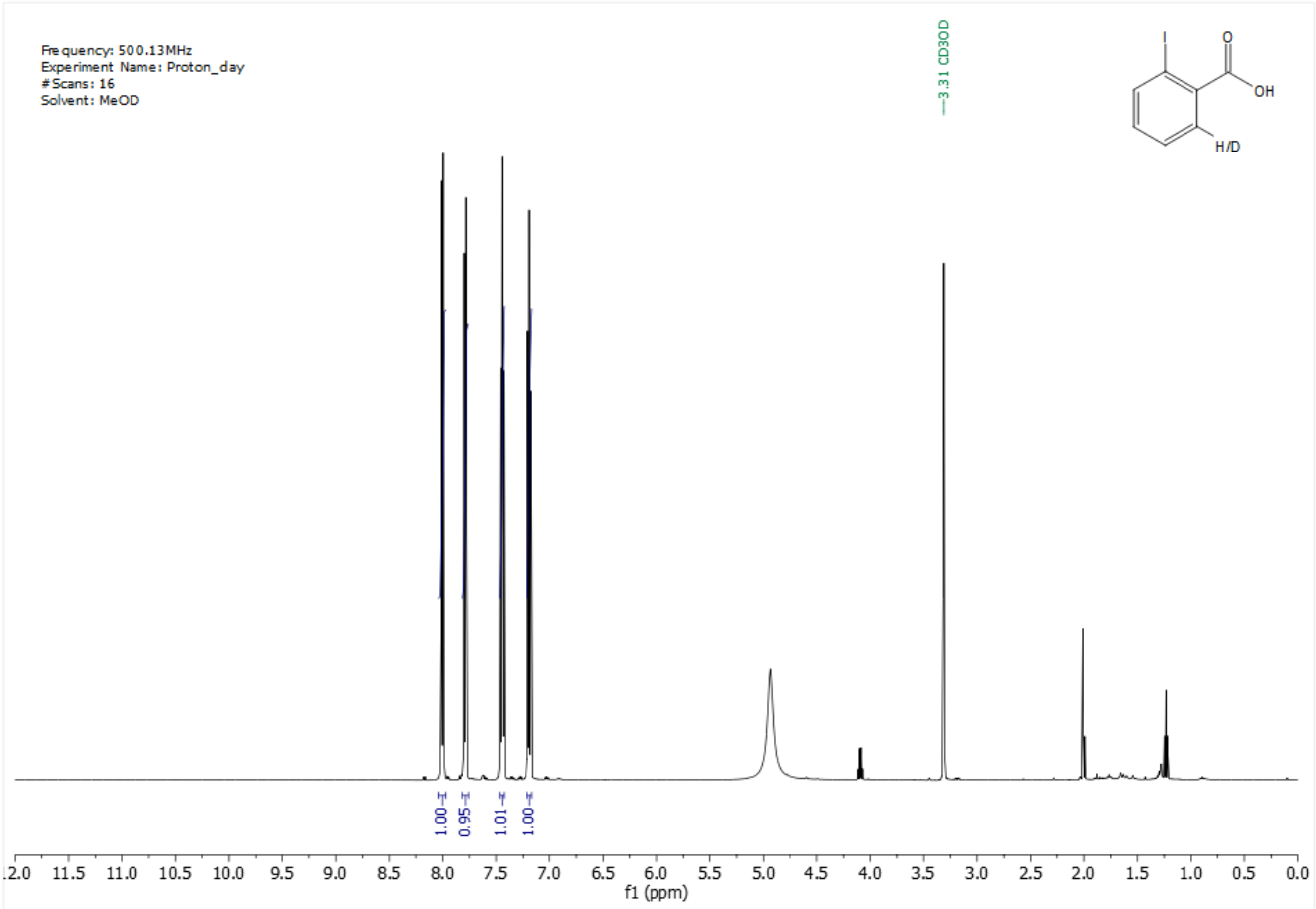
Frequency: 500.13MHz
Experiment Name: Proton_day
#Scans: 16
Solvent: MeOD
3.31 CD3OD
OH
H/D
1.00
0.95
1.01
1.00
f1 (ppm)

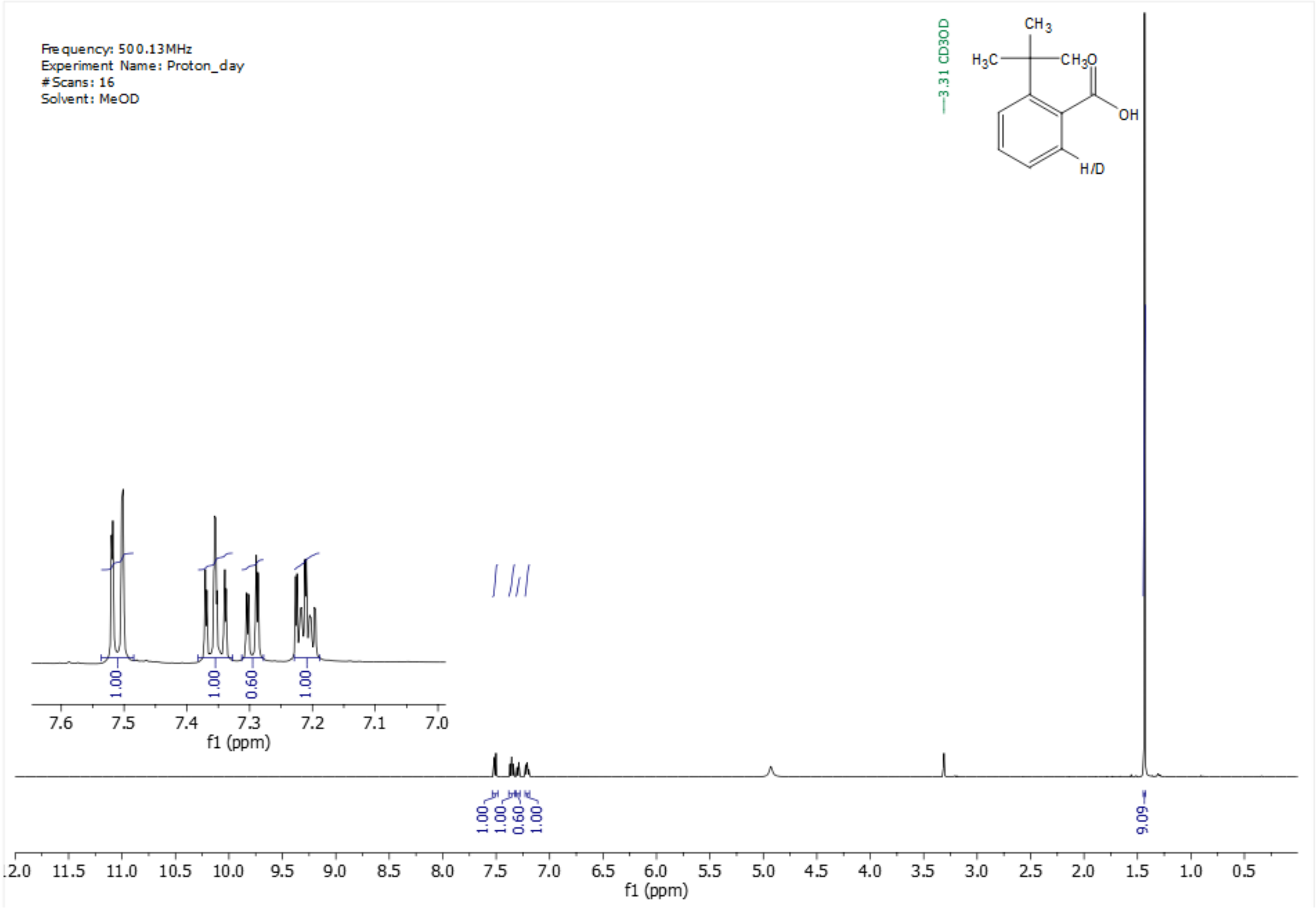
Frequency: 500.13MHz
Experiment Name: Proton_day
#Scans: 16
Solvent: MeOD
3.31 CD3OD
CH3
H3C
CH3
OH
H/D
1.00
1.00
0.60
1.00
9.09
f1 (ppm)

***para*-Substituent effect**

Conditions identical to *ortho*-Substituent effect study. Deuterium incorporation determined by ^{1}H NMR (Table S8). Percentage of mono- and dideuteration established by ion count from direct injection HRMS (adjusted to ^{13}C content, Table S8).

Table S8. *para*-substituent effect on deuterium incorporation

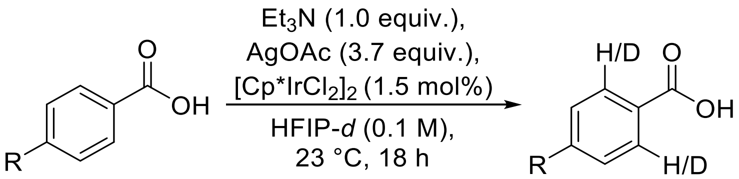

Entry	R	D incorporation	% Mono	% Di
1b	OMe	52%	31	39
2b	H	45%	24	34
3b	F	18%	18	10
4b	CF_3	15%	16	7
5b	NO_2	0%		

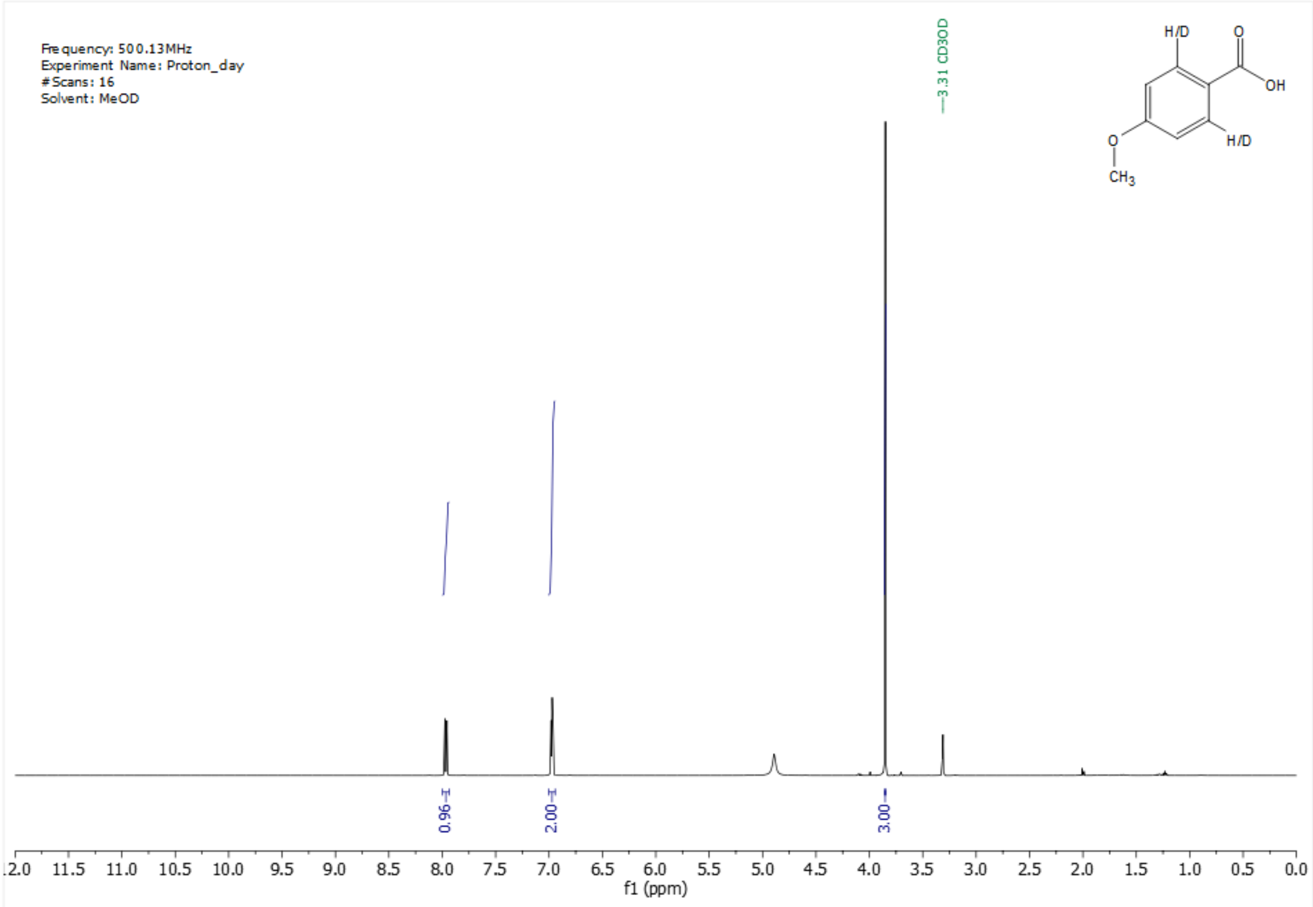

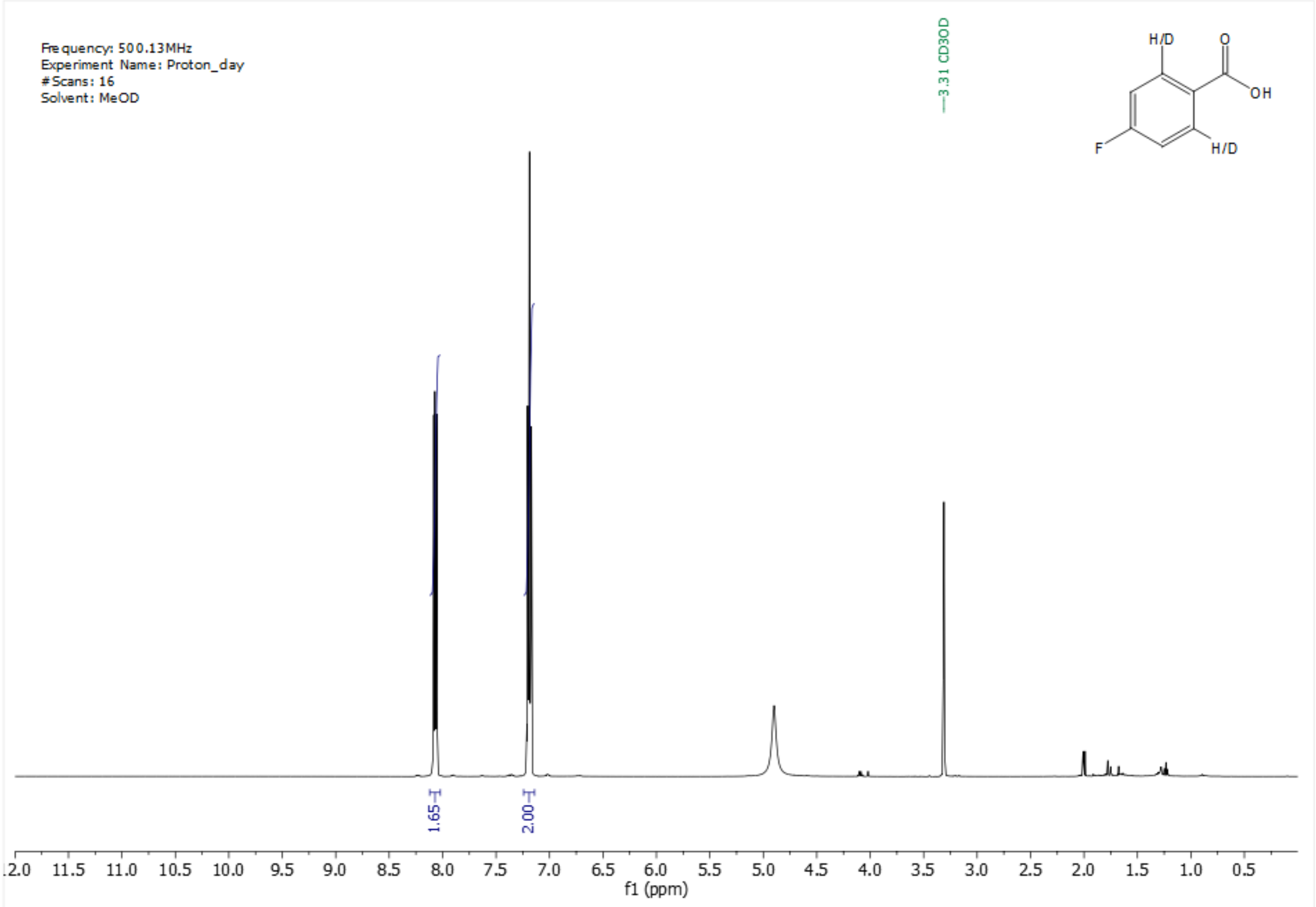
Frequency: 500.13MHz
Experiment Name: Proton_day
#Scans: 16
Solvent: MeOD
3.31 CD3OD
H/D
O
OH
F
H/D
1.65
2.00
f1 (ppm)

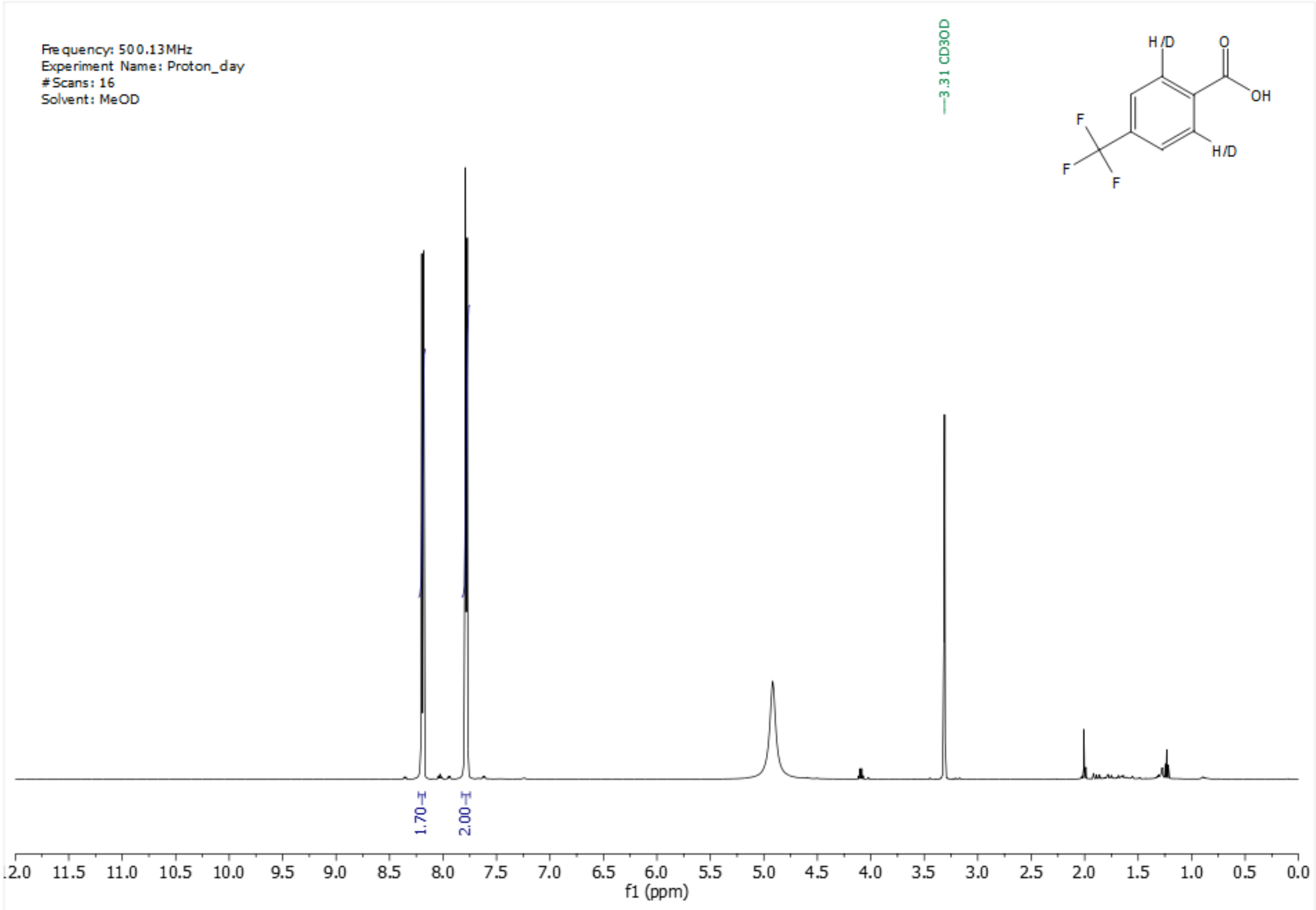
Frequency: 500.13MHz
Experiment Name: Proton_day
#Scans: 16
Solvent: MeOD
3.31 CD3OD
H/D
O
OH
F
F
F
H/D
1.70
2.00
f1 (ppm)

Deuterium incorporation: site selectivity

The site selectivity for the *ortho*-deuteration was confirmed for selected substrates by comparing ^{1}H and ^{2}H NMR spectra. In all representative cases complete selectivity for *ortho*-deuteration was observed, with no deuterium incorporation to other sp^2 and sp^3 C-H bonds. The samples investigated were from the deuterium incorporation study presented above. In Figure S2, top spectrum (purple): ^{2}H; bottom spectrum (black): ^{1}H.

Standard: comparison of commercial BzOH-*d*5 and BzOH

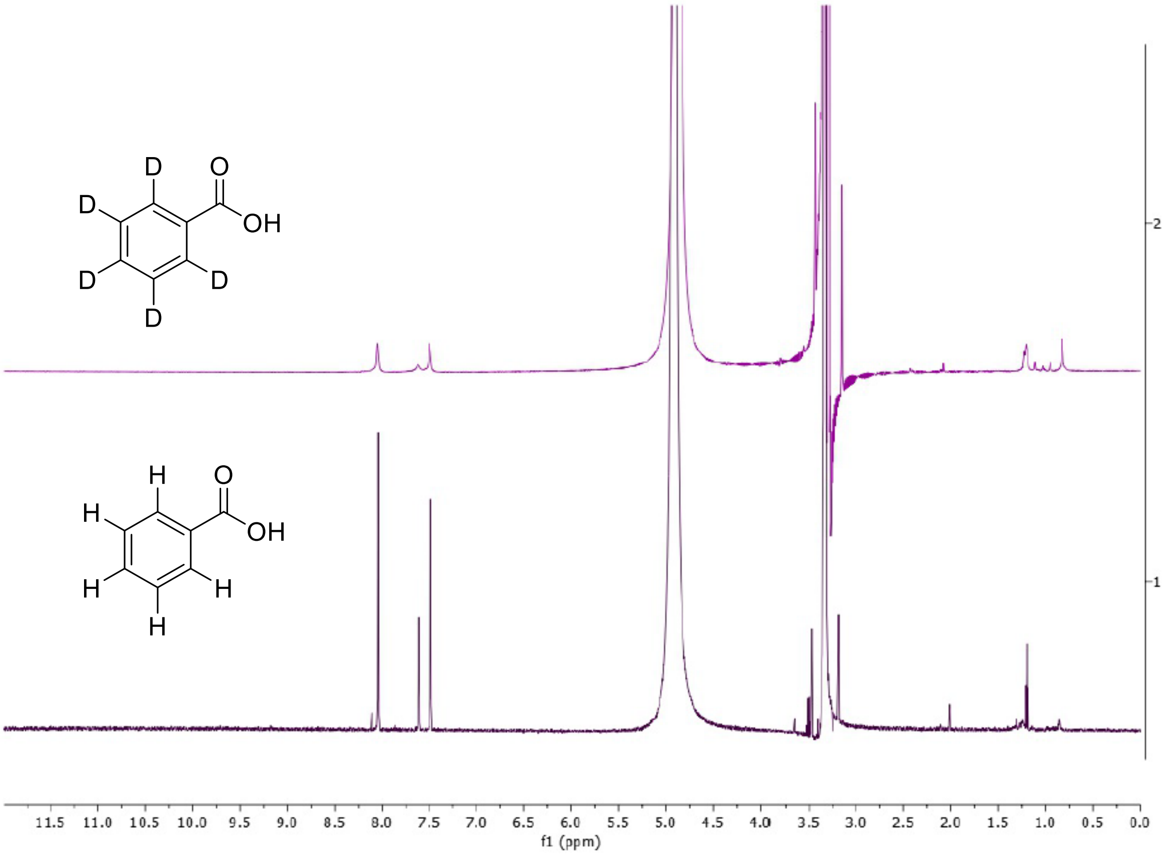

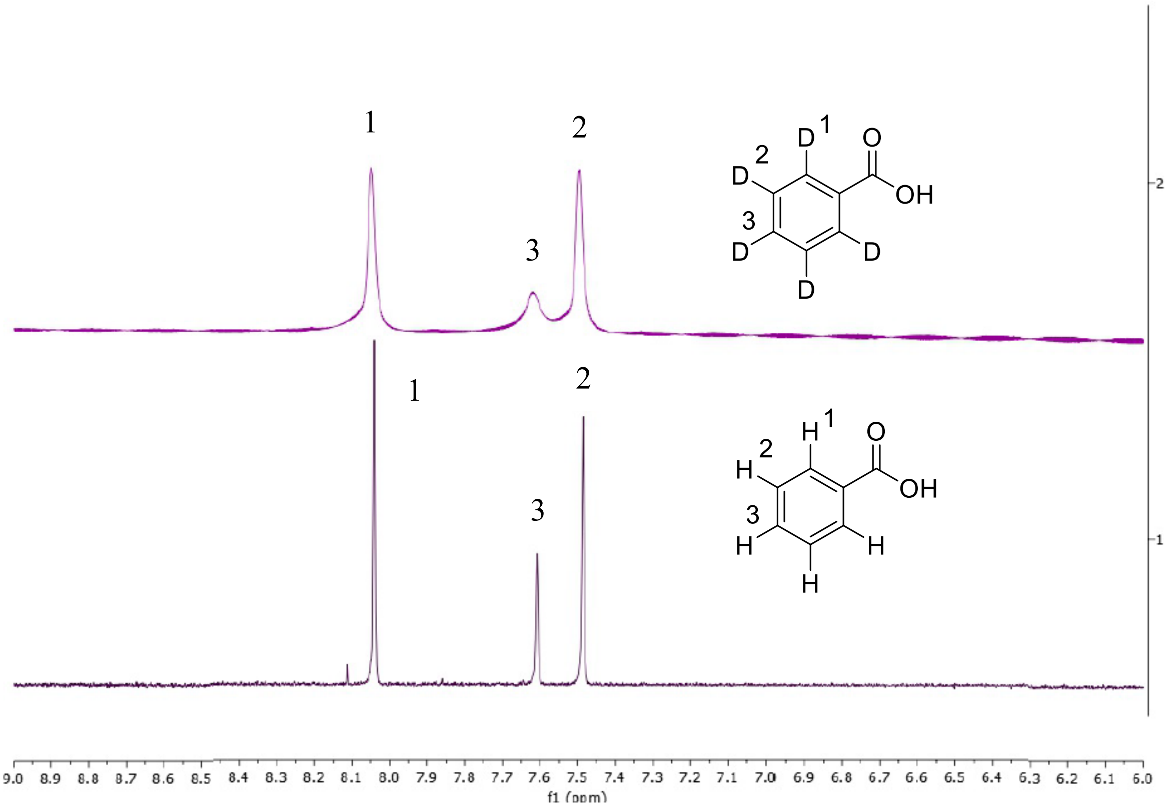

1
2
3
D
D
D
D
D
O
OH
1
2
3
H
H
H
H
H
O
OH
9.0 8.9 8.8 8.7 8.6 8.5 8.4 8.3 8.2 8.1 8.0 7.9 7.8 7.7 7.6 7.5 7.4 7.3 7.2 7.1 7.0 6.9 6.8 6.7 6.6 6.5 6.4 6.3 6.2 6.1 6.0
f1 (ppm)

Entry 1a (Table S7), 2b (Table S8)

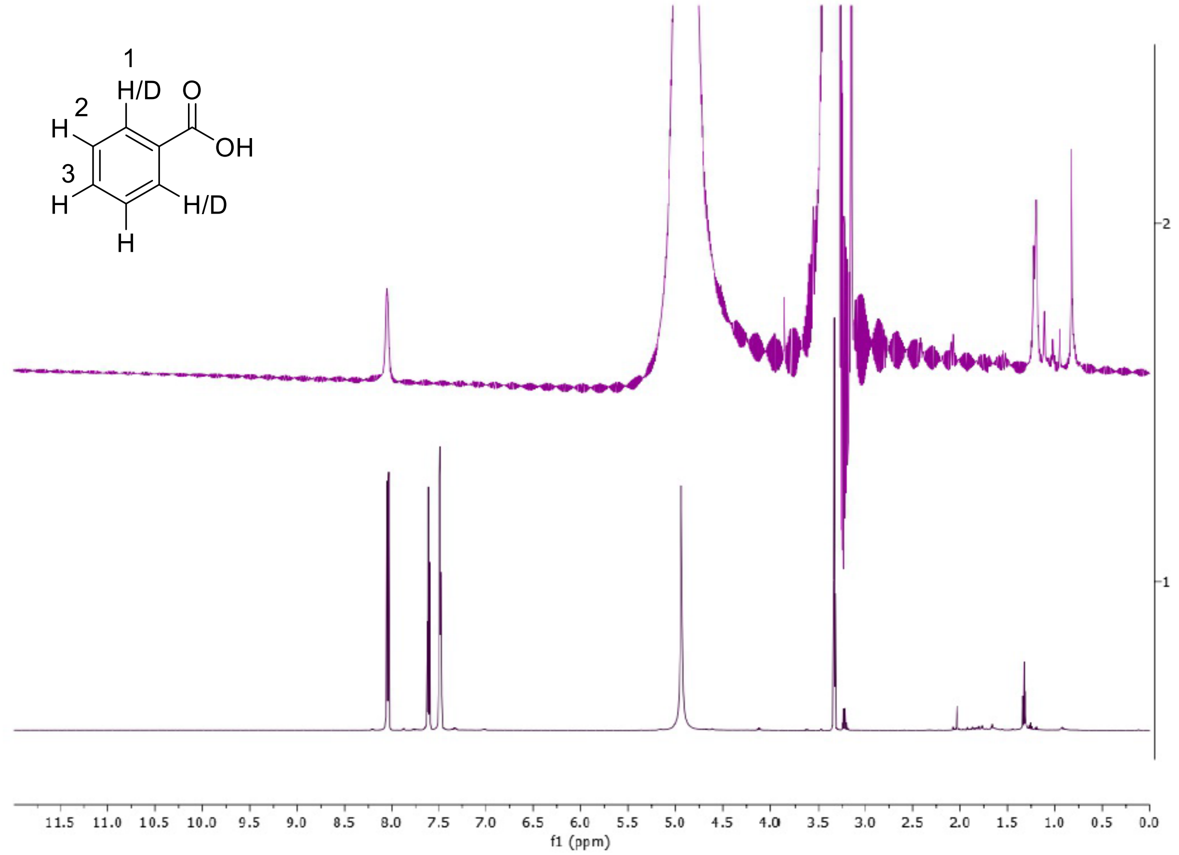

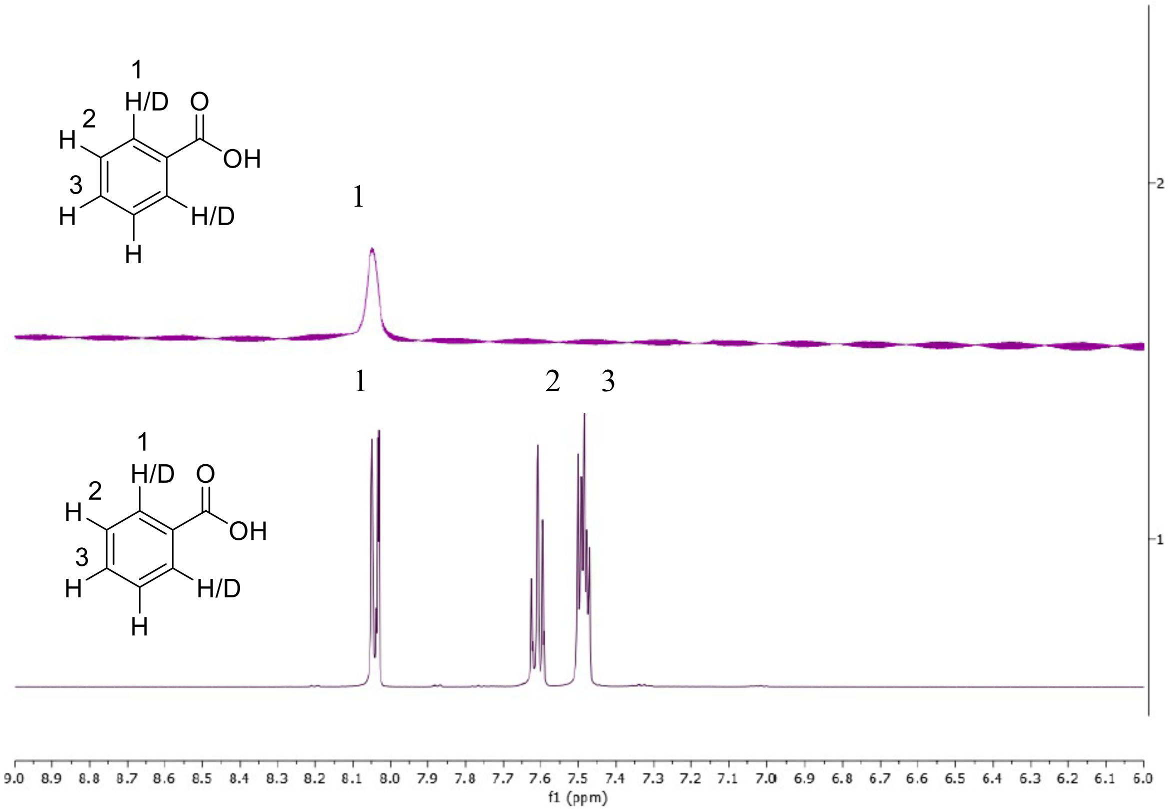

Entry 2a (Table S7)

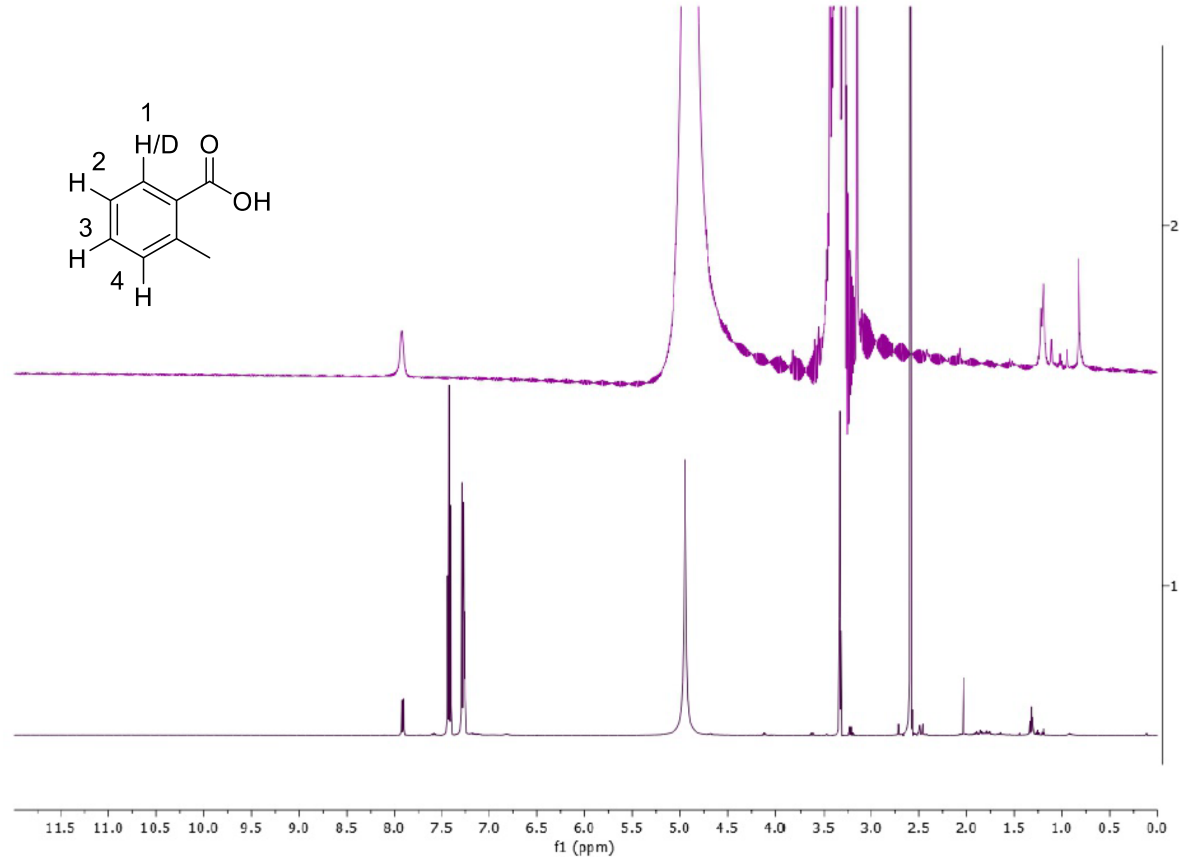

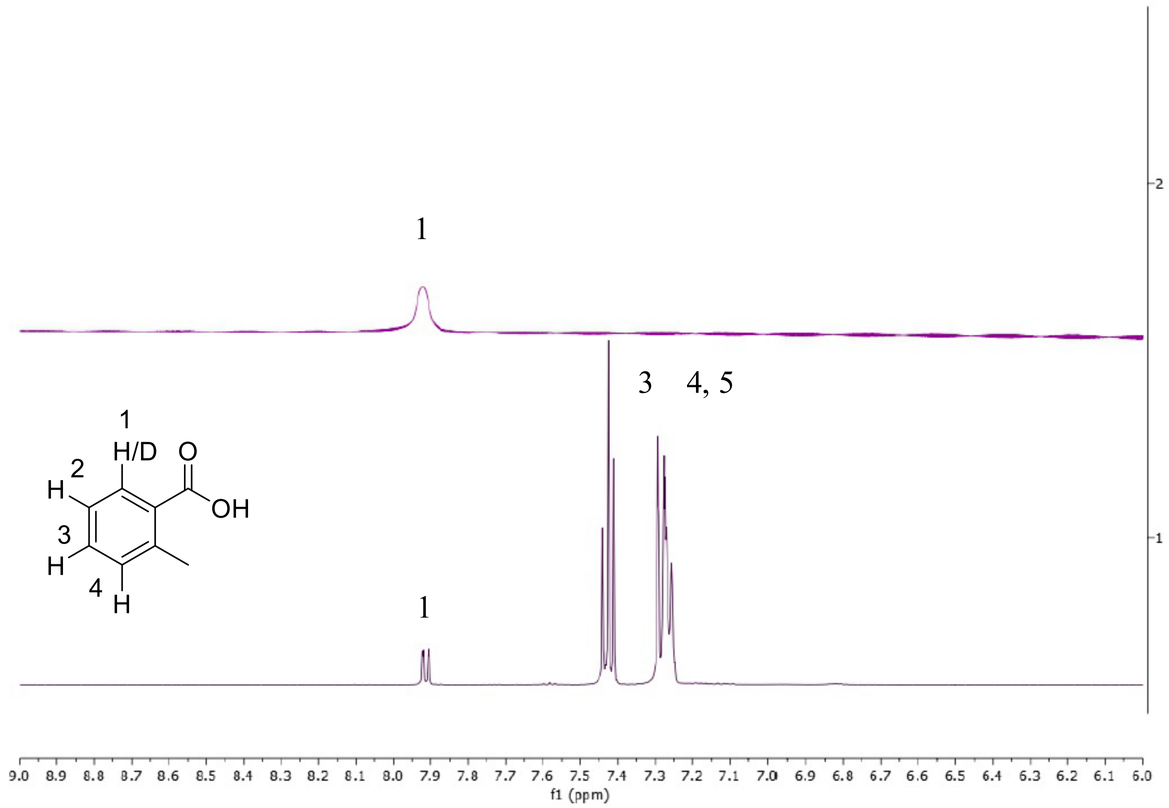

Entry 3a (Table S7)

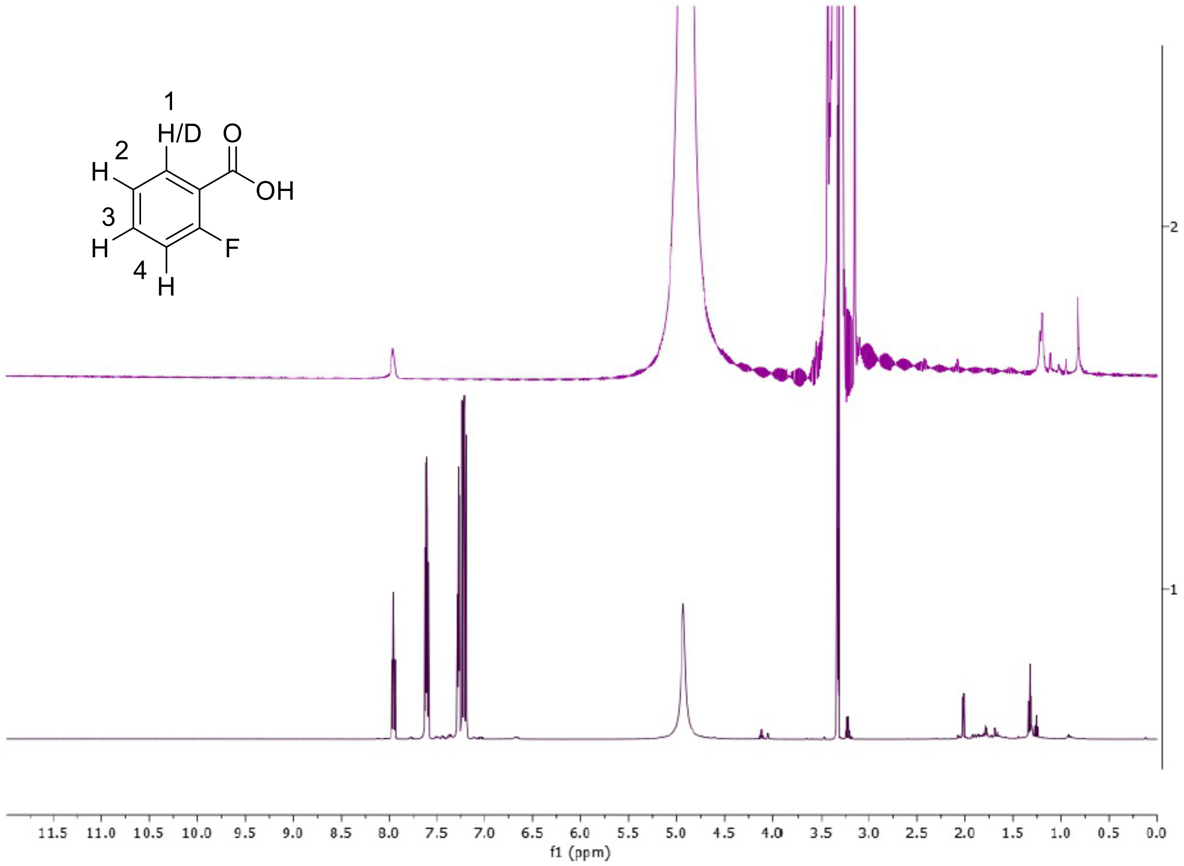

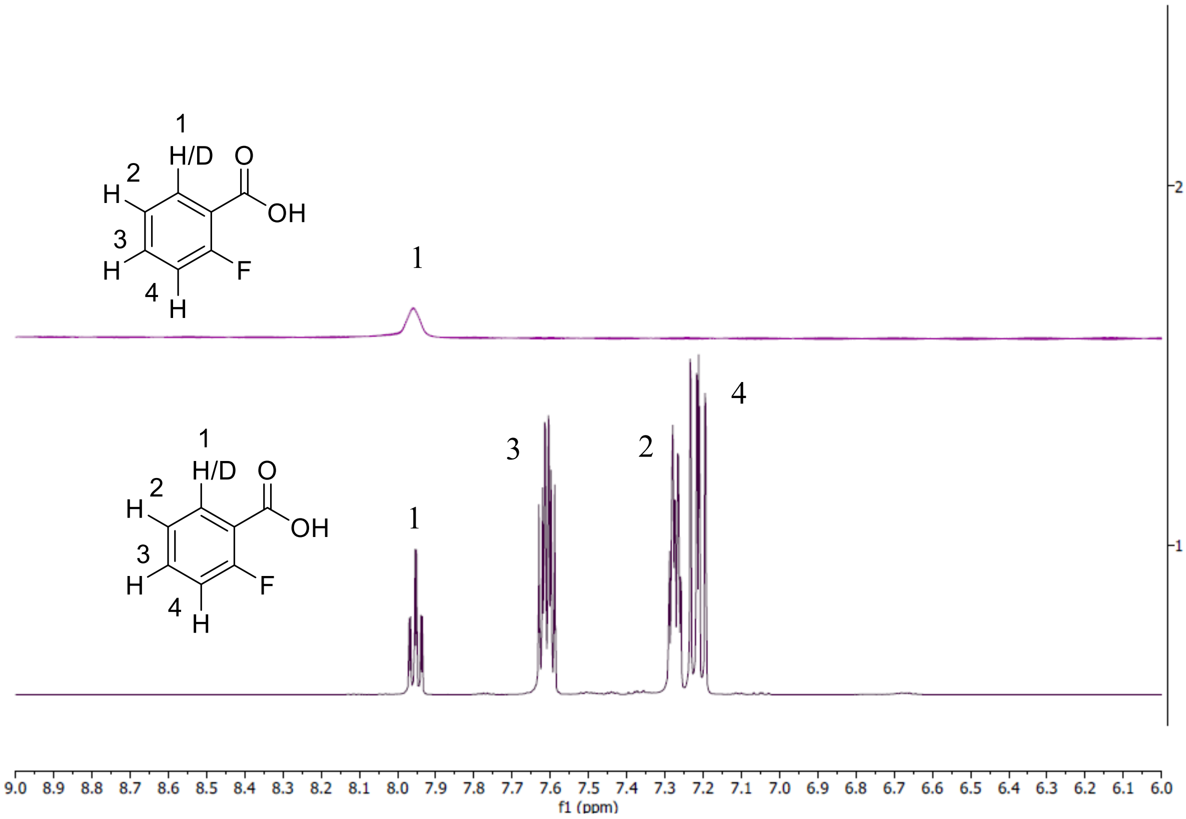

Entry 1b (Table S8)

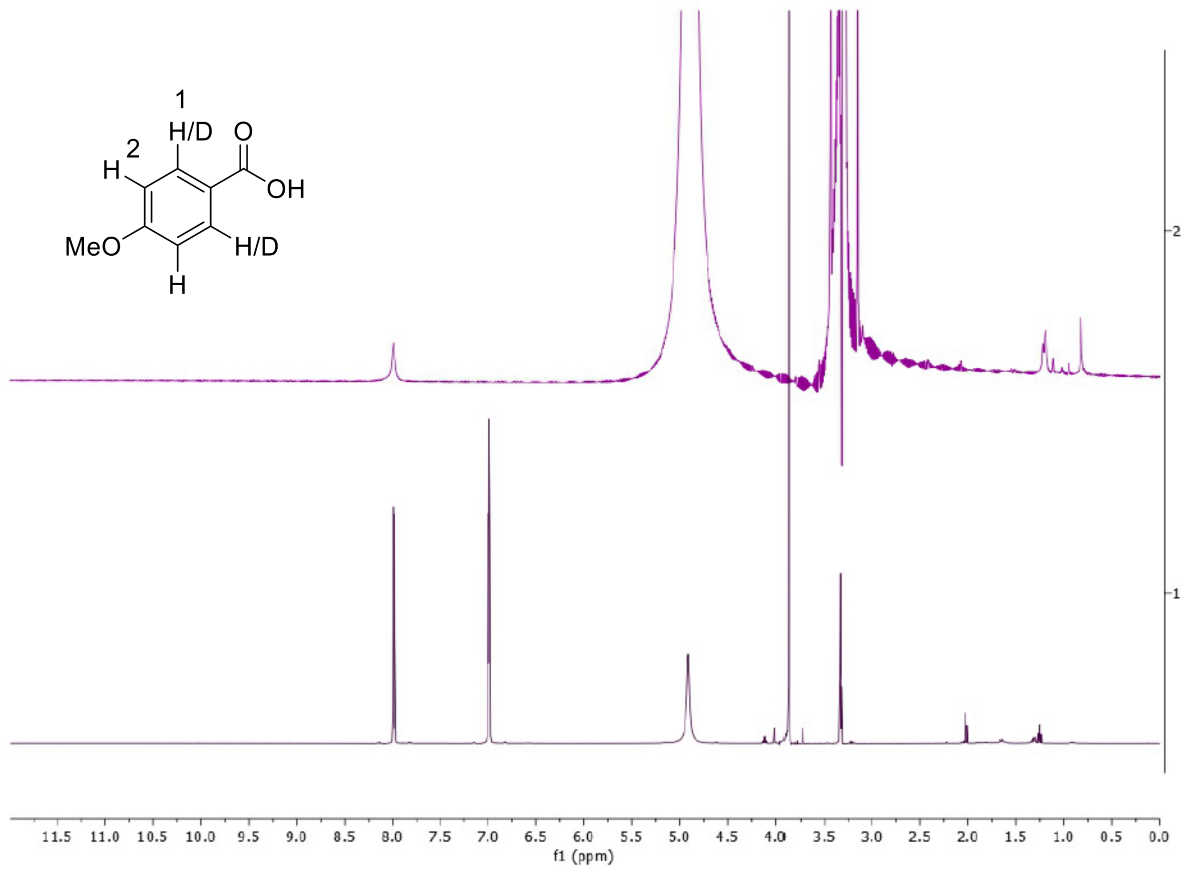

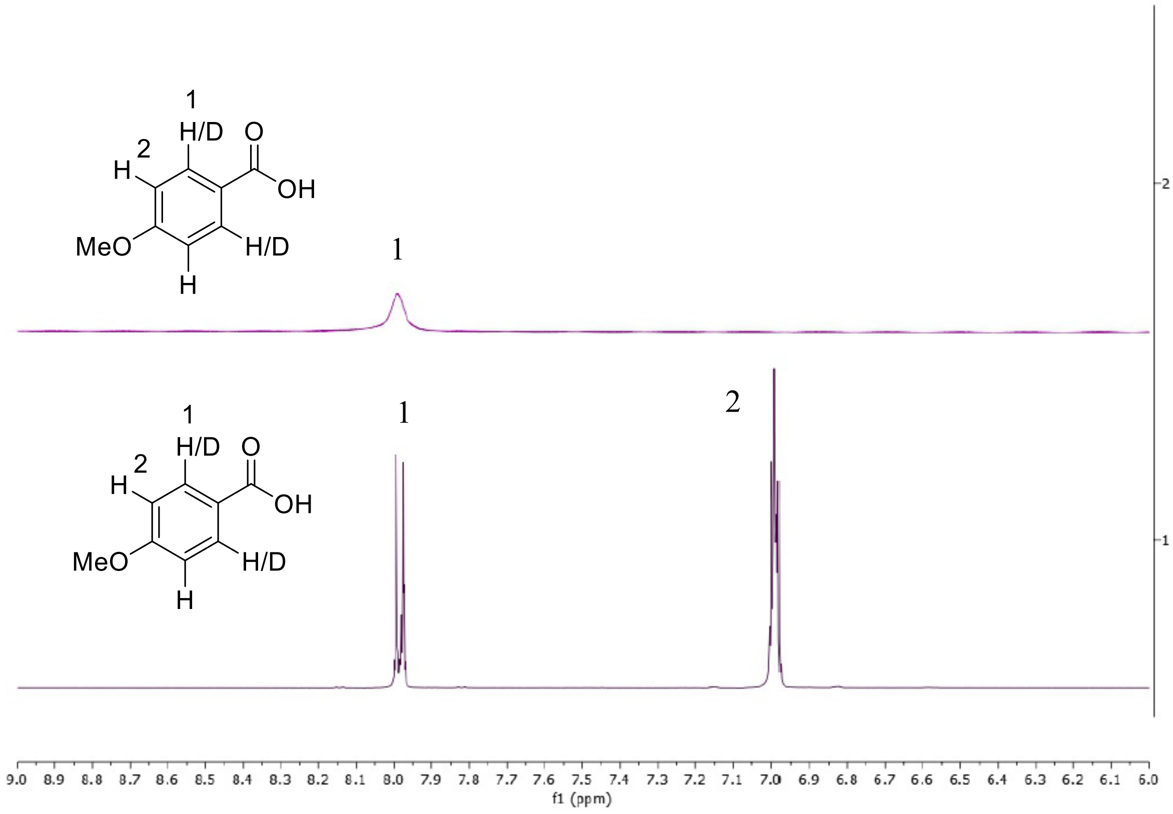

Deuterium incorporation under standard conditions (S26)

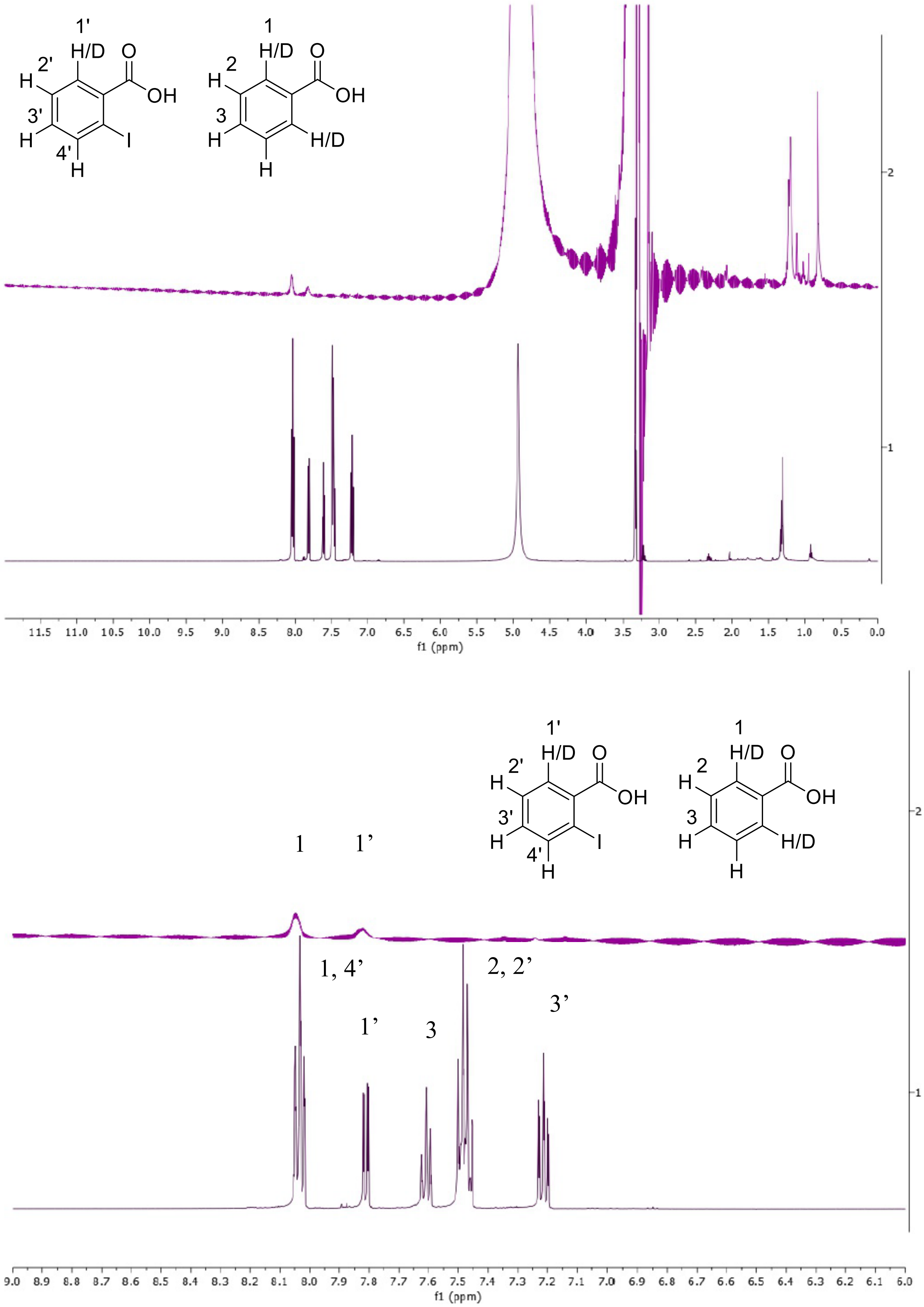

Figure S2. Deuterium incorporation site selectivity studies. The site selectivity was established by comparison of ^{2}H NMR (upper) and ^{1}H NMR spectra (lower).

Ag^I effect on C–H activation of 2a

Table S9. Ag^I effect on deuterium incorporation in **2a**

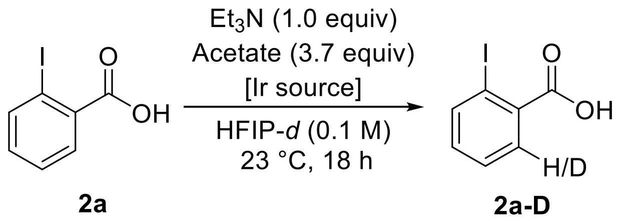

Entry	Precatalyst	Acetate source	D incorporated
**1**	$[Cp^*IrCl_2]_2$	AgOAc	6%
**2**	$[Cp^*Ir(H_2O)_3]SO_4$	AgOAc	5%
**3**	$[Cp^*IrCl_2]_2$	KOAc	21%
**4**	$[Cp^*Ir(H_2O)_3]SO_4$	KOAc	48%

2a (24.8 mg, 0.1 mmol) was dissolved in 1,1,1,3,3,3-hexafluoro-2-propanol-OD (HFIP-*d*, 1.0 mL, 0.1 M), followed by addition of Et_3N (13.9 µL, 0.1 mmol). In a separate amber vial $[Cp^*IrCl_2]_2$ (1.2 mg, 1.5 mol%) or $[Cp^*Ir(H_2O)_3]SO_4$ (1.5 mg, 3 mol%) was added. Acetate source, KOAc (36.3 mg, 0.37 mmol), or AgOAc (61.8 mg, 0.37 mmol) was added. The substrate solution was added to this mixture and stirred vigorously at room temperature (23 °C) for 18 hours. The mixture was then partitioned between aqueous HCl (1.0 M, 8 mL) and EtOAc (10 mL). The aqueous phase was extracted with EtOAc (4 x 10 mL). The combined organic phases were washed with brine (10 mL), dried over $MgSO_4$ and concentrated under reduced pressure. The residue was dissolved in DCM and applied on a silica plug (2.0 g). The plug was washed with heptane/EtOAc (1:1, 2% AcOH, 30 mL). The organic phase was concentrated under reduced pressure. The residue was dissolved in CD_3OD (3 mL). Deuterium incorporation to the *ortho* position was established by ^1H NMR (Table S9).

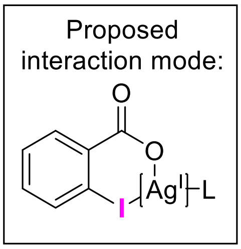

Significant increase in deuterium incorporation was observed with KOAc as the acetate source compared to AgOAc. We propose the formation of an Ag^I complex of **2a**, which supresses further C-H activation and thus protodemetalation and deuterium incorporation.

Iridacycle Ic-3 as precatalyst

The iridacycle **Ic-3** was prepared according to published procedure.[2]

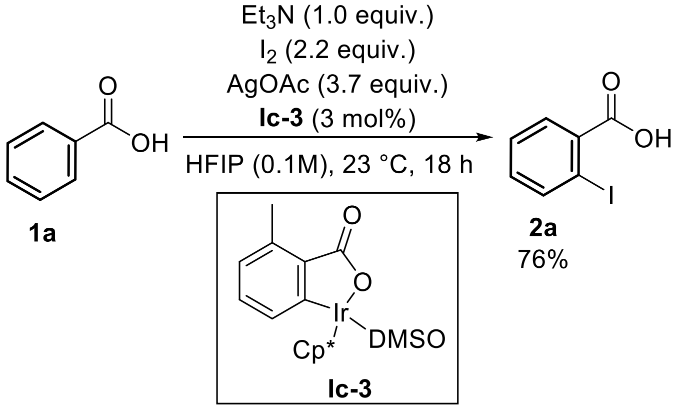

Benzoic acid (**1a**, 61.1 mg, 0.5 mmol) was dissolved in 1,1,1,3,3,3-hexafluoro-2-propanol (HFIP, 5.0 mL, 0.1 M), followed by addition of Et_3N (69.7 µL, 0.5 mmol). In a separate vial **Ic-3** (8.1 mg, 3 mol%) and AgOAc (309 mg, 1.85 mmol) were added. The benzoic acid (**1a**) solution was added, followed by addition of I_2 (279 mg, 1.1 mmol), and the reaction vessel covered with aluminum foil and the mixture was stirred vigorously at room temperature (23 °C) for 18 hours. After this Na_2SO_3 (600 mg) and water (5 mL) were added and the mixture shaked. The mixture was then partitioned between aqueous HCl (1.0 M, 50 mL) and EtOAc (40 mL). The aqueous phase was extracted with EtOAc (4 x 40 mL). The combined organic phases were washed with brine (50 mL), dried over $MgSO_4$ and concentrated under reduced pressure. The residue was dissolved in CH_2Cl_2 and applied on a silica plug (10 g). The plug was washed with heptane/EtOAc (1:1, 2% AcOH, 100 mL). The organic phase was concentrated under reduced pressure. The residue was redisolved in CD_3OD (3 mL) and 1,1,2,2-tetrachloroethane (52.8 µL, 0.5 mmol) was added as internal standard. NMR yield 76%. 2-toluic acid generated from **Ic-3** observed by NMR.

C–I bond cleavage experiments

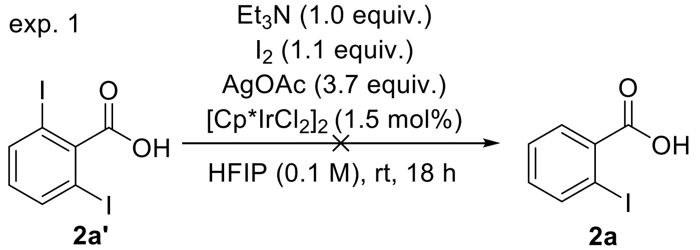

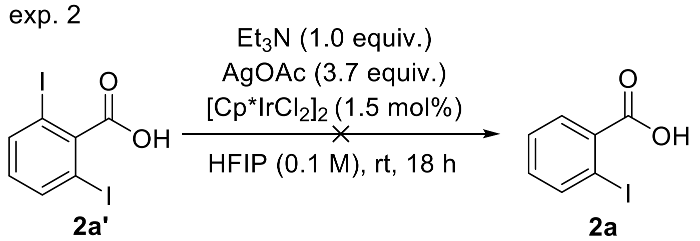

2,6-diiodobenzoic acid (**2a'**, 37.4 mg, 0.1 mmol) was dissolved in 1,1,1,3,3,3-hexafluoro-2-propanol-OD (HFIP, 1.0 mL, 0.1 M), followed by addition of Et_3N (13.9 µL, 0.1 mmol). In a separate amber vial $[Cp^*IrCl_2]_2$ (1.2 mg, 1.5 mol%) and AgOAc (61.8 mg, 0.37 mmol) were added. The substrate solution was added to this mixture. To the e1 mixture I_2 (27.9 mg, 0.11 mmol) was also added. The reactions were stirred vigorously at room temperature (23 °C) for 18 hours. SFC-MS analysis of the reaction mixtures revealed no C-I bond cleavage and formation of **2a**, which was further confirmed by ^{1}H NMR.

KIE experiments

E1: Benzoic acid (1a, 1a-D5) as model system

Two parallel reactions, one with benzoic acid (**1a**, 30.5 mg, 0.25 mmol) and one with benzoic acid-d5 (**1a-D5**, 31.8 mg, 0.25 mmol) were carried out.

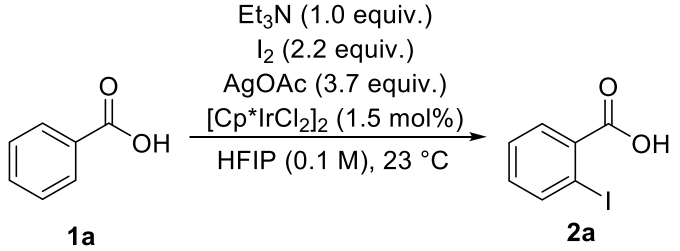

Benzoic acid (**1a**, 30.5 mg, 0.25 mmol) was dissolved in 1,1,1,3,3,3-hexafluoro-2-propanol (HFIP, 2.5 mL, 0.1 M), followed by addition of Et_3N (34.8 µL, 0.25 mmol). In a separate vial $[Cp^*IrCl_2]_2$ (3.0 mg, 1.5 mol%) and AgOAc (154.4 mg, 0.925 mmol) were added. The substrate (**1a**) solution was added to this mixture, followed by addition of I_2 (139.6 mg, 0.55 mmol), and stirred vigorously at room temperature (23 °C). Aliquots of 20 µL were taken from the reaction mixtures every 30 seconds for 2 minutes, added to MeCN/MeOH (1:4, 470 µL) and filtered. The samples were analysed by SFC-MS. Conversion was monitored by UV detection as percentage of product formed. Experiments were carried out in triplicates. Adjusted to relative molar absorptivities between **1a** and **2a**. Results shown in Figure S3.

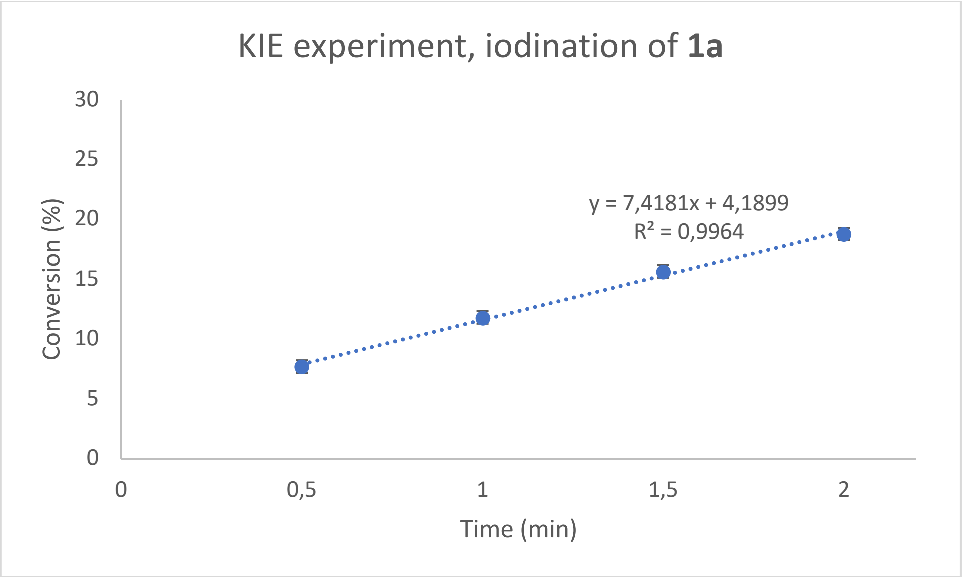

Figure S3. Iodination of **1a**, initial rate kinetic profile

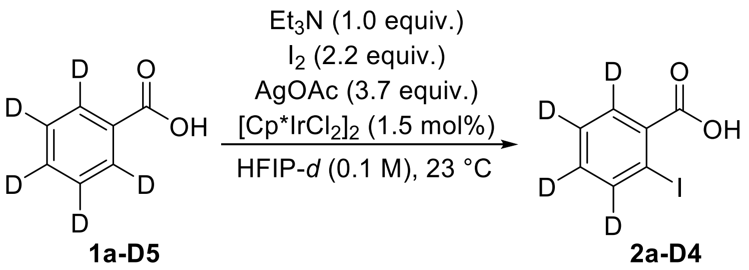

Benzoic acid-d5 (**1a-D5**, 31.8 mg, 0.25 mmol) was dissolved in 1,1,1,3,3,3-hexafluoro-2- propanol-OD (HFIP-*d*, 2.5 mL, 0.1 M), followed by addition of Et_3N (34.8 µL, 0.25 mmol). In a separate vial $[Cp^*IrCl_2]_2$ (3.0 mg, 1.5 mol%) and AgOAc (154.4 mg, 0.925 mmol) were added. The substrate (**1a-D5**) solution was added to this mixture, followed by addition of I_2 (139.6 mg, 0.55 mmol), and stirred vigorously at room temperature (23 °C). Aliquots of 20 µL were taken from the reaction mixtures every minute for 2.5 minutes, added to MeCN/MeOH (1:4, 470 µL) and filtered. The samples were analysed by SFC-MS. Conversion was monitored by UV detection as percentage of product formed. Experiments were carried out in triplicates. Adjusted to relative molar absorptivities between **1a** and **2a**. Results shown in Figure S4.

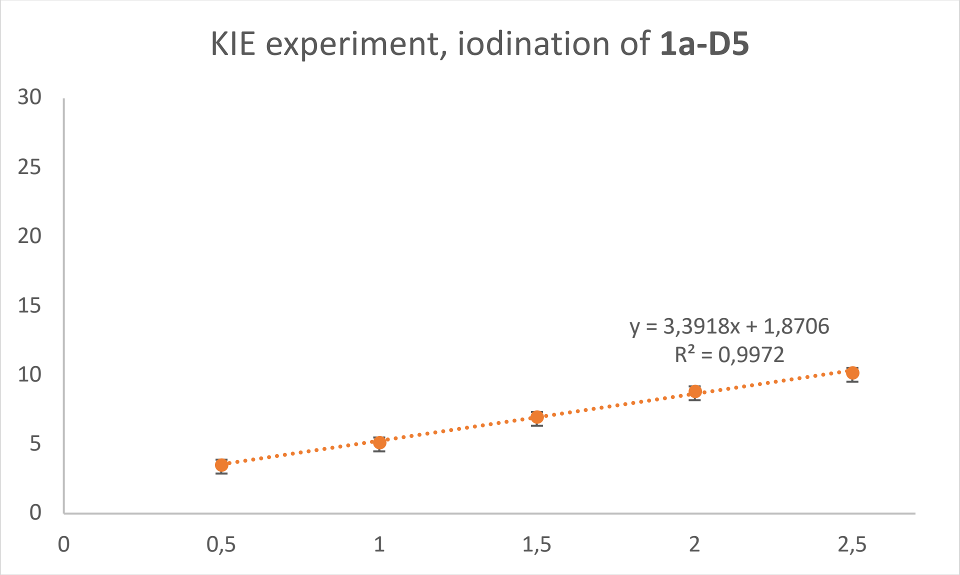

Figure S4. Iodination of **1a-D5**, initial rate kinetic profile

The measured k_H/k_D = 2.19. Based on the k_H/k_D value, together with the observation of deuterium incorporation for this substrate from previous experiments (Table S8) C-H bond cleavage to take part in the turnover-limiting step together with the subsequent oxidative addition step for this substrate.

E2: 4-(trifluoromethyl) benzoic acid (1l, 1l-D2) as model system

Two parallel reactions, one with 4-(trifluoromethyl) benzoic acid (**1l**, 47.5 mg, 0.25 mmol) and one with 4-(trifluoromethyl) benzoic acid-d2 (**1l-D2**, 48.0 mg, 0.25 mmol) were carried out.

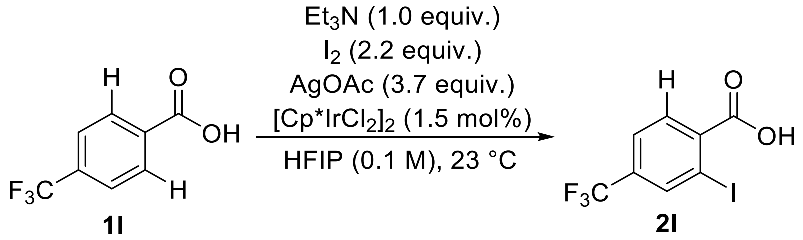

4-(trifluoromethyl) benzoic acid (**1l**, 47.5 mg, 0.25 mmol) was dissolved in 1,1,1,3,3,3-hexafluoro-2-propanol (HFIP, 2.5 mL, 0.1 M), followed by addition of Et_3N (34.8 µL, 0.25 mmol). In a separate vial $[Cp^*IrCl_2]_2$ (3.0 mg, 1.5 mol%) and AgOAc (154.4 mg, 0.925 mmol) were added. The substrate (**1l**) solution was added to this mixture, followed by addition of I_2 (139.6 mg, 0.55 mmol), and stirred vigorously at room temperature (23 °C). Aliquots of 20 µL were taken from the reaction mixtures 30 seconds for 2.5 minutes, added to MeCN/MeOH (1:4, 470 µL) and filtered. The samples were analysed by SFC-MS. Conversion was monitored by UV detection as percentage of product formed. Experiments were carried out in triplicates. Adjusted to relative molar absorptivities between **1l** and **2l**. Results shown in Figure S5.

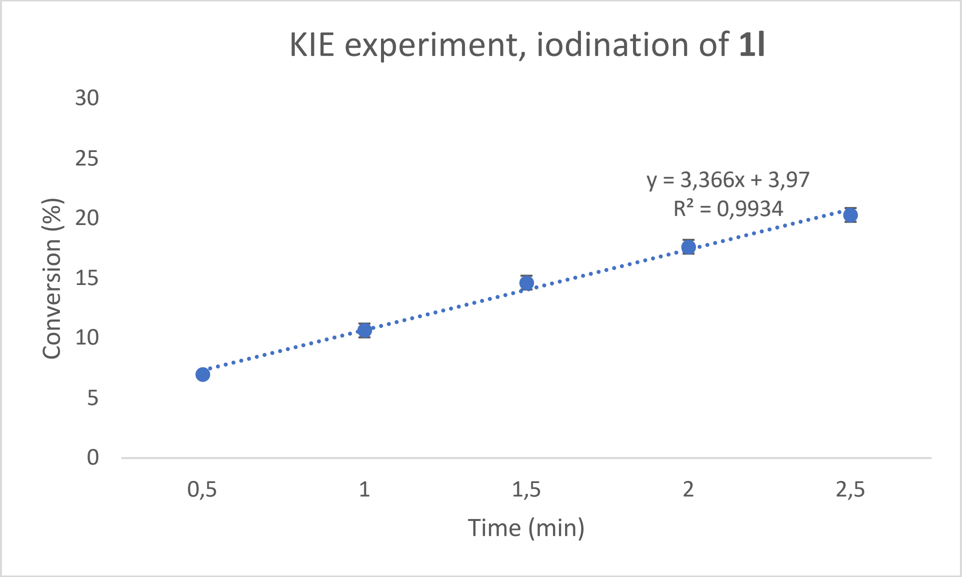

Figure S5. Iodination of **1l**, initial rate kinetic profile

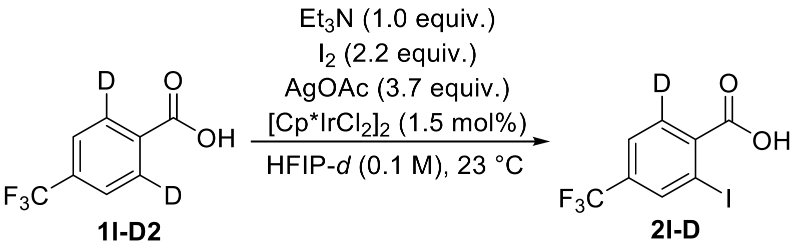

4-(trifluoromethyl) benzoic acid-d2 (**1l-D2**, 48.0mg, 0.25 mmol) was dissolved in 1,1,1,3,3,3-hexafluoro-2- propanol-OD (HFIP-*d*, 2.5 mL, 0.1 M), followed by addition of Et_3N (34.8 µL, 0.25 mmol). In a separate vial $[Cp^*IrCl_2]_2$ (3.0 mg, 1.5 mol%) and AgOAc (154.4 mg, 0.925 mmol) were added. The substrate (**1l-D2**) solution was added to this mixture, followed by addition of I_2 (139.6 mg, 0.55 mmol), and stirred vigorously at room temperature (23 °C). Aliquots of 20 µL were taken from the reaction mixtures every minute for 5 minutes, added to MeCN/MeOH (1:4, 470 µL) and filtered. The samples were analysed by SFC-MS. Conversion was monitored by UV detection as percentage of product formed. Experiments were carried out in triplicates. Adjusted to relative molar absorptivities between **1l** and **2l**. Results shown in Figure S6.

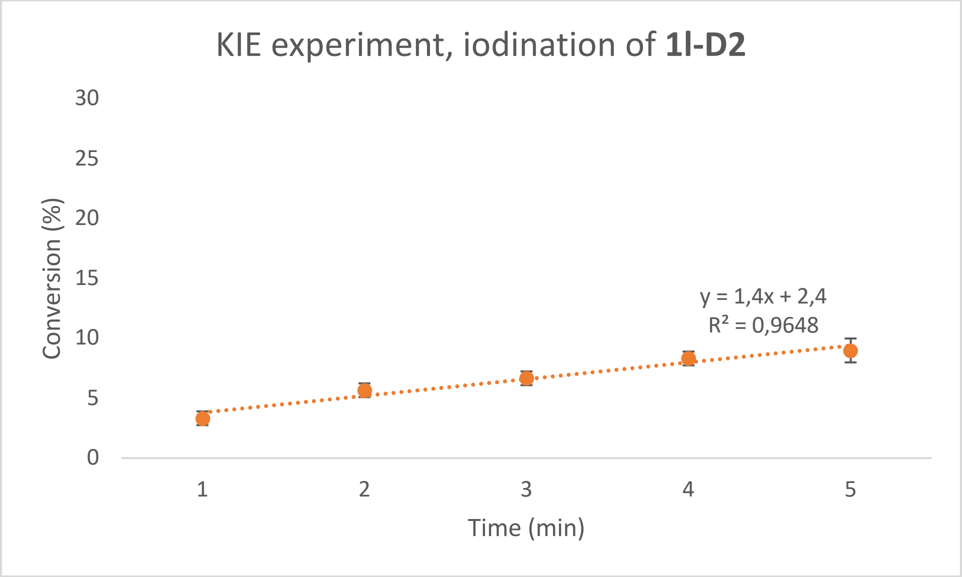

Figure S6. Iodination of **1l-D2**, initial rate kinetic profile

The measured k_H/k_D = 4.95. Based on the observed KIE we propose the C-H bond cleavage to be the turnover-limiting step for this substrate.

Hammett plot studies

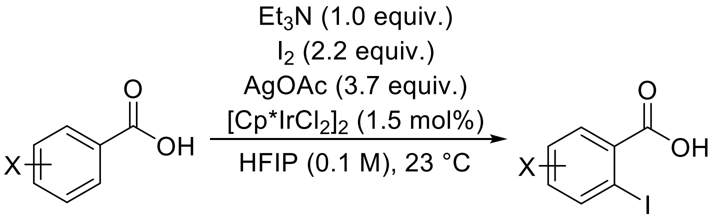

Substrate (0.25 mmol) was dissolved in 1,1,1,3,3,3-hexafluoro-2-propanol (HFIP, 2.5 mL, 0.1 M), followed by addition of Et_3N (34.8 µL, 0.25 mmol). In an amber vial $[Cp^*IrCl_2]_2$ (3.0 mg, 1.5 mol%) and AgOAc (154.4 mg, 0.925 mmol) were added. The substrate solution was added to this mixture, followed by addition of I_2 (139.6 mg, 0.55 mmol), and stirred vigorously at room temperature (23 °C). Aliquots of 20 µL were taken from the reaction mixtures every 30 seconds for 5 minutes, added to MeCN/MeOH (1:4, 470 µL) and filtered. The samples were analysed by SFC-MS. Conversion was monitored by UV detection as percentage of product formed. Experiments were carried out in triplicates. For the 3-substituted substrates the results are shown in Table S10, plotted in Figure S7. For the 4-substituted substrates the results are shown in Table S11, plotted in Figure S8.

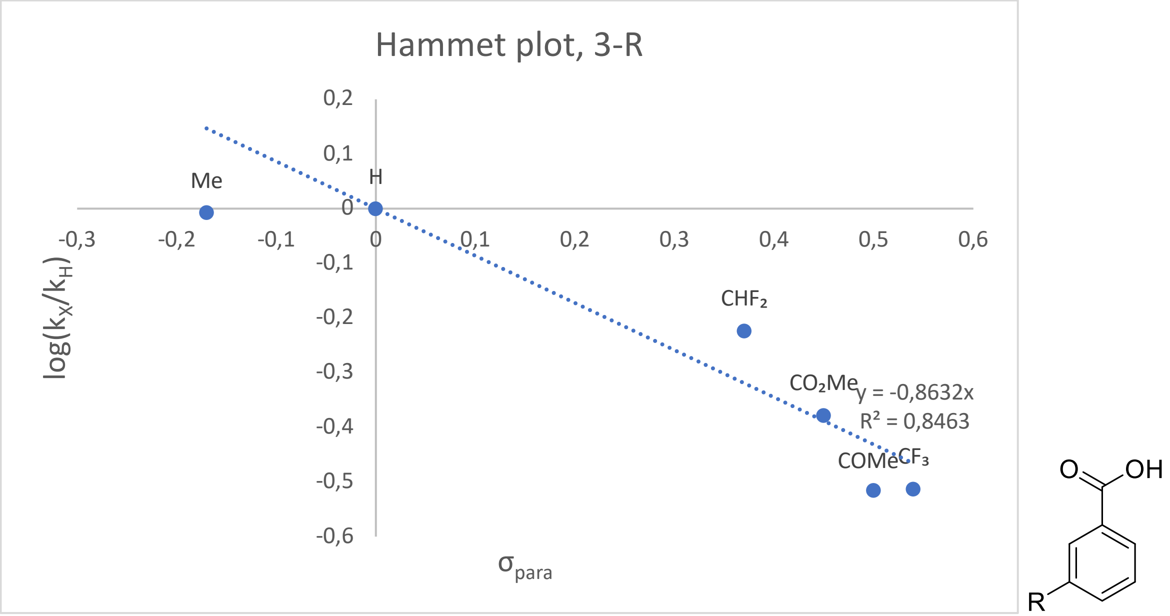

Figure S7. Hammett plot study, iodination of *meta*-substituted substrates

Table S10. Hammett plot *meta*-substituent data

R	k_{rel} (slope)	log (kX/KH)	σ_{para}
Me	7.29	-0.00743	-0.17
H	7.42	0	0
CHF2	4.43	-0.22346	0.37
CF3	2.28	-0.51262	0.54
CO2Me	3.11	-0.37761	0.45
COMe	2.27	-0.51459	0.50

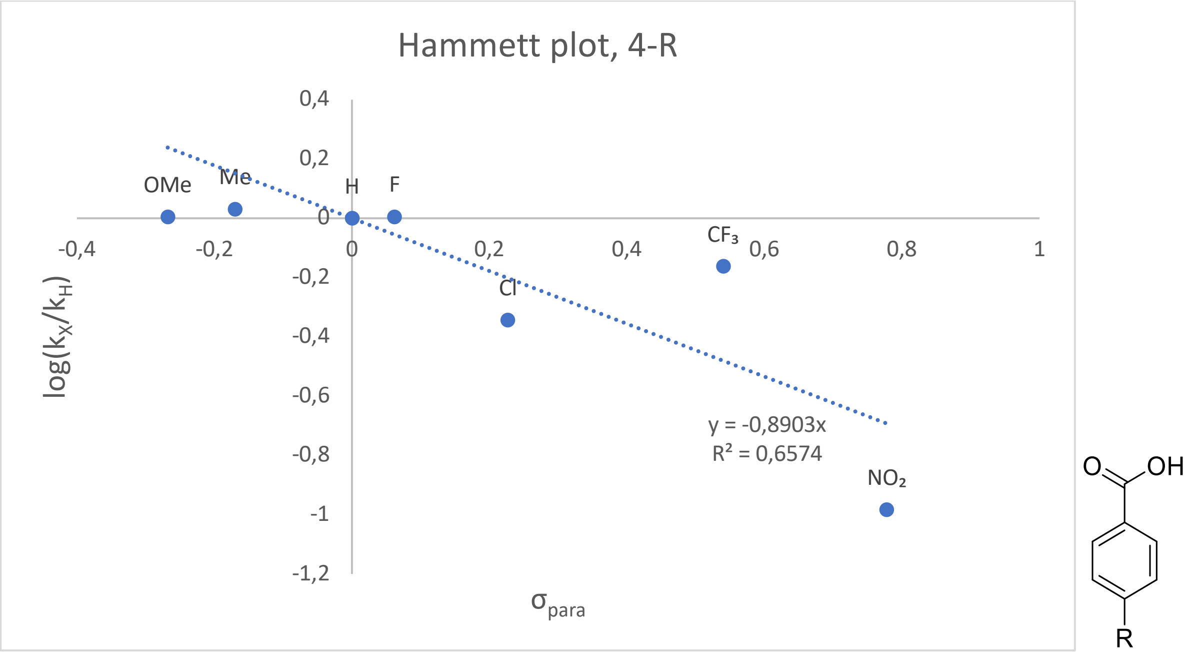

Figure S8. Hammett plot study, iodination of *para*-substituted substrates

Table S11. Hammett plot *para*-substituent data

4-R	k_{rel} (slope)	logkX/kH	σ_{para}

MeO	7.49951	0.004746	-0.268
Me	7.969	0.031117	-0.17
H	7.418	0	0
Cl	3.365	-0.3433	0.227
NO2	0.769785	-0.98392	0.778
F	7.5158	0.005688	0.062
CF3	5.1005	-0.16267	0.54

Conclusion: Due to the C-H activation step being reversible by a protodemetalation depending on the substrate electronics (Table S8), the respective entries in the Hammett plots do not give a simple trend due to varying characters of the turnover-limiting steps.

Resting state studies

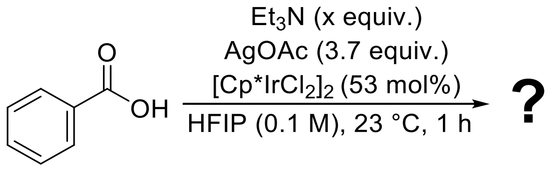

1a

Experiment	Et_3N equiv.
e1	0
e2	1
e3	4

Benzoic acid (**1a**, 12.2 mg, 0.1 mmol) was dissolved in 1,1,1,3,3,3-hexafluoro-2-propanol (HFIP, 1 mL, 0.1 M), followed by addition of Et_3N (x equiv.). To an amber vial $[Cp^*IrCl_2]_2$ (42.4 mg, 53 mol%) and AgOAc (61.8 mg, 0.37 mmol) were added. The substrate (**1a**) solution was added to this mixture and stirred vigorously at room temperature (23 °C) for 1 hour. After this an aliquot (200 µL) was taken from the reaction mixture and added to C_6D_6 (0.6 mL) in an NMR tube. ^{1}H, ^{13}C and HMBC NMR experiments were carried out to identify the potential resting state. Benzoic acid and the corresponding $Et_3NH^+BzO^-$ salt were used as standards for comparison.
Experiments 1 and 2 showed no major change compared to the benzoic acid Et_3N salt in ^{1}H NMR.
In an HMBC NMR experiment the disappearance of the carboxylate carbon cross-peak was observed in experiment 2 (Table S12, Entry 3, visualised in Figure S10). The cross-peak was detected when HMBC was recorded at 50 °C (Entry 4, visualised in Figure S10). Furthermore, the NMR shift differed from both standards (Entries 1 and 2), indicating an interaction of the benzoate. Results shown in Table S12, spectral overlaps in Figure S9.

Table S12. Resting state study, reagent composition and ^{13}C NMR study

Entry	Composition	13C shift carboxylate	Note
1	BzOH, HFIP	170.4	
2	BzOH, HFIP, Et_3N (1 equiv.)	176.5	
3	Reaction mixture (e2)	Missing (broad)	Not detected by HMBC
4	Reaction mixture (e2)	173.9	HMBC, 50 °C

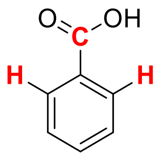

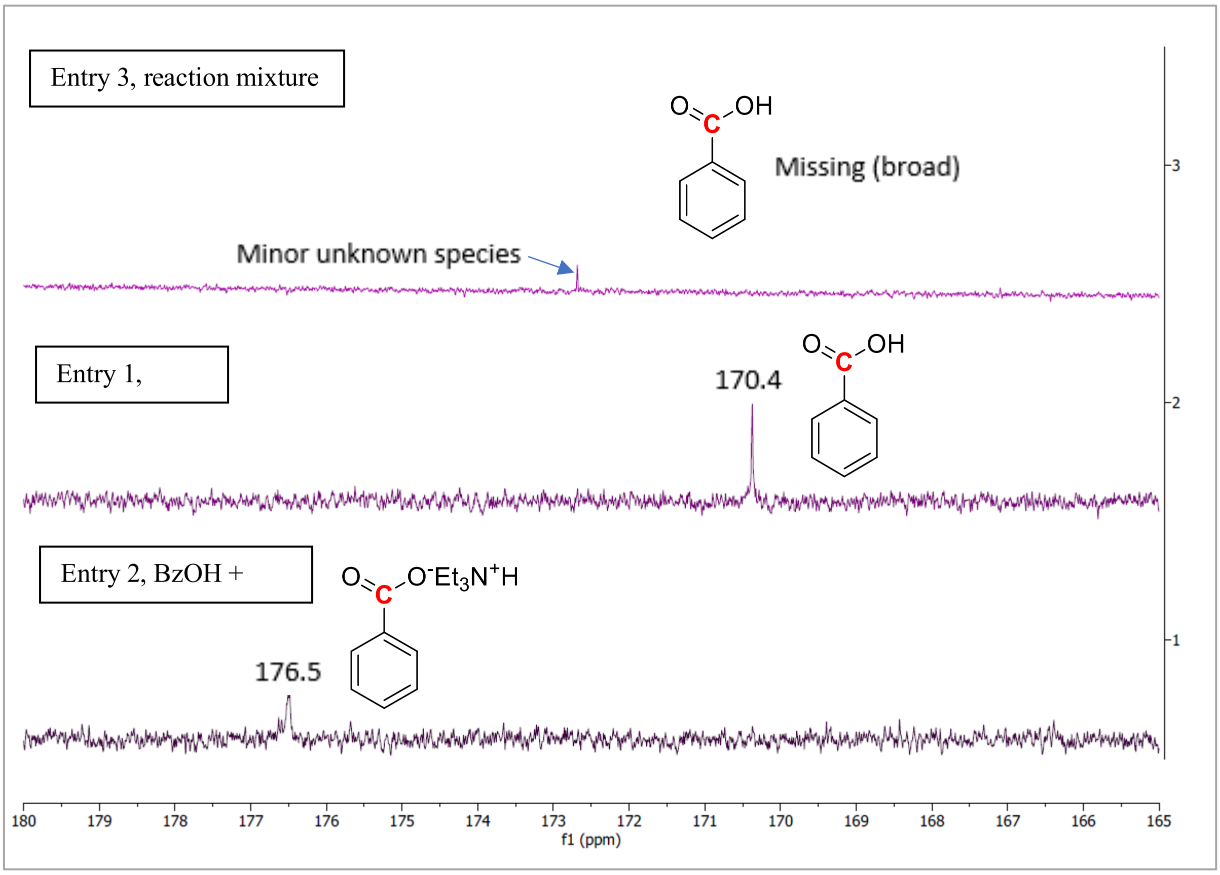

Figure S9. Changes in ^{13}C shifts of the carbonyl carbon.

Entry 3, HMBC

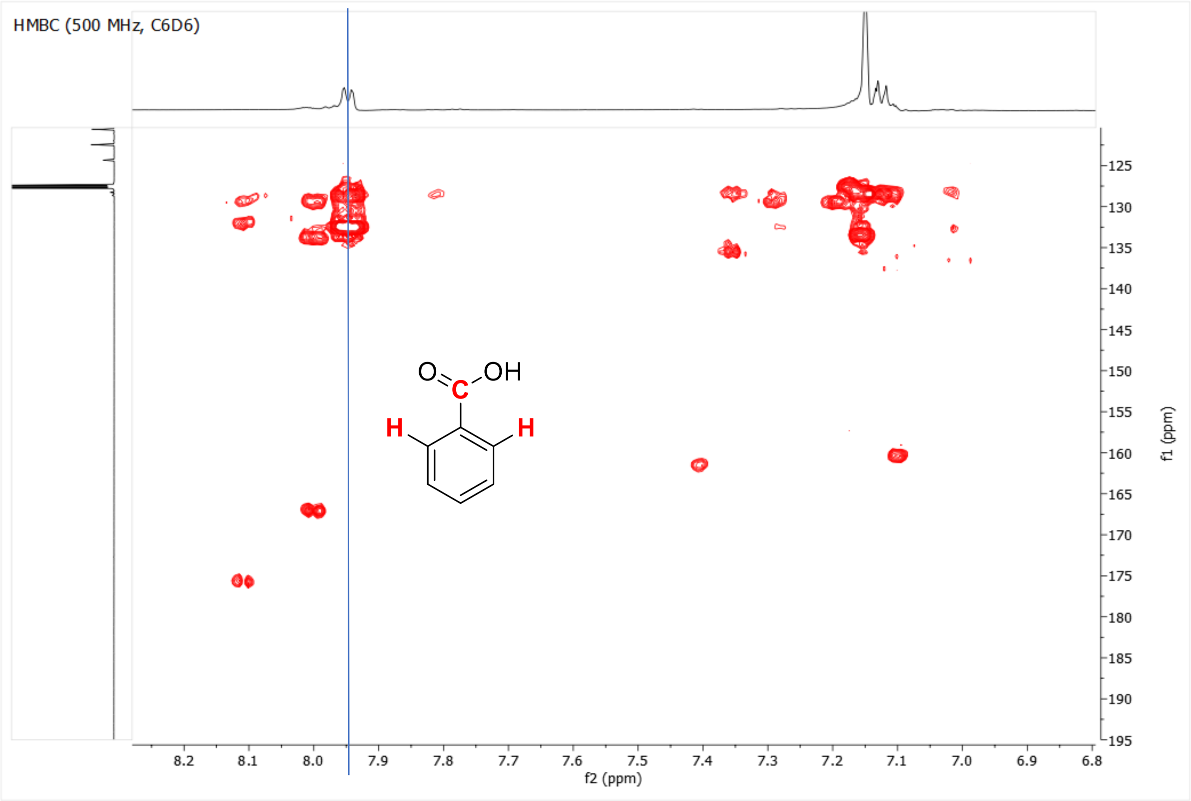

Entry 4, HMBC

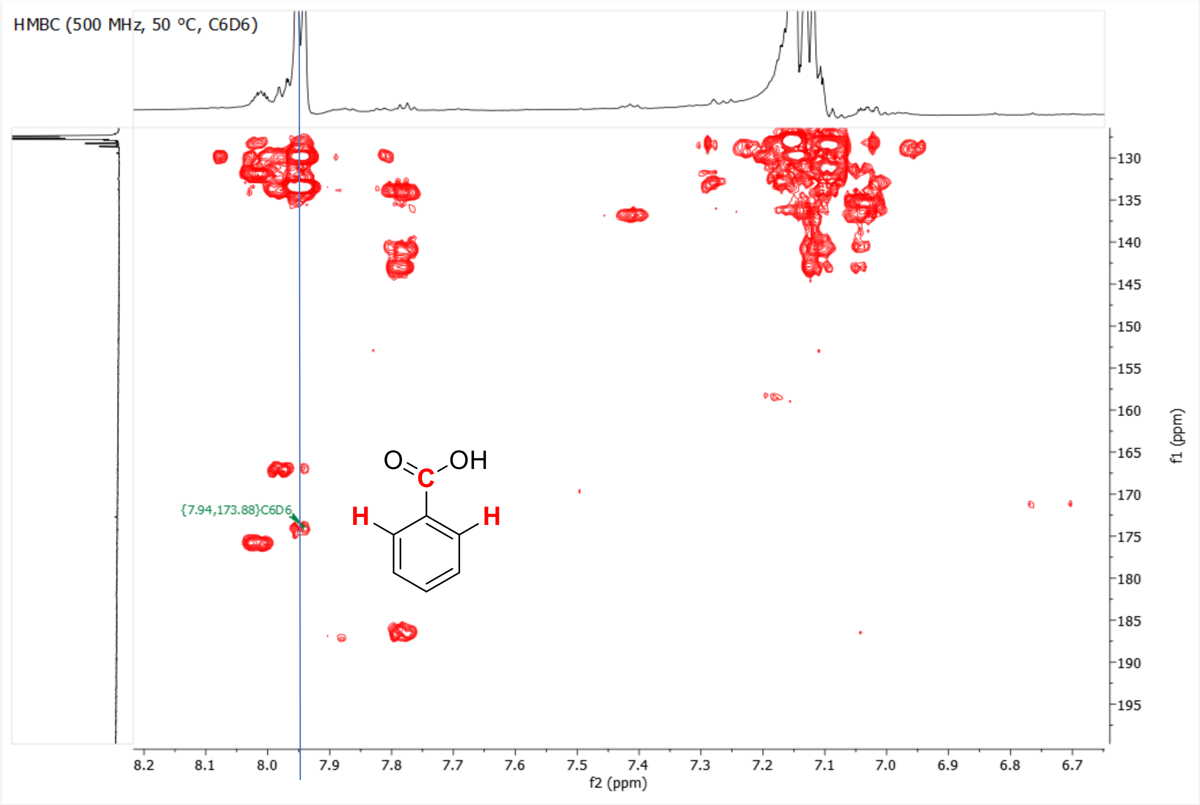

Figure S10. HMBC experiment of the reaction mixture e2. Up: Entry 3 – no cross-peak. Entry 4 – cross-peak observed at elevated temperature.

In a competition experiment with equimolar 2-iodobenzoic acid **2a** only the disappearance of the benzoic acid **1a** carboxylate cross-peak was observed (Figure S11).

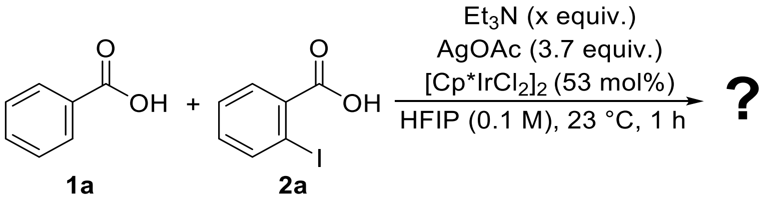

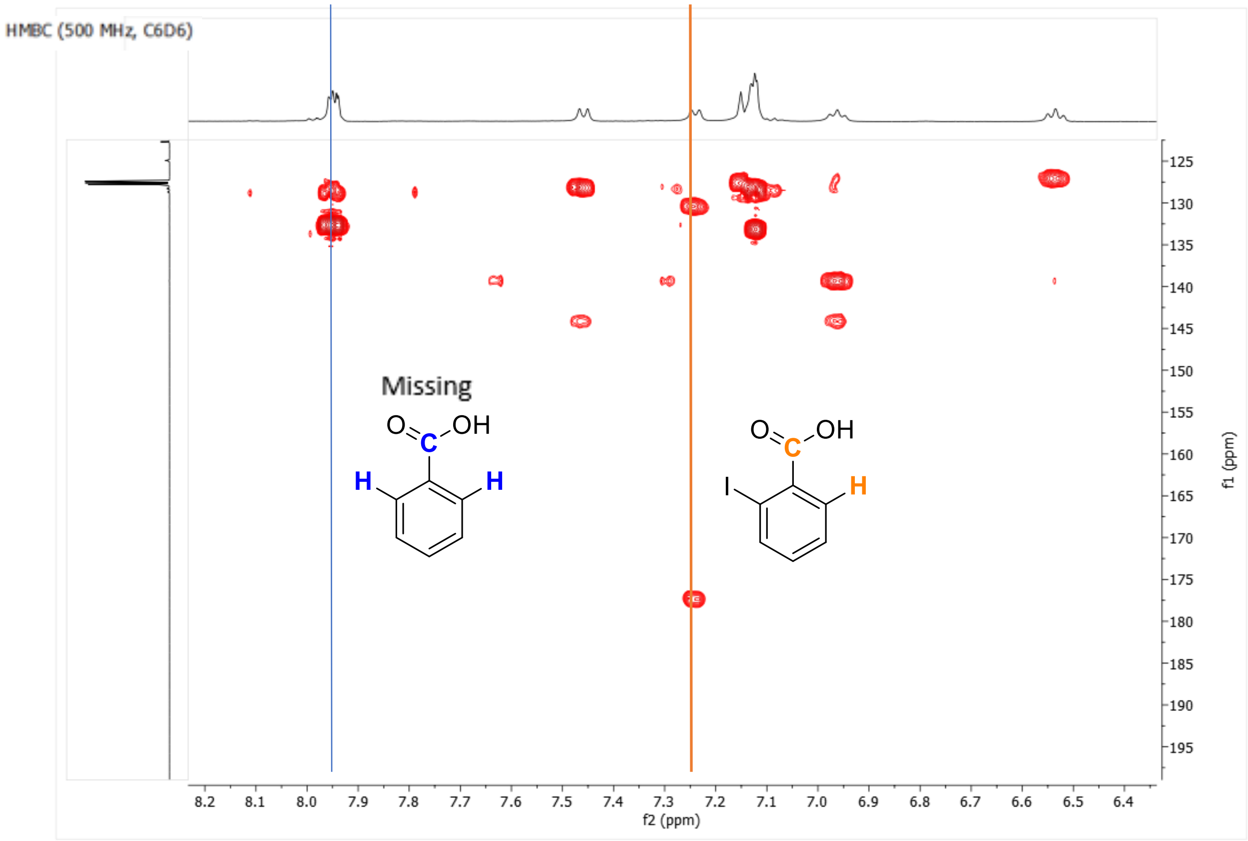

Figure S11. Resting state competition experiment. **2a** cross-peak remains visible, while **1a** cross-peak is missing, suggesting incorporation in the resting state

In combination, these point to a resting state with benzoic acid bound complex such as **3a**, where L is possibly an oxygen from a μ_2 bound acetate as reported by Ison.[[14]]

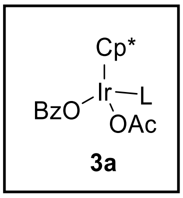

Iridacyle Ic-2

Experiment 3 (e3) showed a 2-substituted benzoic acid as the major species in ^{1}H NMR. The species was isolated by preparative SFC and identified as iridacycle **Ic-2**. Identity was confirmed by SFC-MS, ^{1}H, ^{13}C, ^{19}F, HSQC, HMBC, NOESY NMR experiments and HRMS.

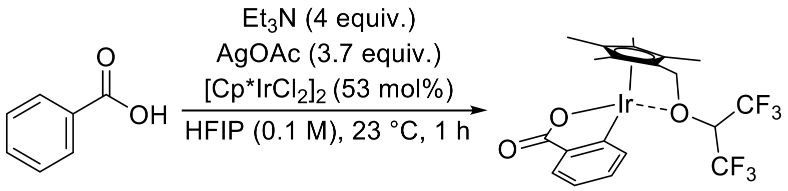

1a **Ic-2**

Ic-1 was subsequently used as a catalyst for the iodination reaction. No conversion was observed.

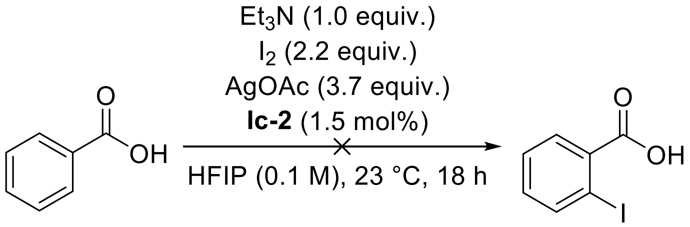

1a **2a**

Ortho substituent steric effect

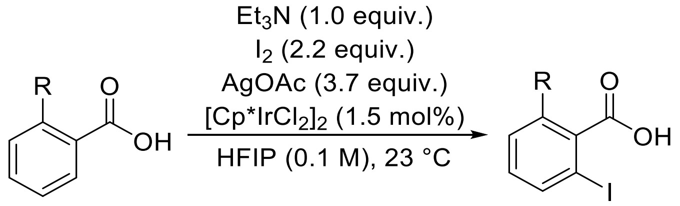

Substrate (0.1 mmol) was dissolved in 1,1,1,3,3,3-hexafluoro-2-propanol (HFIP, 1.0 mL, 0.1 M), followed by addition of Et_3N (13.9 µL, 0.1 mmol). In an amber vial $[Cp^*IrCl_2]_2$ (1.2 mg, 1.5 mol%) and AgOAc (61.8 mg, 0.37 mmol) were added. The substrate solution was added to this mixture, followed by I_2 (55.8 mg, 0.22 mmol), and stirred vigorously at room temperature (23 °C). Aliquots of 20 µL were taken from the reaction mixtures every 30 seconds for 5 minutes, added to MeCN/MeOH (1:4, 470 µL) and filtered. The samples were analysed by SFC-MS. Conversion was monitored by UV detection as percentage of product formed. Experiments were carried out in triplicates.

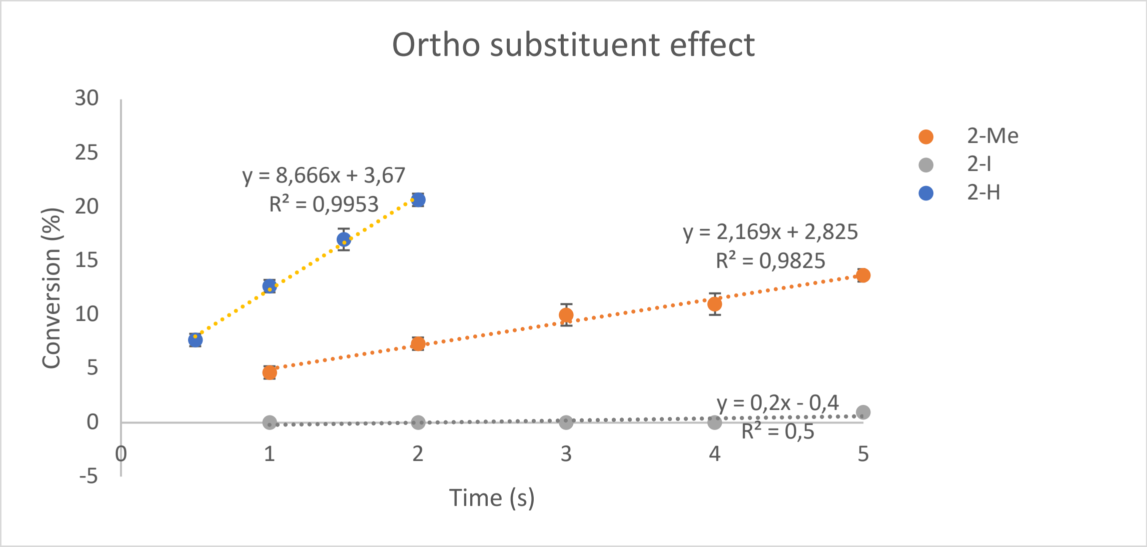

Figure S12. *Ortho* substituent effect on iodination rate

From the obtained results an *ortho*-substituent steric effect was apparent, reaction rates decreasing as substituent size was increased (Figure S12). A ratio k_H/k_{Me} = 4.00 was measured.

Comparison with NIS methodology

The monoiodination methodology described in this paper was compared to a previously published methodology.[[11]]

2-iodobenzoic acid (**2a**) was used as the model system in a reaction kinetics study.

Experiment 1 (NIS):

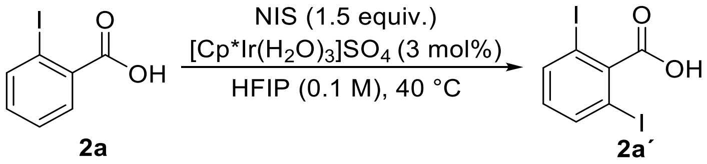

2-iodobenzoic acid (**2a**, 124.0 mg, 0.5 mmol), *N*-iodosuccinimide (168.7 mg, 0.75 mmol) and [Cp*Ir(H_2O)$_3$]SO_4 (6.9 mg, 3 mol%) were added to a vial. In a separate vial 1,1,1,3,3,3-hexafluoro-2-propanol (HFIP, 5.0 mL) was heated to 40 °C. The hot HFIP was added to the vial with the reagents and the mixture was stirred vigorously at 40 °C. Aliquots of 20 µL were taken from the reaction mixtures every minute for 10 minutes, added to MeCN/MeOH (1:4, 470 µL) and filtered. The samples were analyzed by SFC-MS. Conversion was monitored by UV detection as percentage of product formed.

Experiment 2 (I2):

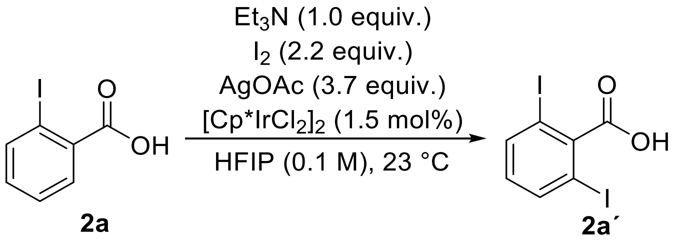

2-iodobenzoic acid (**2a**, 124.0 mg, 0.5 mmol) was dissolved in 1,1,1,3,3,3-hexafluoro-2-propanol (HFIP, 5.0 mL, 0.1 M). Et_3N (69.7 µL, 0.5 mmol) was added and the mixture sonicated for 30 seconds. To a separate vial $[Cp^*IrCl_2]_2$ (6.0 mg, 7.5 µmol, 1.5 mol%) and AgOAc (309 mg, 1.85 mmol) were added. The substrate (**2a**) solution was added to this mixture, followed by I_2 (279 mg, 1.1 mmol), and the mixture was stirred vigorously at 23 °C. Aliquots of 20 µL were taken from the reaction mixtures every minute for 10 minutes, added to MeCN/MeOH (1:4, 470 µL) and filtered. The samples were analyzed by SFC-MS. Conversion was monitored by UV detection as percentage of product formed (Figure S13).

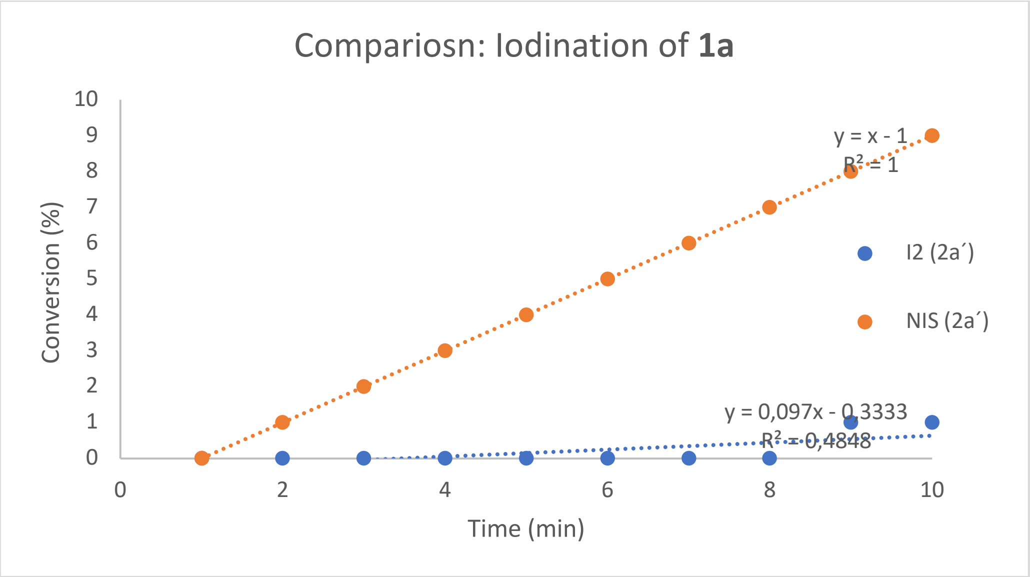

Figure S13. Comparison experiment with previously published methodology using **2a** as starting material.

Reaction kinetic profile

(2a) reaction kinetics

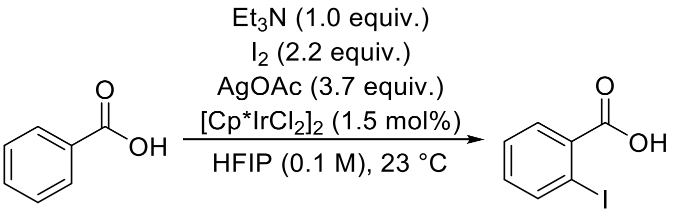

Benzoic acid (**1a**, 61.0 mg, 0.5 mmol) was dissolved in 1,1,1,3,3,3-hexafluoro-2-propanol (HFIP, 5.0 mL, 0.1 M). Et_3N (69.7 µL, 0.5 mmol) was added and the mixture sonicated for 30 seconds. To a separate vial $[Cp^*IrCl_2]_2$ (6.0 mg, 7.5 µmol, 1.5 mol%) and AgOAc (309 mg, 1.85 mmol) were added. The substrate (**2a**) solution was added to this mixture, followed by I_2 (279 mg, 1.1 mmol), and the mixture was stirred vigorously at 23 °C. Aliquots of 20 µL were taken from the reaction mixtures every minute for 10 minutes, added to MeCN/MeOH (1:4, 470 µL) and filtered. The samples were analyzed by SFC-MS. Conversion was monitored by UV detection as percentage of product formed (Table S13).

Table S13. Iodination of **1a** kinetic profile

Time (s)	**1a**	**2a**	**2a'**
20	94	6	0
50	90	10	0
80	81	19	0
110	78	22	0
140	74	26	0
200	63	36	0
260	52	48	0
320	36	63	1
620	18	81	1
920	11	88	1
1220	8	91	1
1820	7	92	1
3620	4	94	2
7220	1	95	4

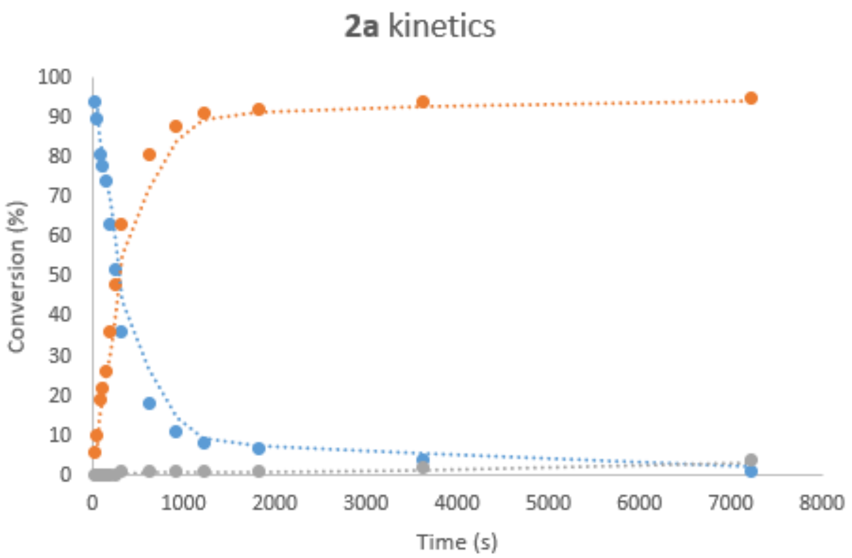

(2l) reaction kinetics

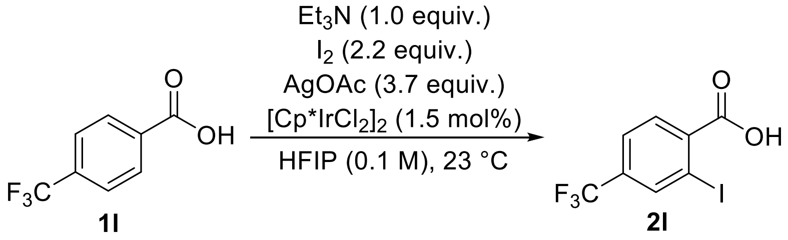

4-(trifluoromethyl) benzoic acid (**1l**, 158.0 mg, 0.5 mmol) was dissolved in 1,1,1,3,3,3-hexafluoro-2-propanol (HFIP, 5.0 mL, 0.1 M). Et_3N (69.7 µL, 0.5 mmol) was added and the mixture sonicated for 30 seconds. To a separate vial $[Cp^*IrCl_2]_2$ (6.0 mg, 7.5 µmol, 1.5 mol%) and AgOAc (309 mg, 1.85 mmol) were added. The substrate (**1l**) solution was added to this mixture, followed by I_2 (279 mg, 1.1 mmol), and the mixture was stirred vigorously at 23 °C. Aliquots of 20 µL were taken from the reaction mixtures for 5 hours, added to MeCN/MeOH (1:4, 470 µL) and filtered. The samples were analysed by SFC-MS. Conversion was monitored by UV detection as percentage of product formed (Table S14).

Table S14. Iodination of **1l** kinetic profile

Time (min)	**1l**	**2l**
0.5	95	5
1	92	8
1.5	89	11
2	88	12
2.5	84	16
3	83	17
3.5	81	19
4	80	20
4.5	78	22

5	77	23
6	75	25
7	72	28
8	69	31
9	68	32
10	67	33
20	56	44
30	51	49
60	44	56
90	40	60
150	32	68
300	23	77

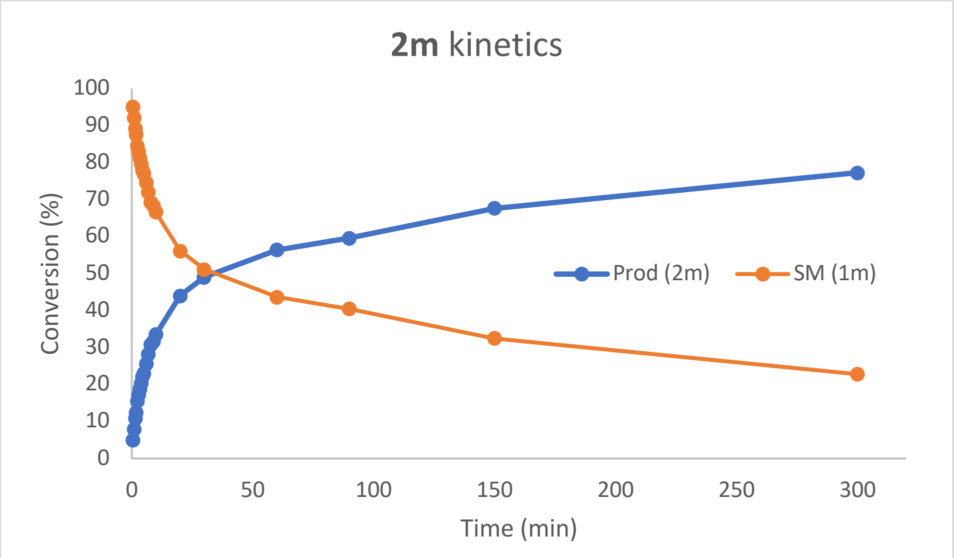

Figure S15. Iodination of **1m**, kinetic profile

References

[1] G. Kaupp, A. Herrmann, J. Schmeyers, *Angew. Chem. Int. Ed.* **2002**, *8*, 1395-1406.

[2] C. A. Panetta, S. M. Garlick, H. D. Durst, F. R. Longo, J. R. Ward, *J. Org. Chem.* **1990**, *55*, 5202-5205.

[3] J. Koizumi, S. Kobayashi, S. Uyeo, *Chem. Pharm. Bull.* **1964**, *12*, 696-705.

[4] J. Carstens, M. R. Heinrich, W. Steglich, *Tetrahedron Lett.* **2013**, *54*, 5445-5447.

[5] C. D. Gabbutt, B. M. Heron, A. C. Instone, *Tetrahedron* **2006**, *62*, 737-745.

[6] M. Hamada, K. Tashiro, H. Sakashita, M. Kiuchi, S. Takeda, K. Adachi, *EP2842937A1* **2015**.

[7] L.-Q. Cui, Z.-L. Dong, K. Liu, C. Zhang, *Org. Lett.* **2011**, *13*, 6488-6491.

[8] A. Kommreddy, M. S. Bowsher, M. R. Gunna, K. Botha, T. K. Vinod, *Tetrahedron Lett.* **2008**, *49*, 4378-4382.

[9] T. Christine, A. Tabey, T. Cornilleau, E. Fouquet, P. Hermange, *Tetrahedron* **2019**, *75*, 130765.

[10] T.-S. Mei, R. Giri, N. Maugel, J.-Q. Yu, *Angew. Chem. Int. Ed.* **2008**, *47*, 5215-5219.

[11] E. Erbing, A. Sanz-Marco, A. Vázquez-Romero, J. Malmberg, M. J. Johansson, E. Gómez-Bengoa, B. Martín-Matute, *ACS Catal.* **2018**, *8*, 920-925.

[12] F. Gohier, J. Mortier, *J. Org. Chem.* **2003**, *68*, 2030-2033.

[13] J. B. Ravnsbæk, M. F. Jacobsen, C. B. Rosen, N. V. Voigt, K. V. Gothelf, *Angew. Chem. Int. Ed.* **2011**, *50*, 10851-10854.

[14] D. A. Frasco, C. P. Lilly, P. D. Boyle, E. A. Ison, *ACS Catal.* **2013**, *3*, 2421-2429.

^{1}H NMR and ^{13}C NMR of iodobenzoic acids 2

2-iodobenzoic acid (2a)

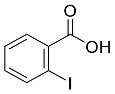

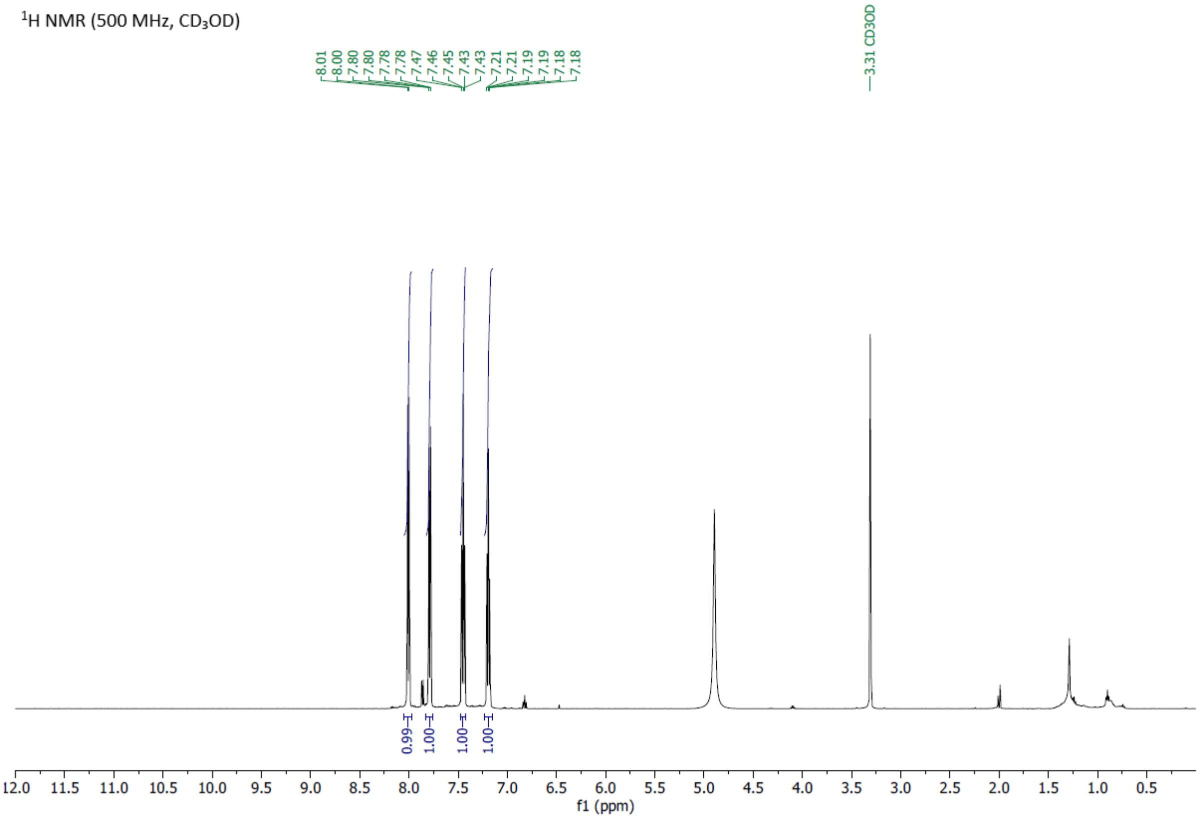

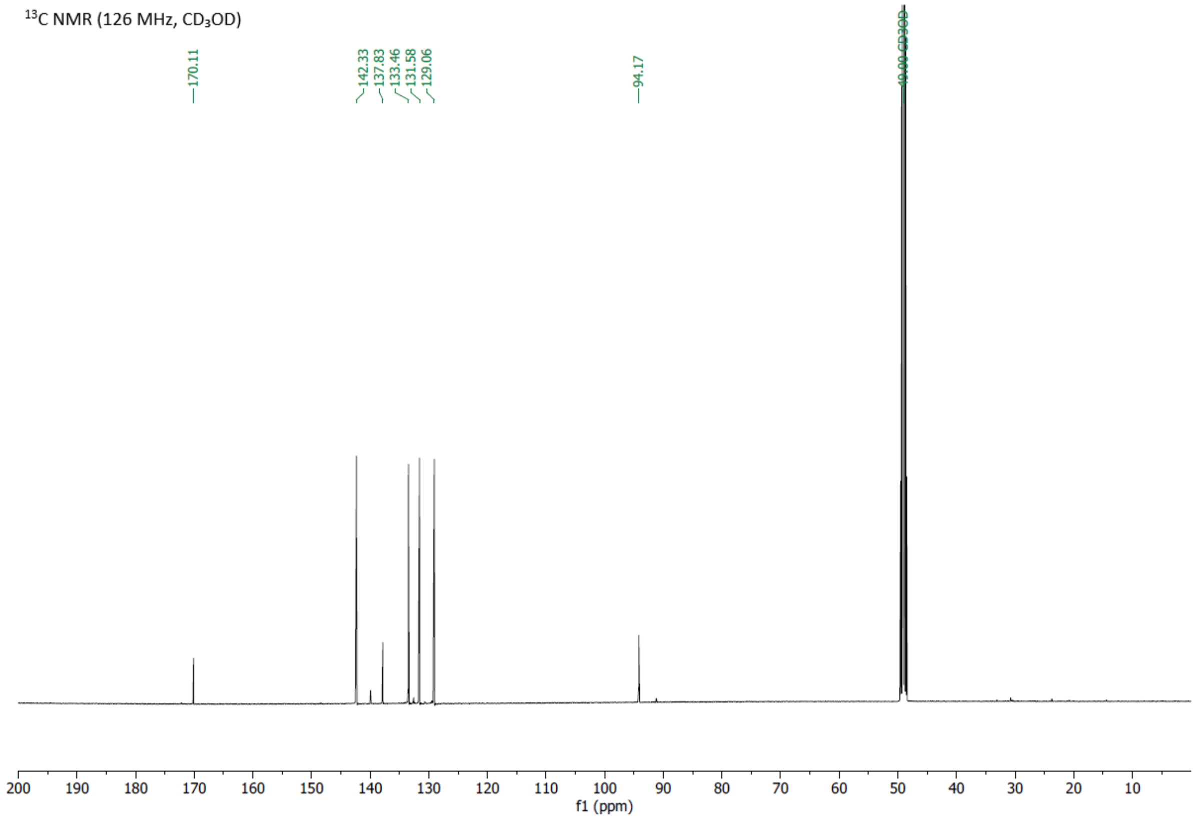

2-iodo-4-methylbenzoic acid (2b)

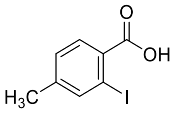

1H NMR (500 MHz, $CDCl_3$)

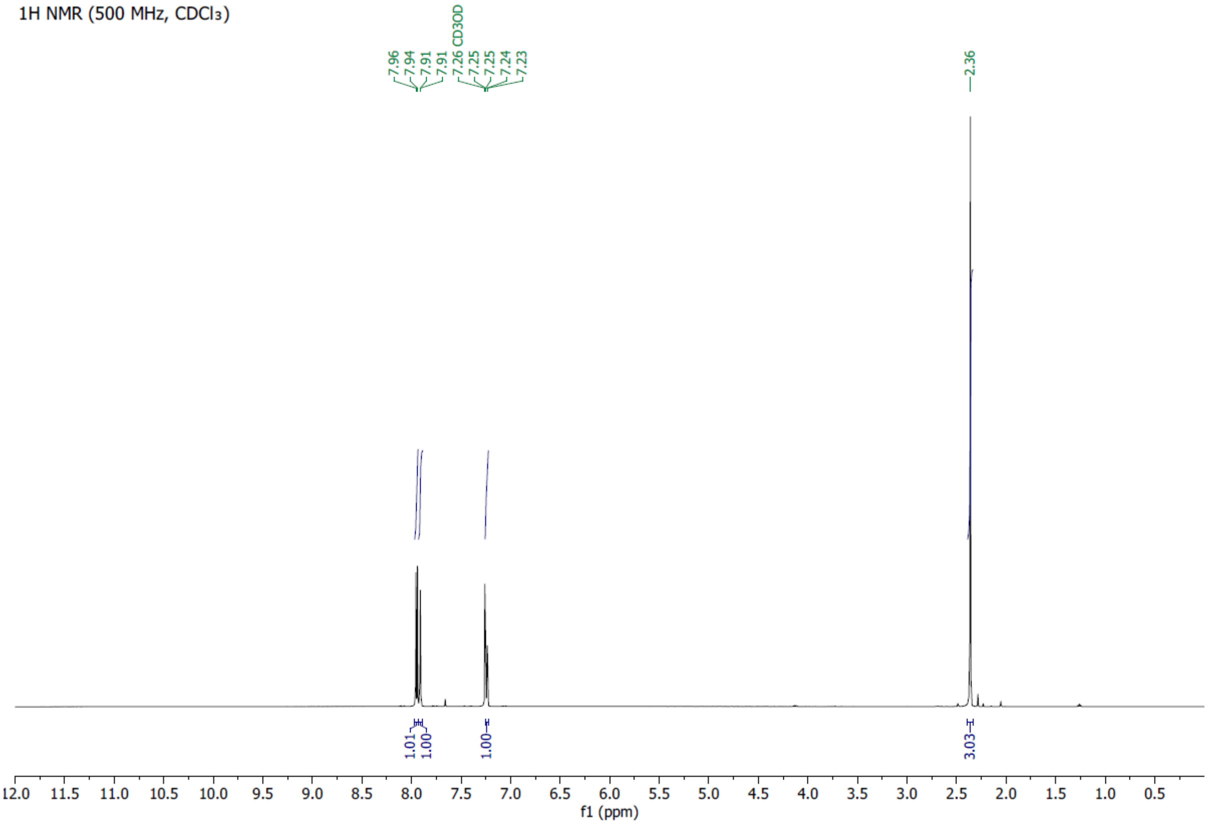

13C NMR (126 MHz, CDCl3)

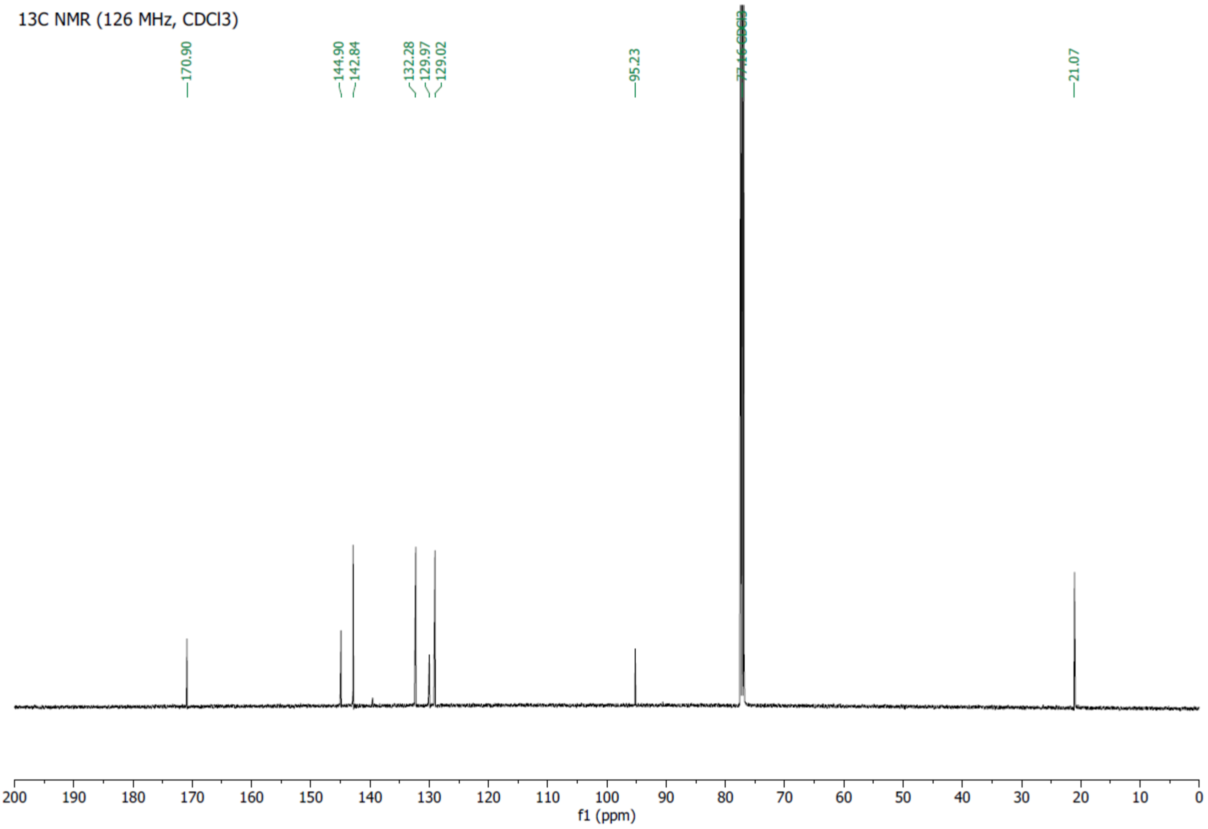

4-cyclohexyl-2-iodobenzoic acid (2c)

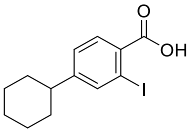

^{1}H NMR (500 MHz, CD_3OD)

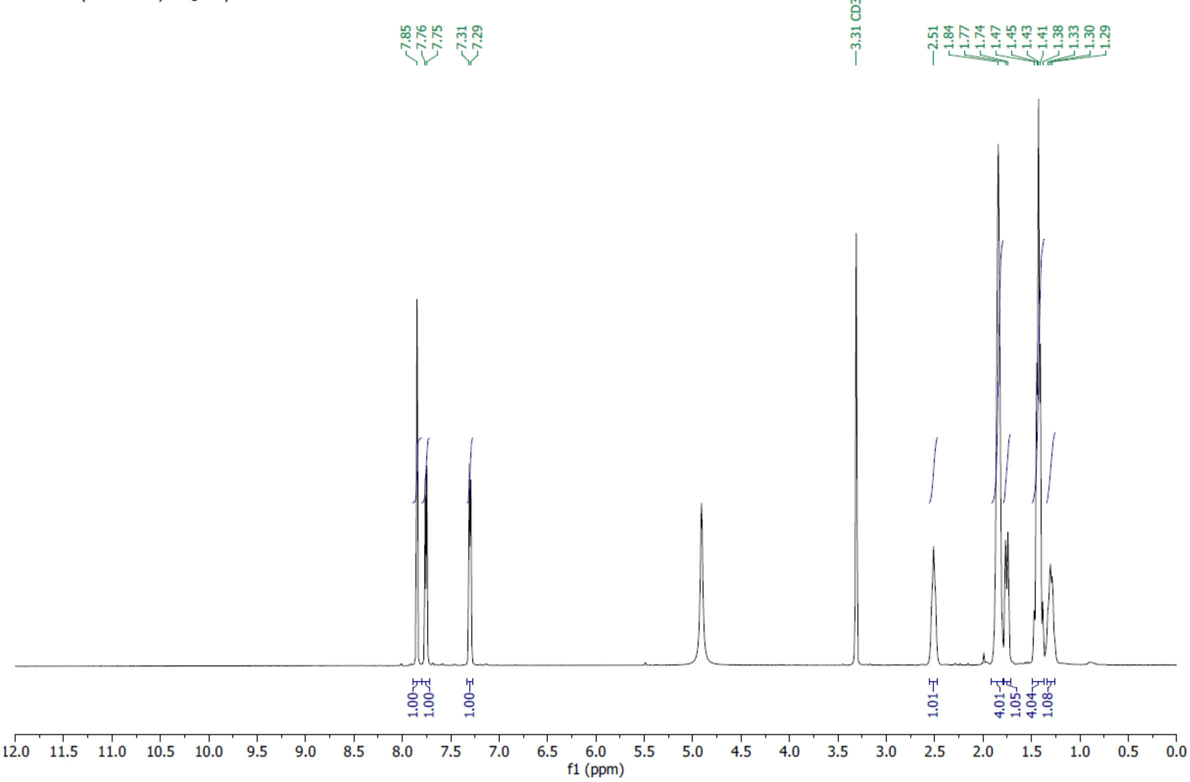

^{13}C NMR (126 MHz, CD_3OD)

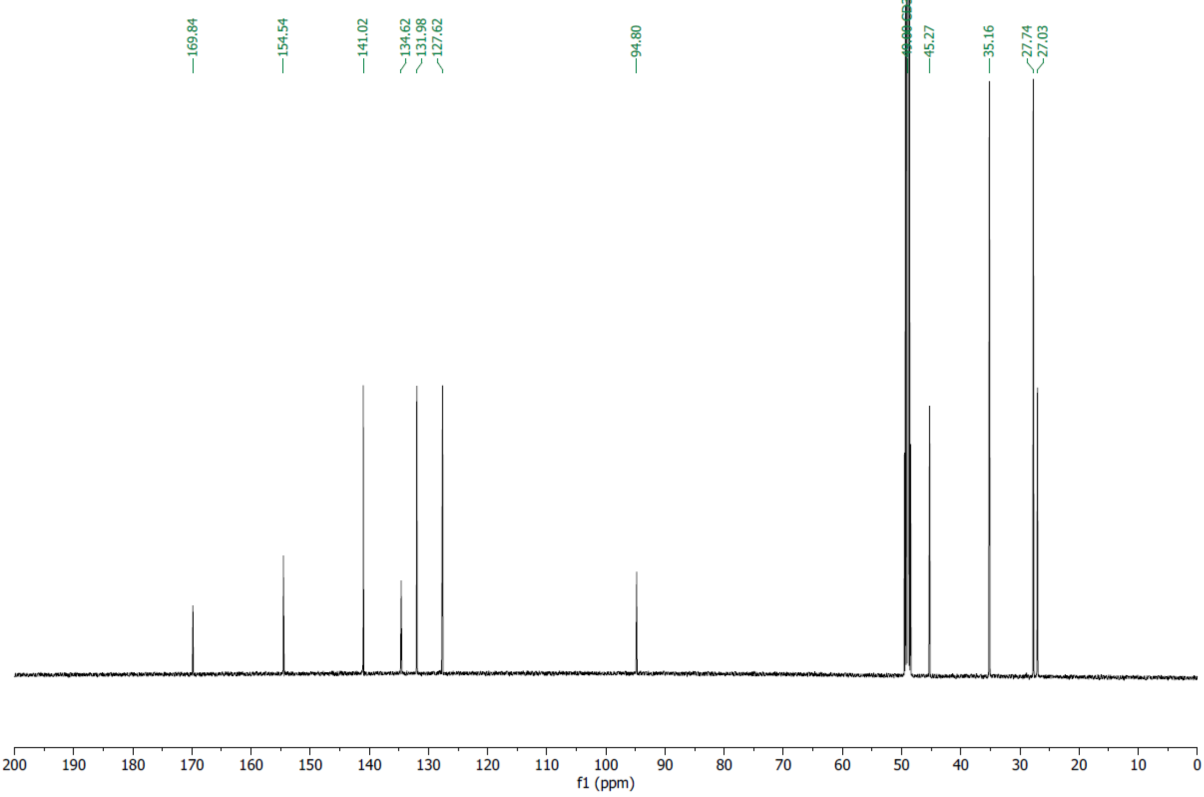

2-iodo-4-methoxybenzoic acid (2d)

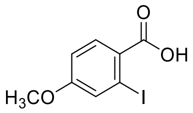

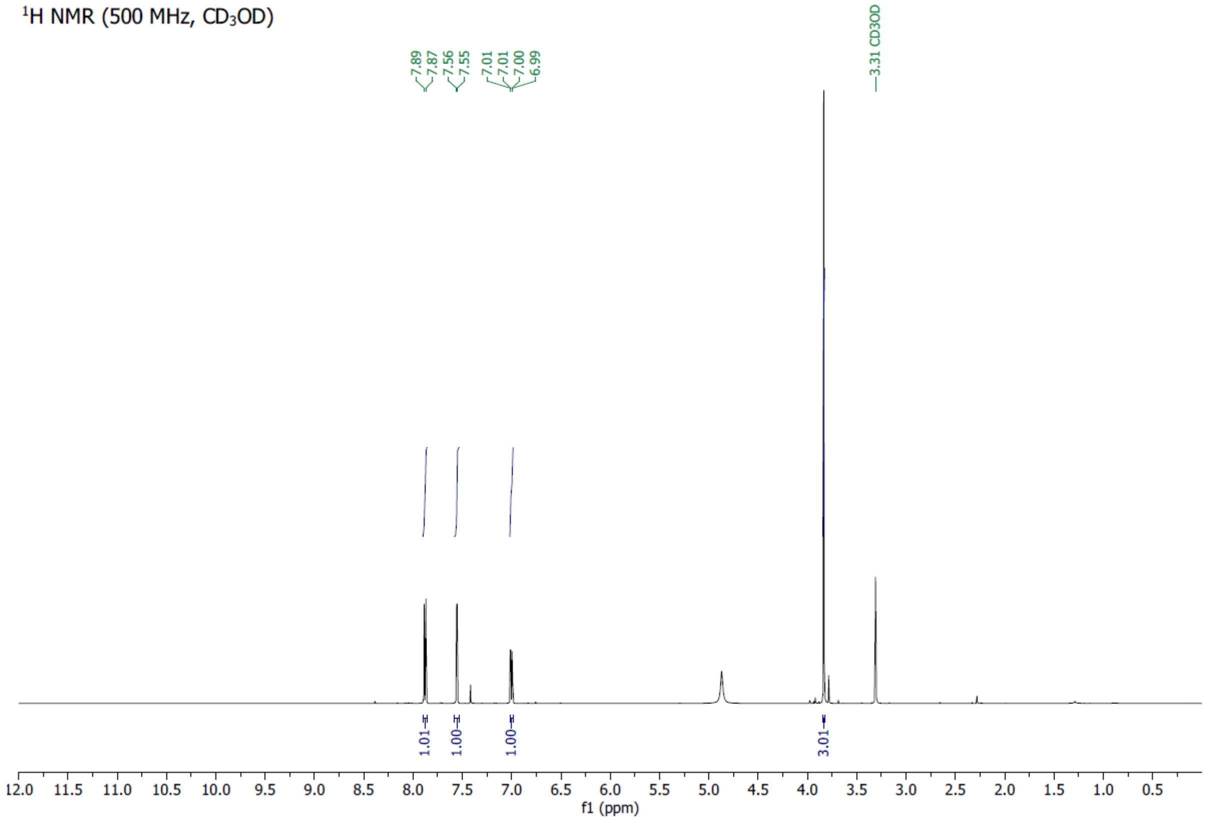

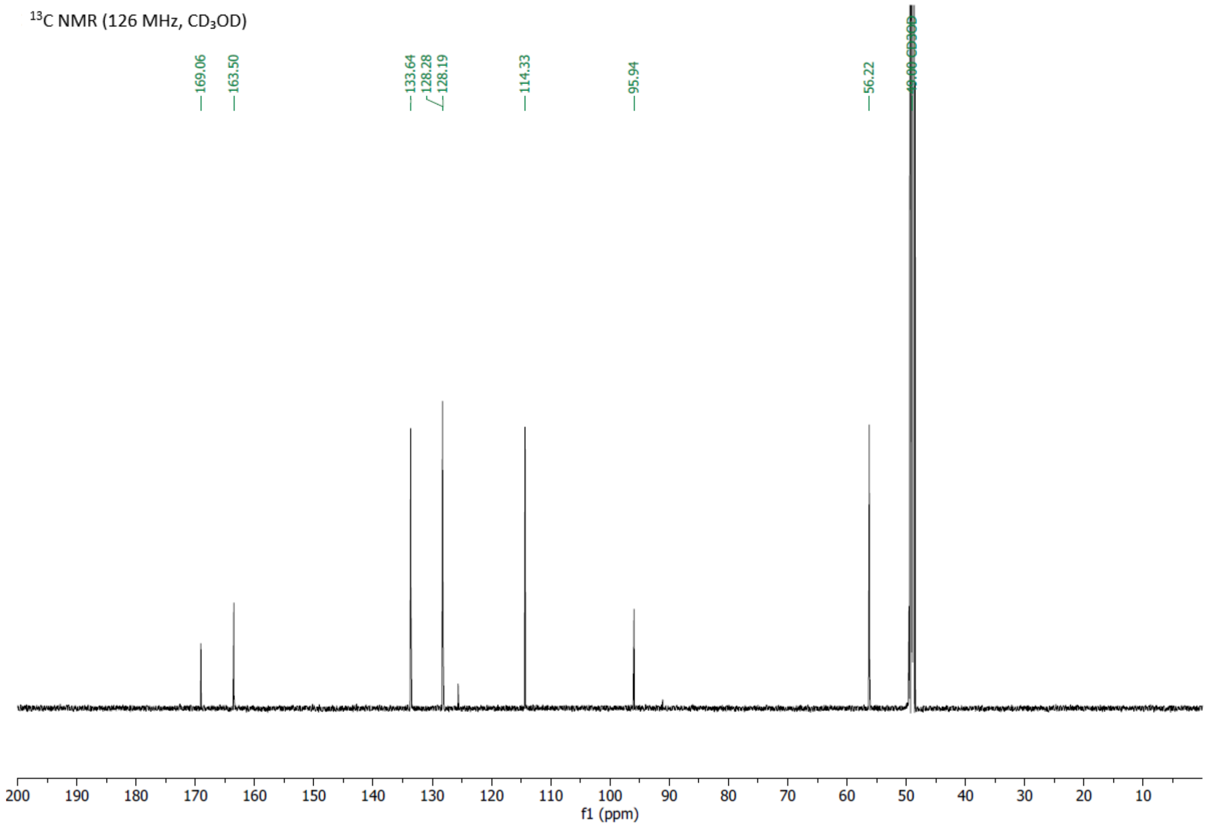

2-iodo-4-(sulfamoylmethyl) benzoic acid (2e)

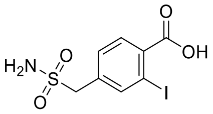

^{1}H NMR (500 MHz, CD_3OD)

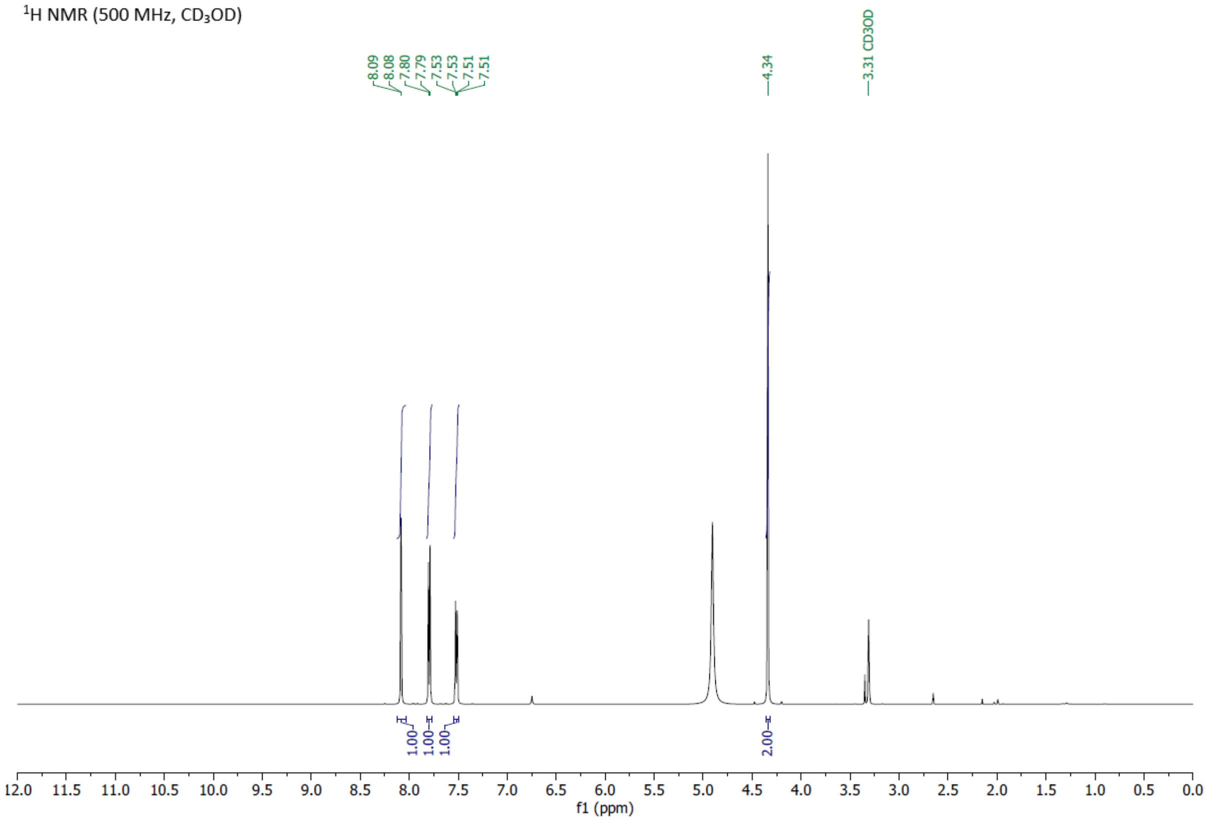

^{13}C NMR (126 MHz, CD_3OD)

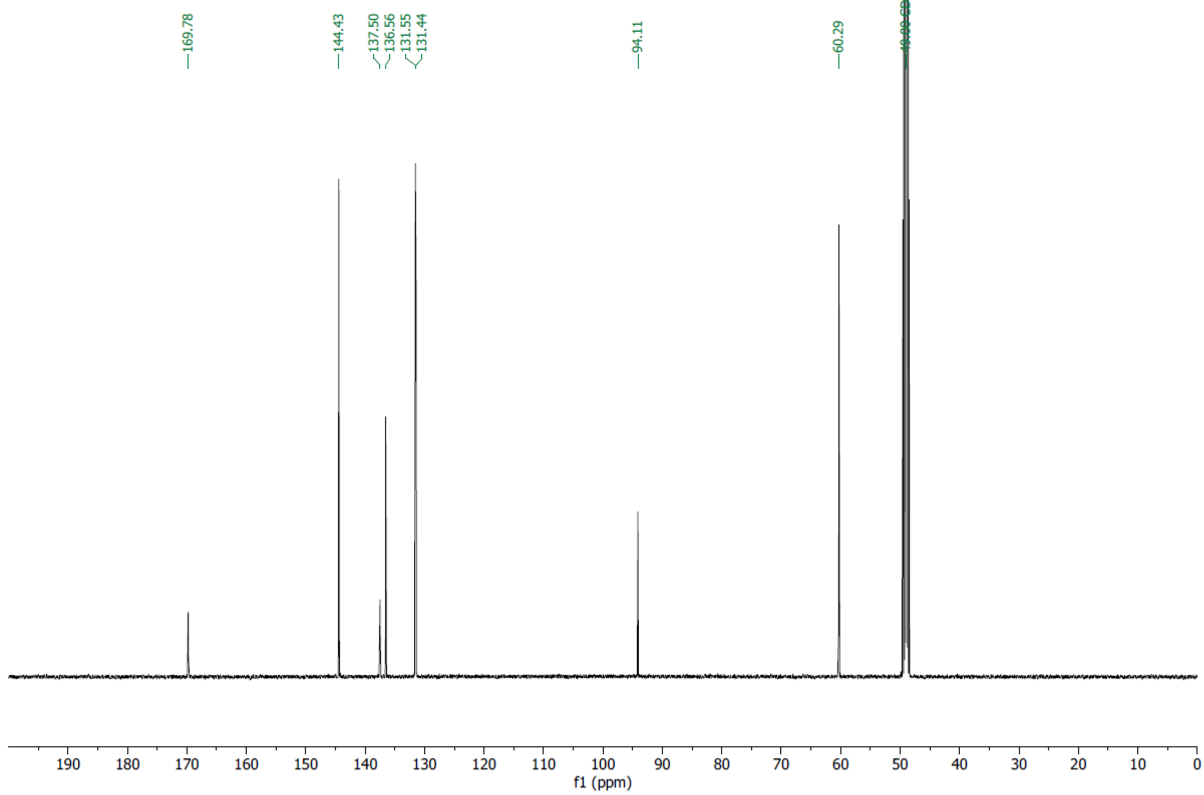

4-(2-hydroxyethyl)- 2-iodobenzoic acid (2f)

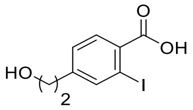

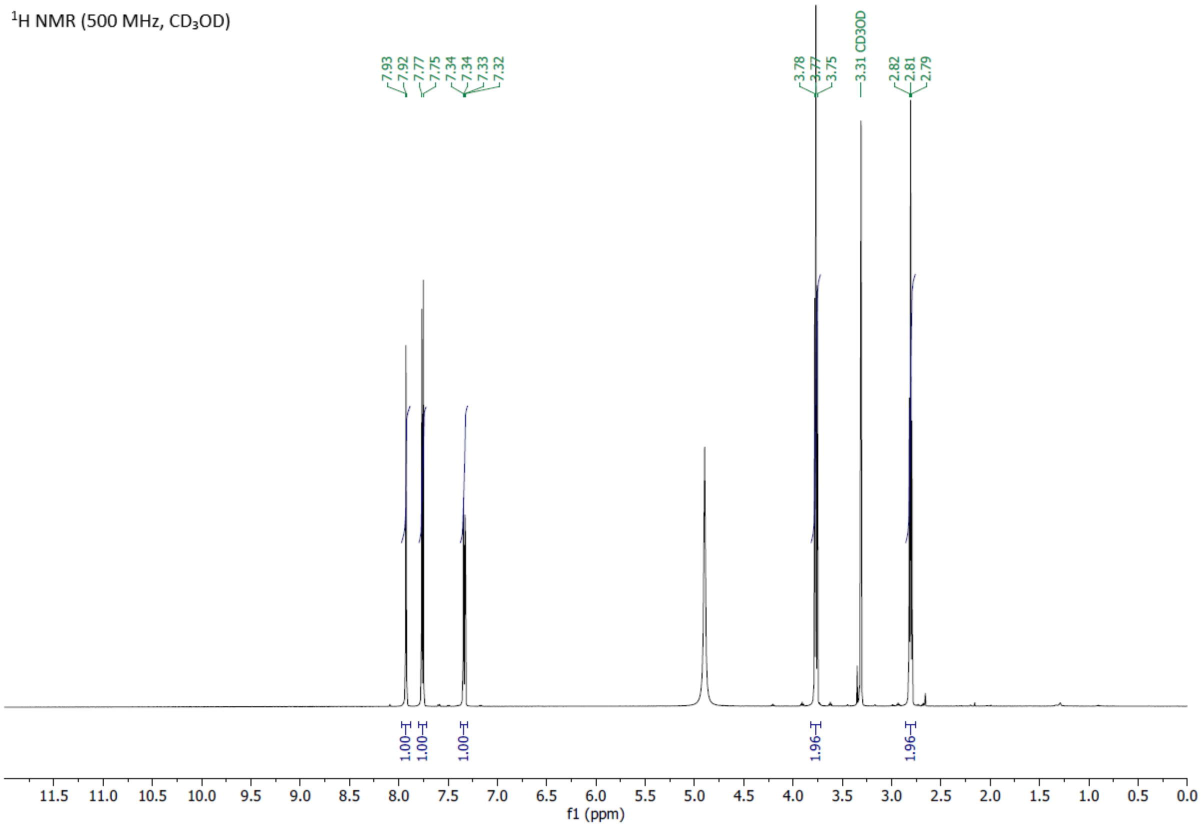

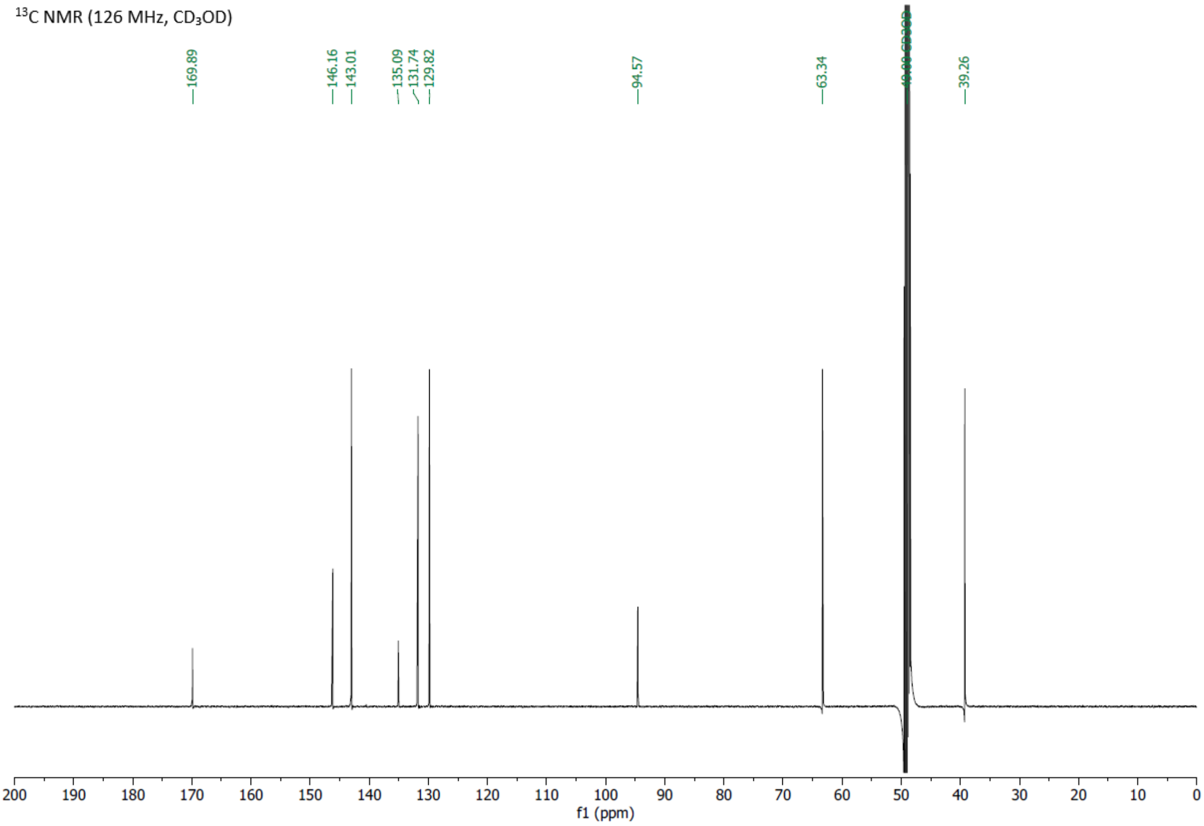

4-acetoxy-2-iodobenzoic acid (2g)

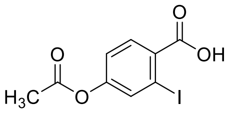

^{1}H NMR (500 MHz, CD_3OD)

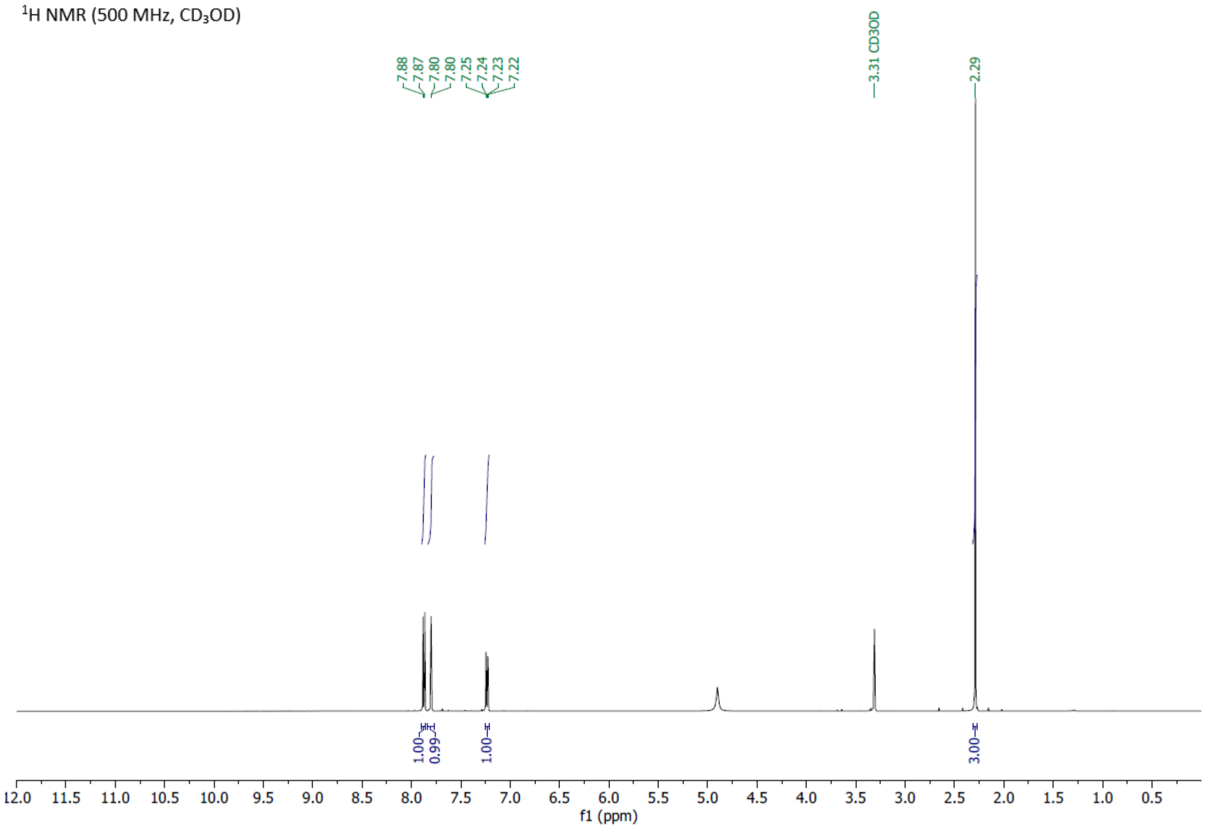

^{13}C NMR (126 MHz, CD_3OD)

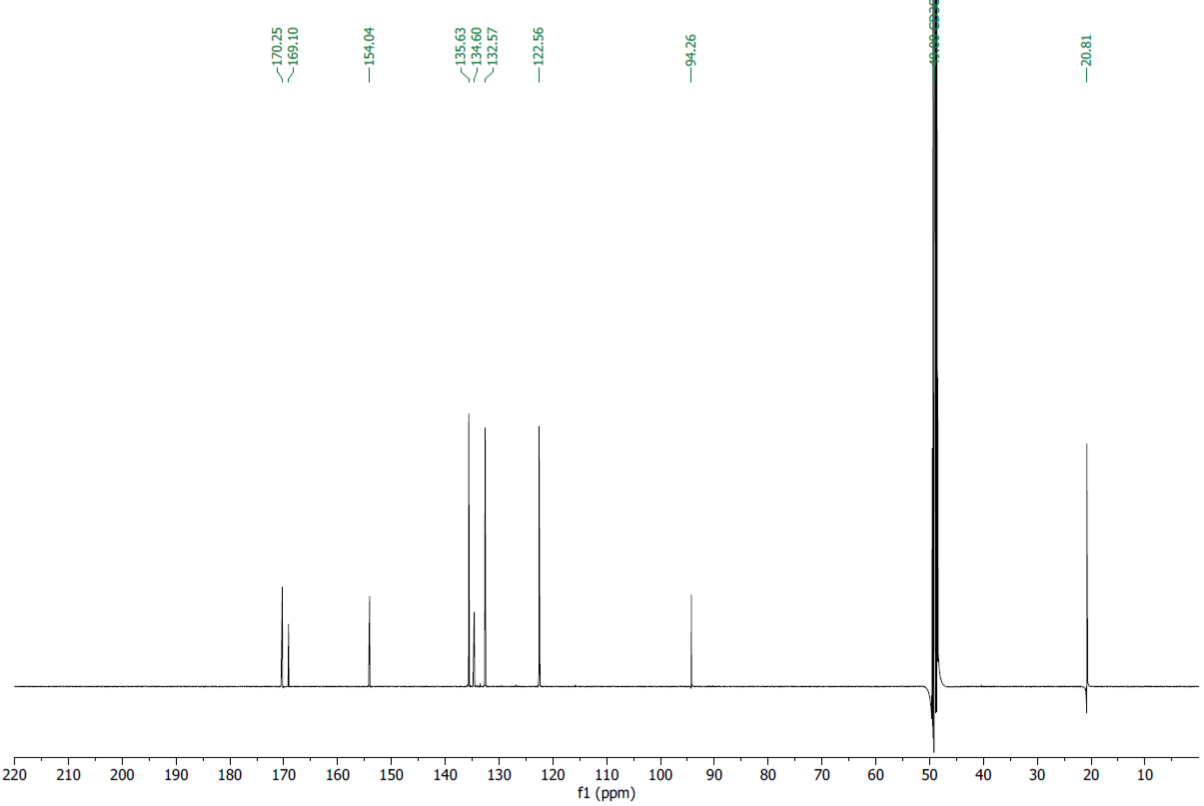

4-fluoro-2-iodobenzoic acid (2h)

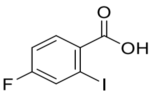

^{1}H NMR (500 MHz, CD_3OD)

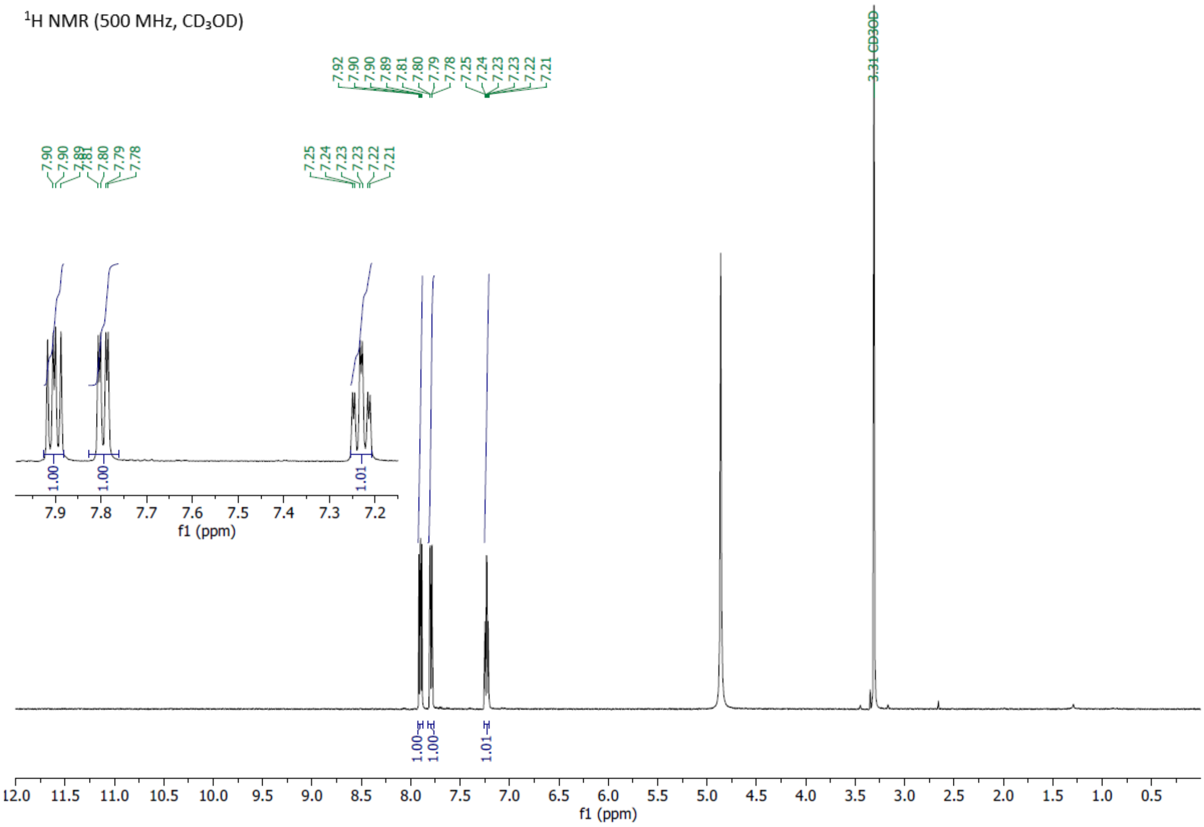

^{13}C NMR (126 MHz, CD_3OD)

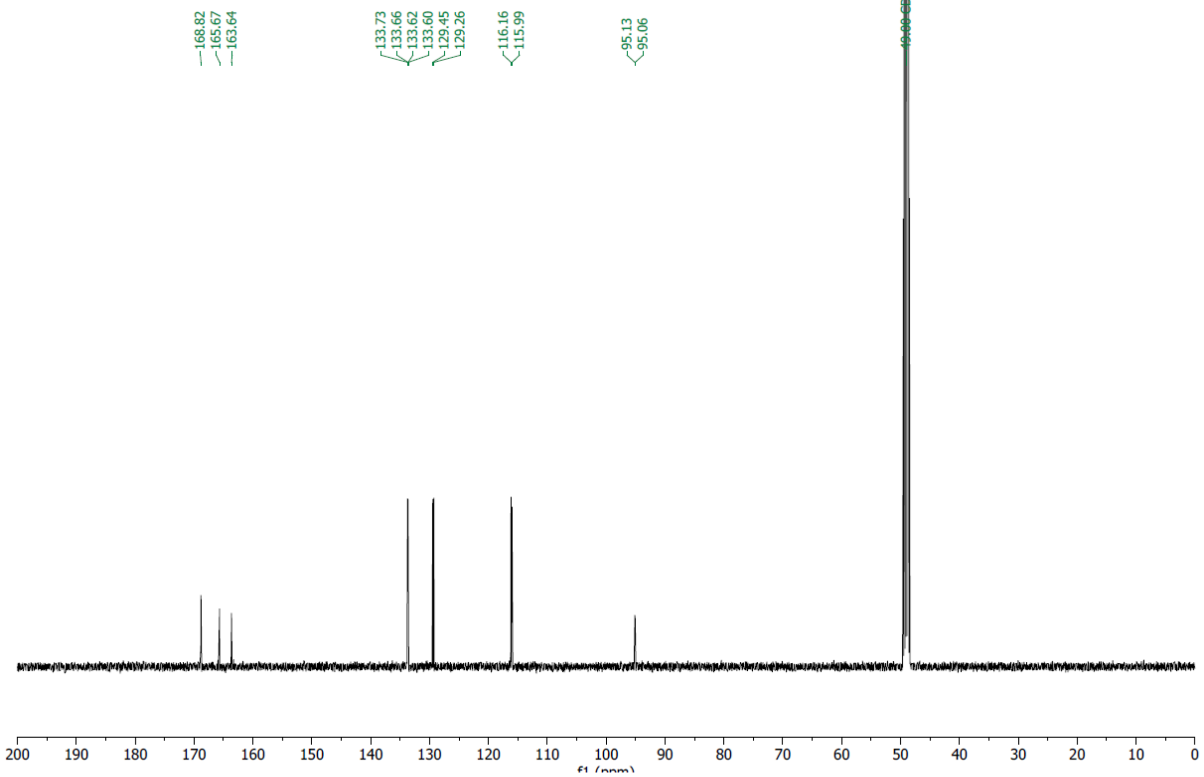

^{19}F NMR (470 MHz, CD_3OD)

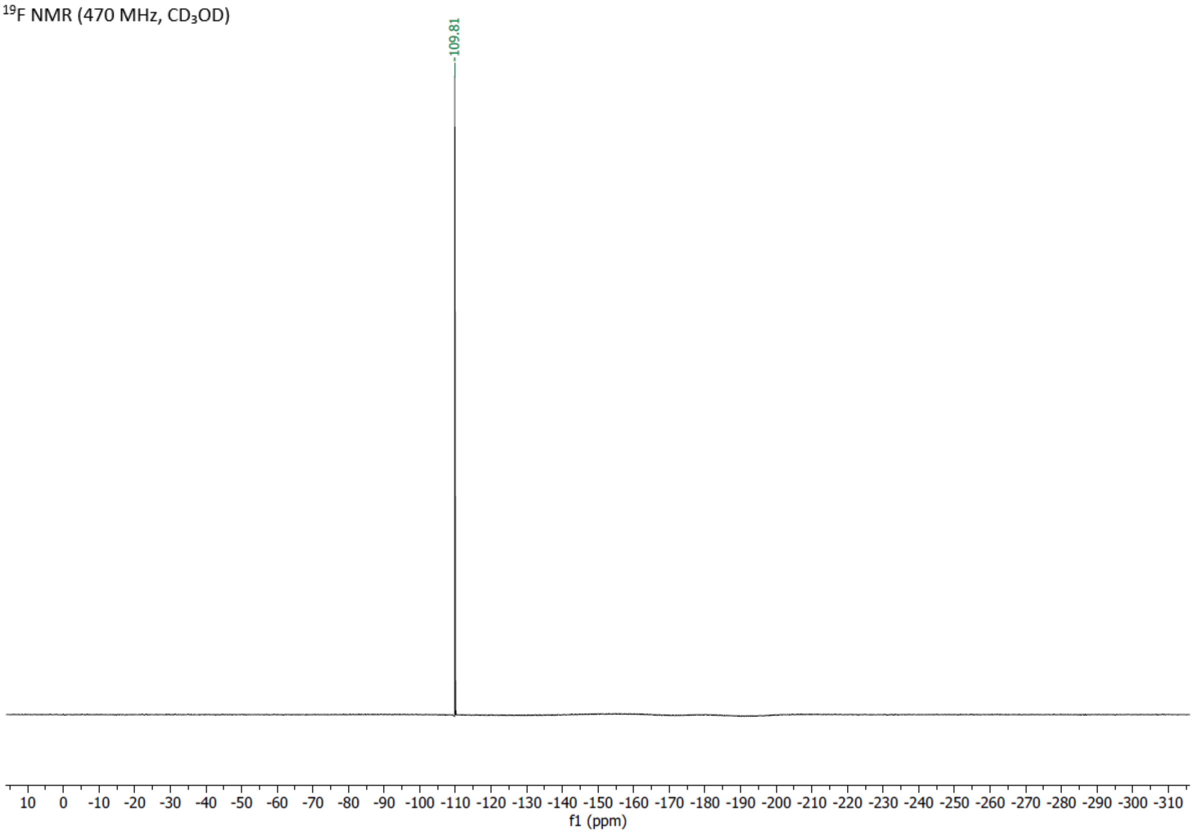

4-chloro-2-iodobenzoic acid (2i)

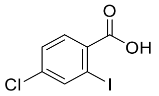

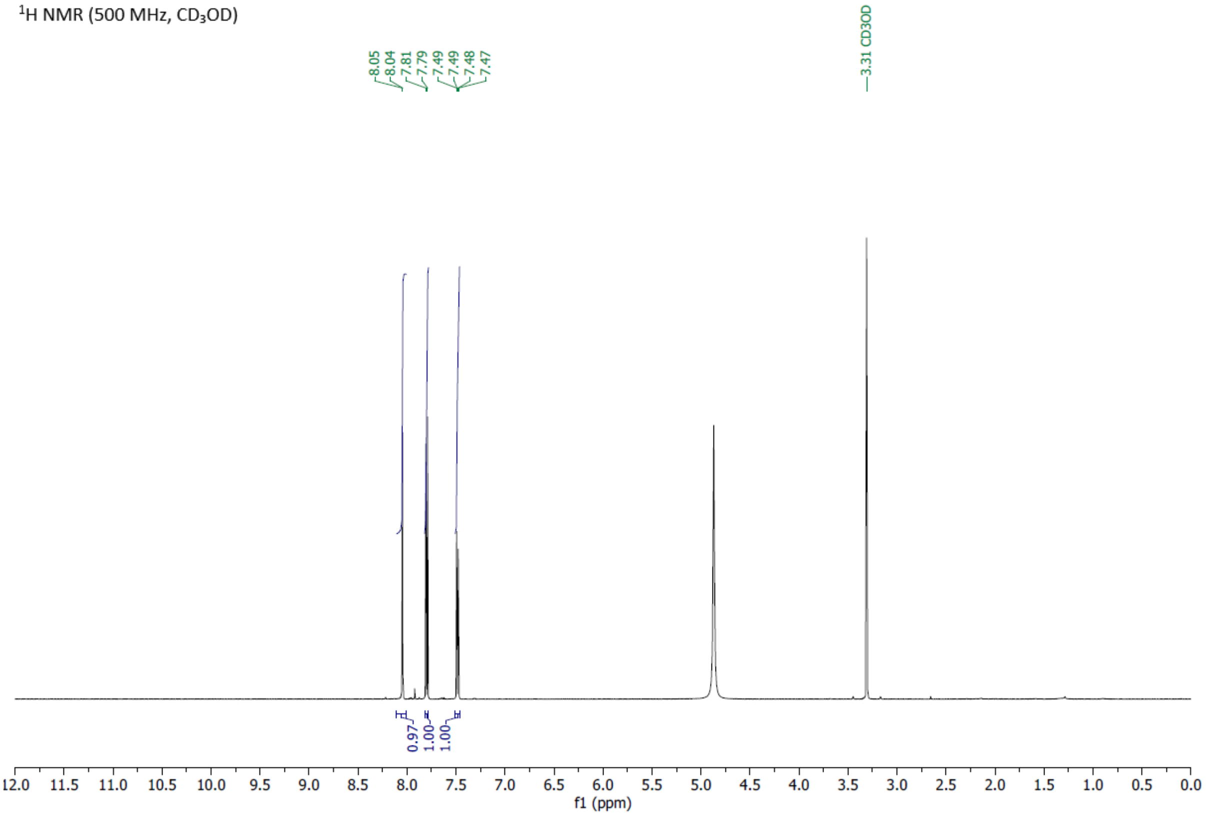

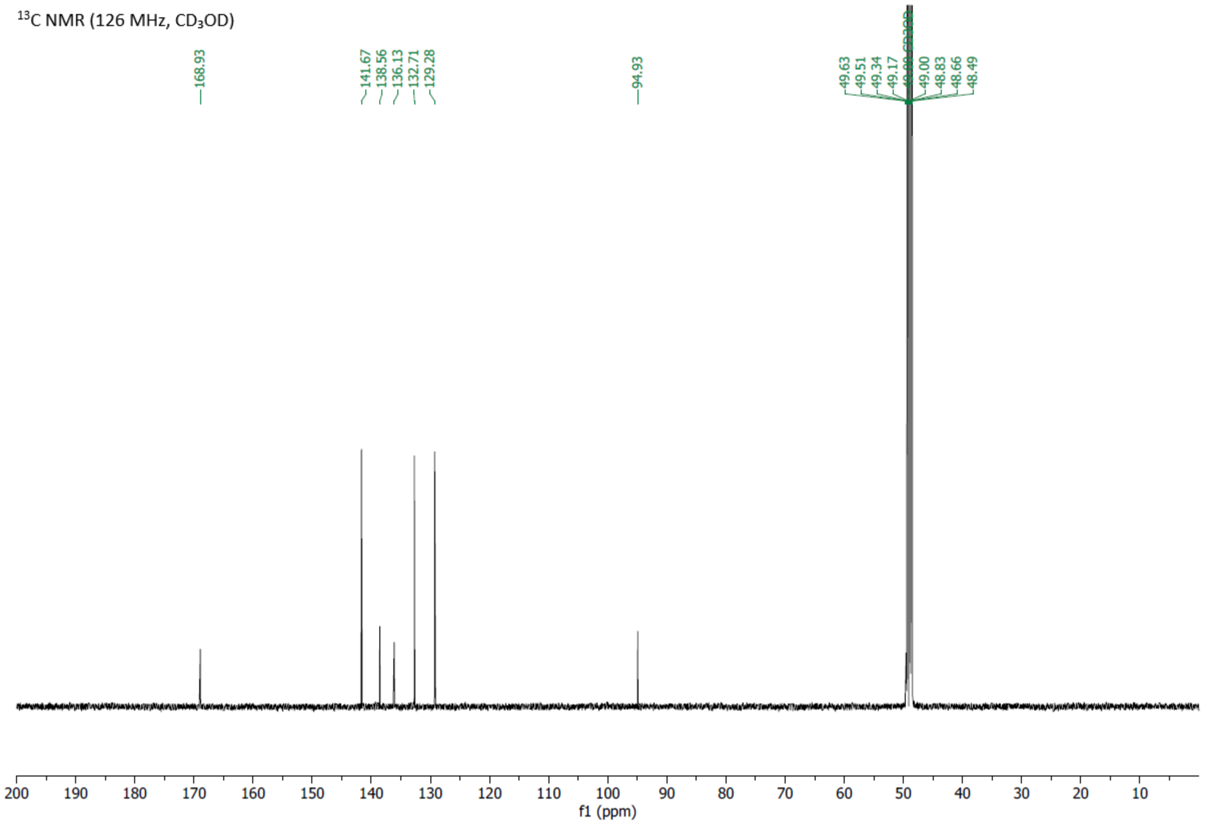

4-bromo-2-iodobenzoic acid (2j)

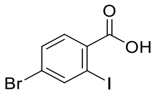

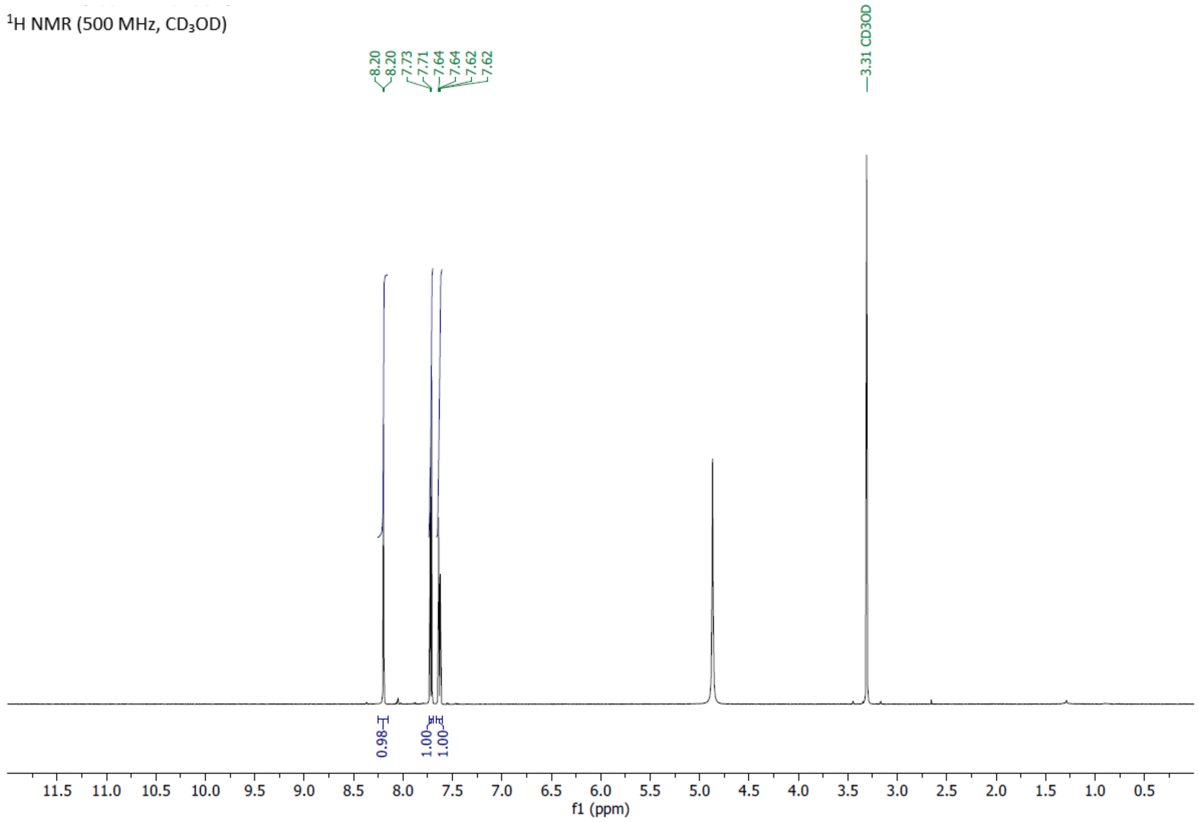

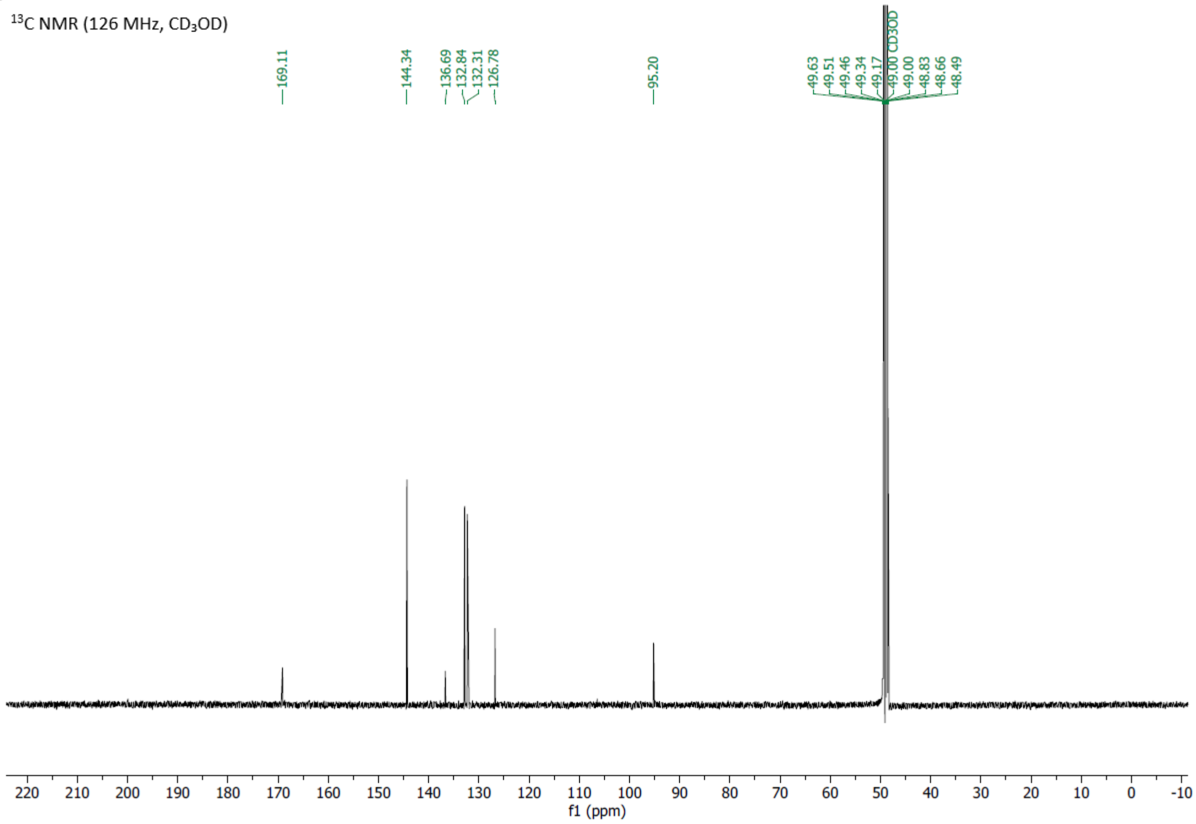

2-iodo-4-(trifluoromethoxy) benzoic acid (2k)

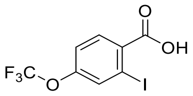

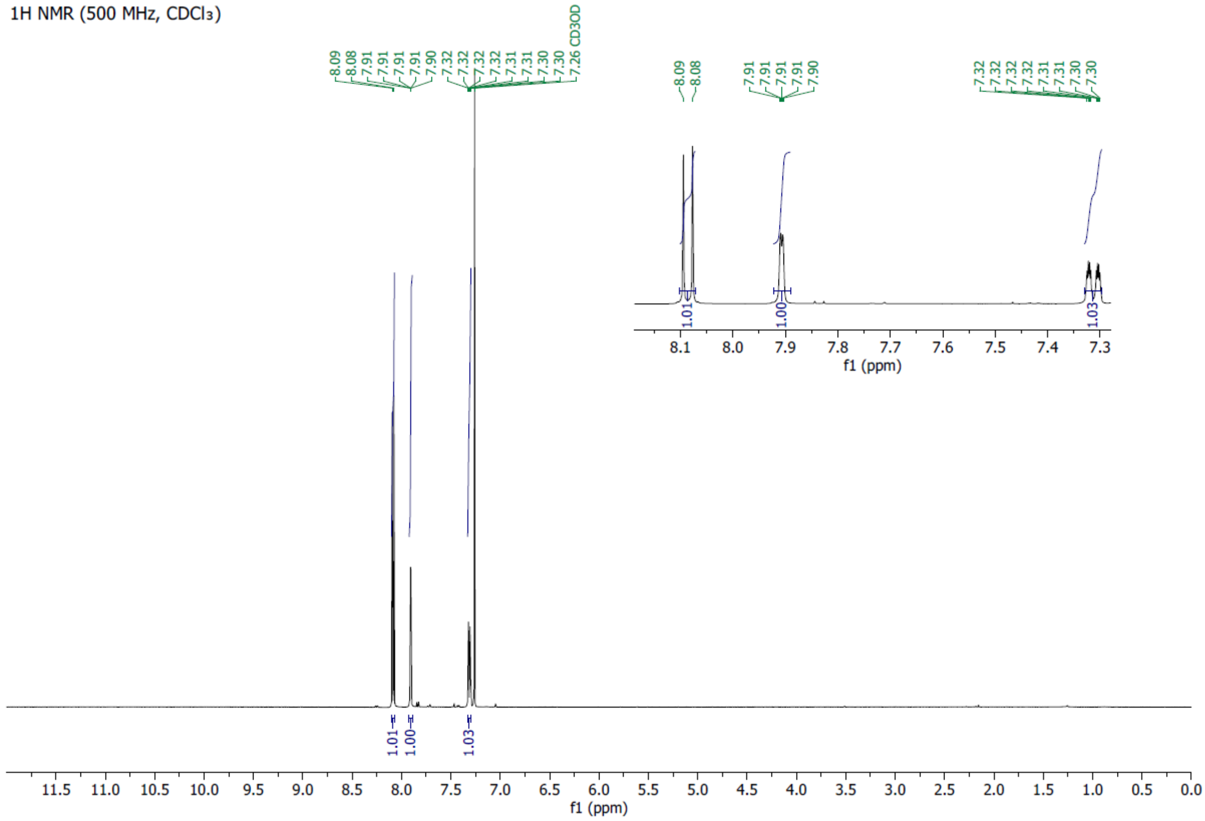

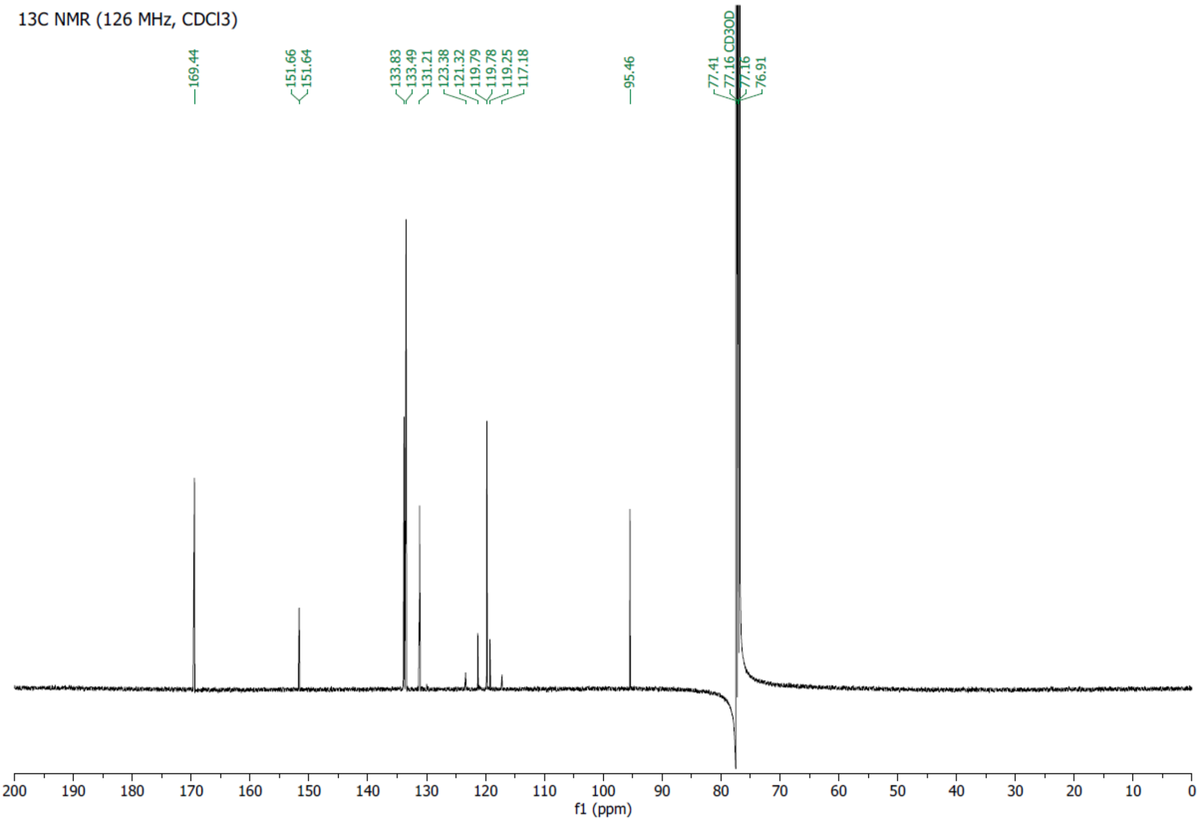

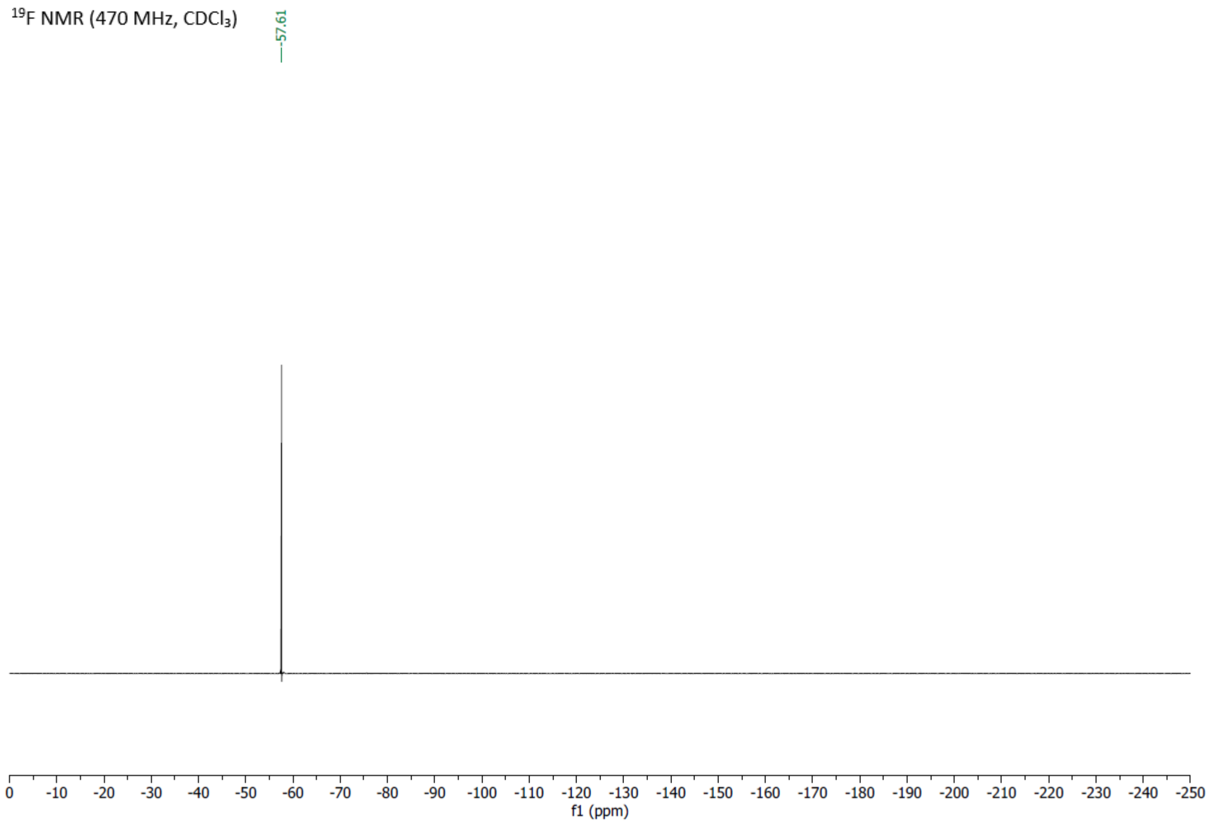

^{19}F NMR (470 MHz, $CDCl_3$)
-57.61
0 -10 -20 -30 -40 -50 -60 -70 -80 -90 -100 -110 -120 -130 -140 -150 -160 -170 -180 -190 -200 -210 -220 -230 -240 -250
f1 (ppm)

2-iodo-4-(trifluoromethyl) benzoic acid (2l)

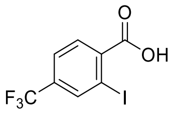

^{1}H NMR (500 MHz, CD_3OD)

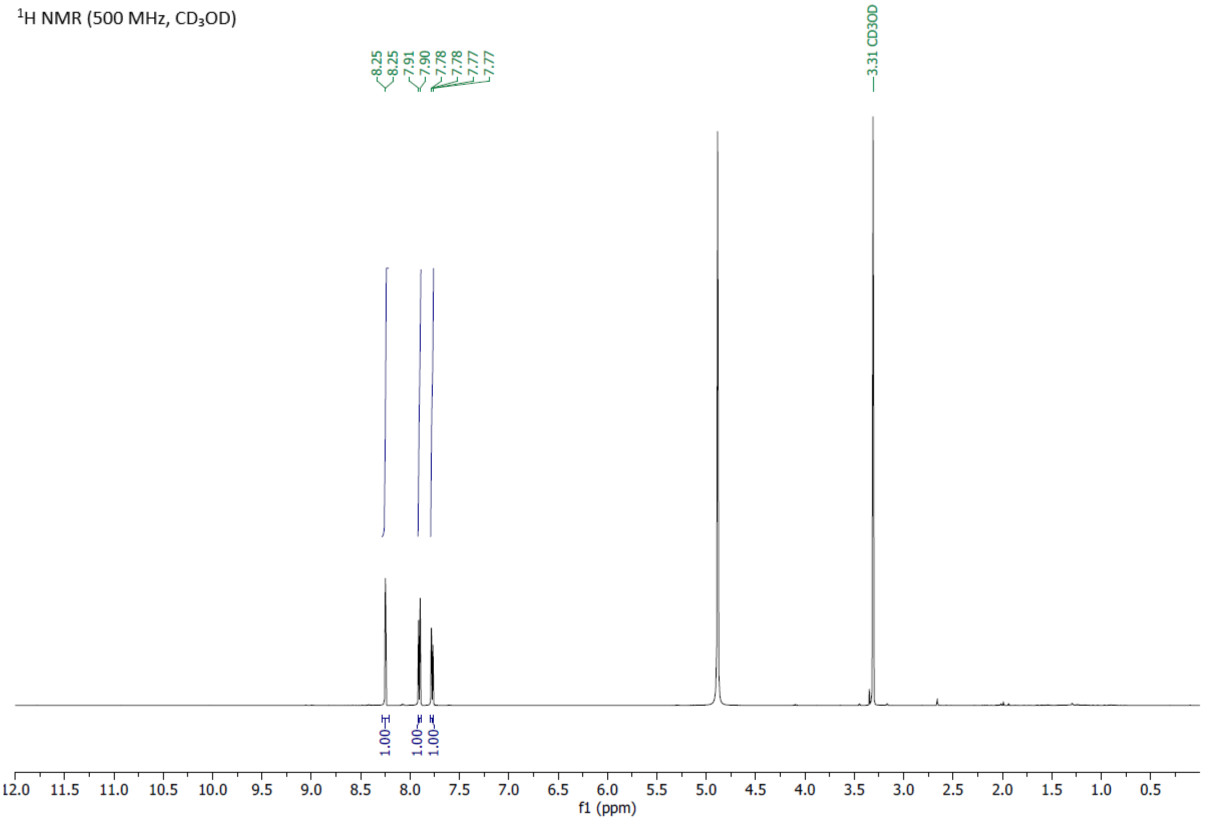

^{13}C NMR (126 MHz, CD_3OD)

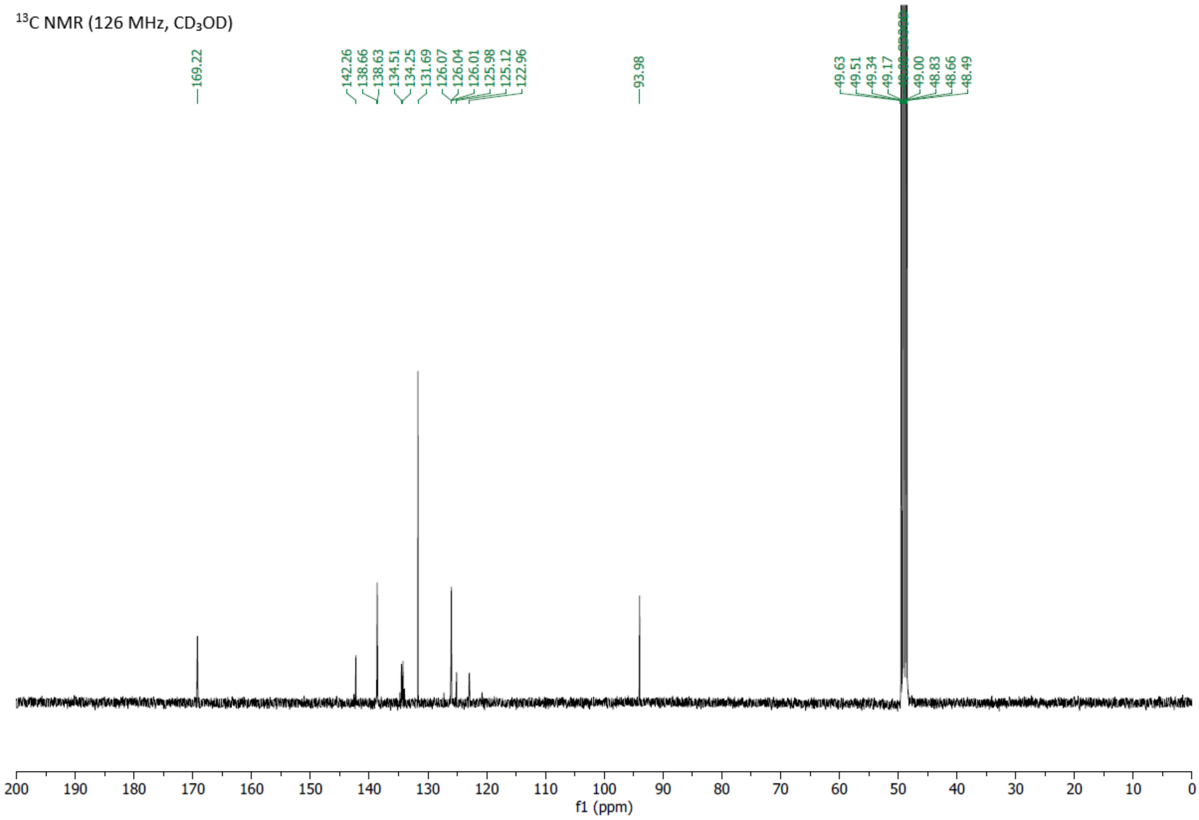

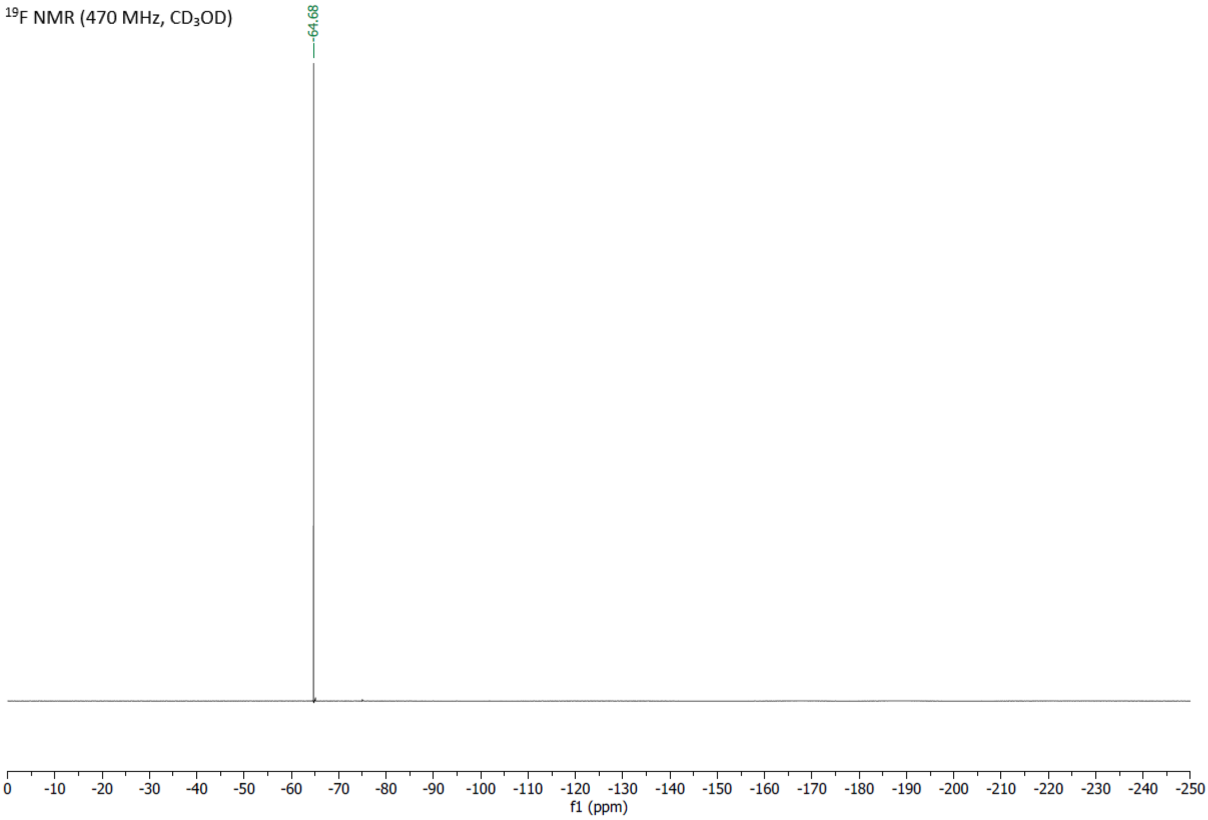

19F NMR (470 MHz, CD3OD)
-64.68
0 -10 -20 -30 -40 -50 -60 -70 -80 -90 -100 -110 -120 -130 -140 -150 -160 -170 -180 -190 -200 -210 -220 -230 -240 -250
f1 (ppm)

2-iodo-4-nitrobenzoic acid (2m)

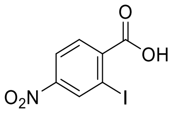

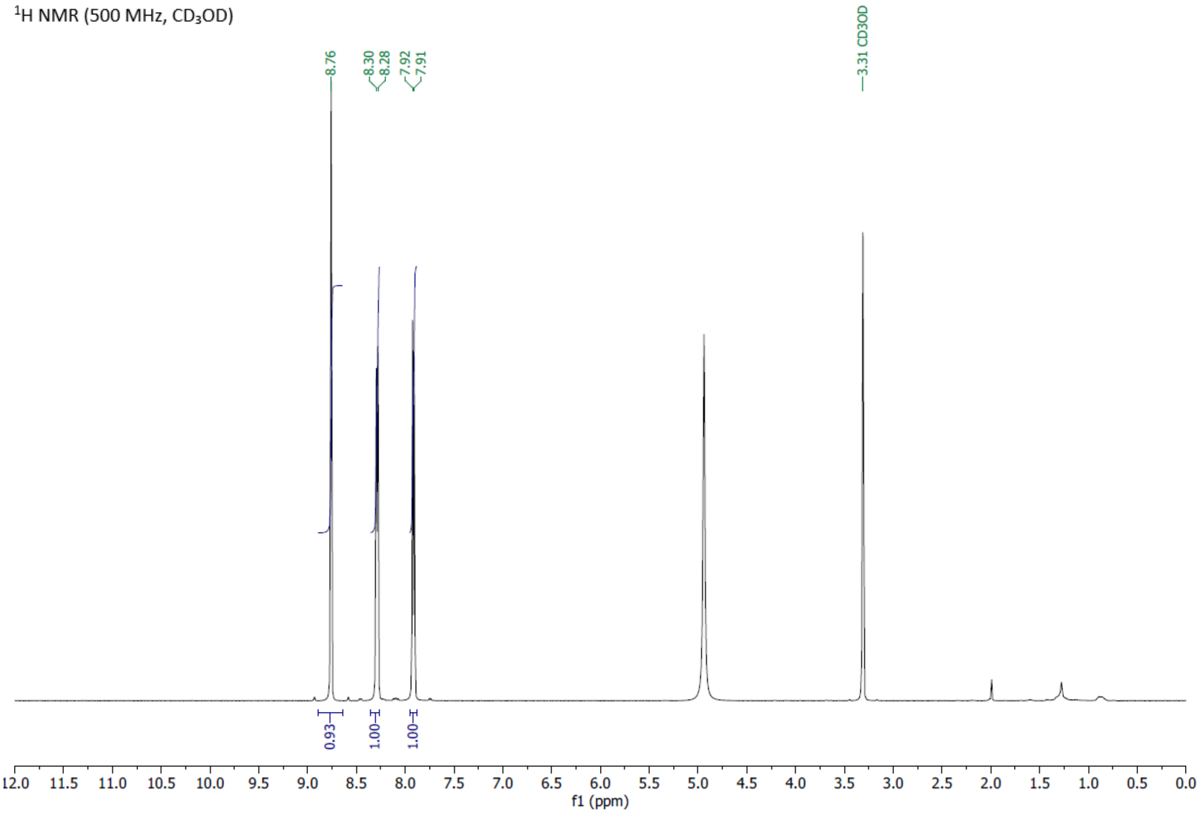

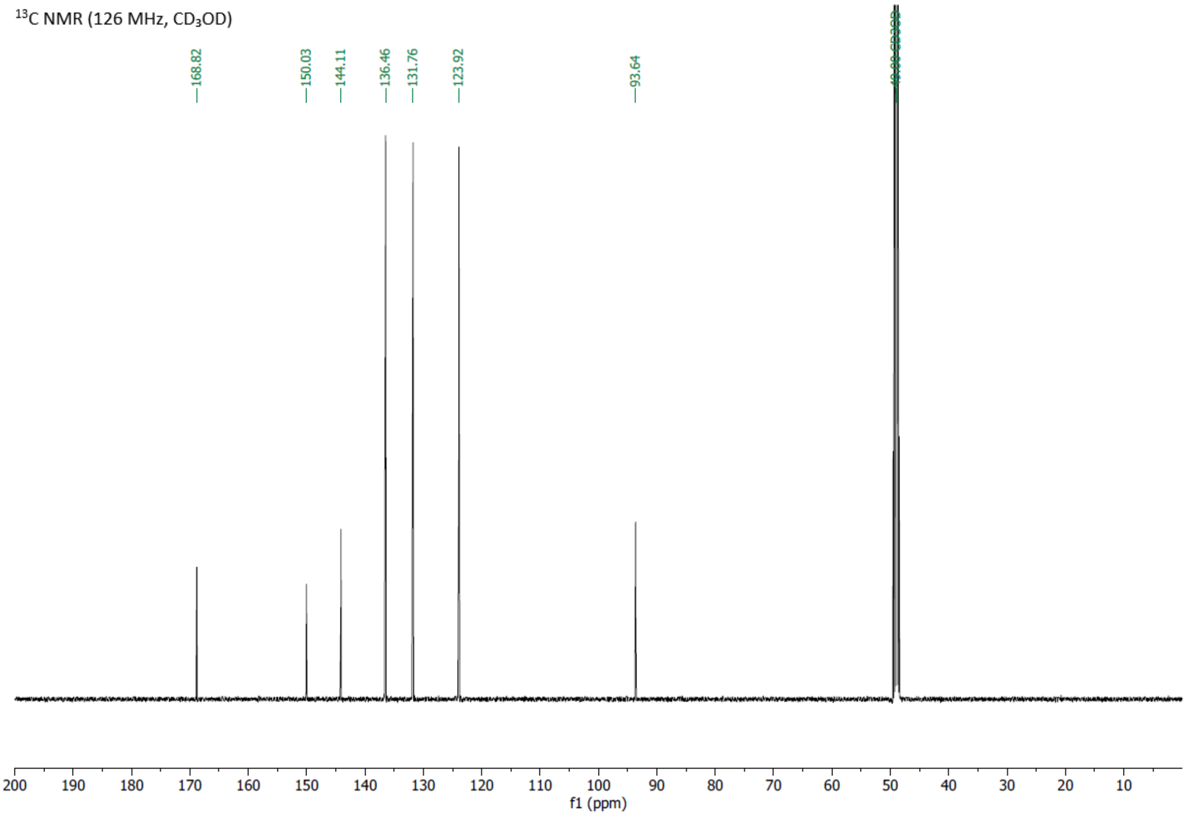

2-iodoterephthalic acid (2n)

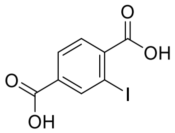

^{1}H NMR (500 MHz, CD_3OD)

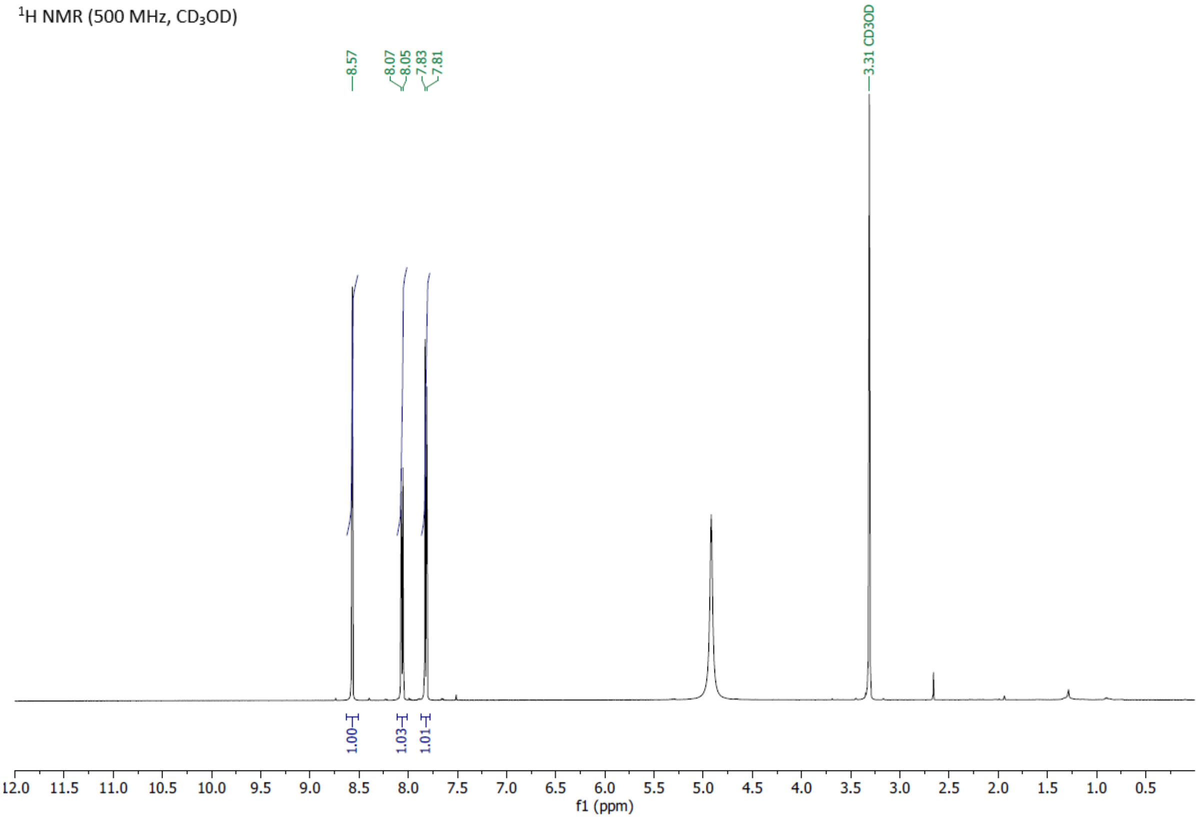

^{13}C NMR (126 MHz, CD_3OD)

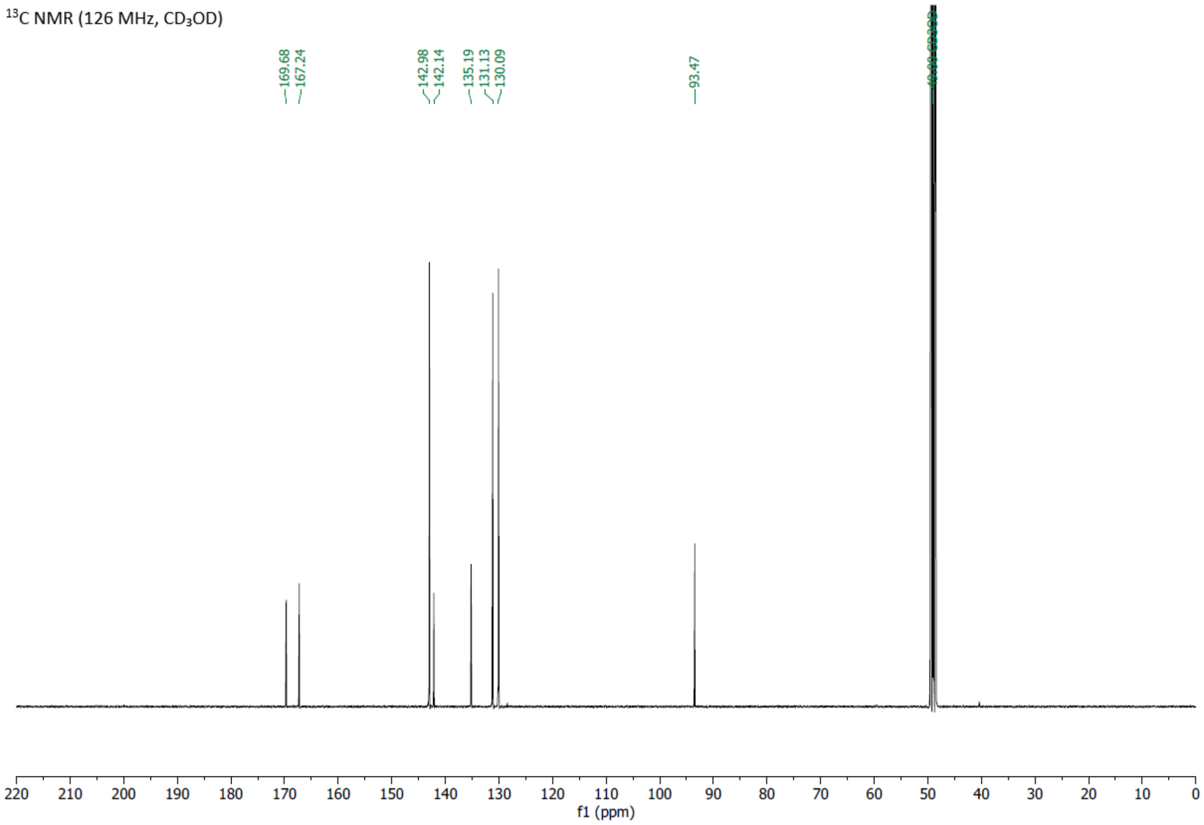

4-(carboxymethyl)-2-iodobenzoic acid (2o)

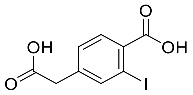

^{1}H NMR (500 MHz, CD_3OD)

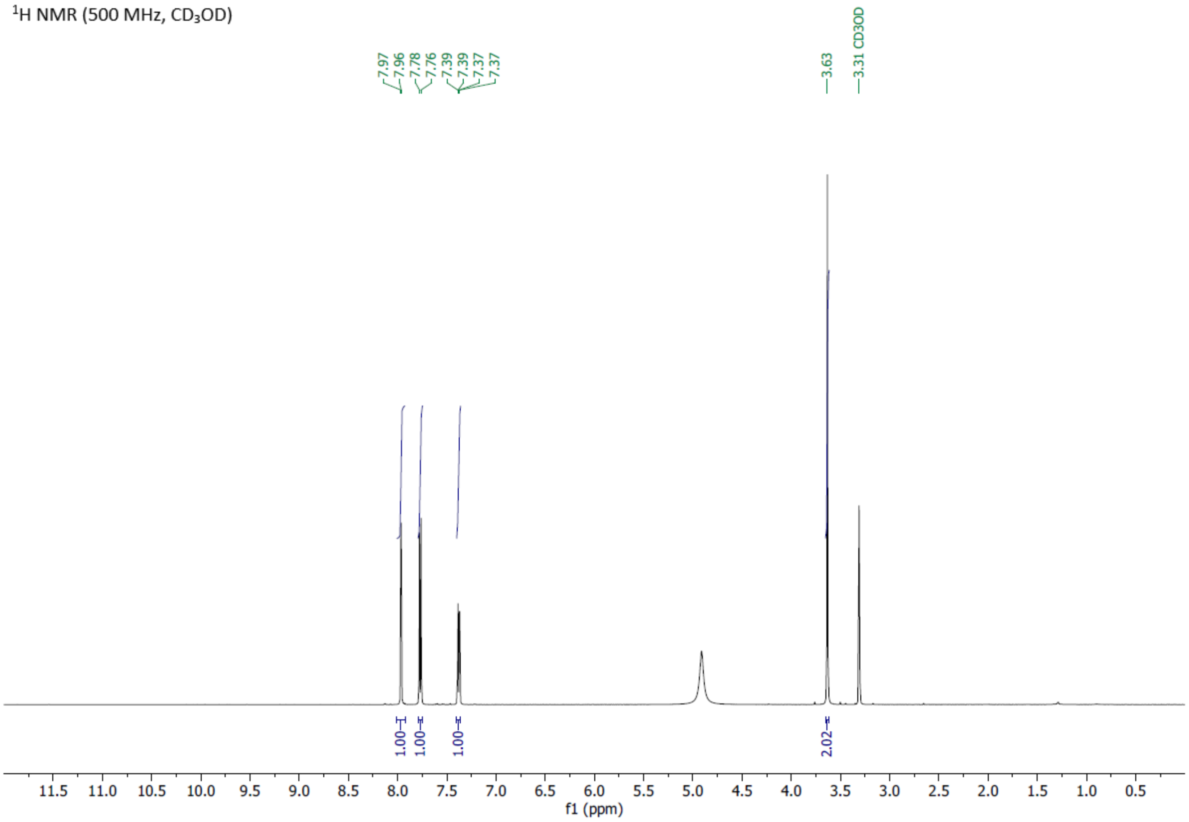

^{13}C NMR (126 MHz, CD_3OD)

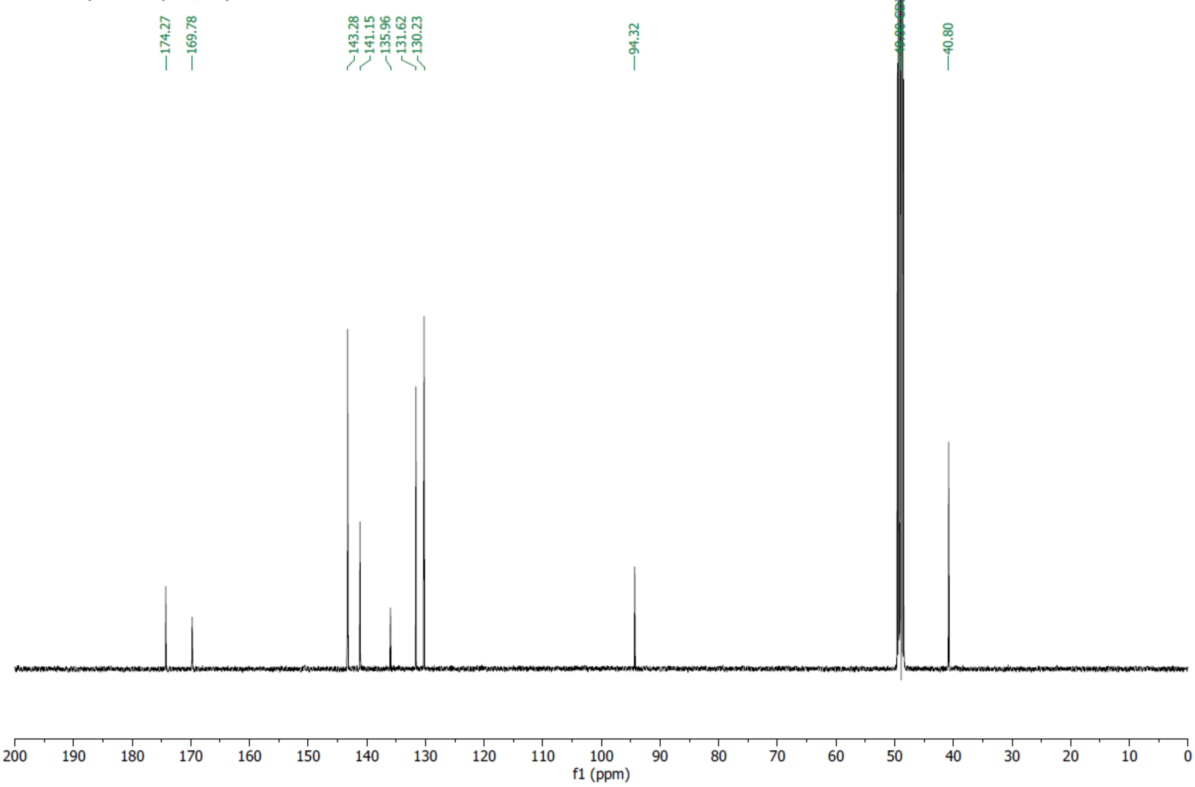

4-acetamido-2-iodobenzoic acid (2p)

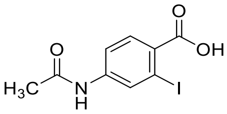

^{1}H NMR (500 MHz, CD_3OD)

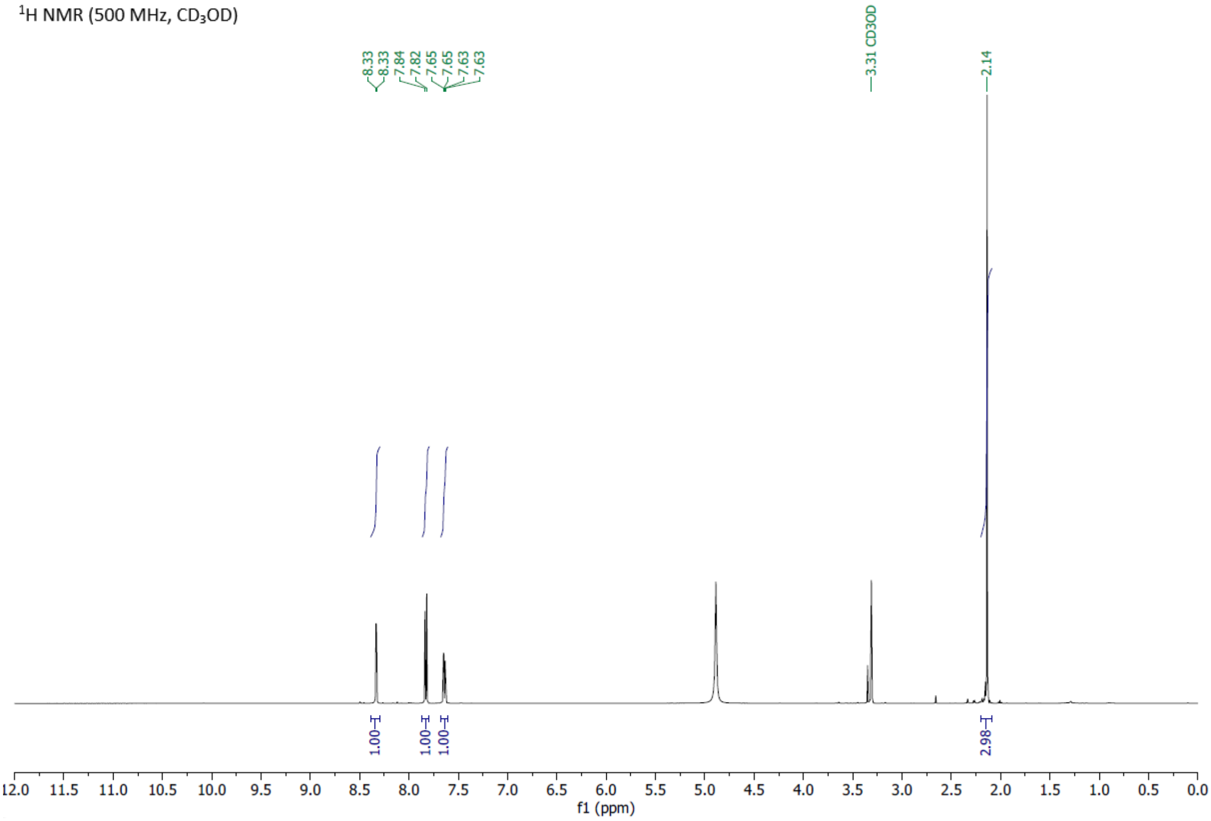

^{13}C NMR (126 MHz, CD_3OD)

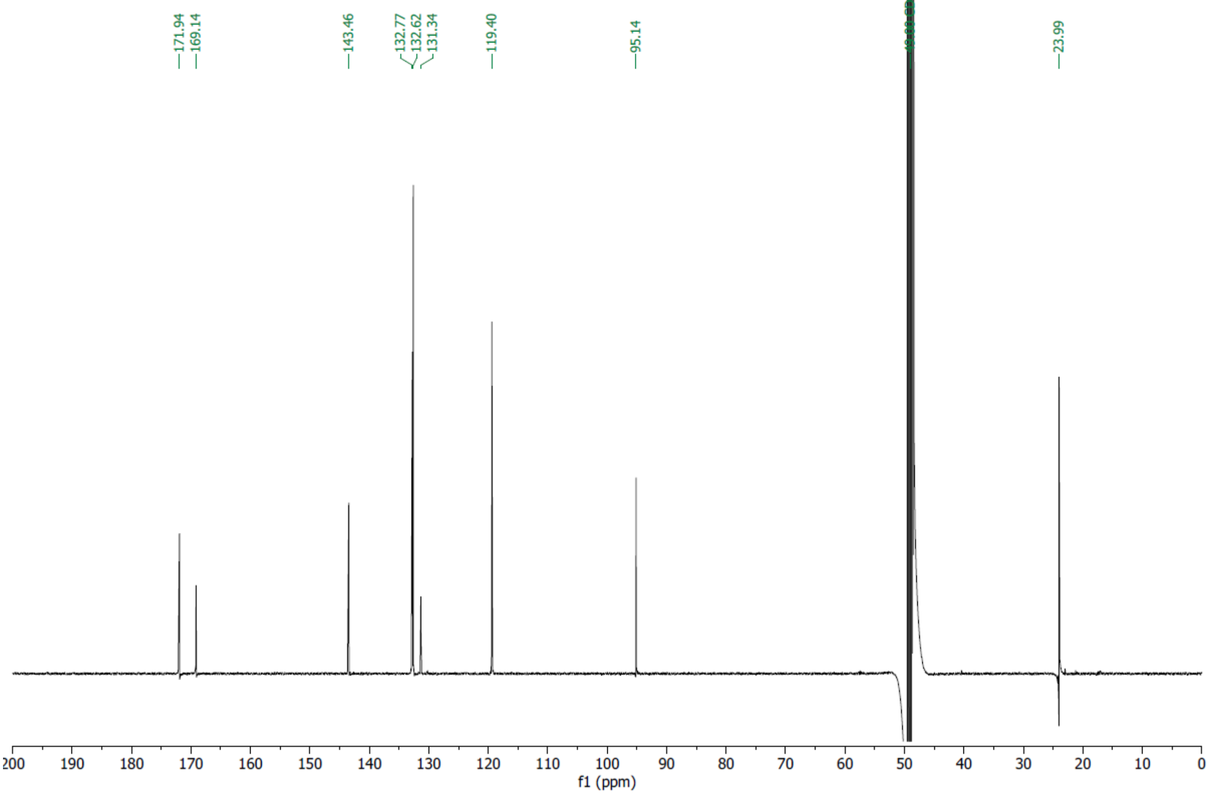

2-iodo-4-(methoxycarbonyl) benzoic acid (2q)

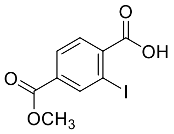

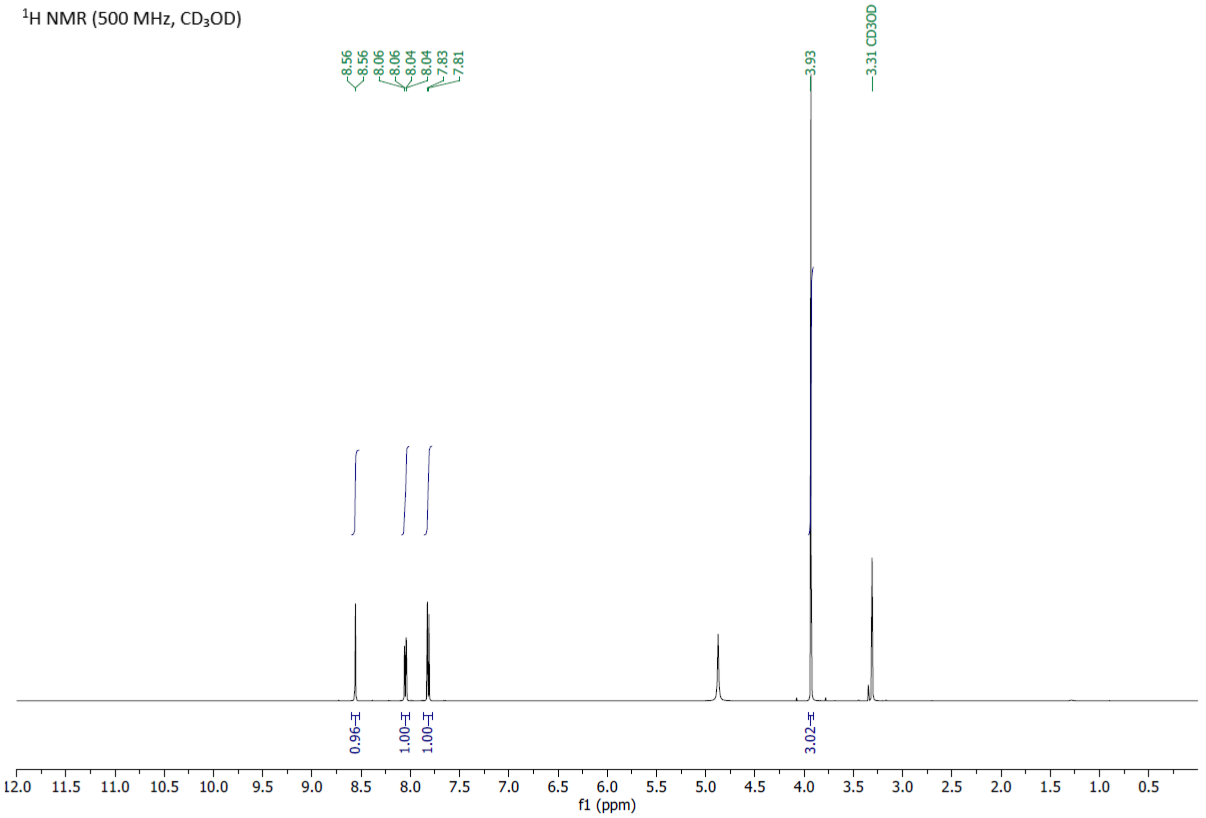

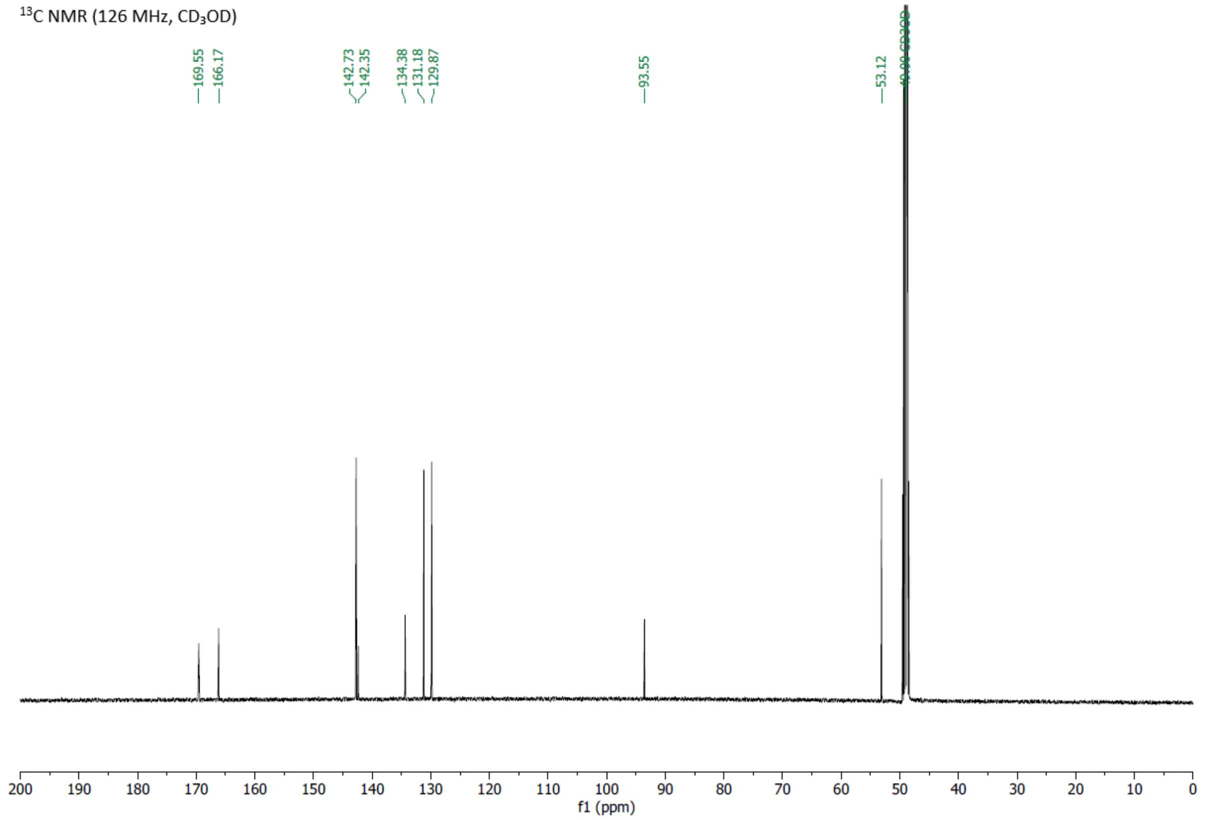

4-acetyl-2-iodobenzoic acid (2r)

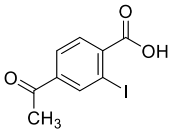

^{1}H NMR (500 MHz, CD_3OD)

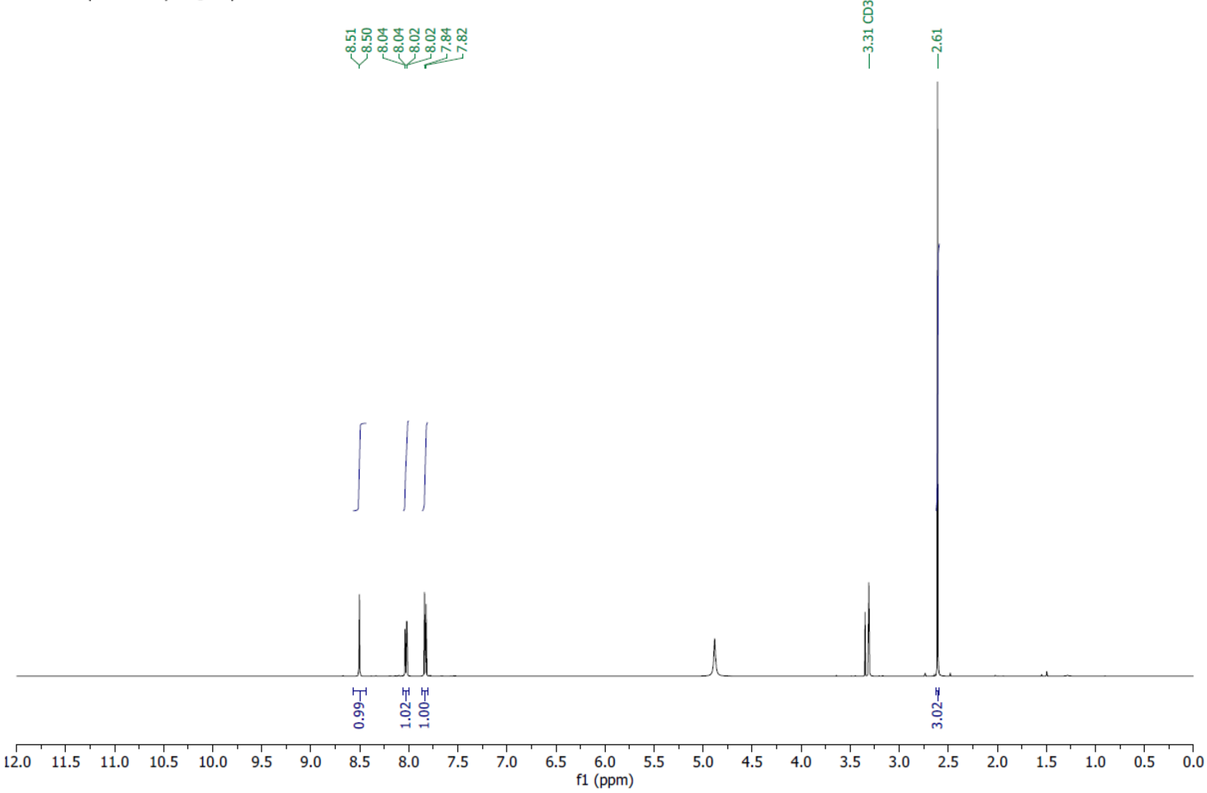

^{13}C NMR (126 MHz, CD_3OD)

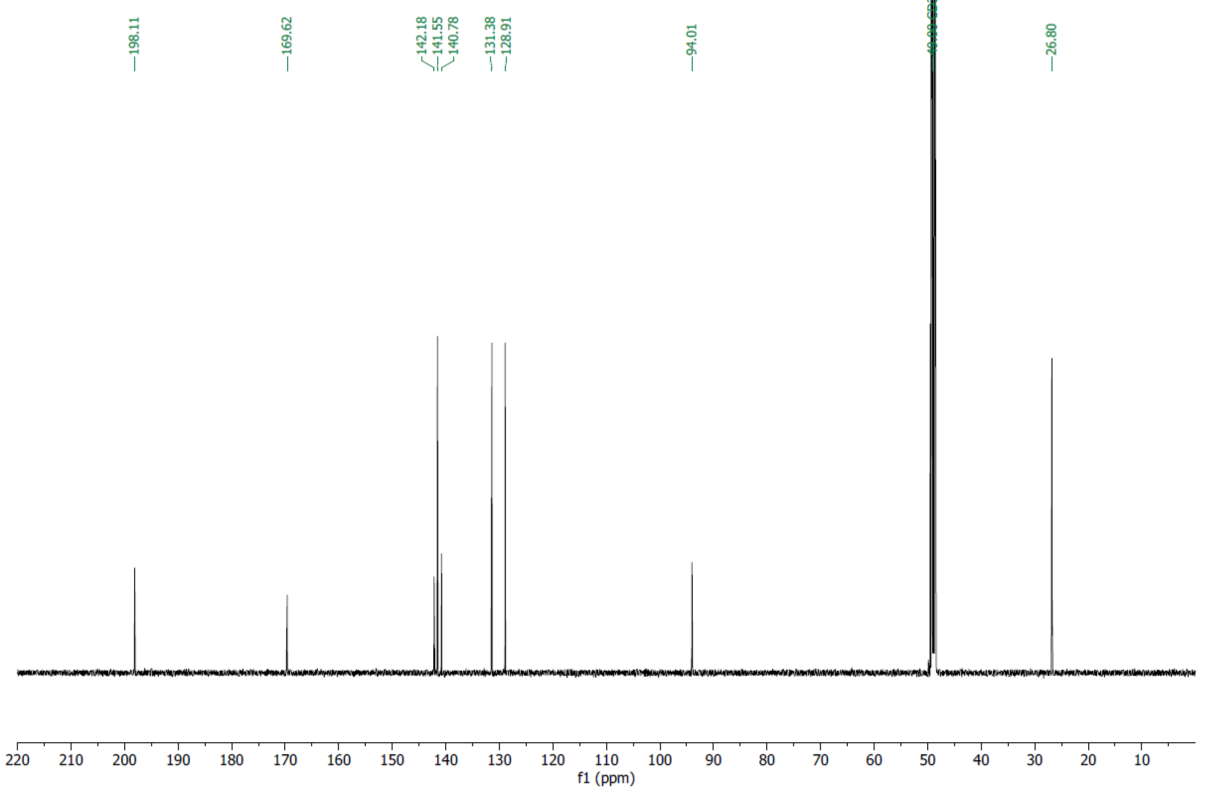

4-carbamoyl-2-iodobenzoic acid (2s)

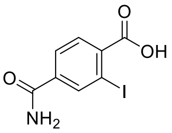

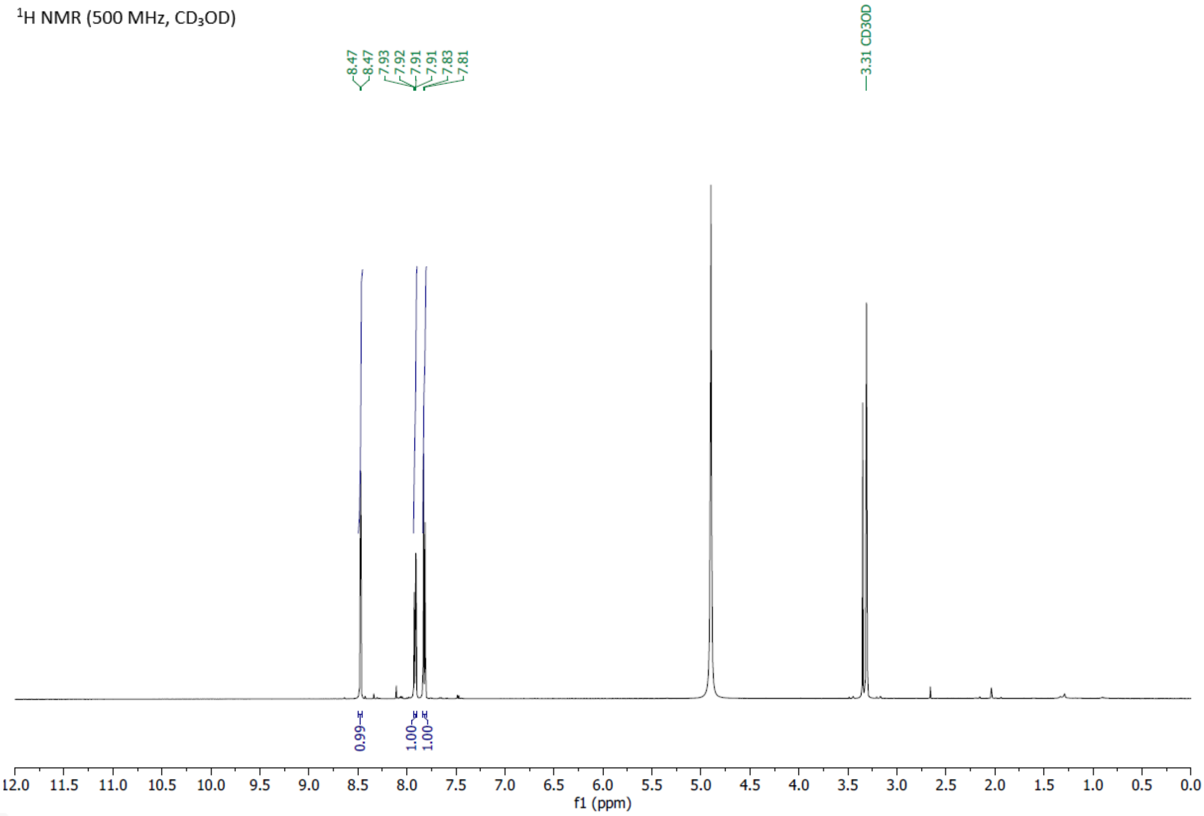

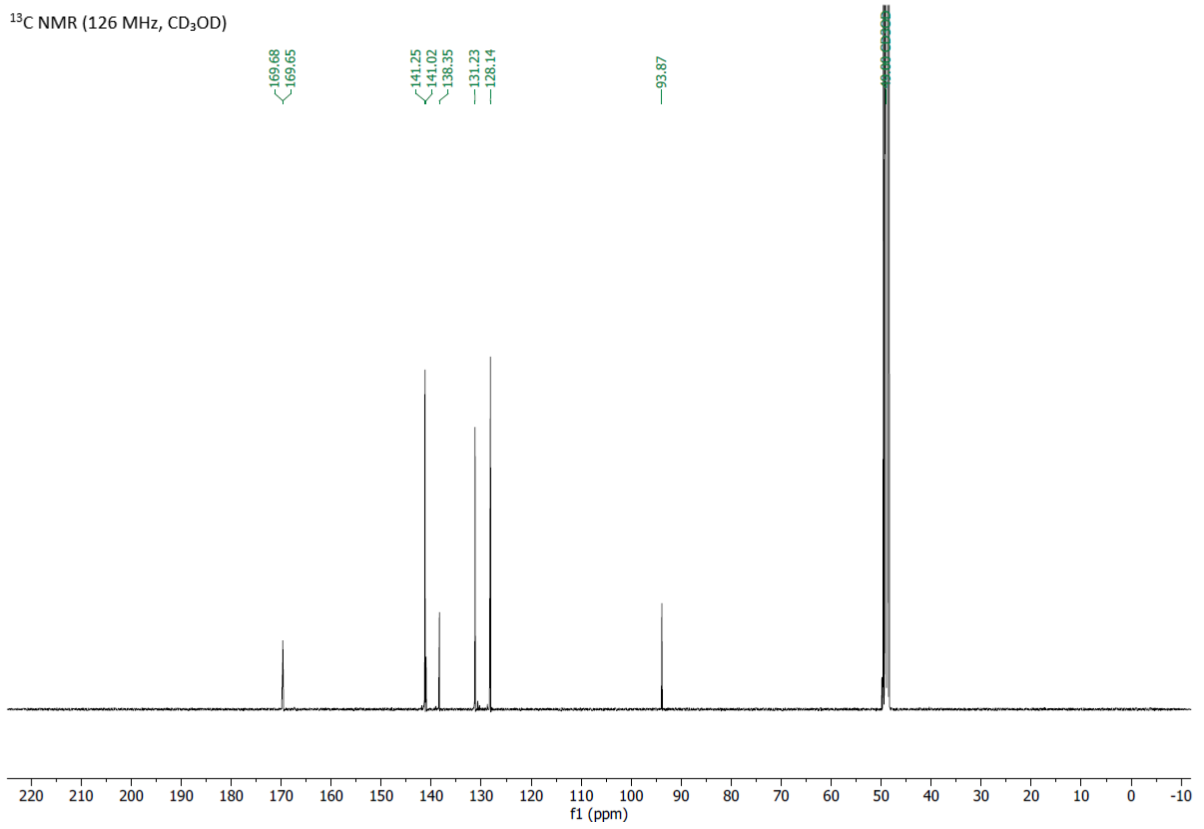

2-iodo-4-(methylcarbamoyl) benzoic acid (2t)

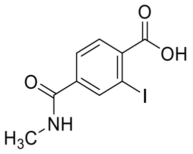

^{1}H NMR (500 MHz, CD$_3$OD)

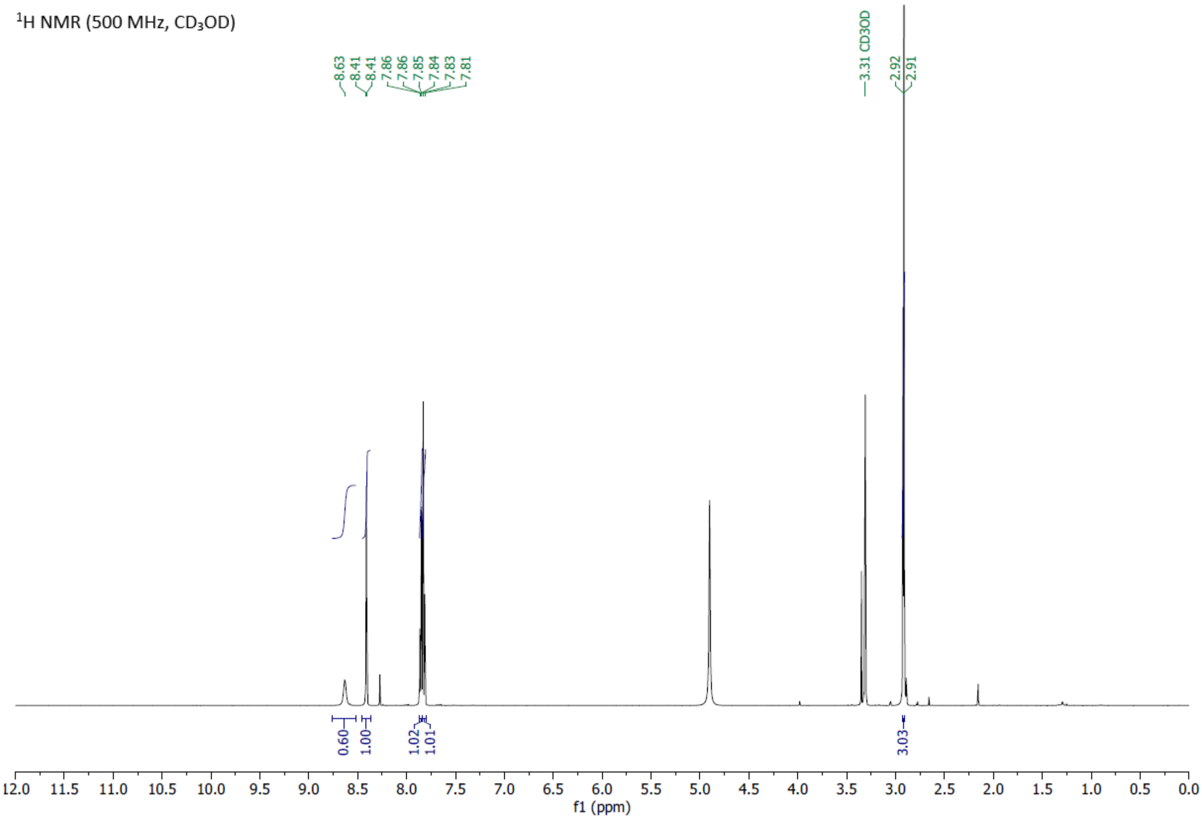

^{13}C NMR (126 MHz, CD$_3$OD)

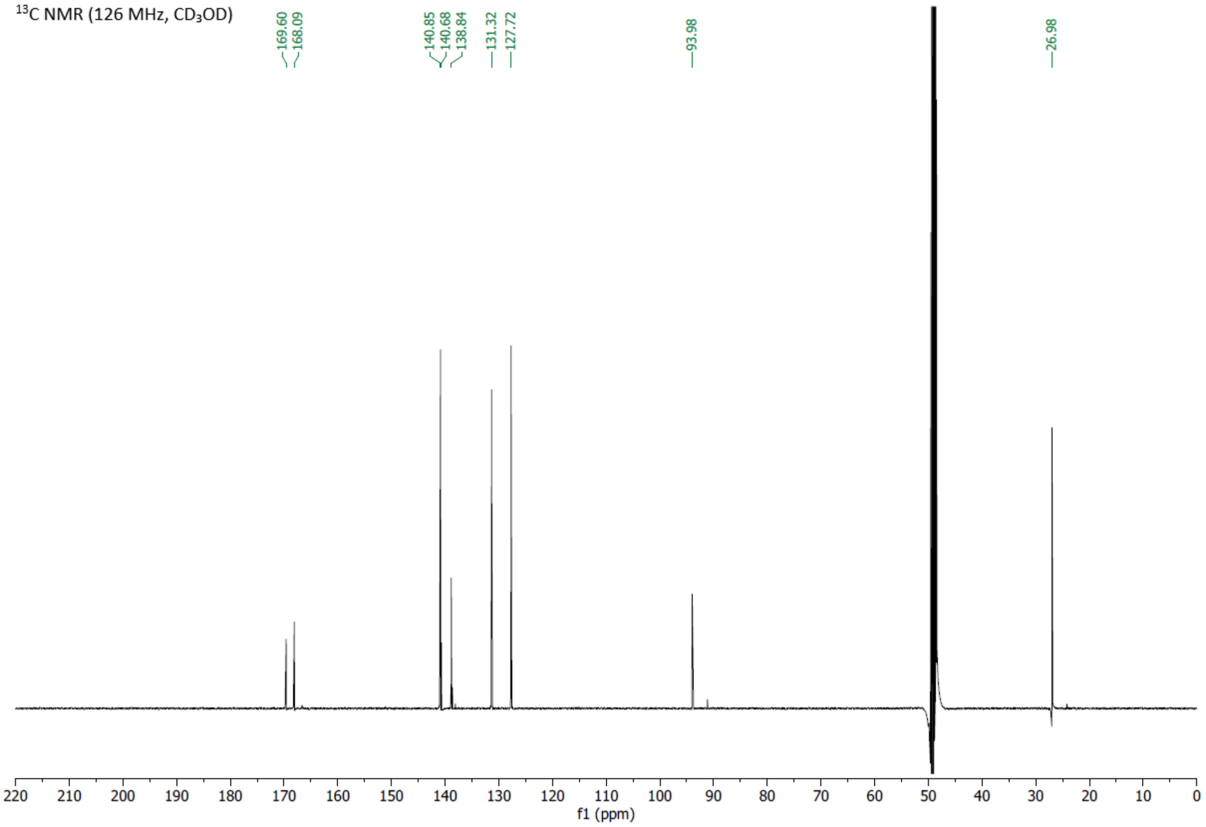

4-(dimethylcarbamoyl)-2-iodobenzoic acid (2u)

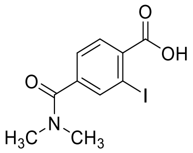

^{1}H NMR (500 MHz, CD_3OD)

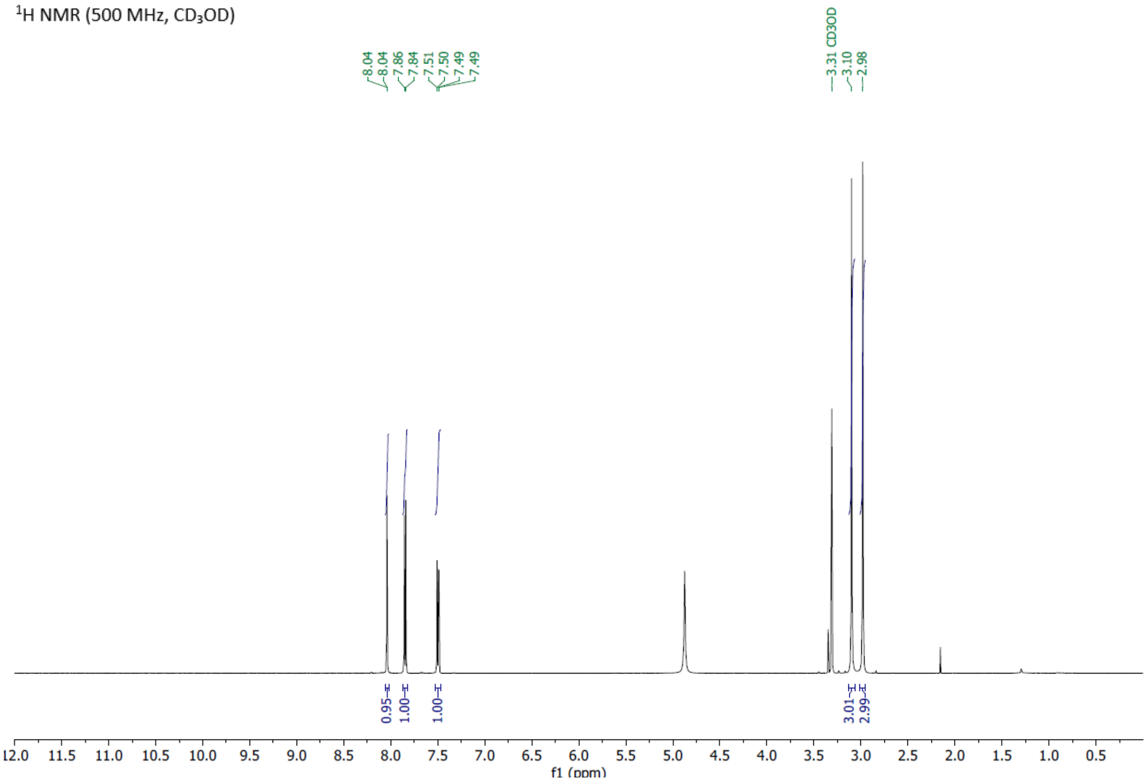

^{13}C NMR (126 MHz, CD_3OD)

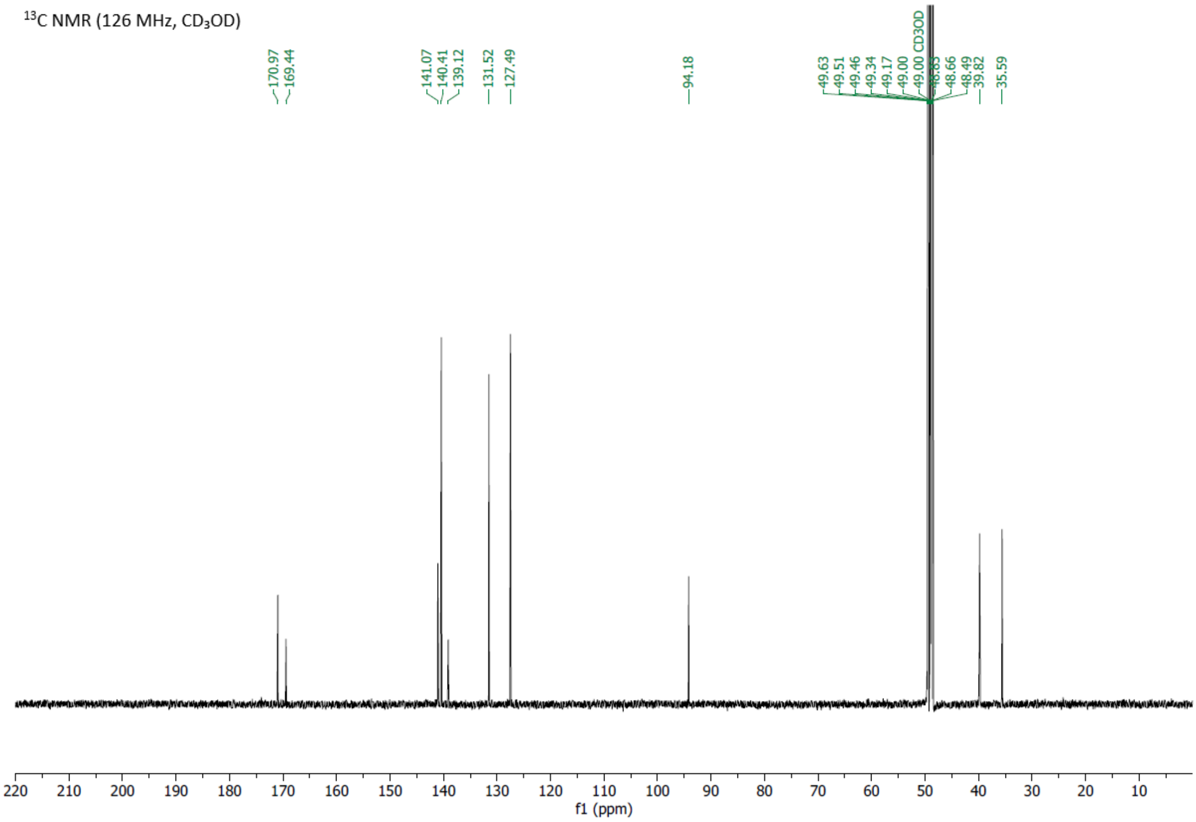

4-(*N*,*N*-dipropylsulfamoyl) -2-iodobenzoic acid (2v)

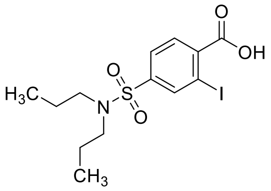

^{1}H NMR (500 MHz, CD_3OD)

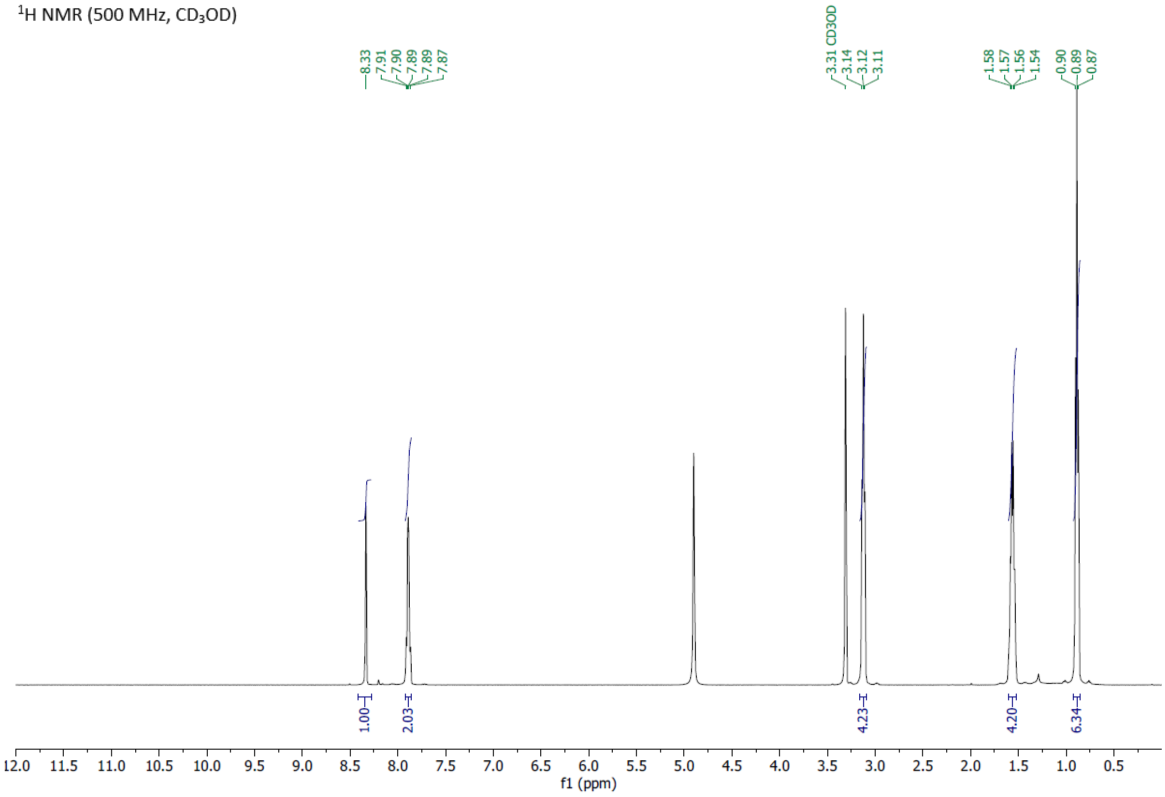

^{13}C NMR (126 MHz, CD_3OD)

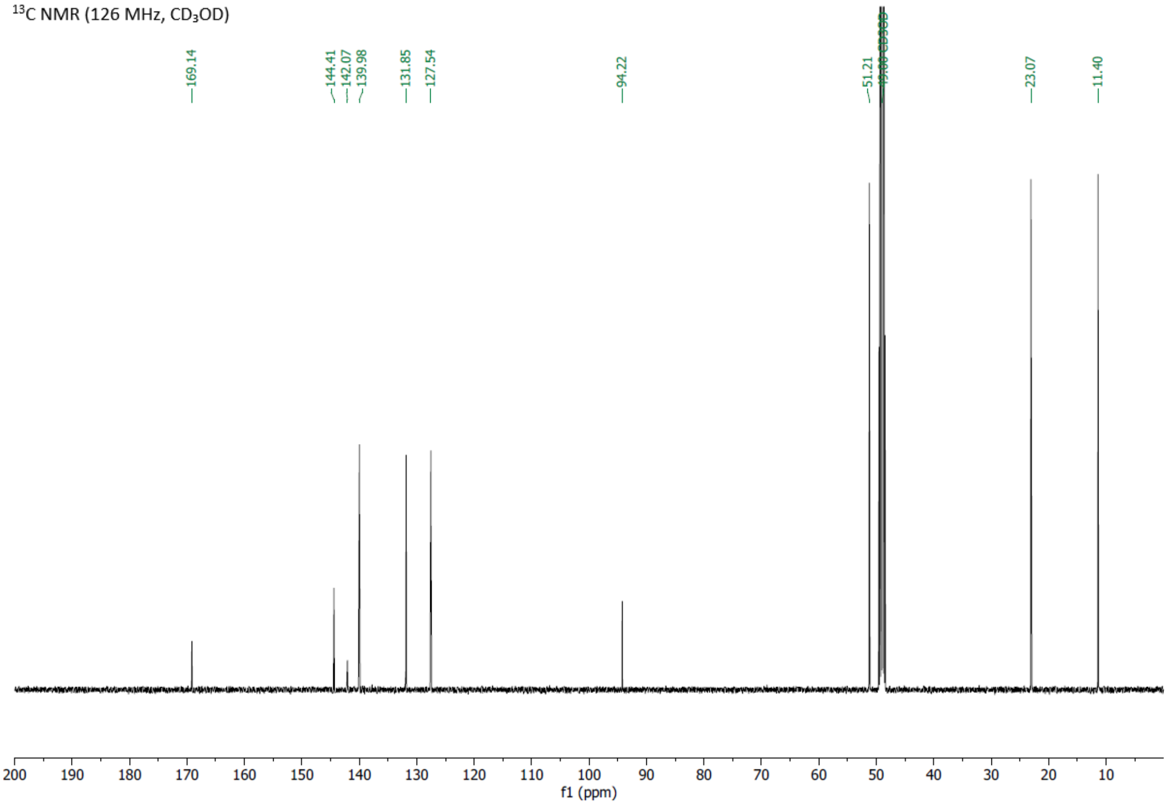

2-iodo-5-methylbenzoic acid (2w)

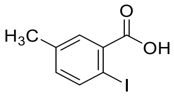

^1H NMR (500 MHz, CD_3OD)

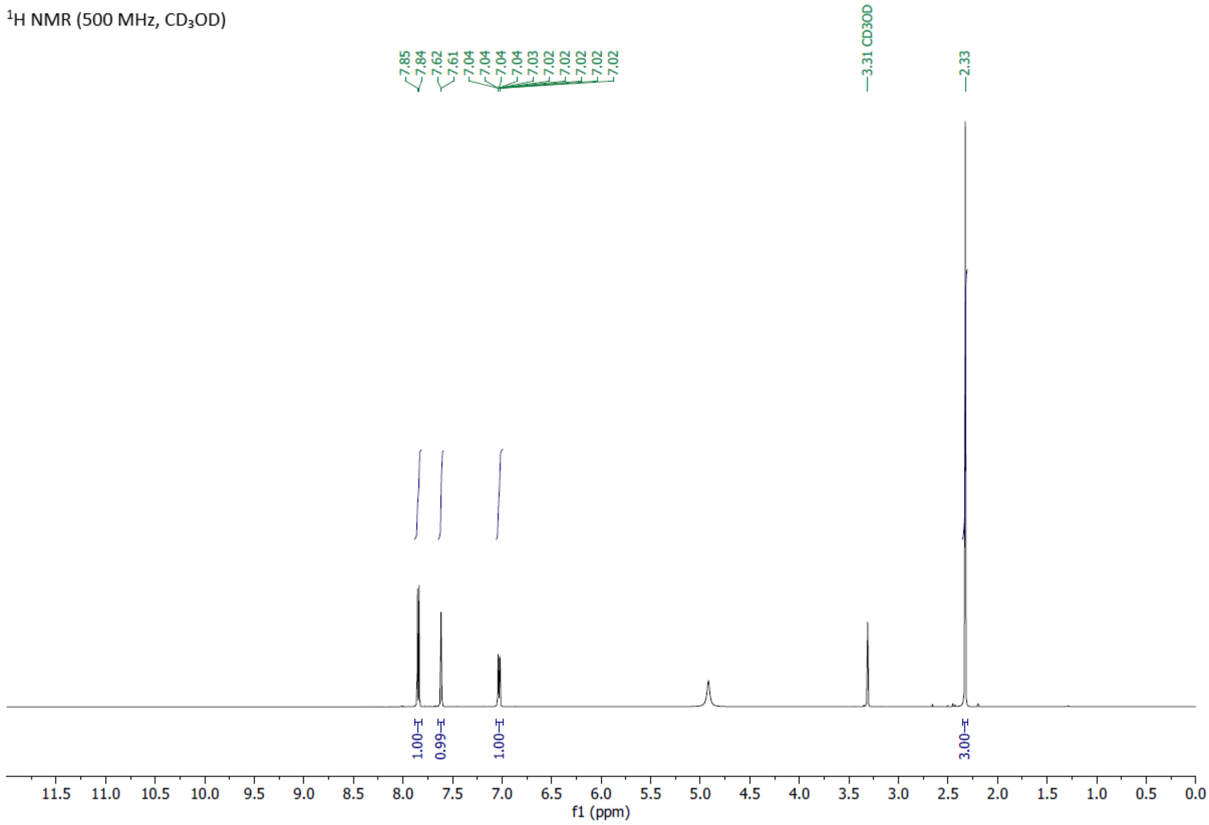

^{13}C NMR (126 MHz, CD_3OD)

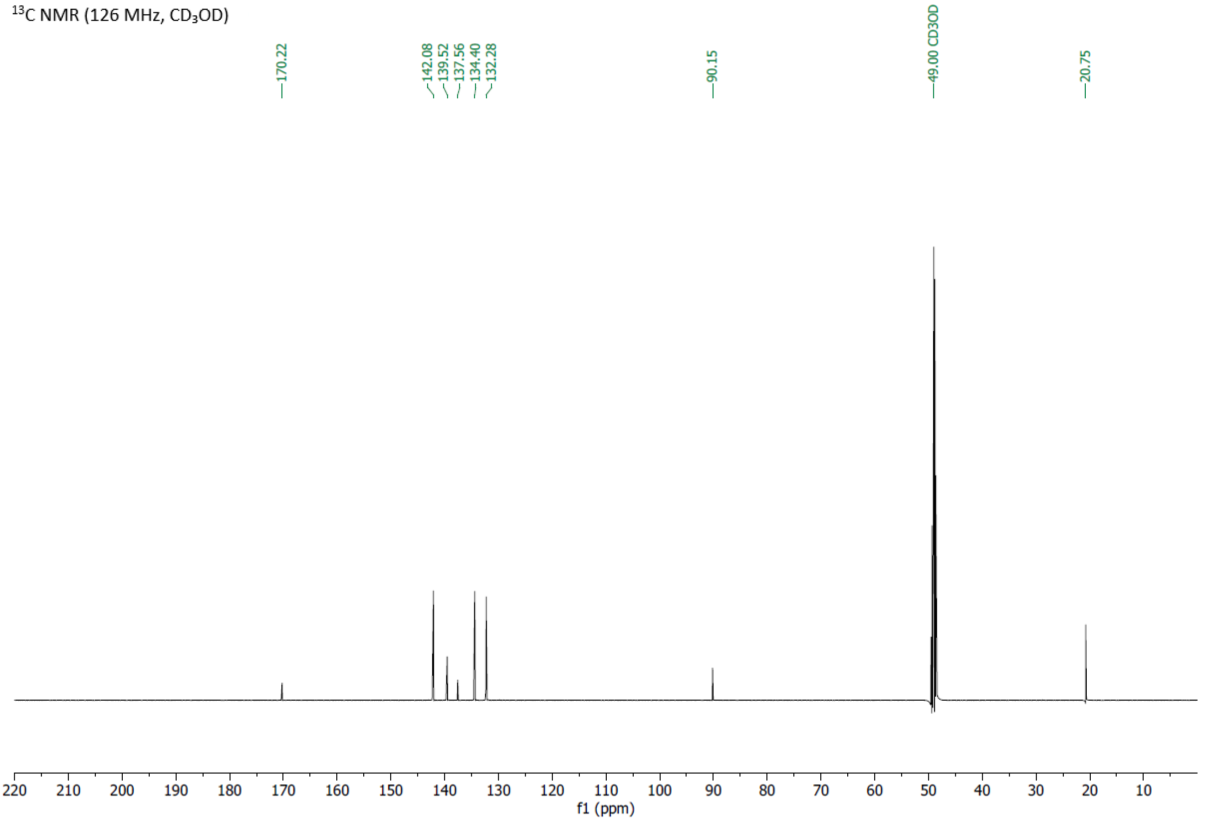

2-iodo-5-(trifluoromethyl) benzoic acid (2x)

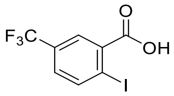

1H NMR (500 MHz, CDCl₃)

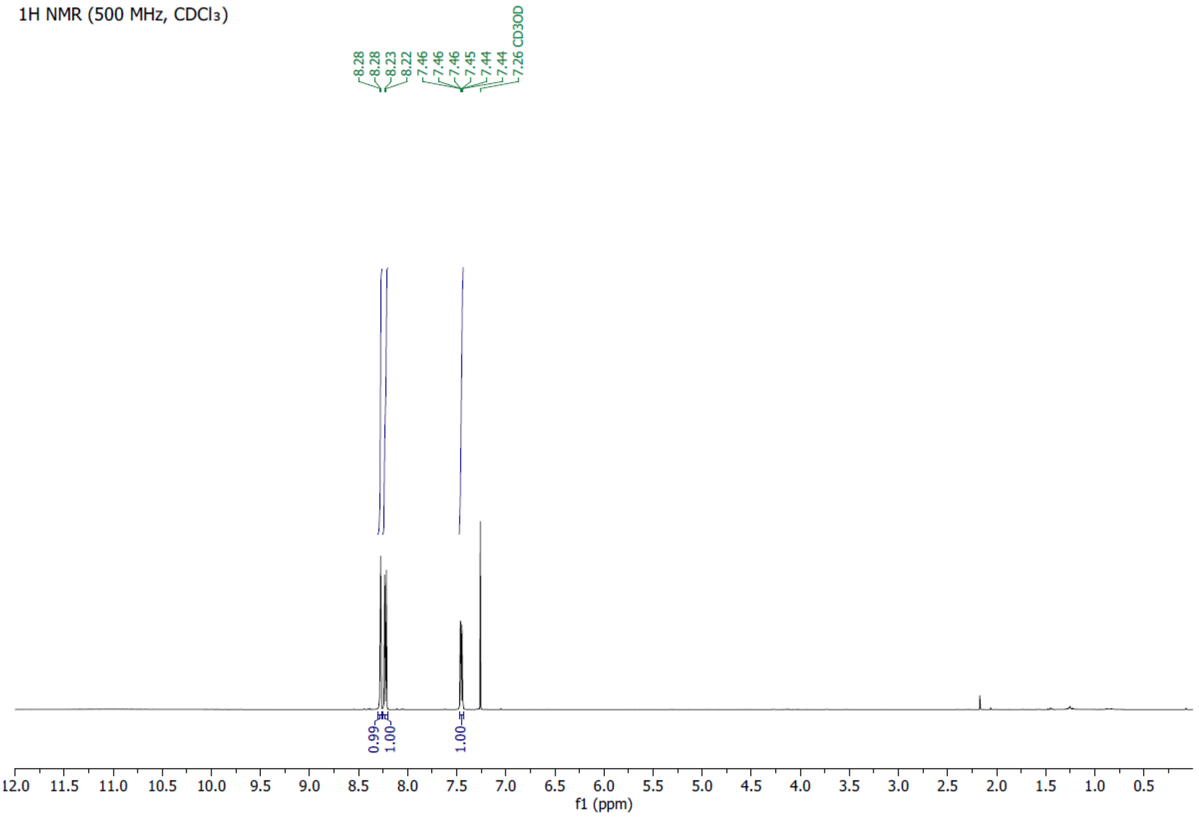

13C NMR (126 MHz, CDCl3)

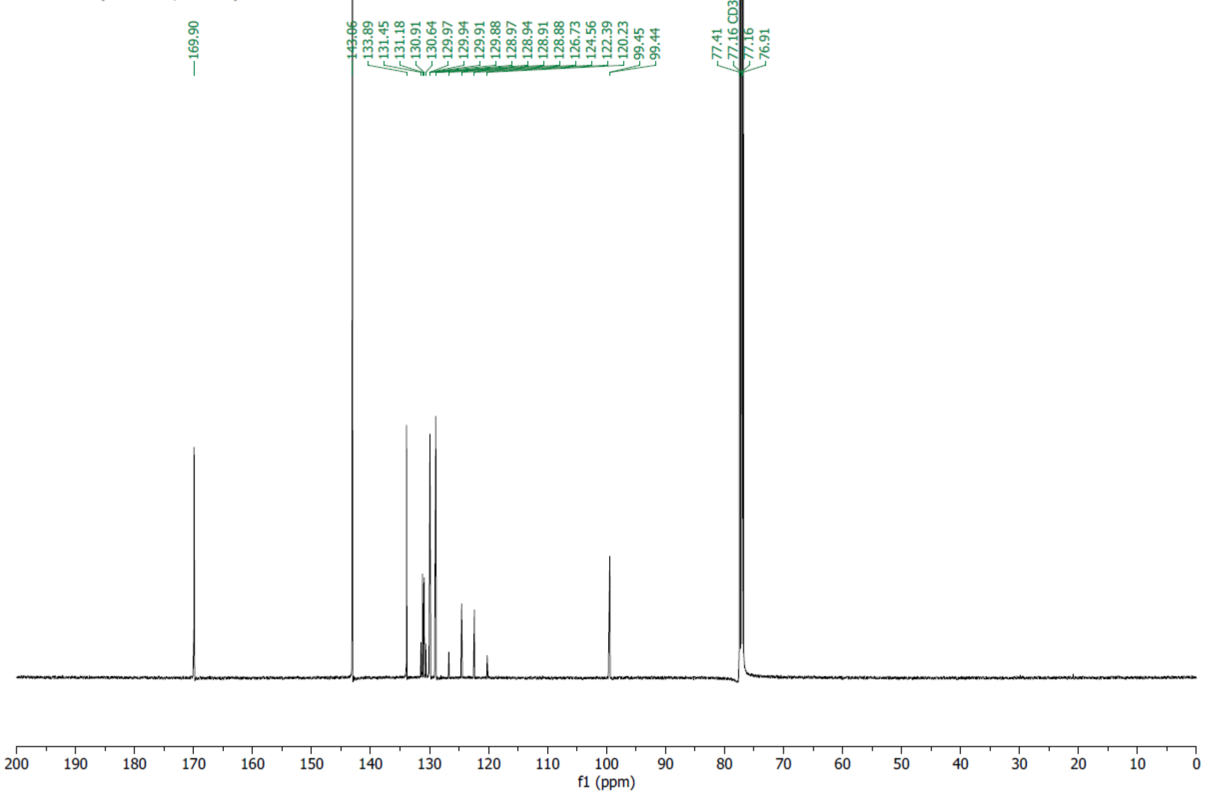

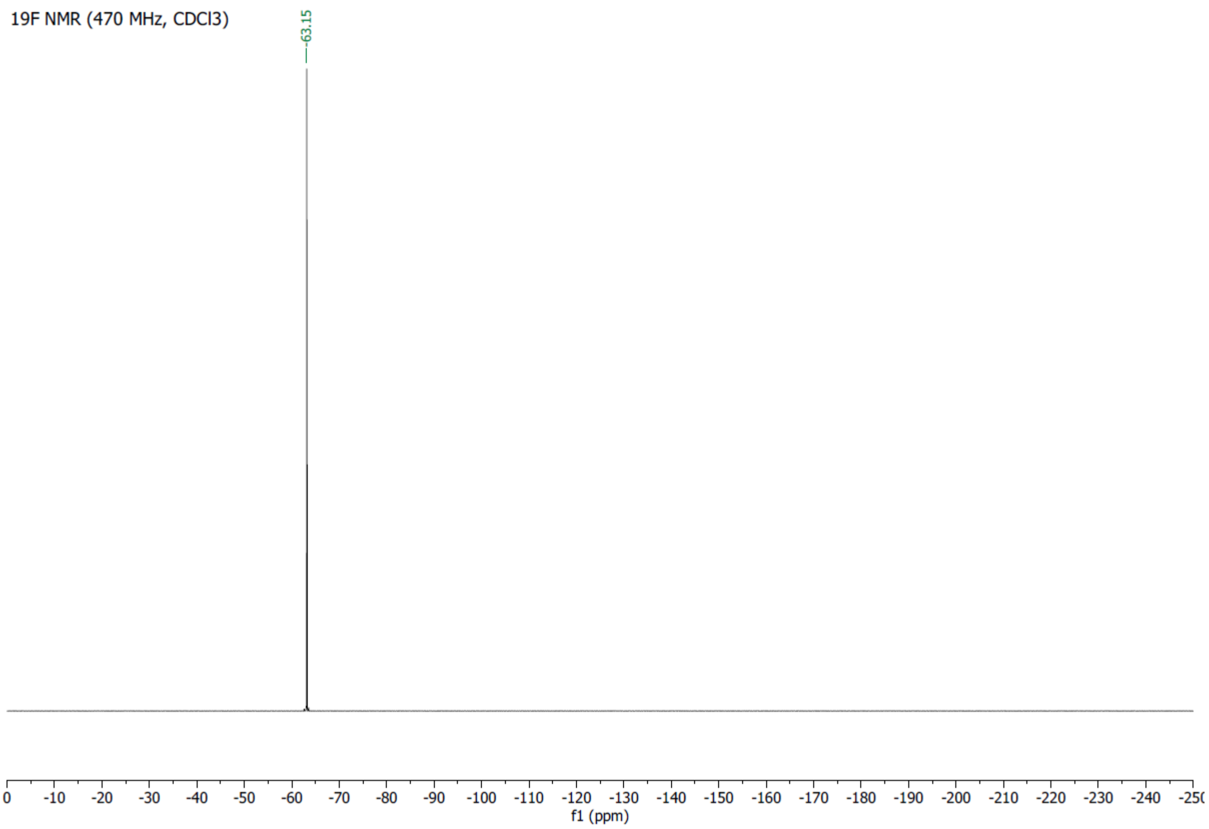

19F NMR (470 MHz, CDCl3)
-63.15
0 -10 -20 -30 -40 -50 -60 -70 -80 -90 -100 -110 -120 -130 -140 -150 -160 -170 -180 -190 -200 -210 -220 -230 -240 -250
f1 (ppm)

5-(difluoromethyl)-2-iodobenzoic acid (2y)

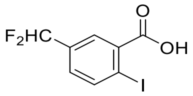

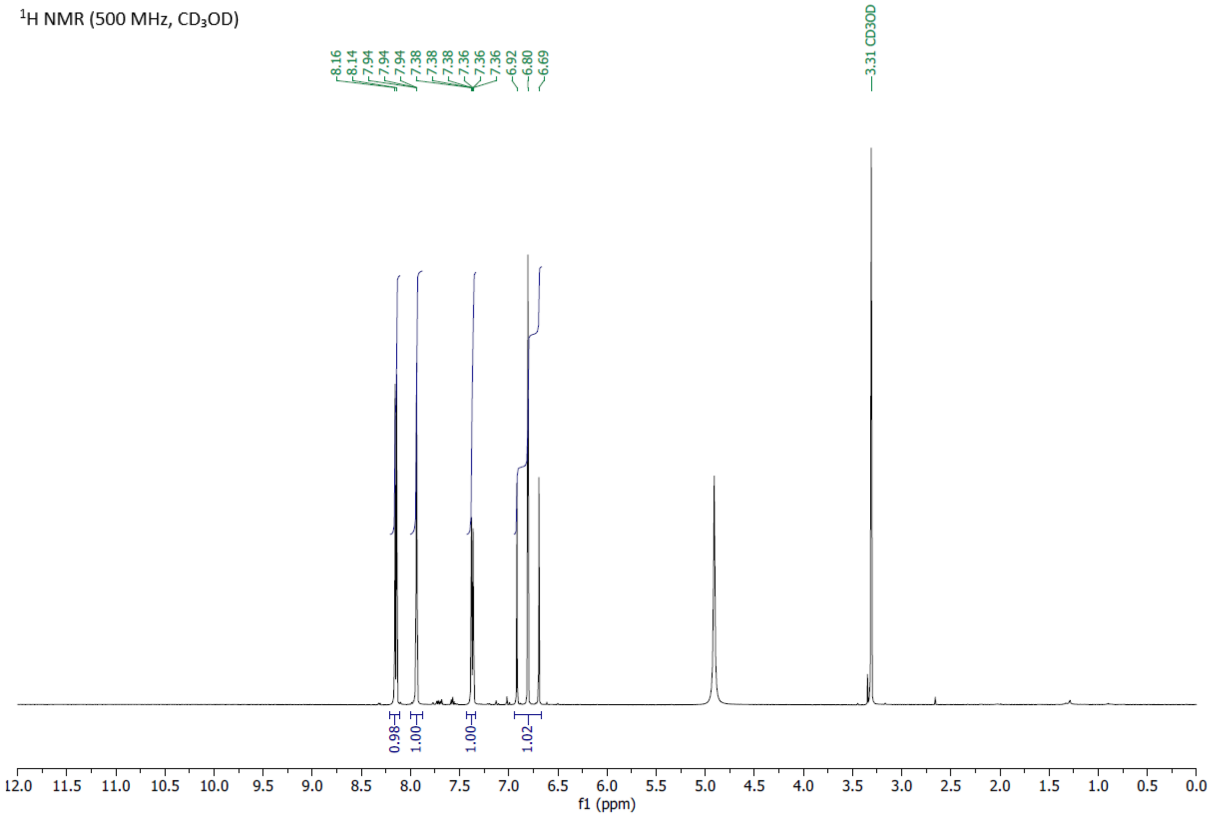

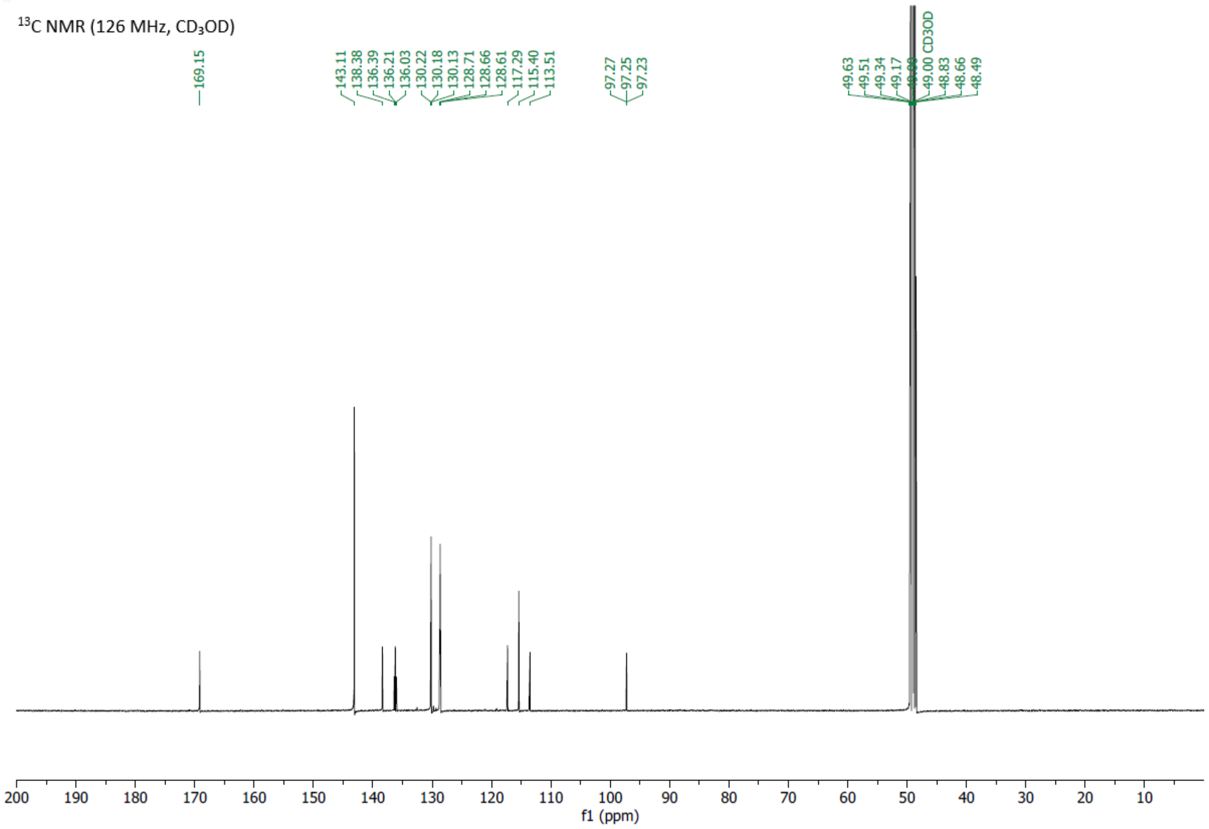

^{19}F NMR (470 MHz, CD_3OD)

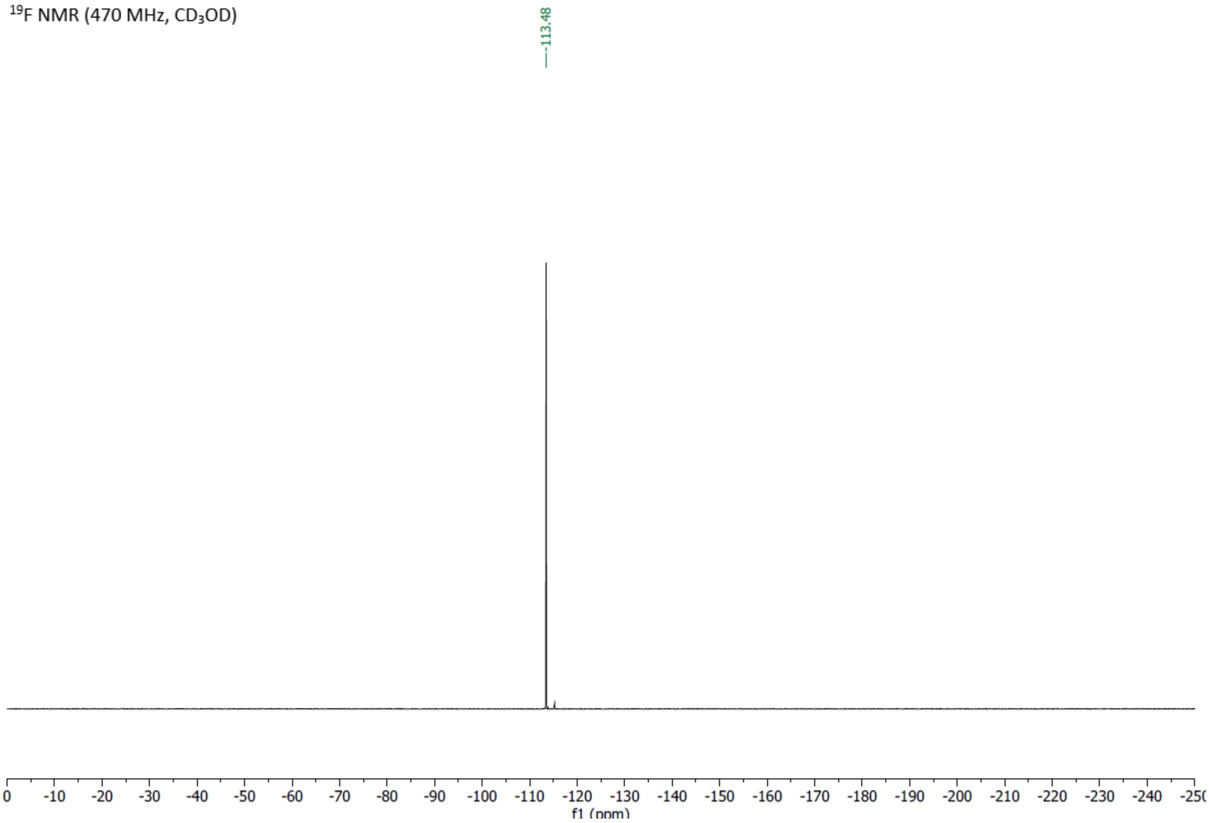

5-acetyl-2-iodobenzoic acid (2z)

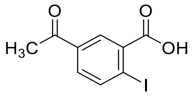

^1H NMR (500 MHz, CD_3OD)

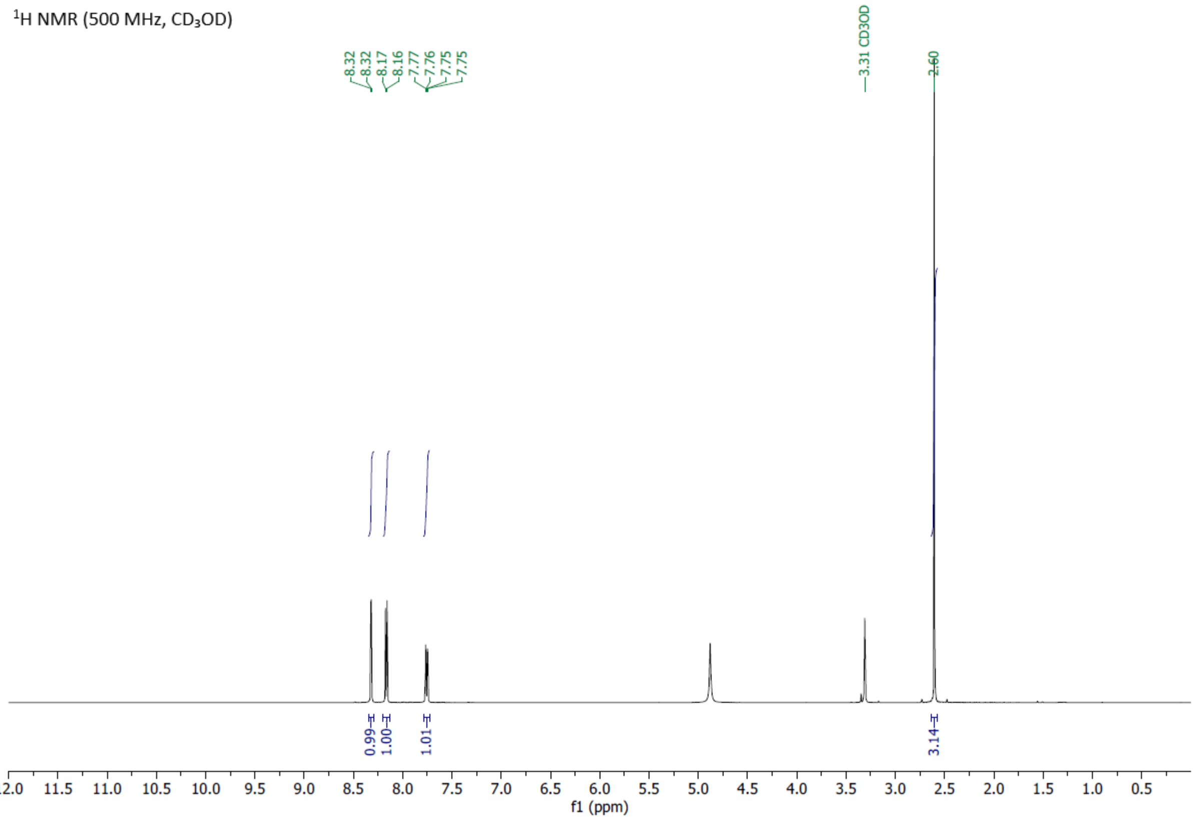

13C NMR (126 MHz, CD3OD)

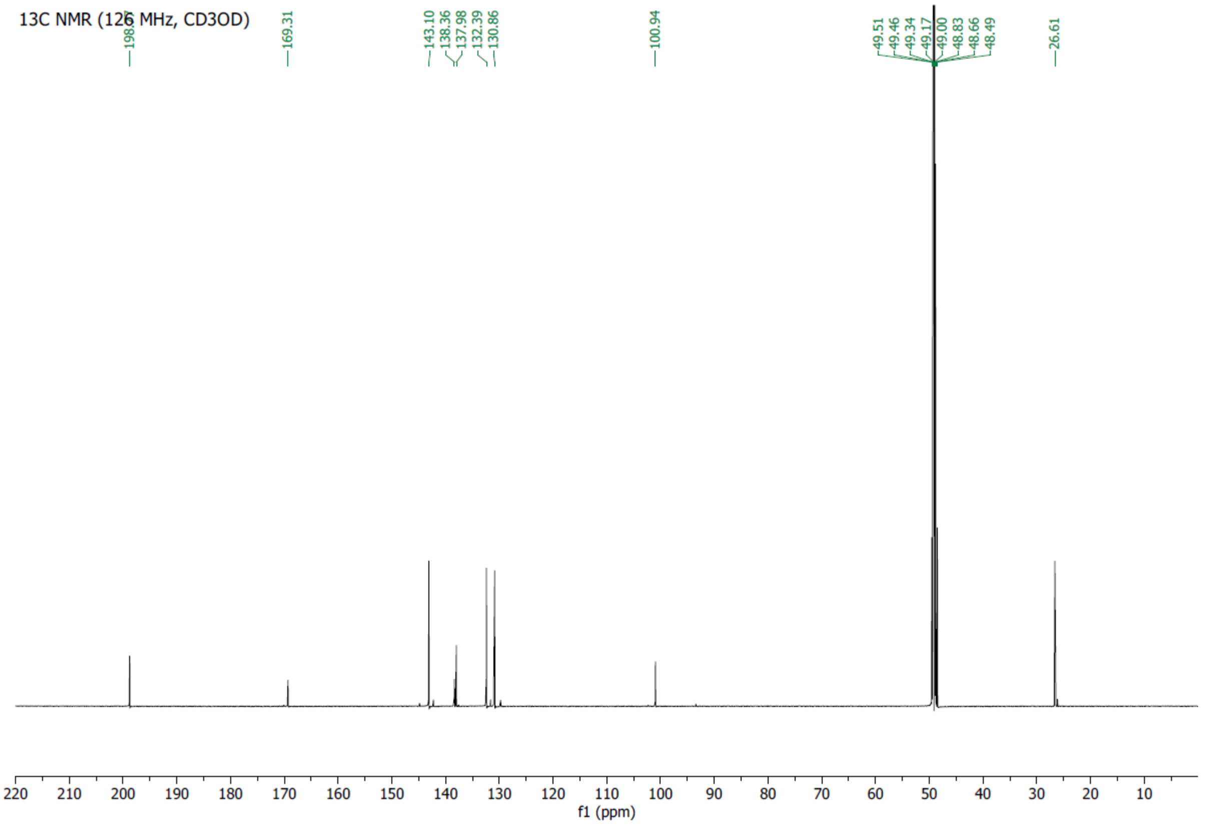

2-iodo-5-(methoxycarbonyl) benzoic acid (2aa)

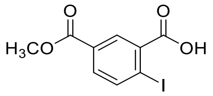

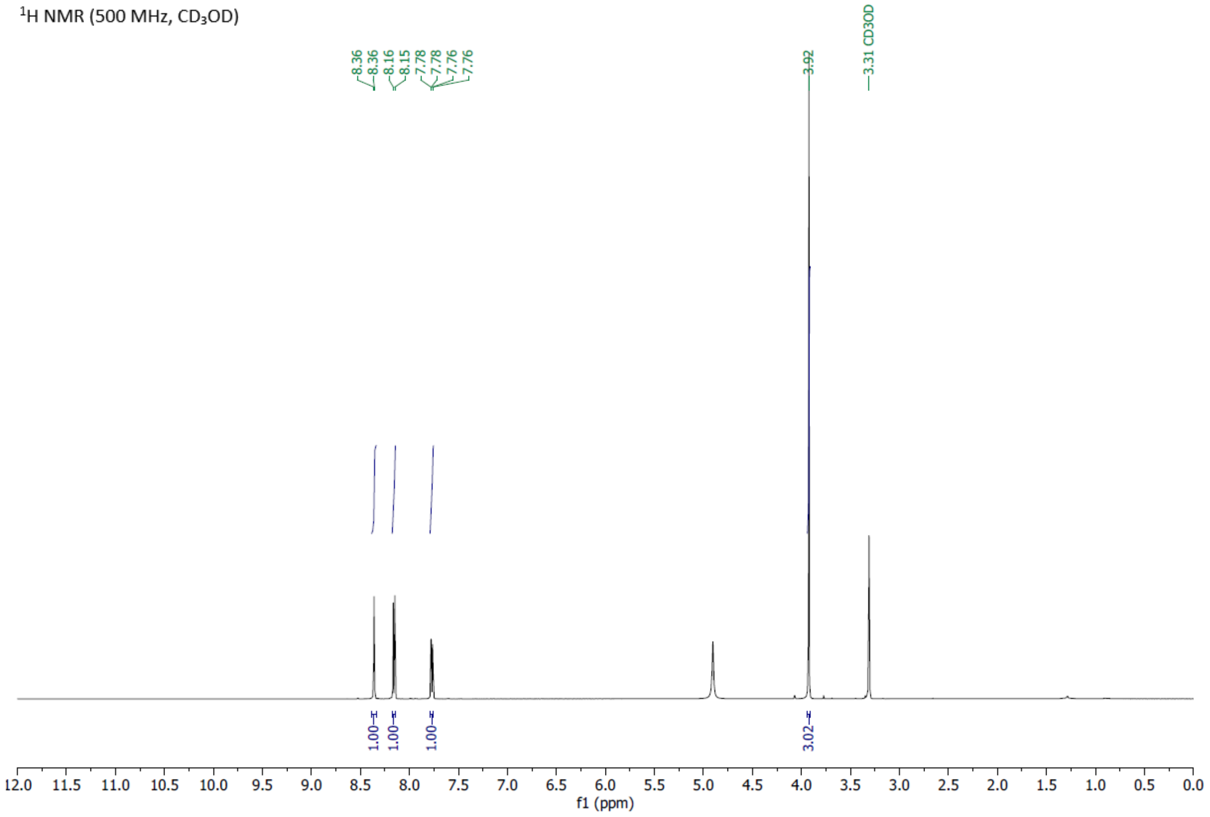

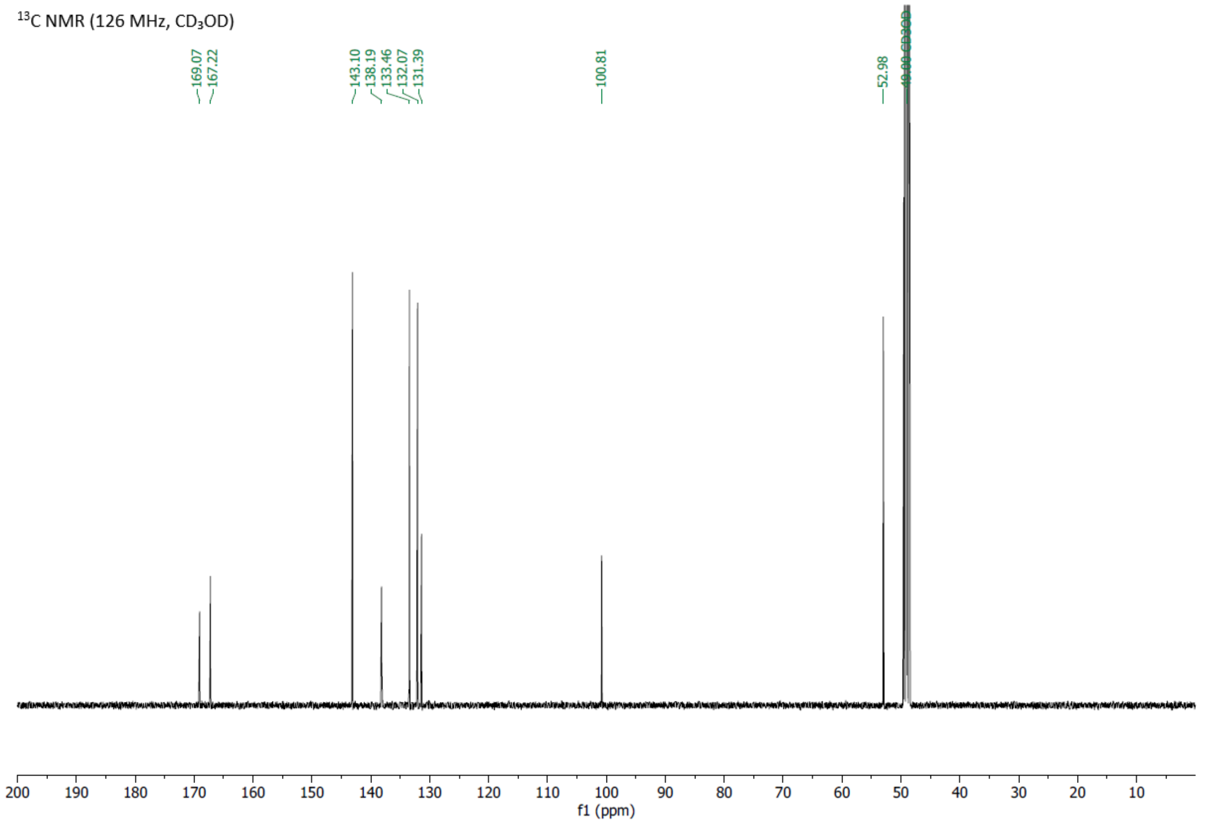

3-fluoro-2-iodobenzoic acid (2ab)

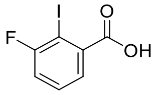

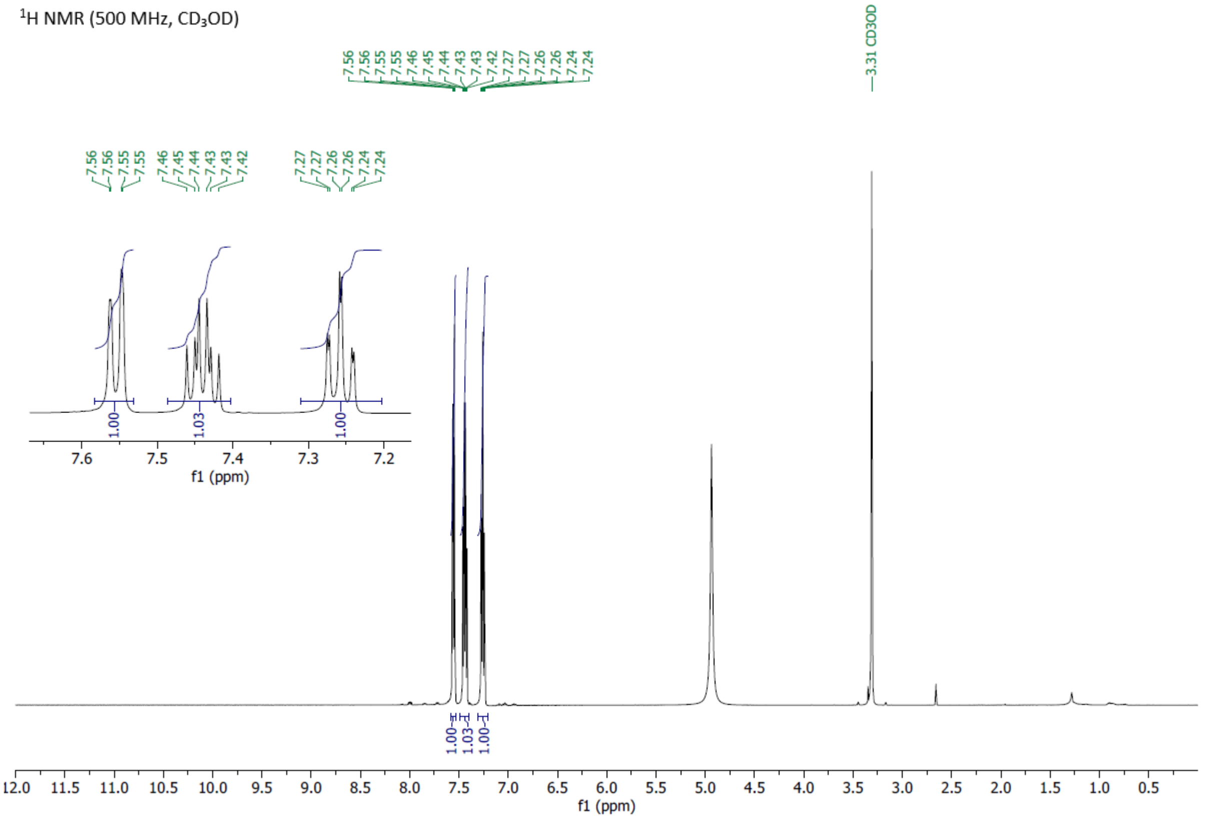

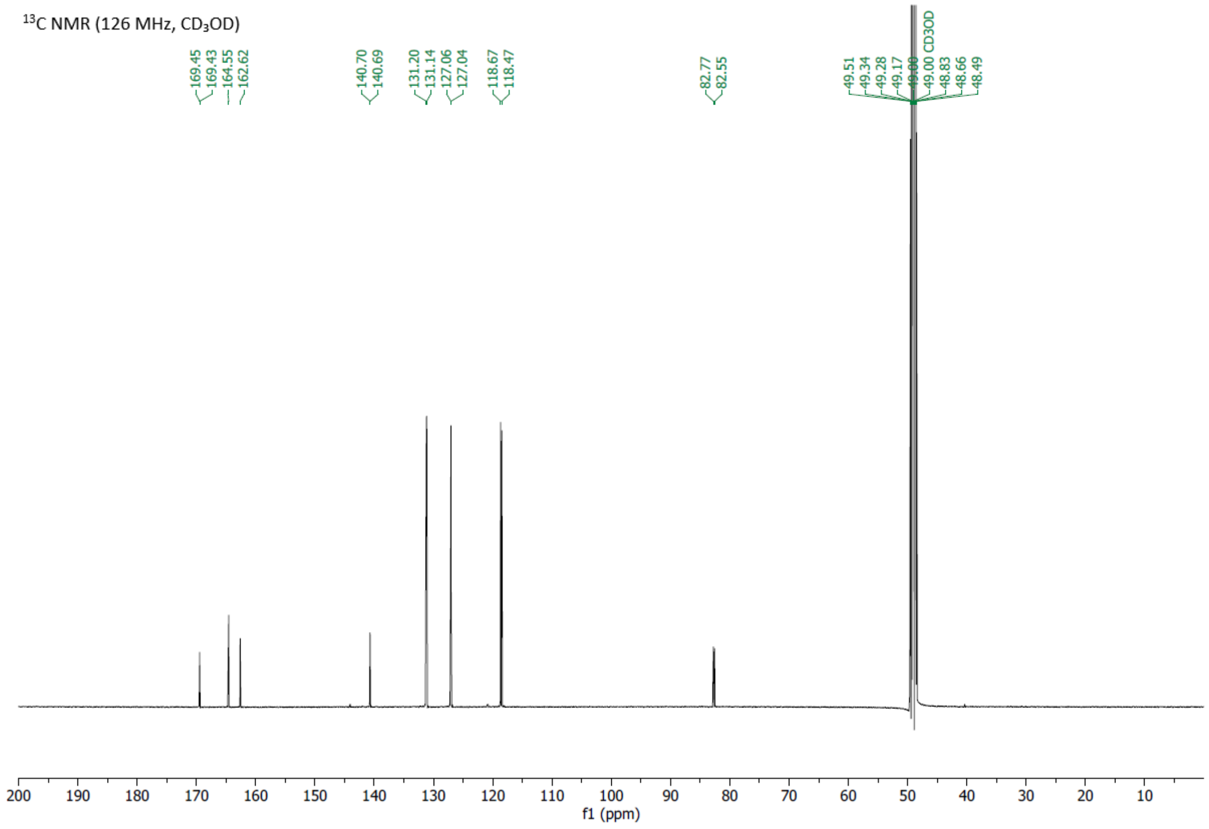

^{19}F NMR (470 MHz, CD_3OD)

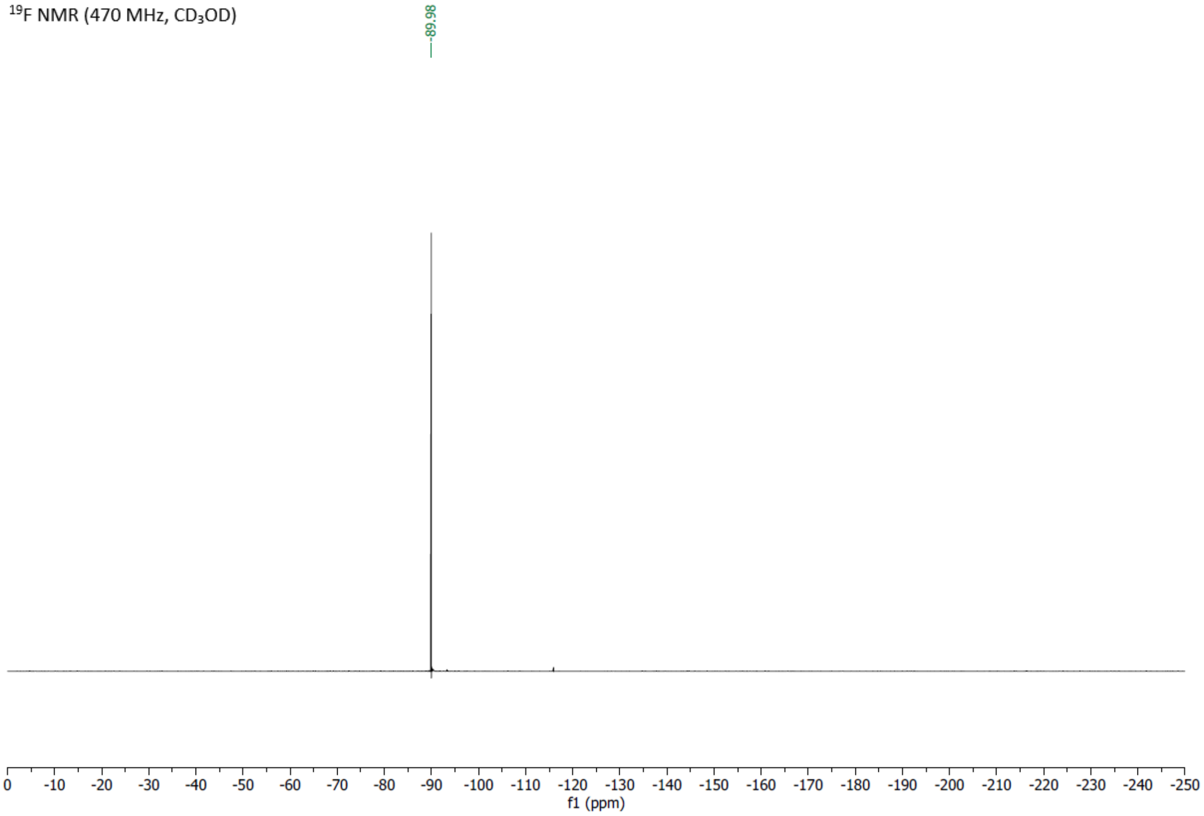

3-chloro-2-iodobenzoic acid (2ac)

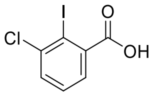

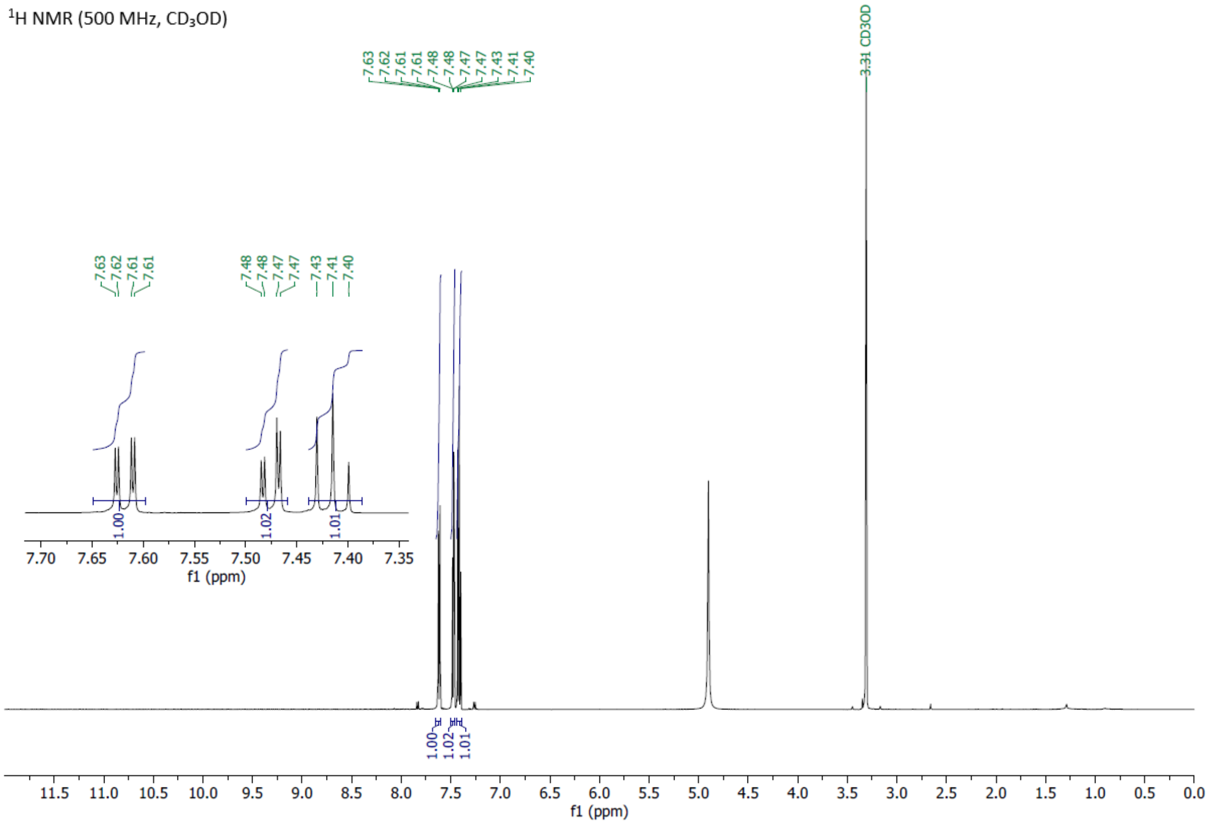

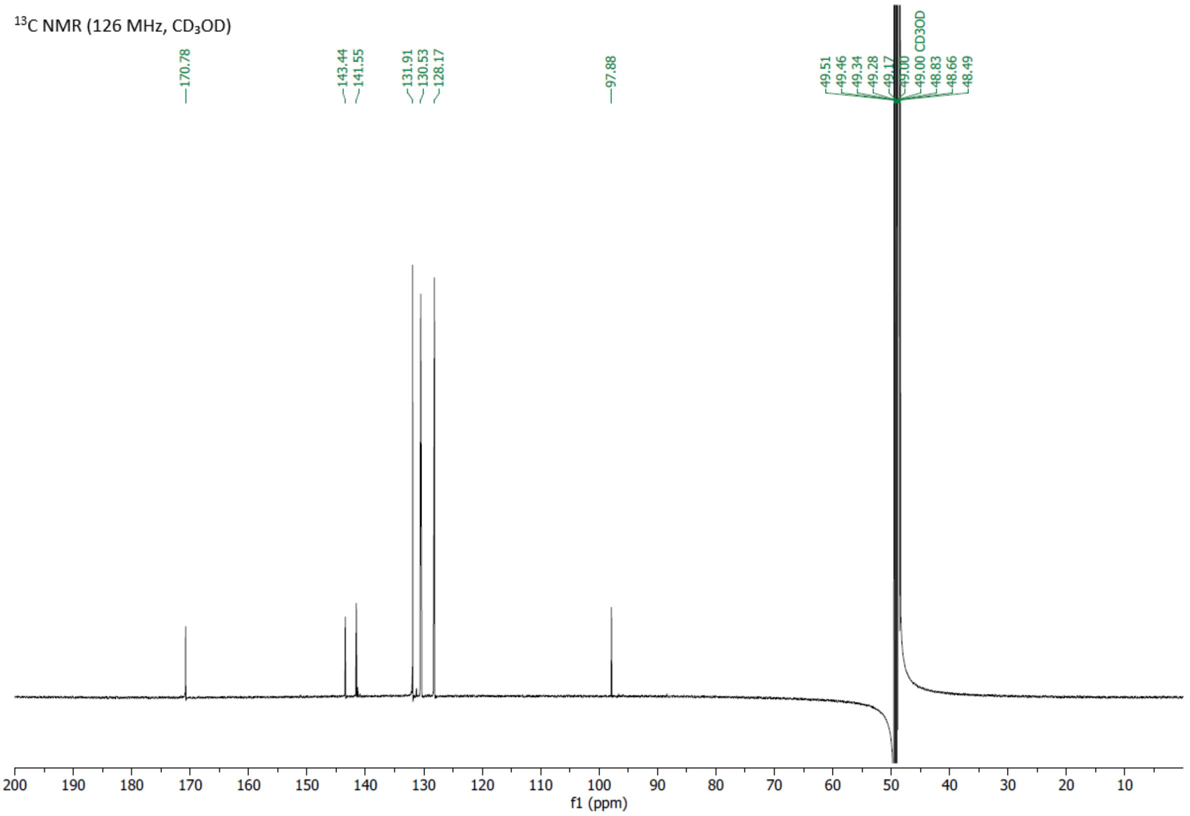

5-chloro-2-iodobenzoic acid (2ad)

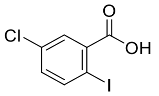

^{1}H NMR (500 MHz, CD_3OD)

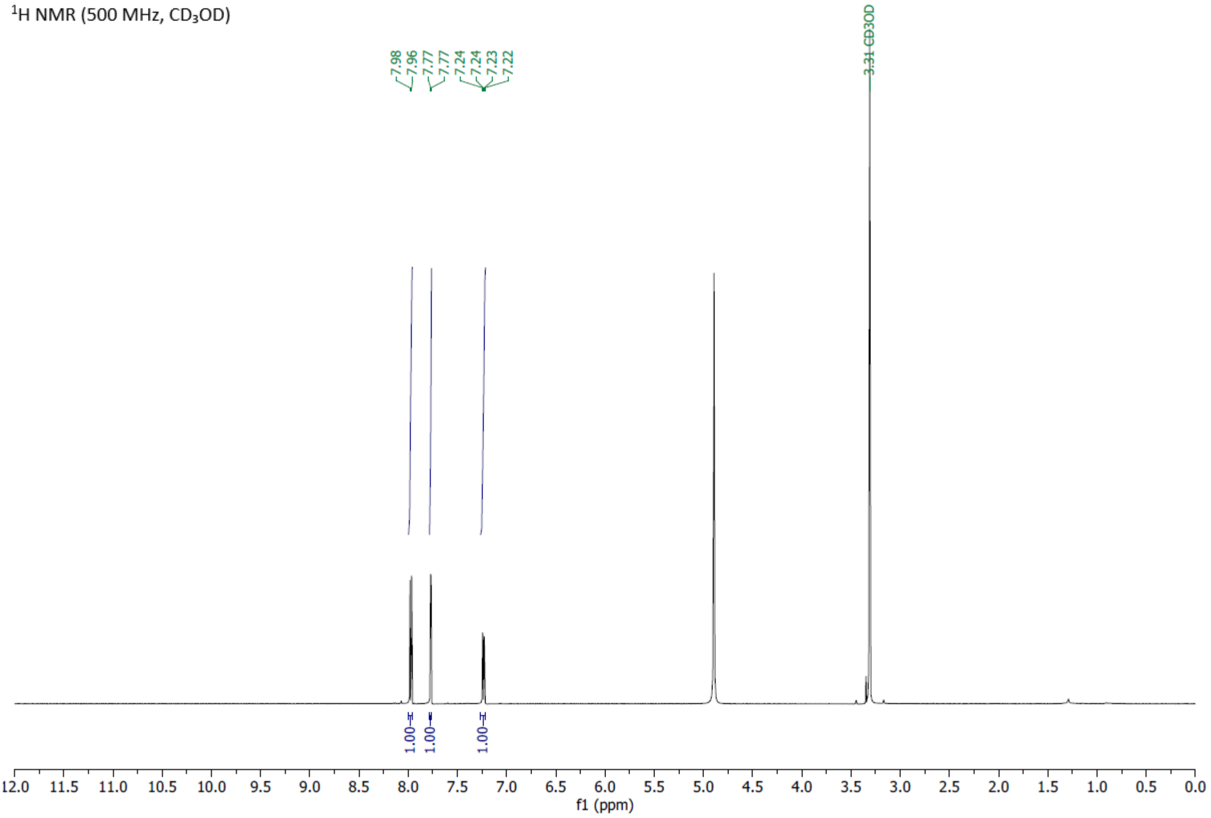

^{13}C NMR (126 MHz, CD_3OD)

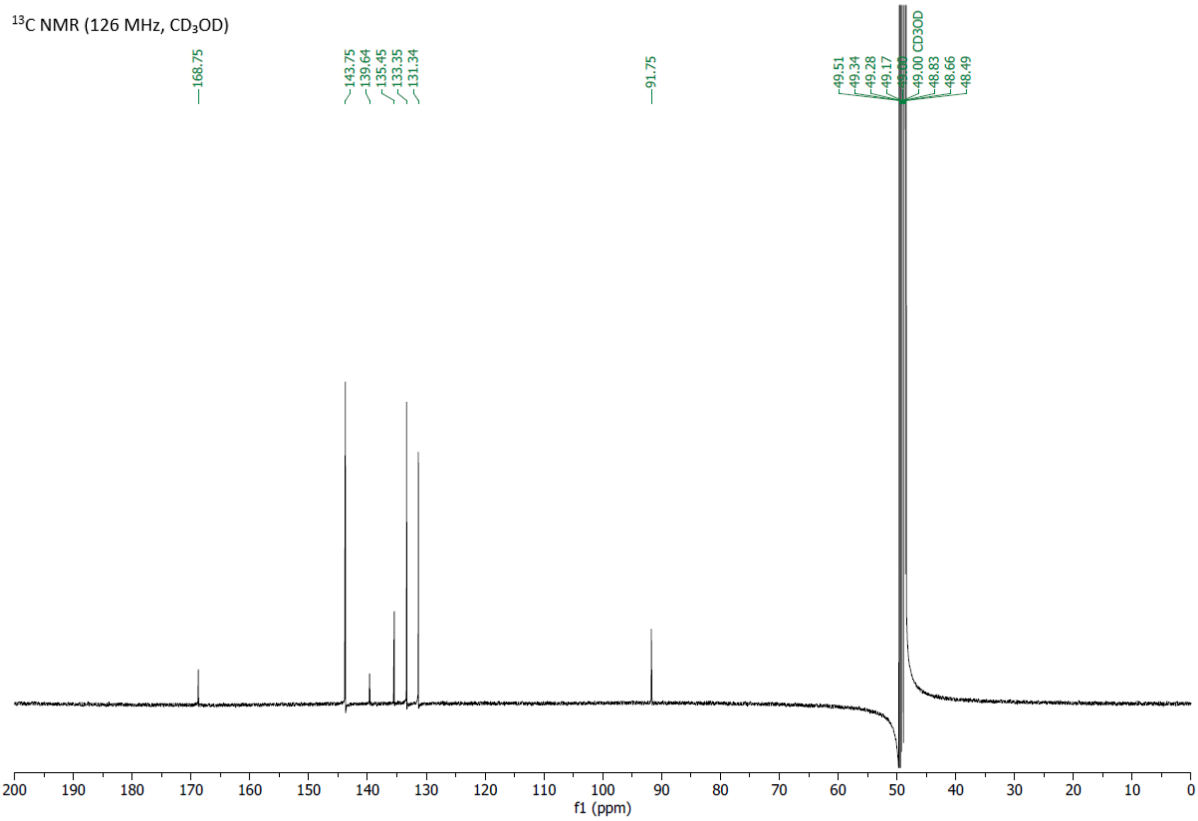

3-bromo-2-iodobenzoic acid (2ae)

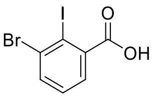

^{1}H NMR (500 MHz, CD_3OD)

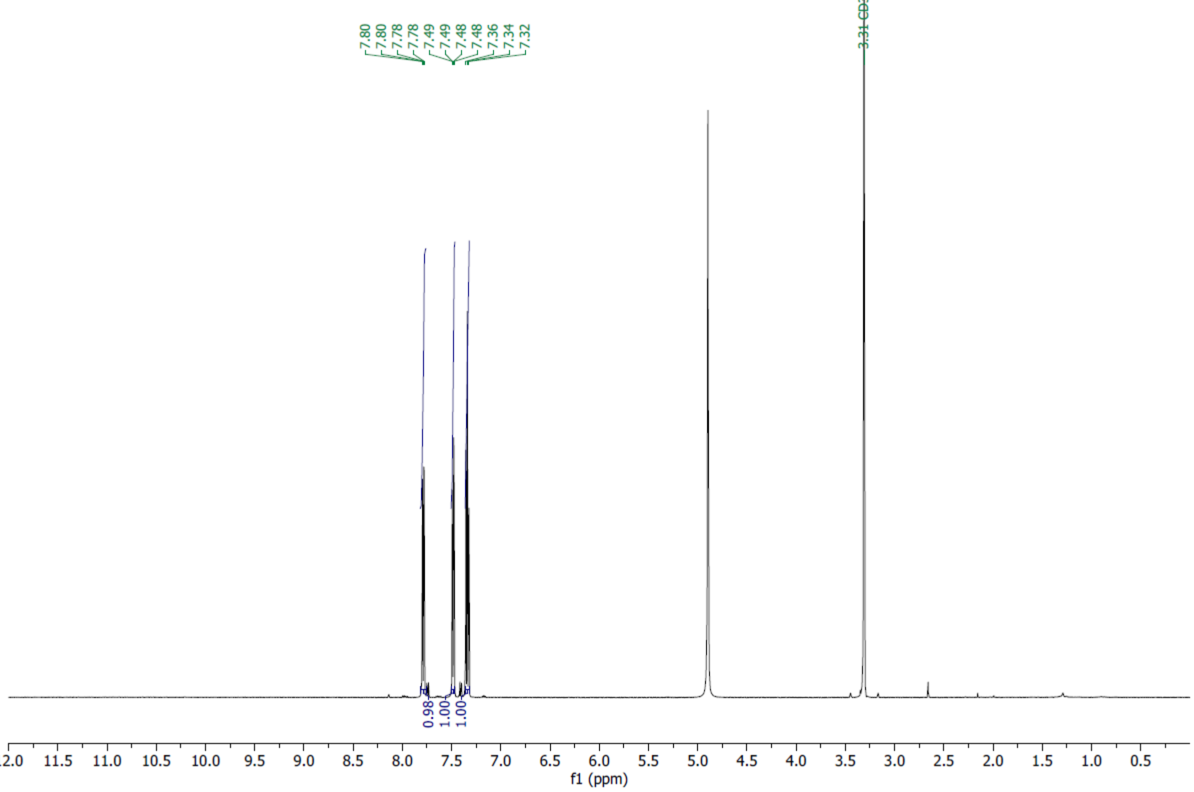

13C NMR (126 MHz, CD3OD)

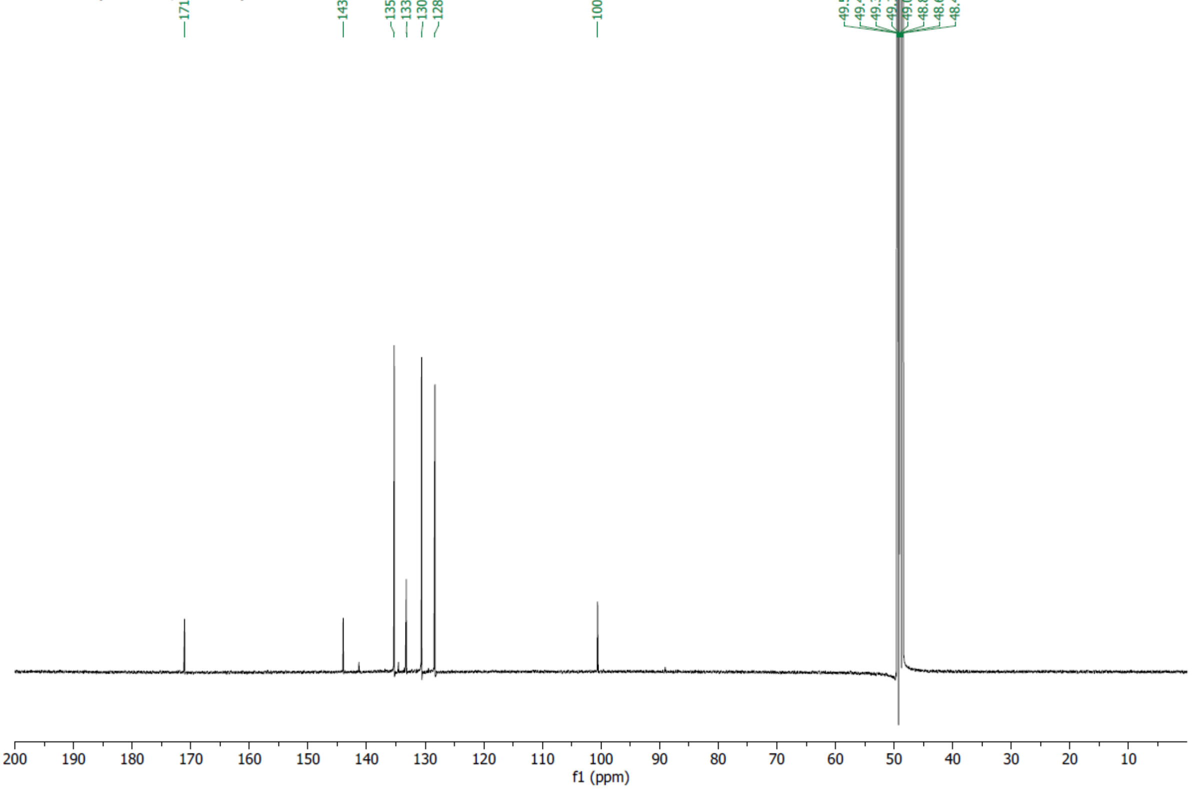

5-bromo-2-iodobenzoic acid (2af)

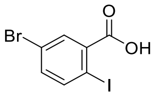

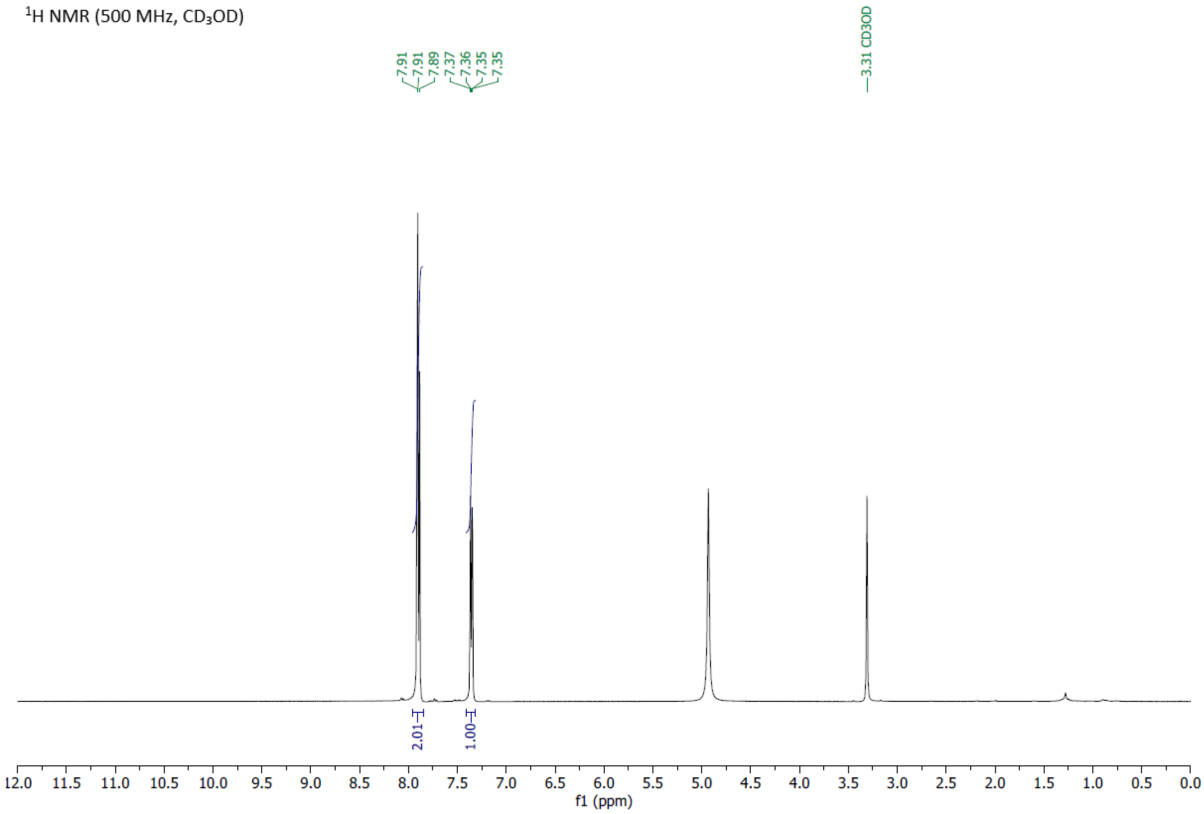

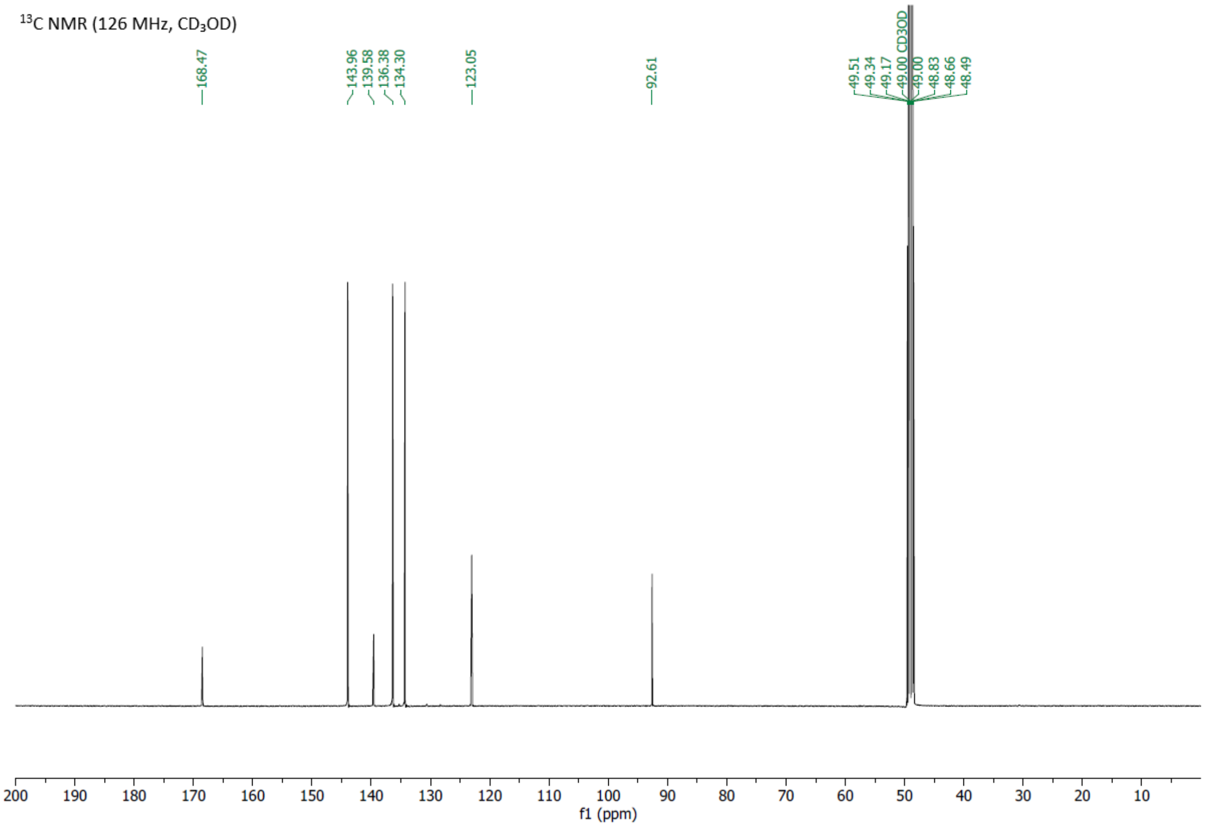

^{1}H NMR, ^{13}C NMR and ^{19}F of 4-(trifluoromethyl) benzoic-2,6-d2 acid ($1l_D$)

4-(trifluoromethyl) benzoic-2, 6-d2 acid ($1l_D$)

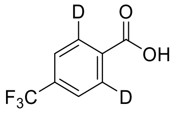

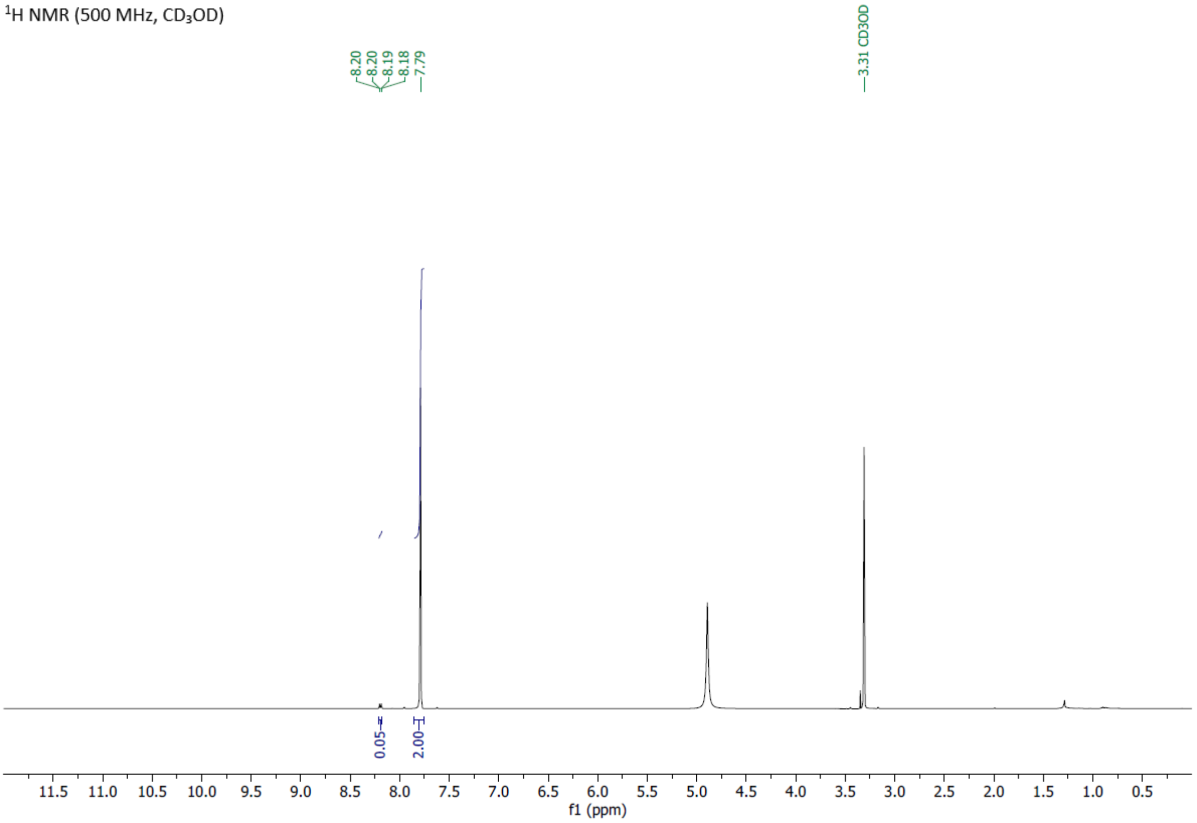

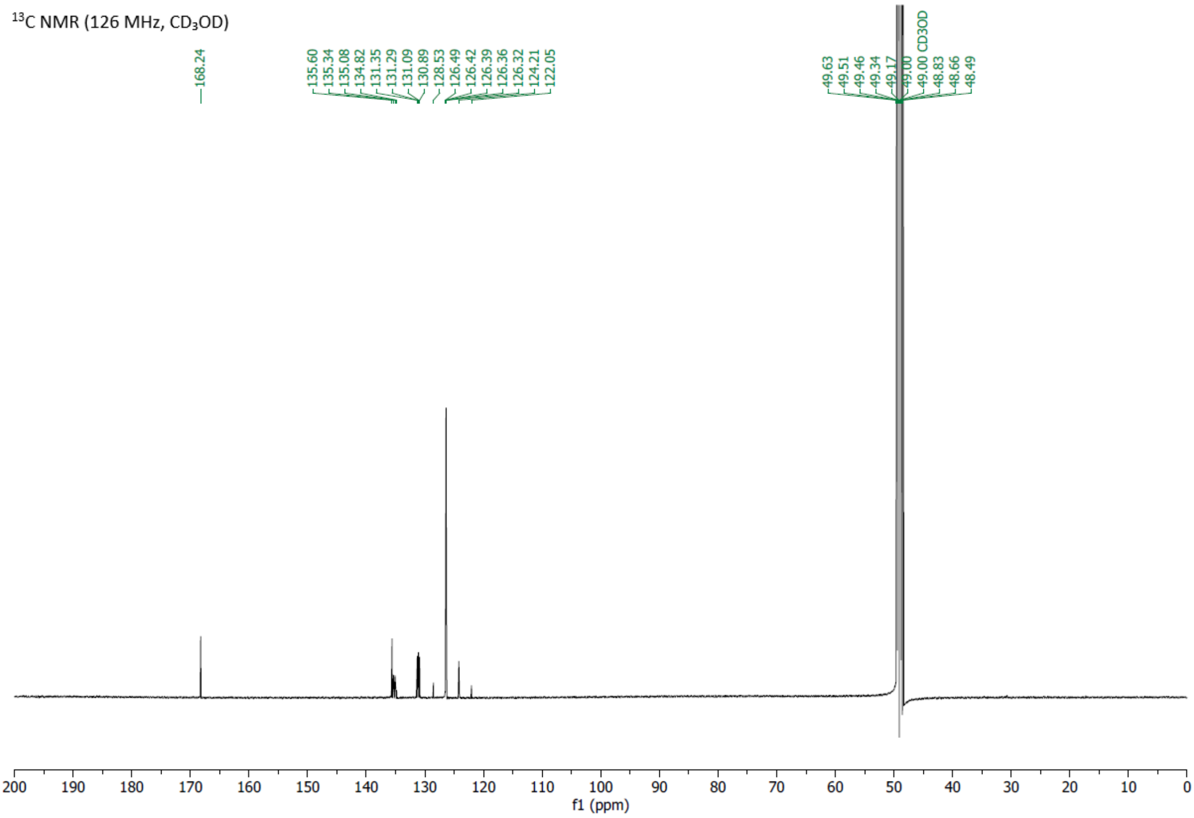

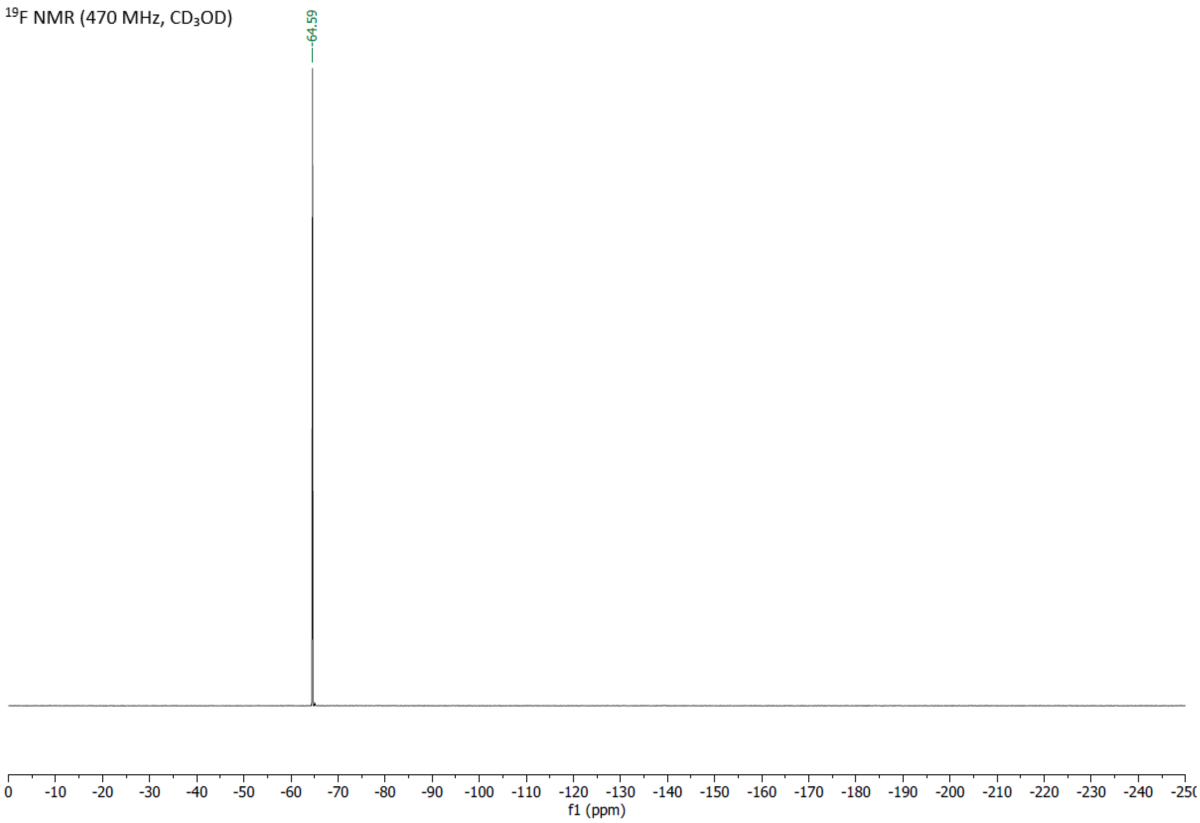

^{19}F NMR (470 MHz, CD$_3$OD)
-64.59
0 -10 -20 -30 -40 -50 -60 -70 -80 -90 -100 -110 -120 -130 -140 -150 -160 -170 -180 -190 -200 -210 -220 -230 -240 -250
f1 (ppm)

^{1}H NMR, ^{13}C NMR, ^{19}F and 2-D NMR spectra of $Cp^{*OCH(CF3)2}Ir(O_2CC_6H_4)$ (Ic-2)

$Cp^{*OCH(CF3)2}Ir(O_2CC_6H_4)$ (Ic-2)

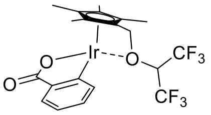

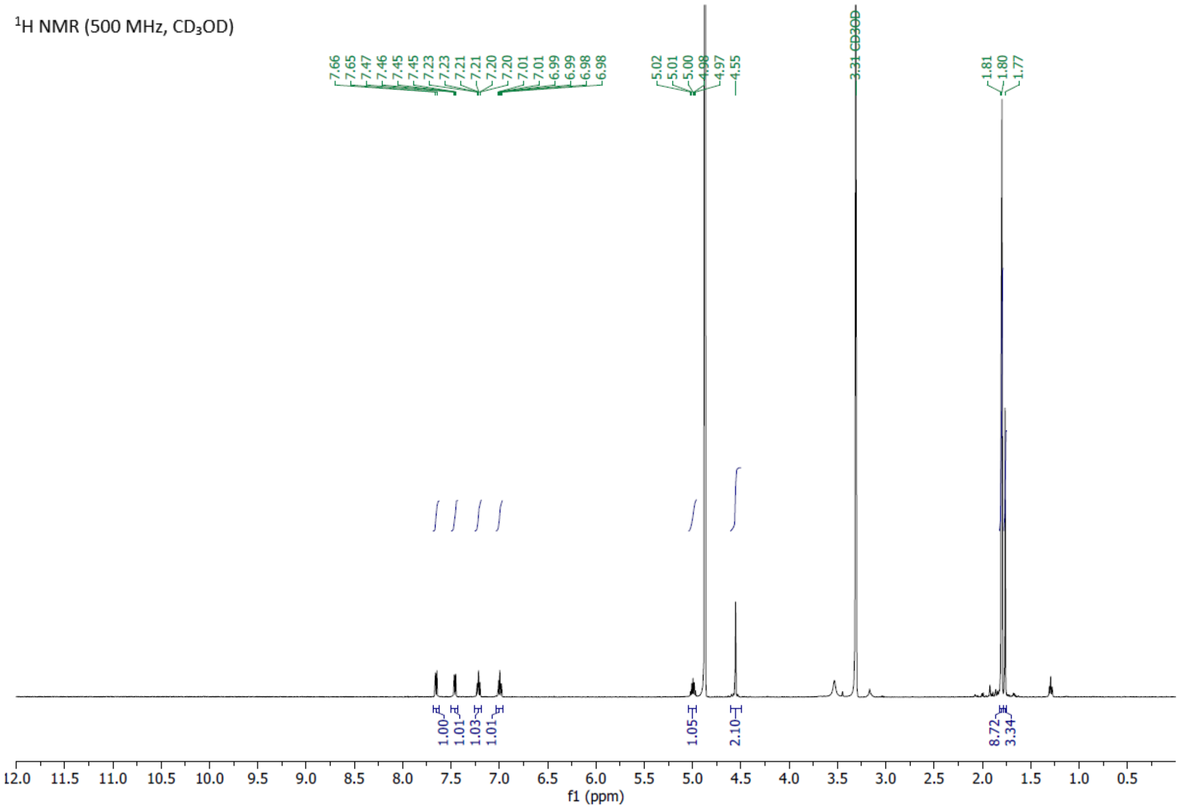

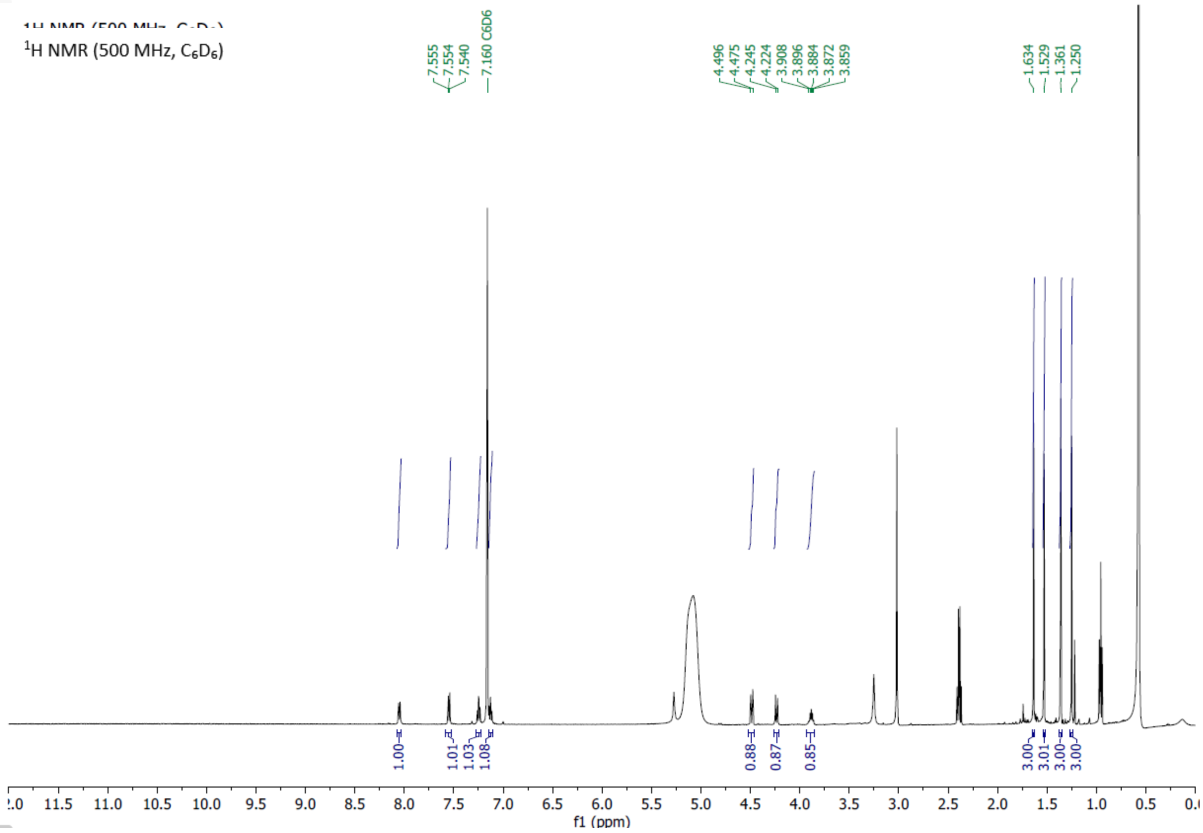

^{13}C NMR (126 MHz, C_6D_6)

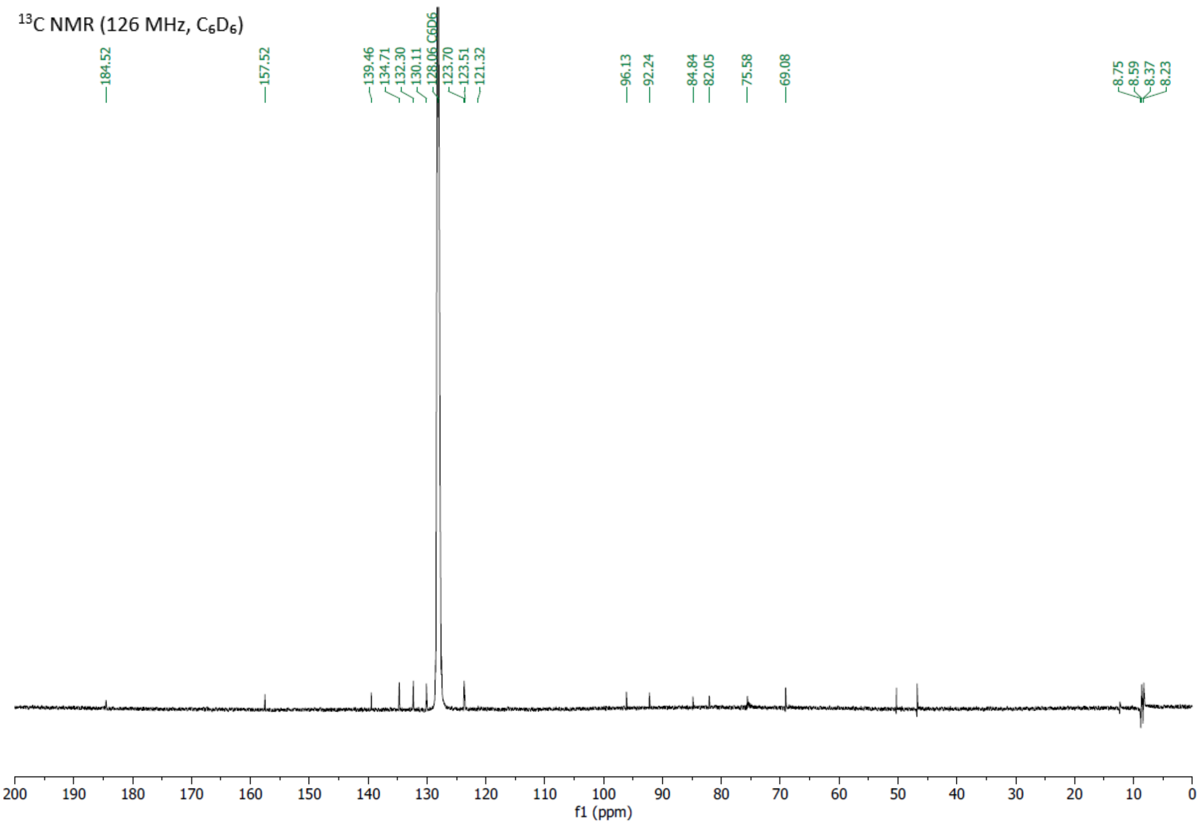

^{19}F NMR (470 MHz, C_6D_6)

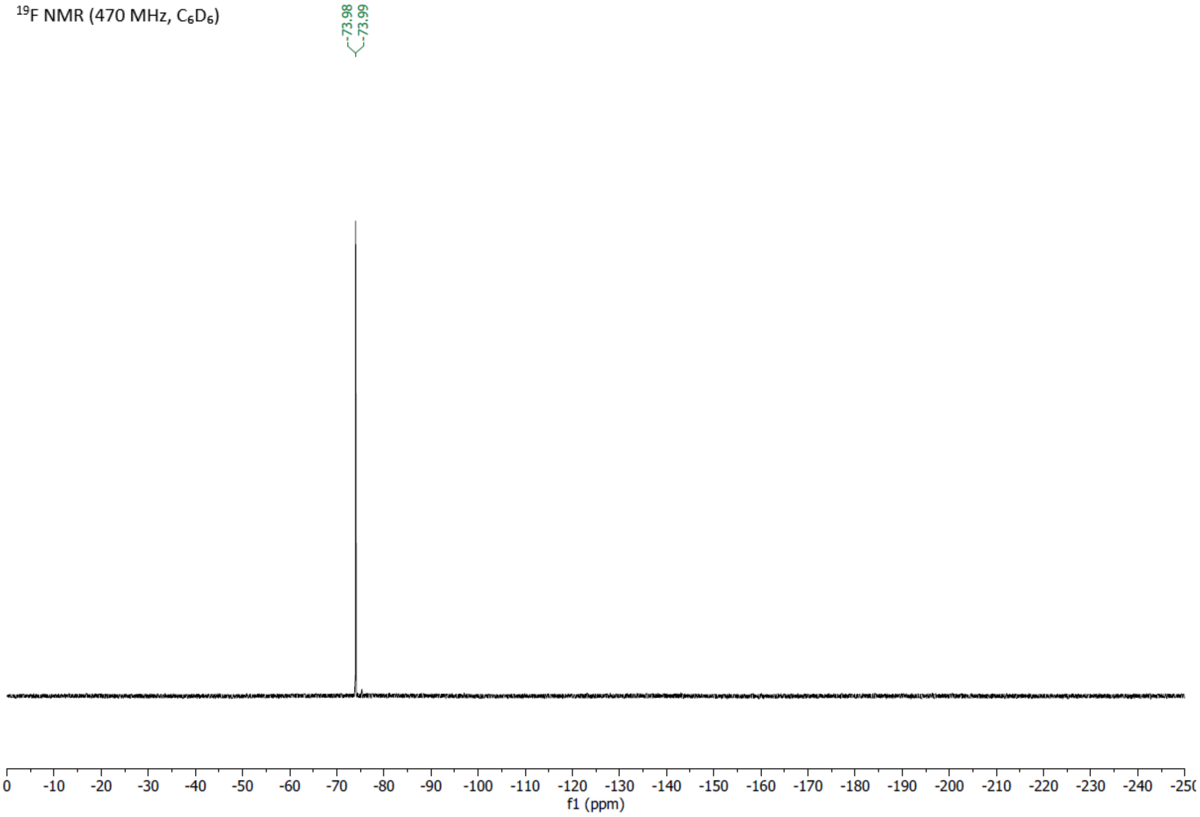

HSQC NMR (500 MHz, C_6D_6)

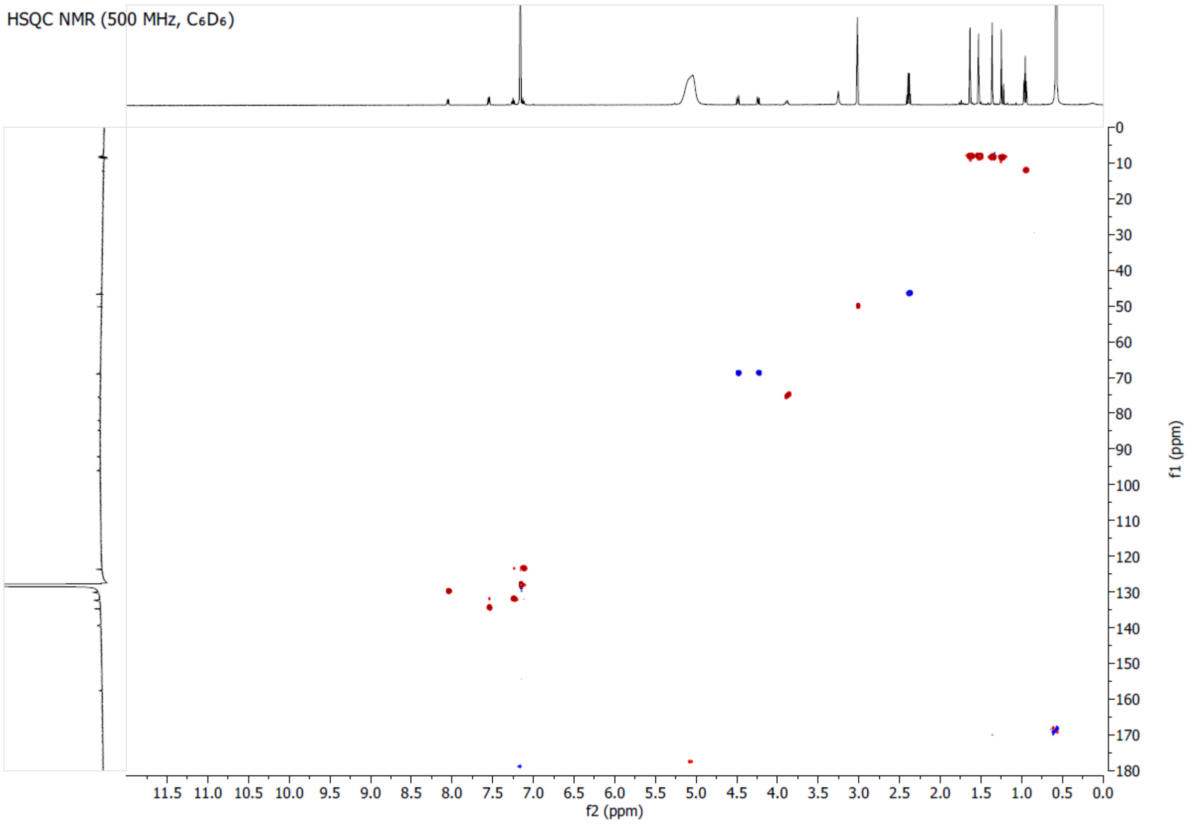

HMBC NMR (500 MHz, C_6D_6)

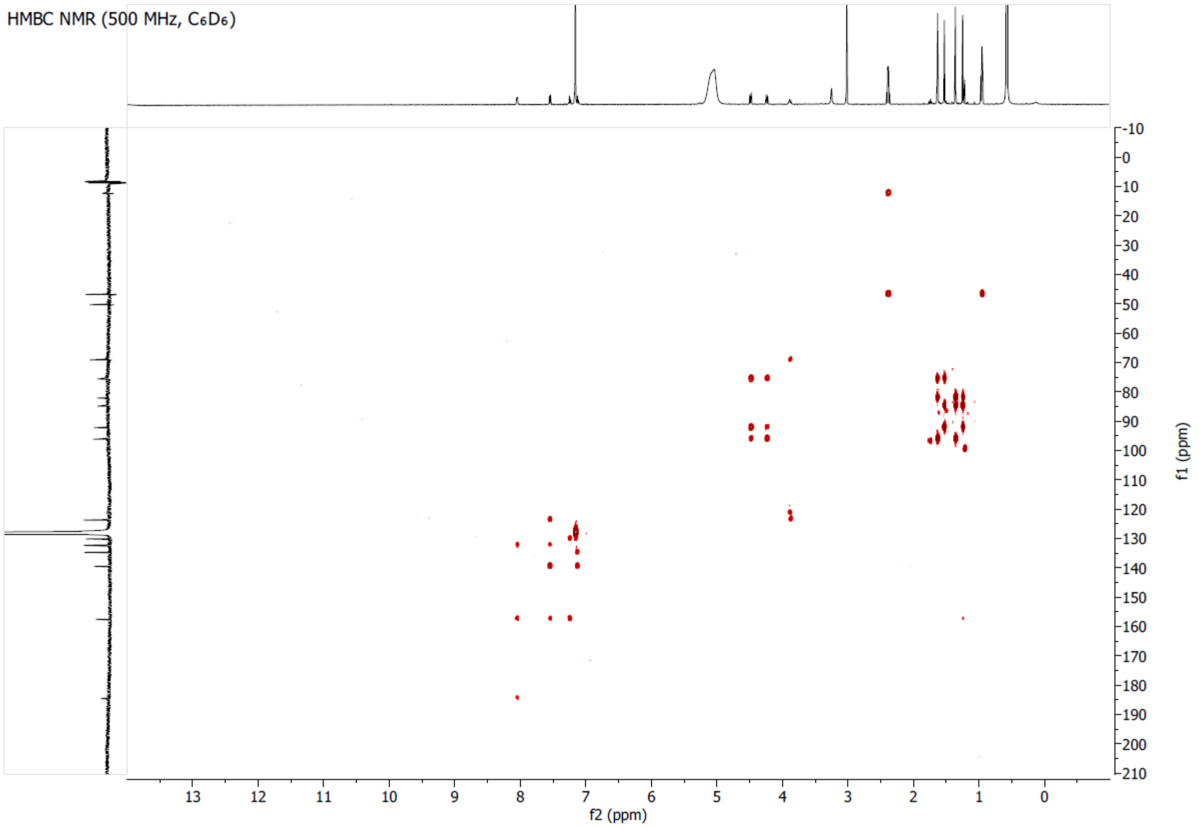

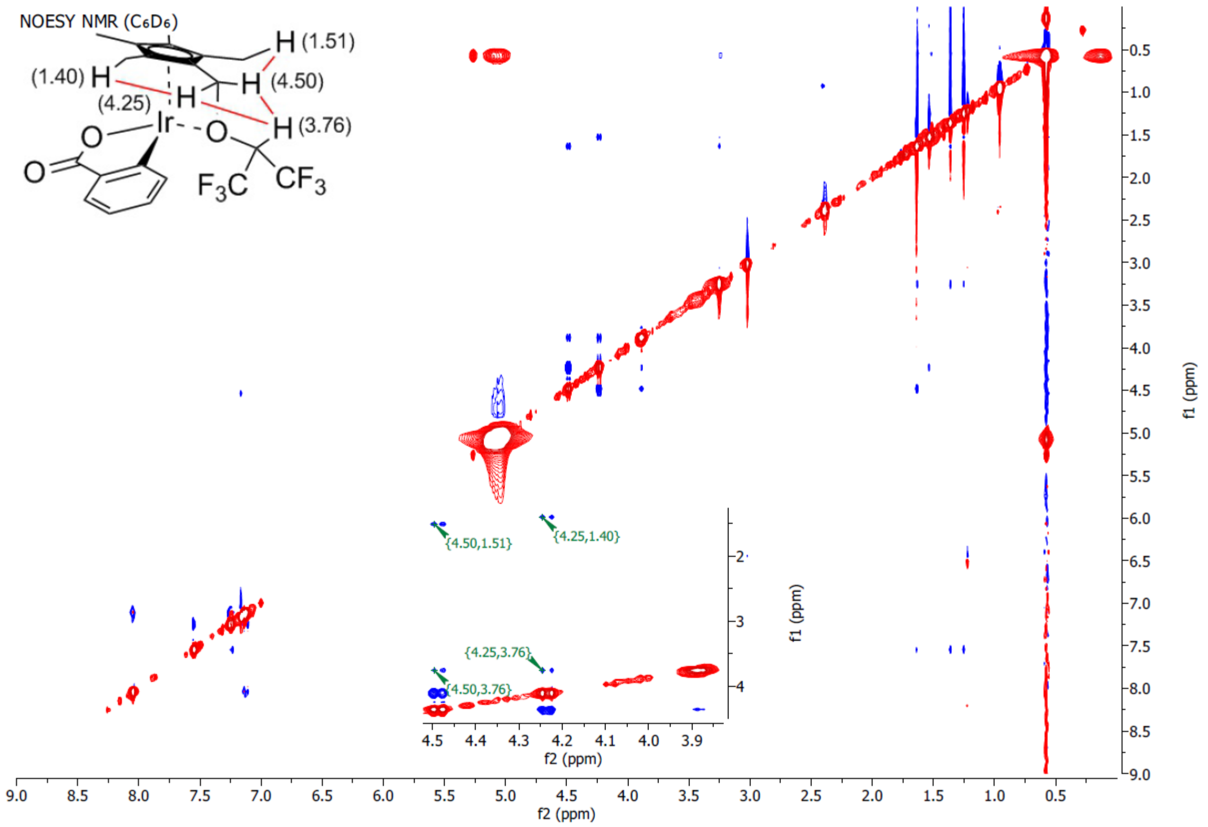
NOESY NMR (C6D6)
H (1.51)
(1.40) H
H (4.50)
(4.25)
H (3.76)
Ir
O
CF3
F3C
{4.50,1.51}
{4.25,1.40}
{4.25,3.76}
{4.50,3.76}
f1 (ppm)
f2 (ppm)
